# Supplementary material for: From 18- to 20-electron ferrocene derivatives via ligand coordination
Source: Nat Commun. 2025 Jul 7;16:6124. doi: 10.1038/s41467-025-61343-7 (PMC12234732; doi:10.1038/s41467-025-61343-7)
Supplement: Supplementary file 1 — Supplementary Information [file 41467_2025_61343_MOESM1_ESM.pdf]

Supplementary Information for

From 18- to 20-electron ferrocene derivatives via ligand coordination

Satoshi Takebayashi, Jama Ariai, Sergey V. Kartashov, Robert R. Fayzullin, Tomoko Onoue, Ko Mibu, Hyung-Been Kang, and Noriko Ishizu

Correspondence to: satoshi.takebayashi@oist.jp, robert.fayzullin@gmail.com

Table of Contents

Supplementary Figures 2

Supplementary Figures 1-54. NMR, IR, and EPR spectra, and VSM data 2

Supplementary Notes 51

Supplementary Note 1. Preparation of ligands 51
Supplementary Note 2. Preparation and reactivity of complexes 58
Supplementary Note 3. Mössbauer spectroscopy 71
Supplementary Note 4. X-ray photoelectron spectroscopy 75
Supplementary Note 5. Computational study 76
Supplementary Note 6 .Quantum topological study 86
Supplementary Note 7. SC-XRD study 91

Supplementary References 109

Supplementary Figures

a

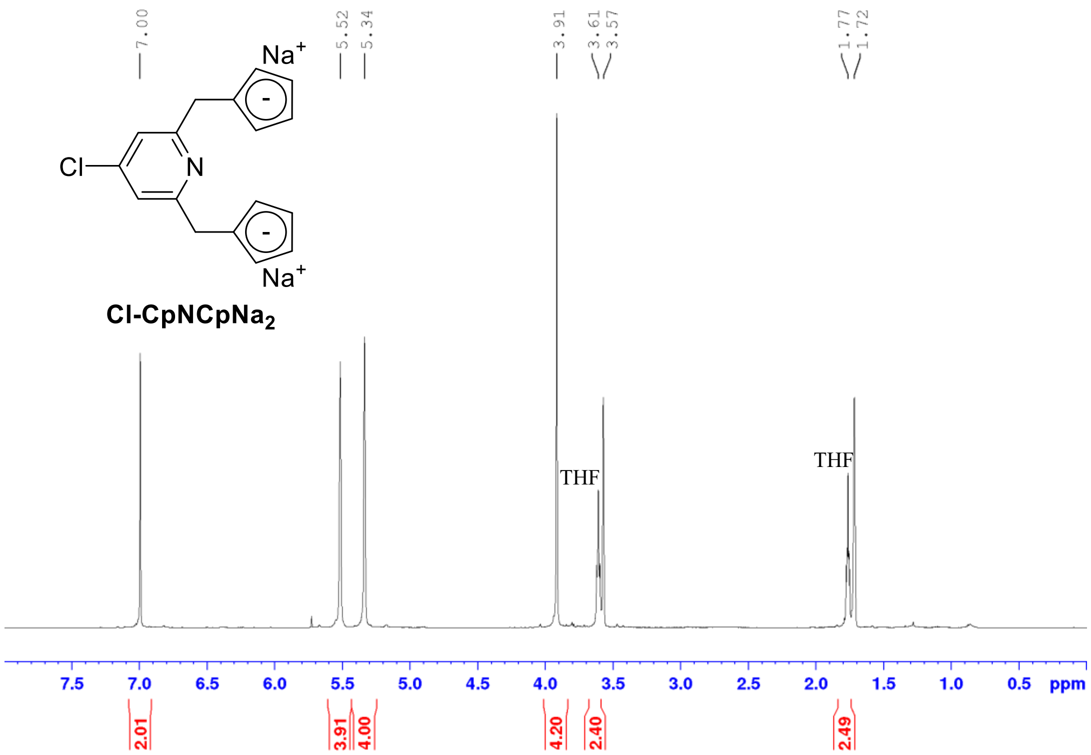

b

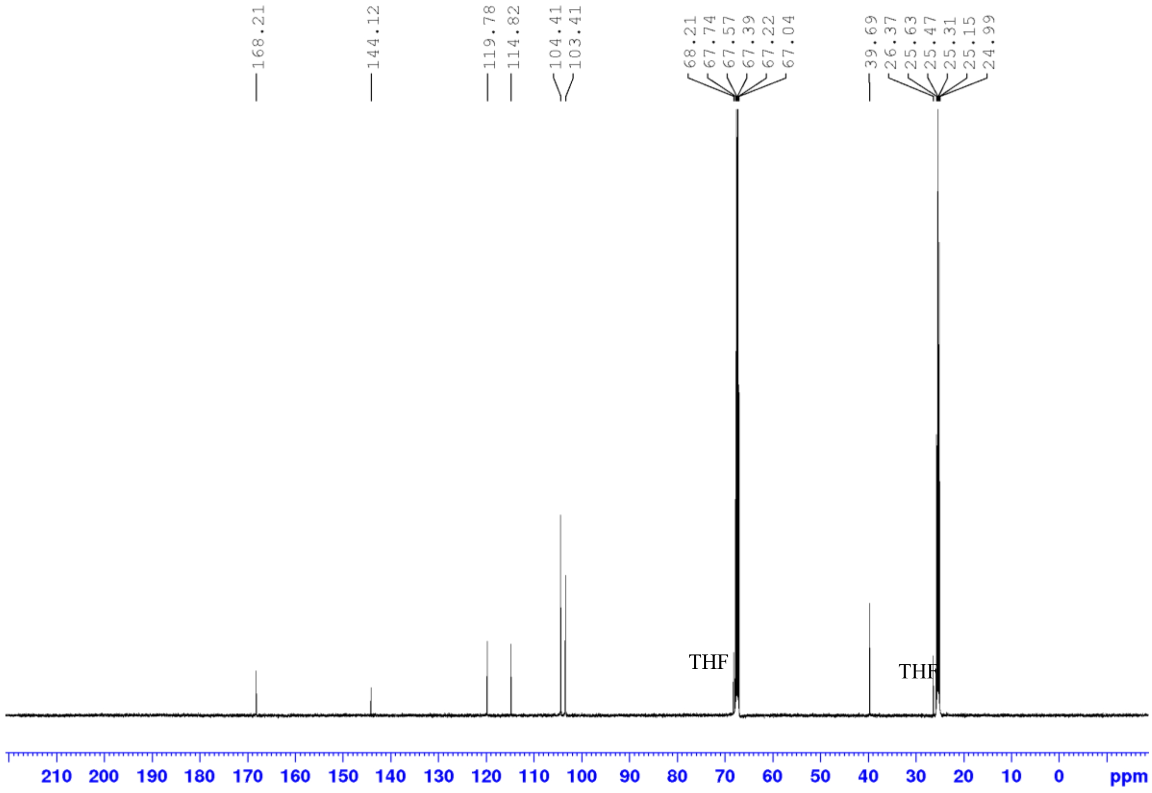

Supplementary Fig. 1.
NMR spectra (THF-d_8, 298 K) of **Cl-CpNCpNa$_2$**. (**a**) ^{1}H NMR (500.13 MHz). (**b**) ^{13}C{^{1}H} NMR (125.76 MHz).

a

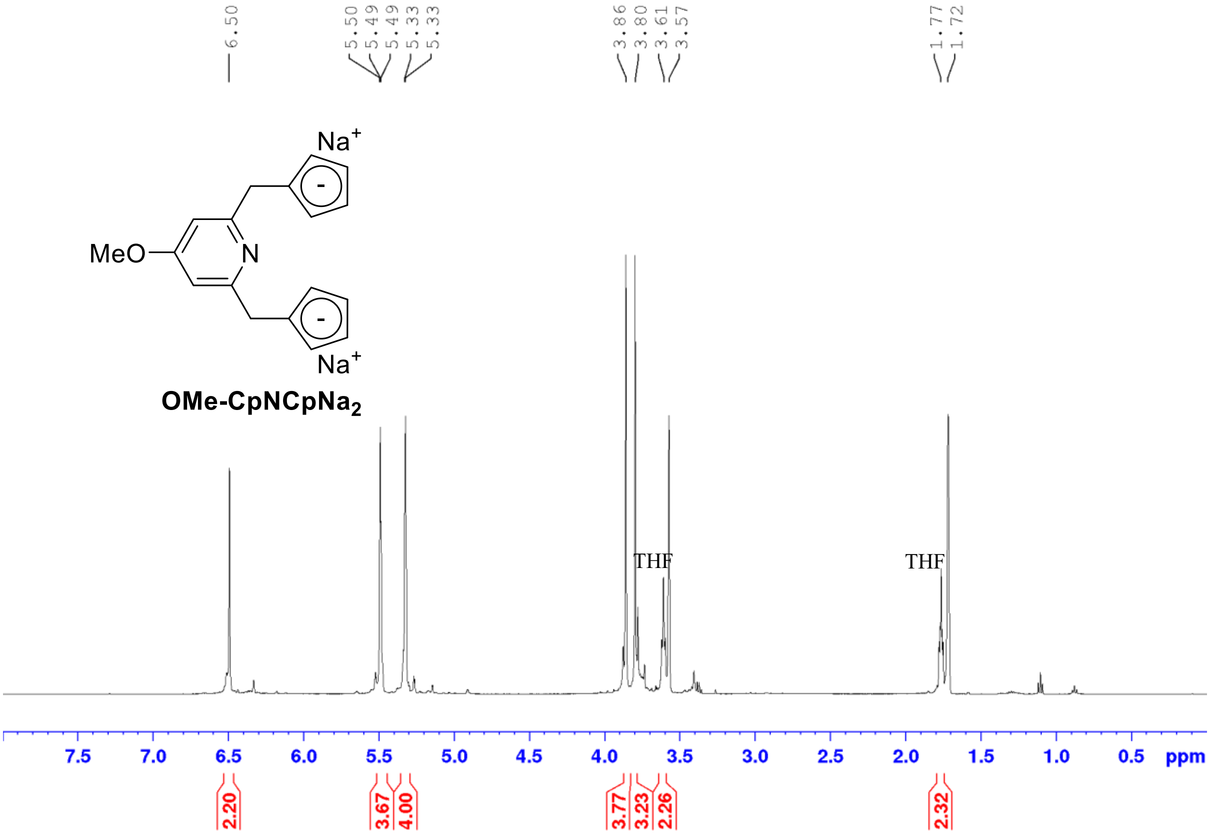

b

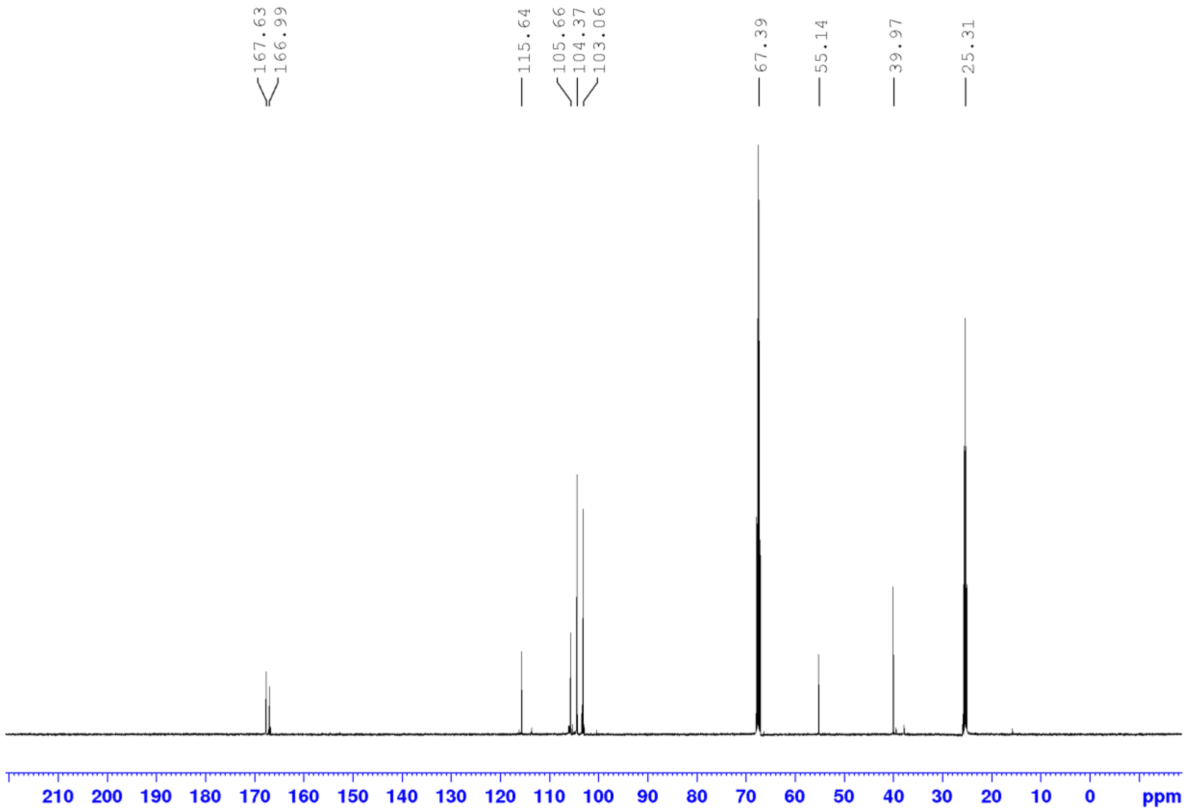

Supplementary Fig. 2.

NMR spectra (THF-d_8, 298 K) of **OMe-CpNCpNa$_2$**. (**a**) ^{1}H NMR (500.13 MHz). (**b**) $^{13}C\{^1H\}$ NMR (125.76 MHz).

a

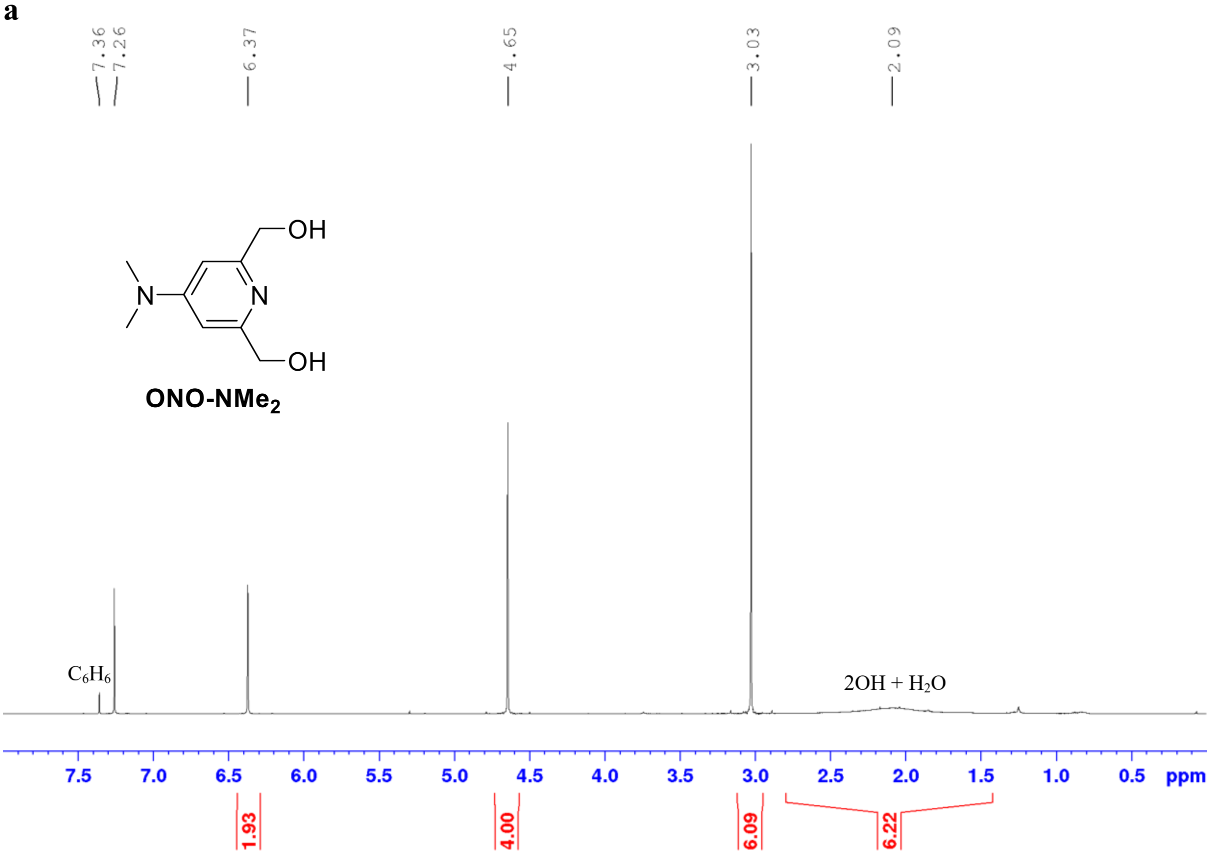

b

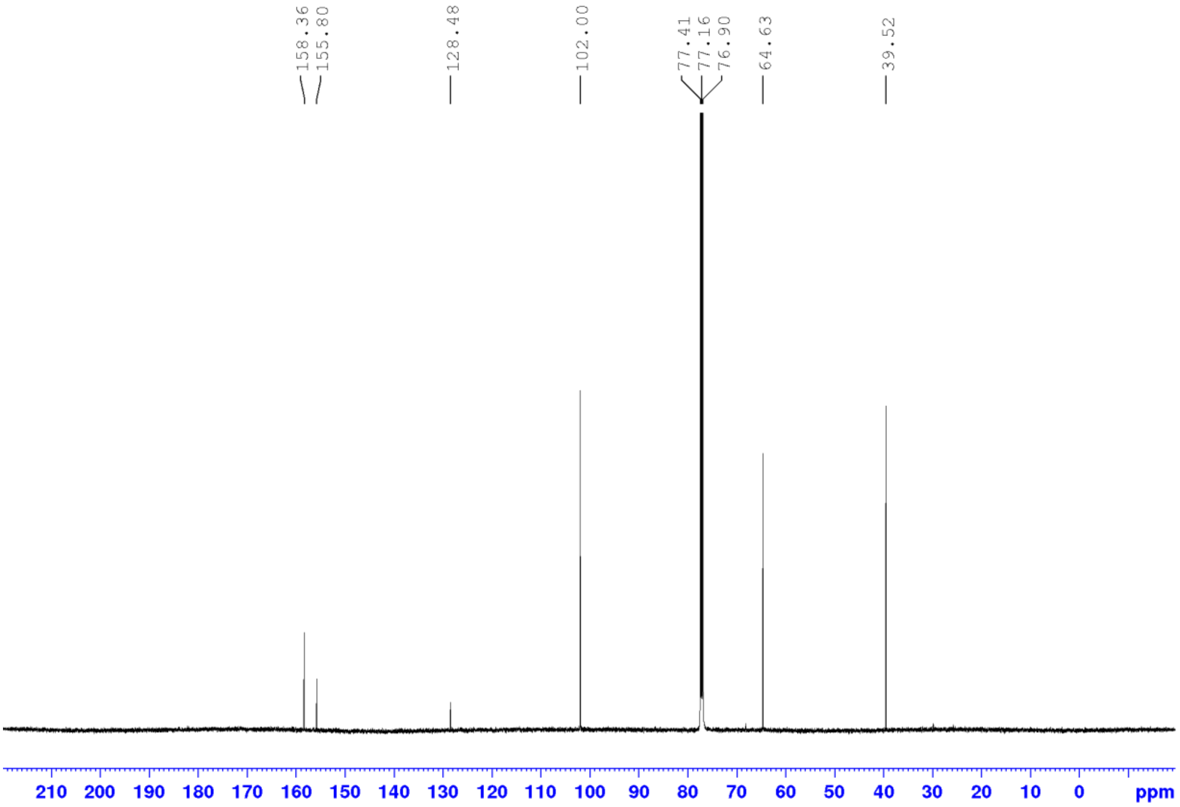

Supplementary Fig. 3.

NMR spectra ($CDCl_3$, 298 K) of **ONO-NMe2** (**a**) ^1H NMR (500.13 MHz). (**b**) $^{13}C\{^1H\}$ NMR (125.76 MHz).

a

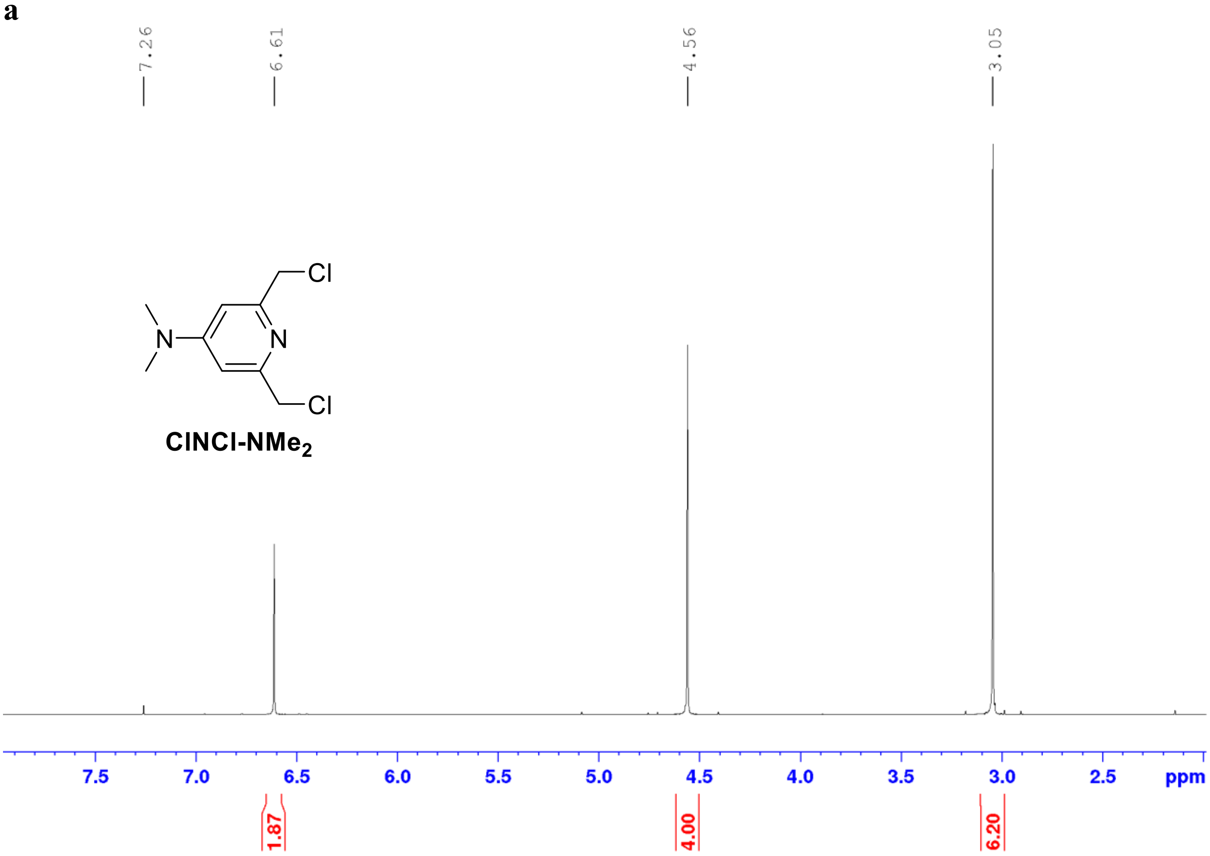

b

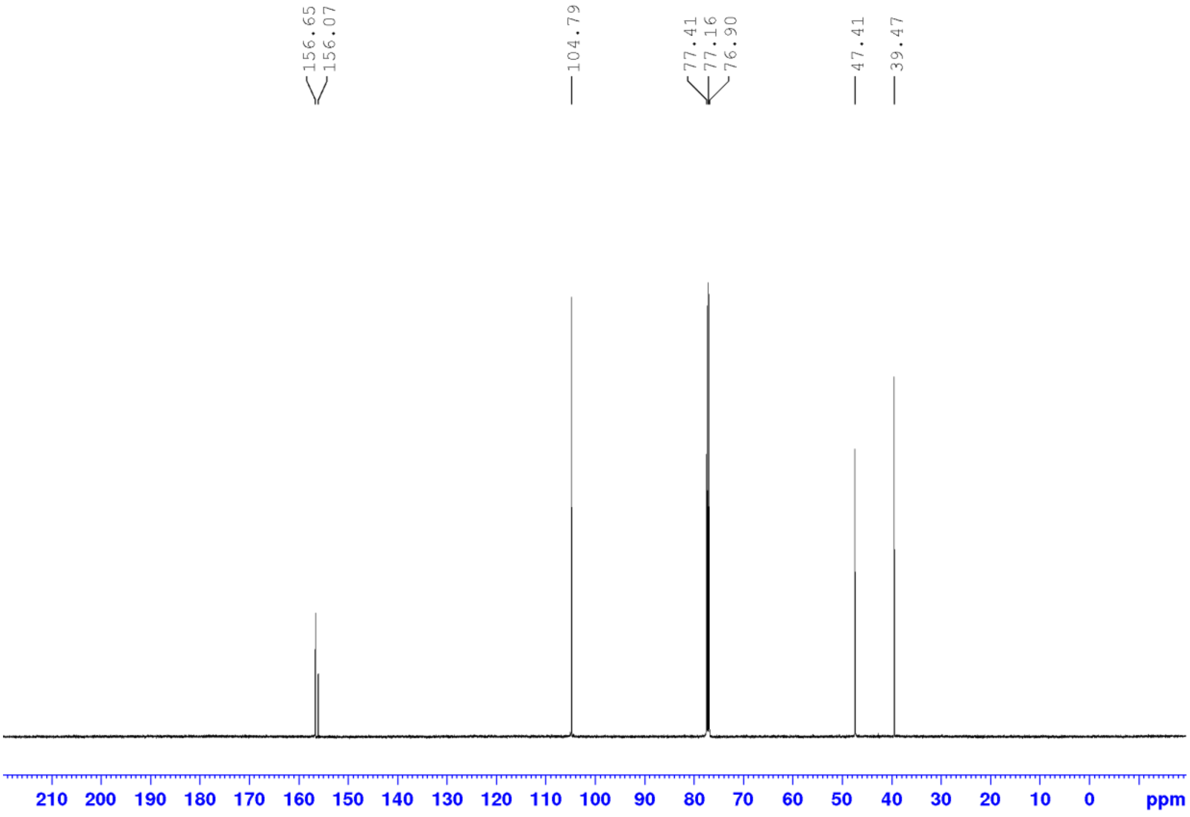

Supplementary Fig. 4.

NMR spectra ($CDCl_3$, 298 K) of **ClNCl-NMe₂** (**a**) ^{1}H NMR (500.13 MHz). (**b**) $^{13}C\{^1H\}$ NMR (125.76 MHz).

a

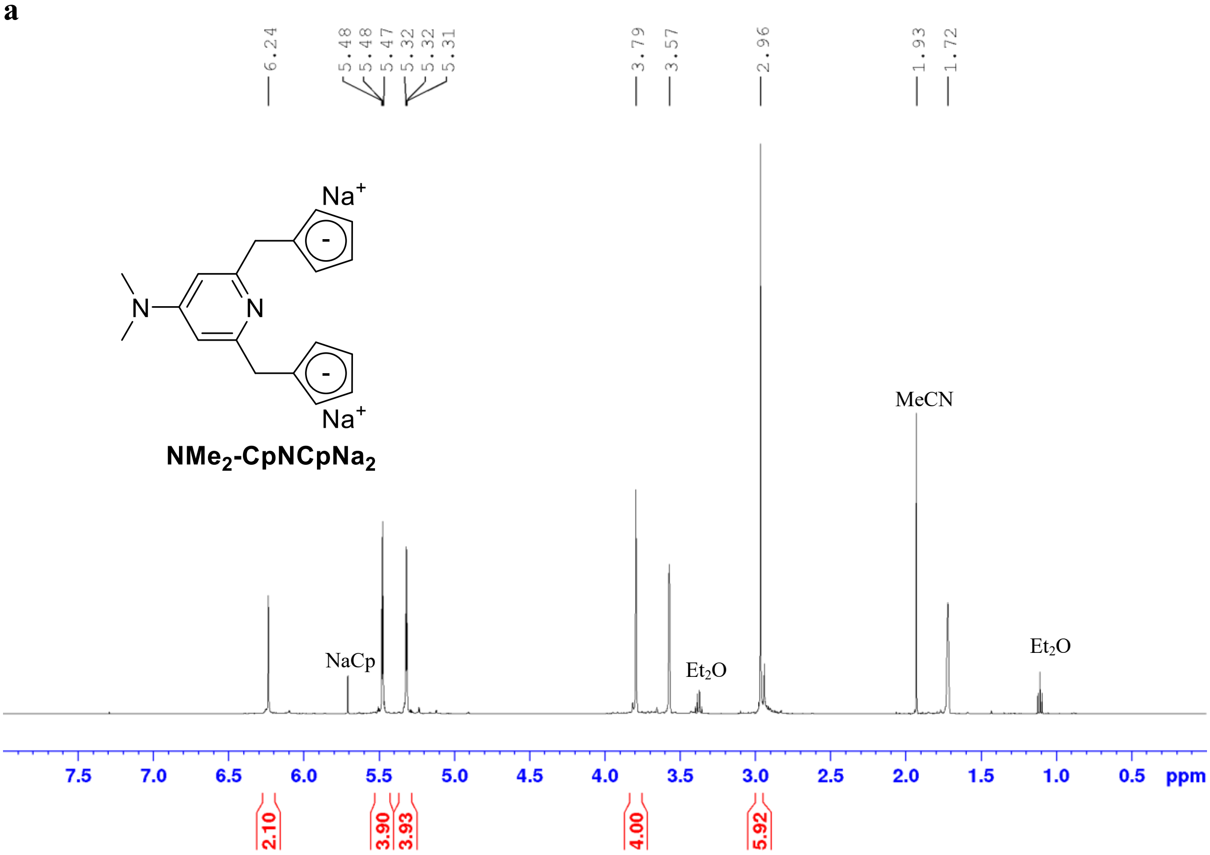

b

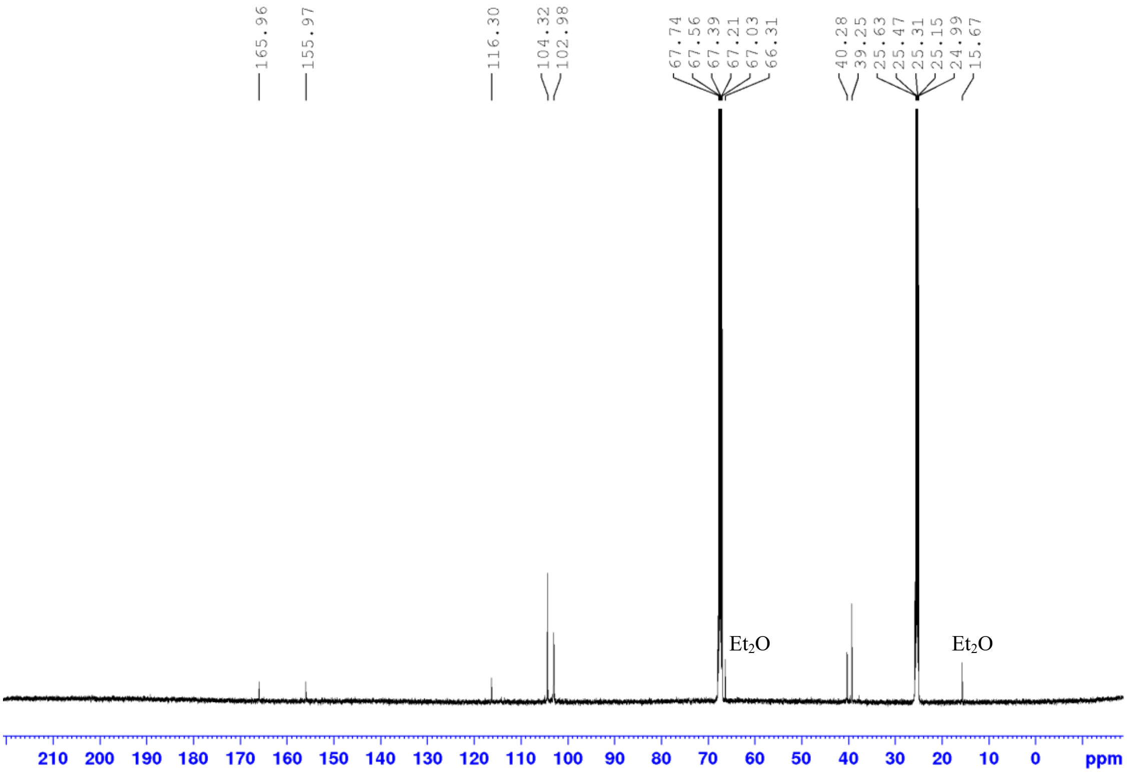

Supplementary Fig. 5.

NMR spectra (THF-d_8, 298 K) of **NMe$_2$-CpNCpNa$_2$**. (**a**) ^{1}H NMR (500.13 MHz). (**b**) ^{13}C{^{1}H} NMR (125.76 MHz).

a

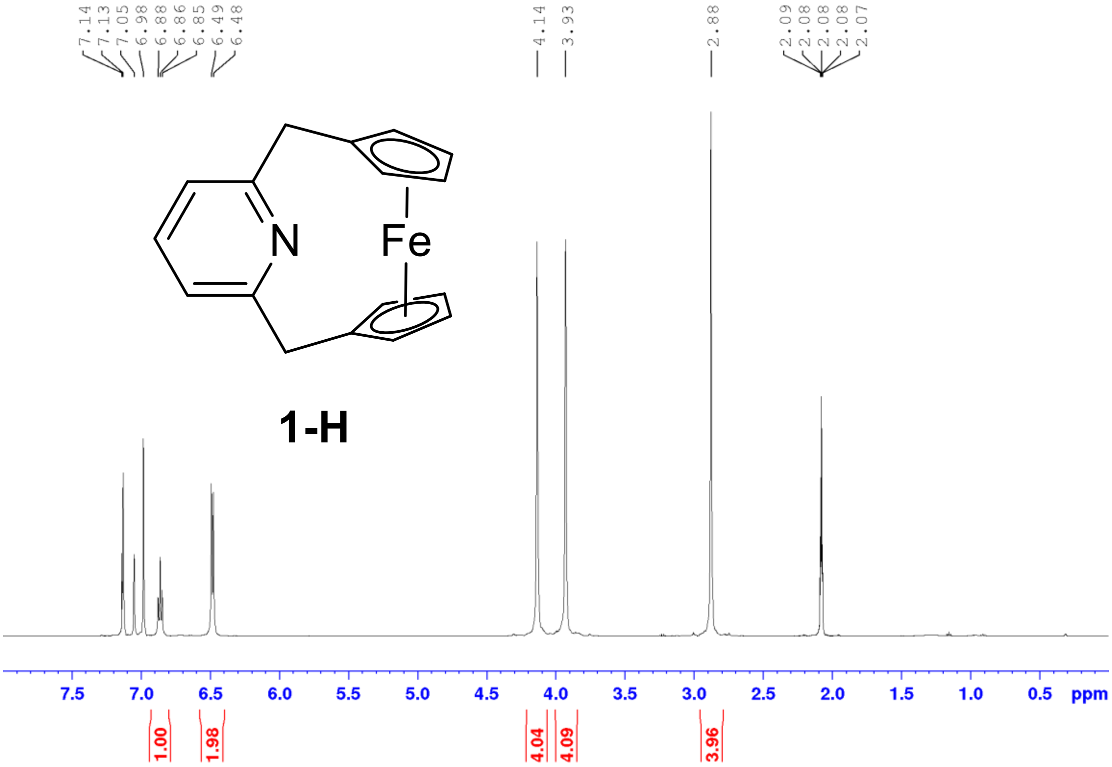

b

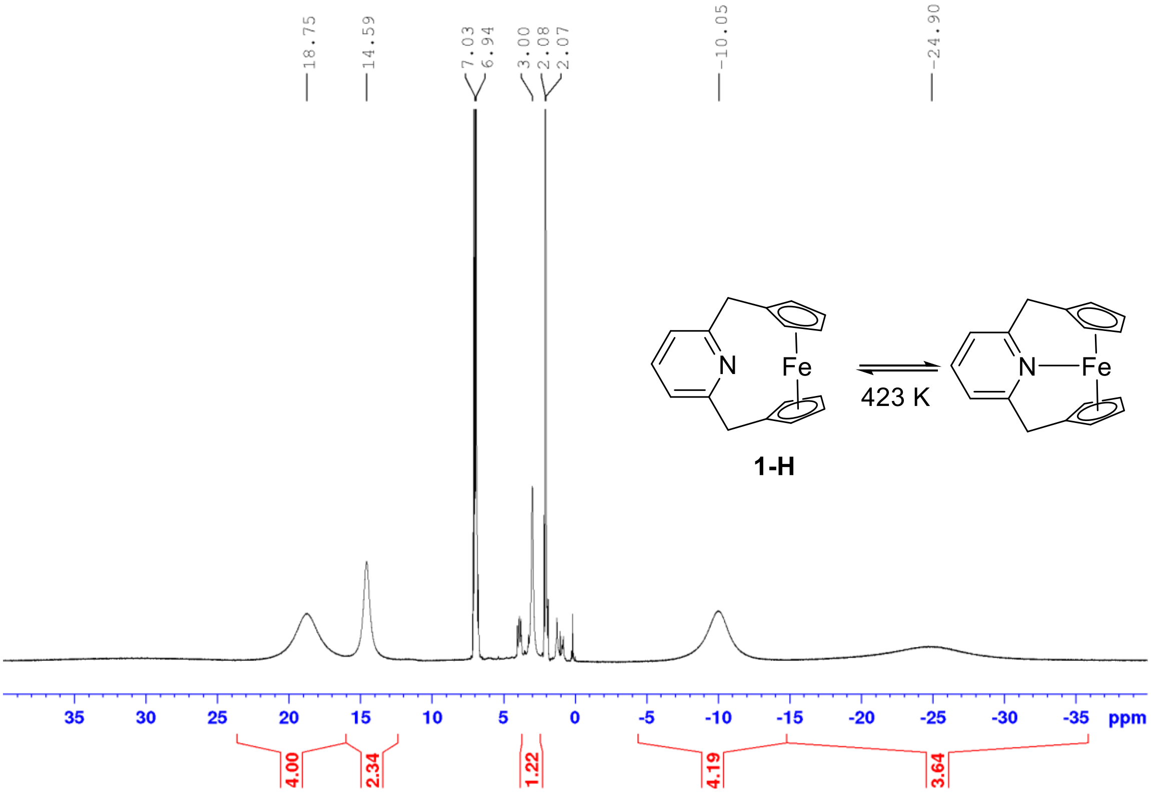

Supplementary Fig. 6.
^{1}H NMR spectra (Toluene-d_8, 500.13 MHz) of **1-H**. (**a**) At 233 K. (**b**) At 423 K, under 5 bar N_2.

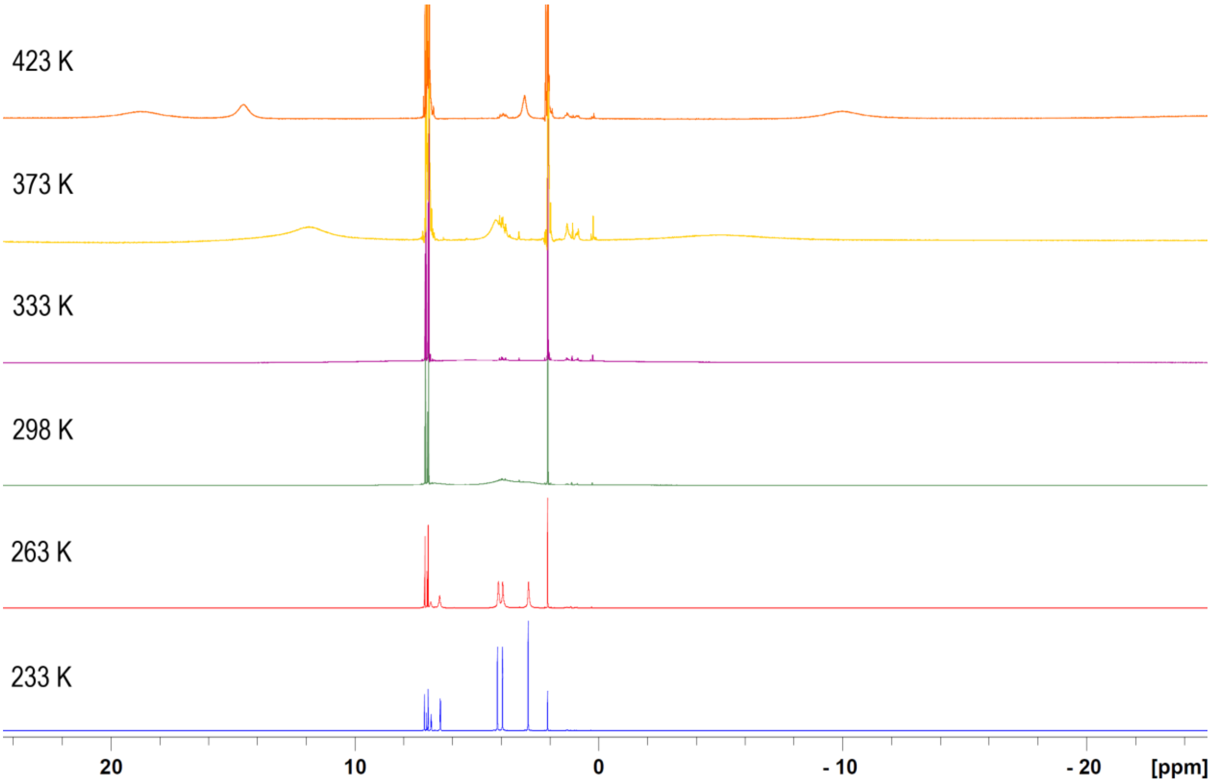

Supplementary Fig. 7.
Variable temperature ^{1}H NMR spectra (Toluene-d_8, 500.13 MHz, 233 to 423 K) of **1-H**.

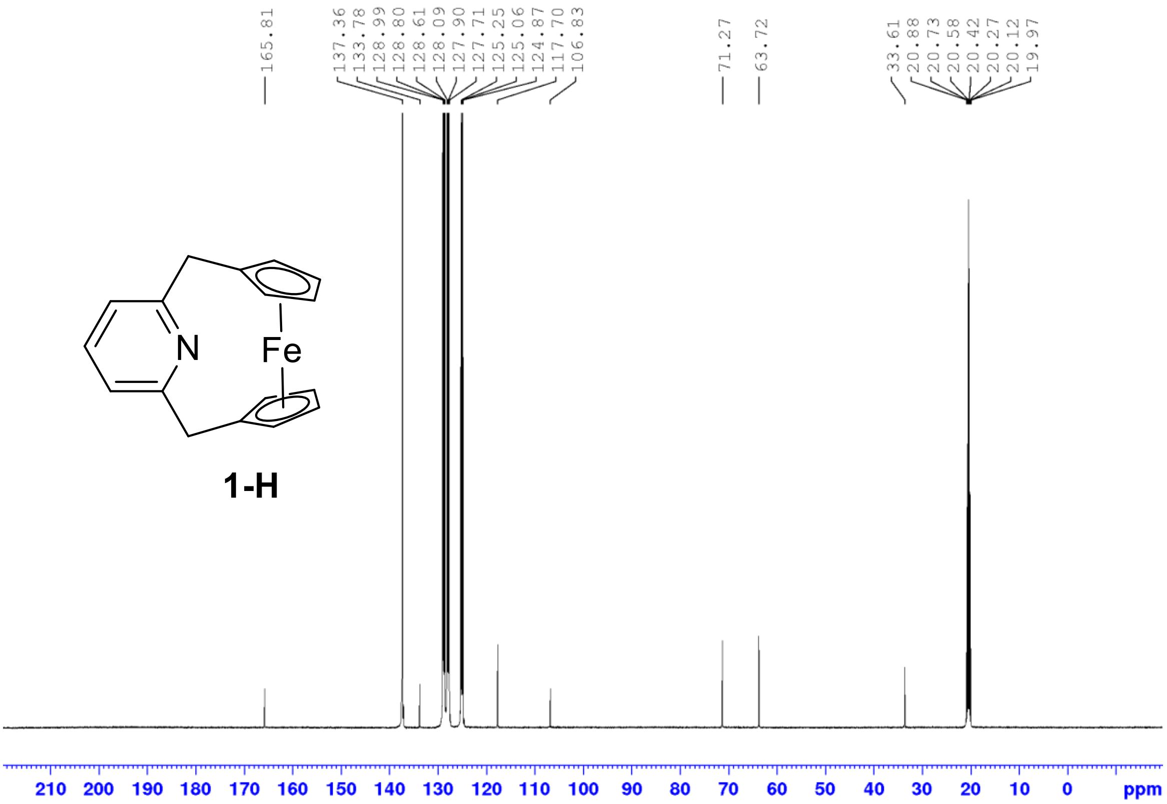

Supplementary Fig. 8.
$^{13}C\{^{1}H\}$ NMR spectrum (Toluene-d_8, 125.76 MHz, 233 K) of **1-H**.

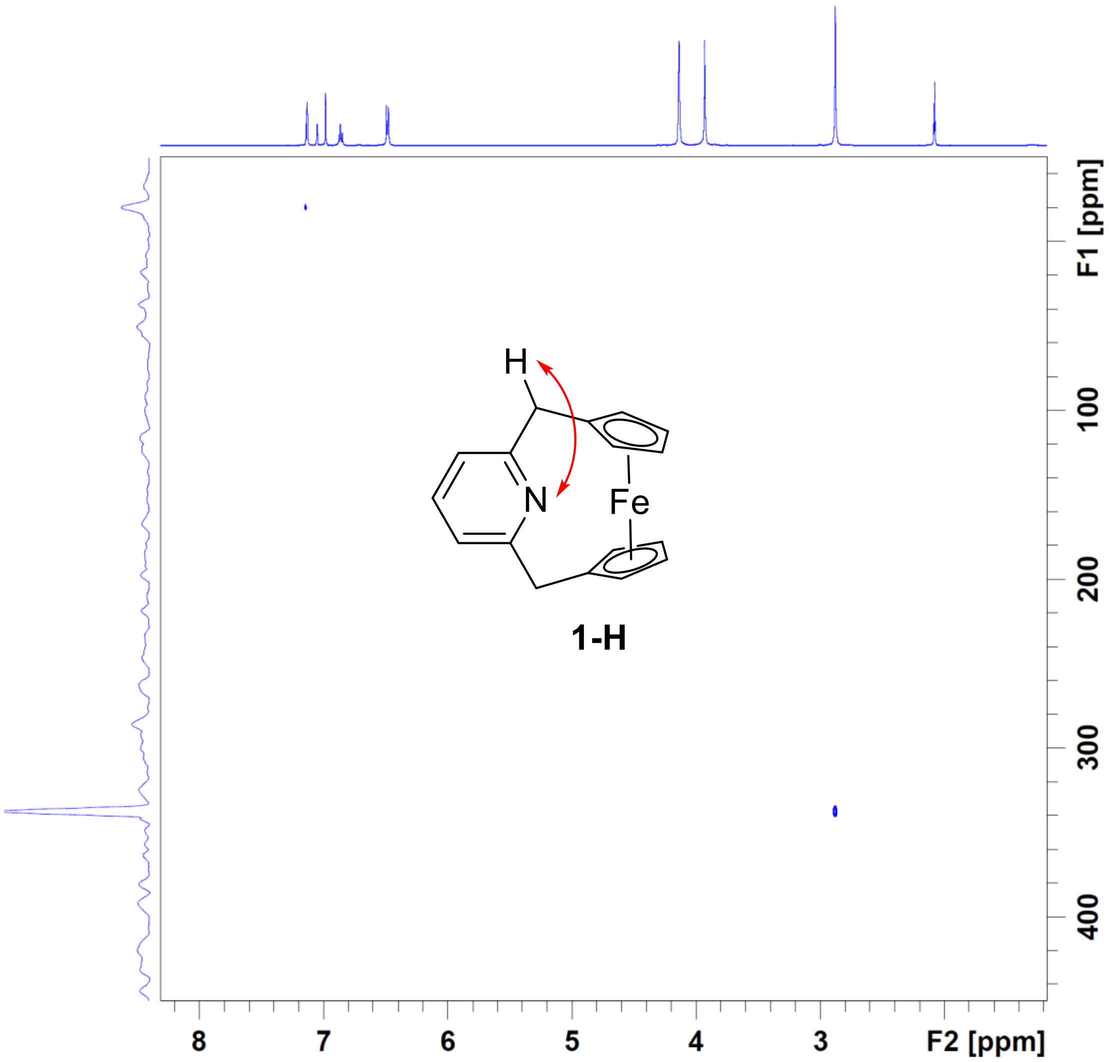

Supplementary Fig. 9.
^{1}H-^{15}N HMBC NMR spectrum (Toluene-d_8, 500.13 MHz, 233 K) of **1-H**.

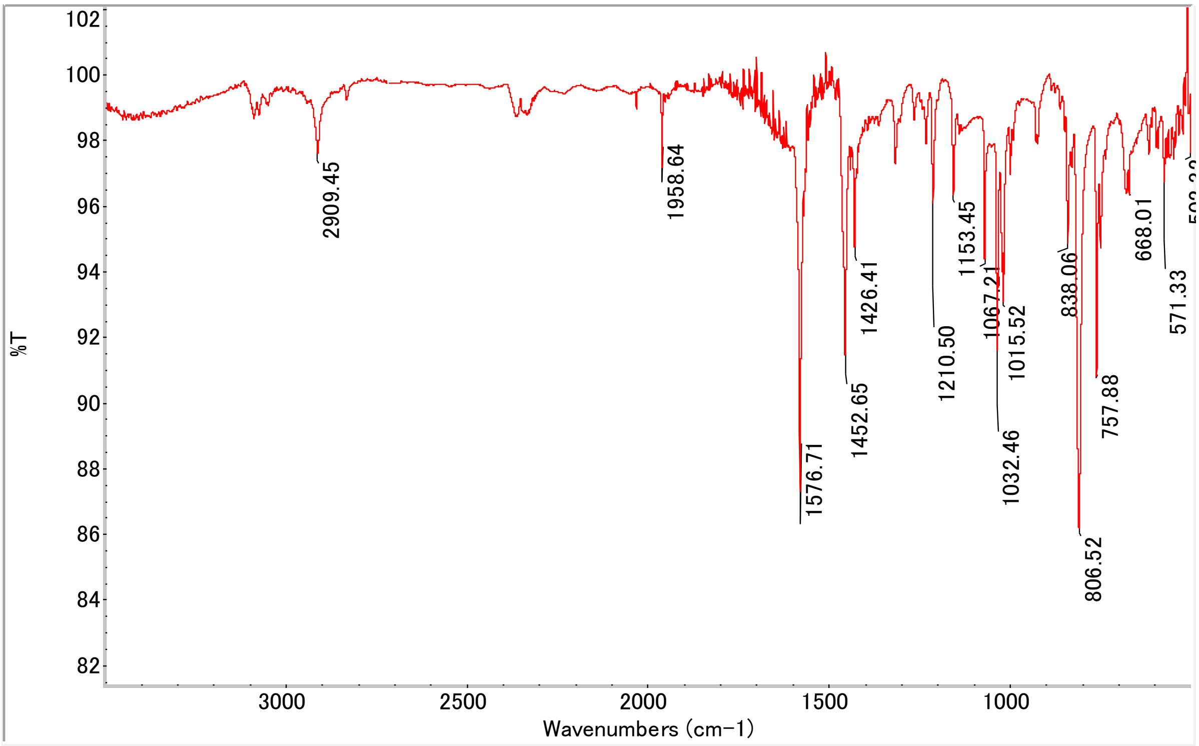

Supplementary Fig. 10.
FTIR spectrum (thin film) of **1-H**.

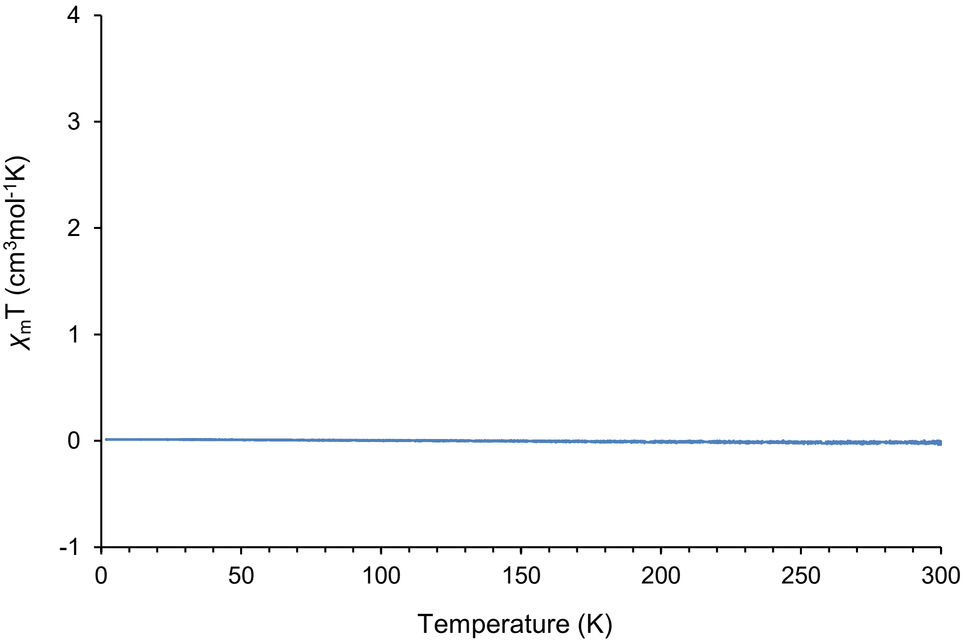

Supplementary Fig. 11.
χT vs. T plot based on VSM measurement of a bulk solid of **1-H** at 2-300 K under 1000 Oe.

a

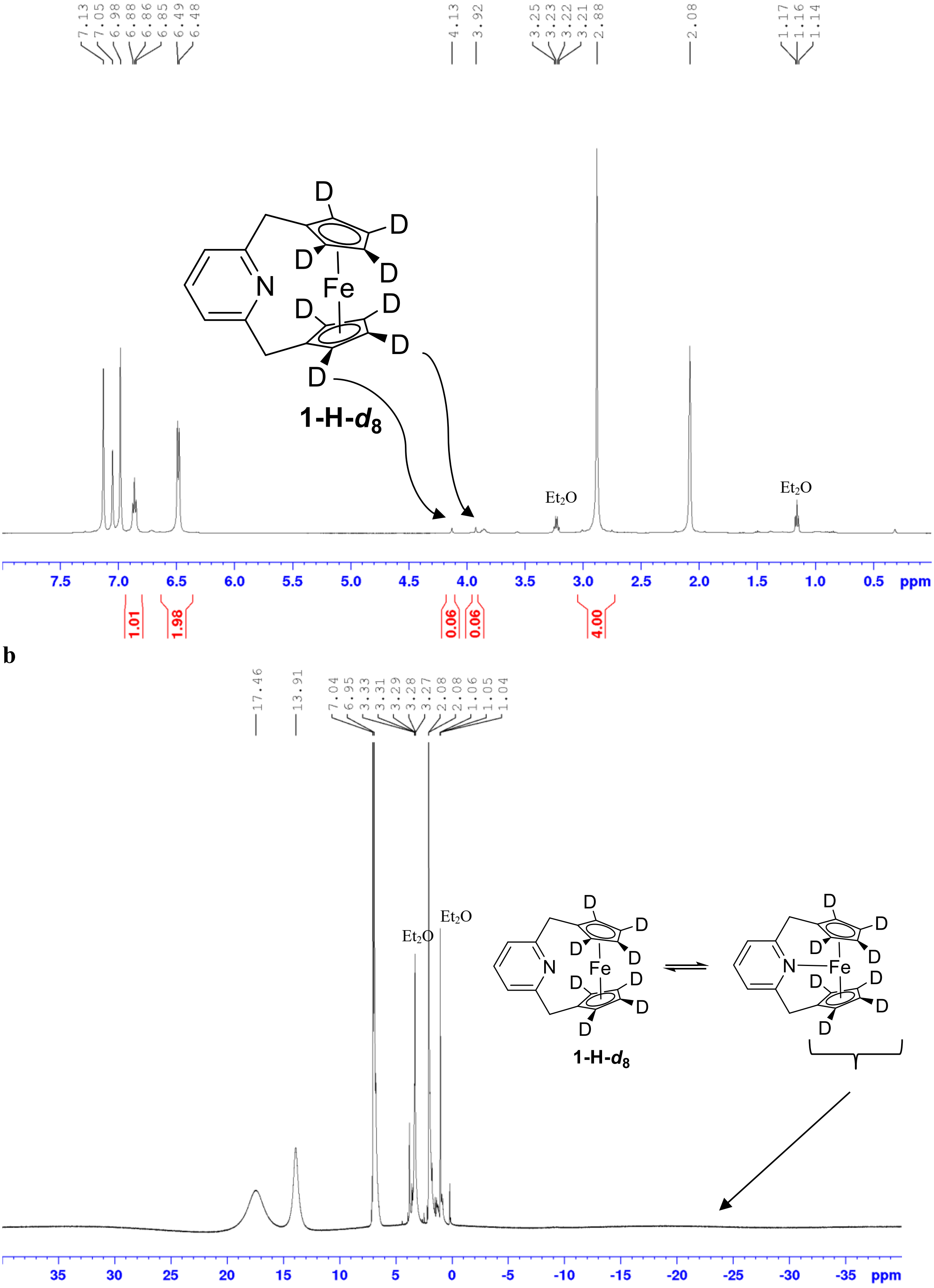

Supplementary Fig. 12.

^{1}H NMR spectra (Toluene-d_8, 500.13 MHz,) of **1-H-d_8**. (**a**) At 233 K. (**b**) At 423 K, under 5 bar N_2.

a

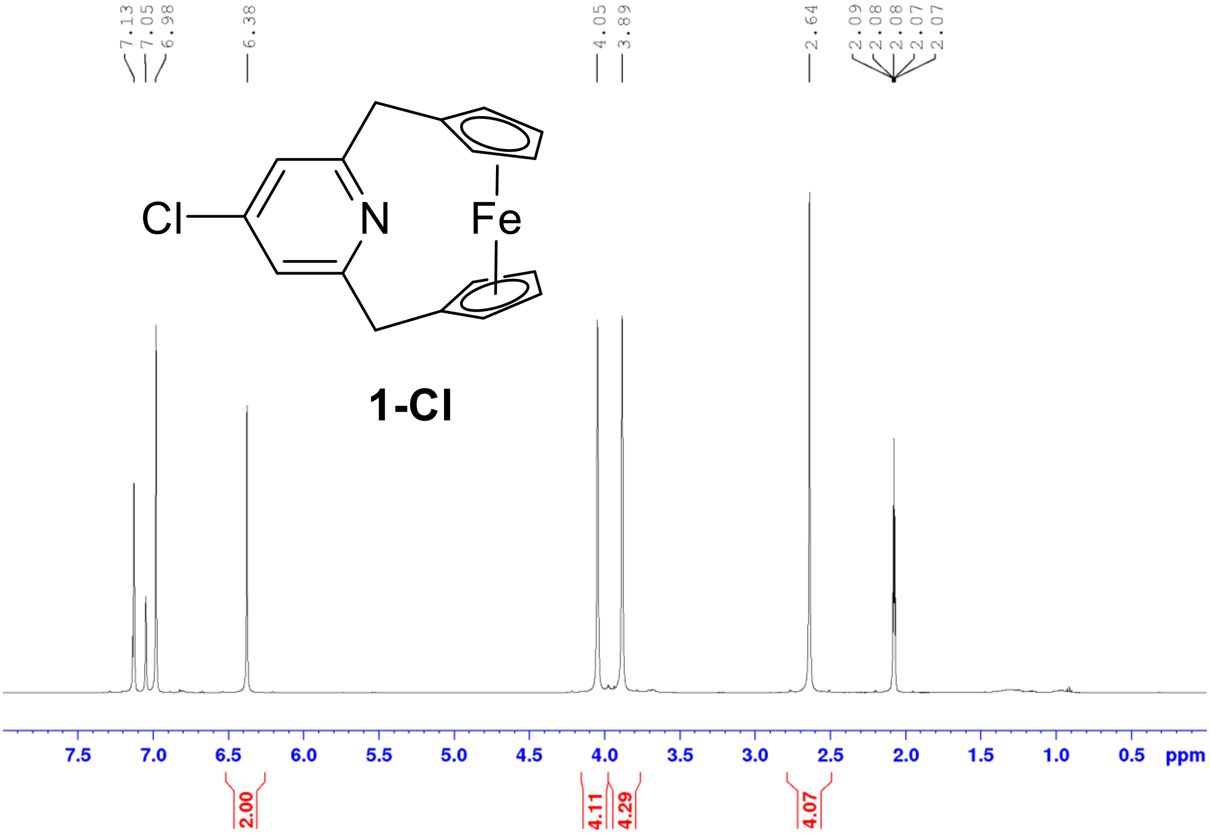

b

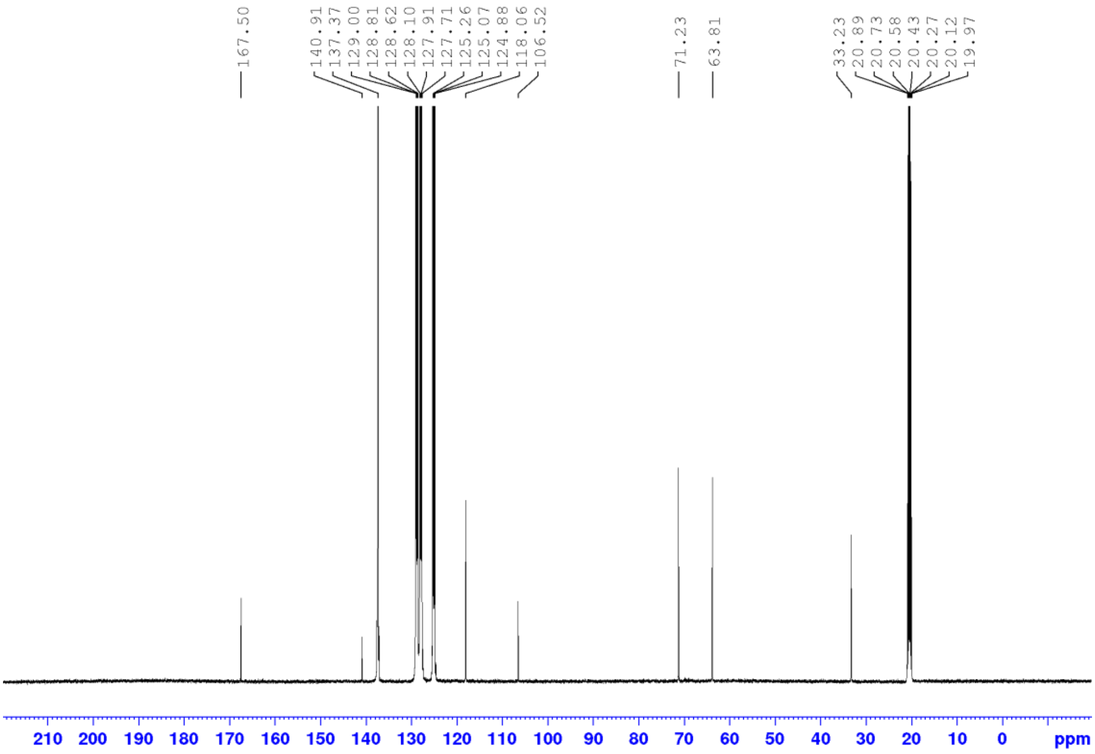

Supplementary Fig. 13.
NMR spectra (Toluene-d_8, 233 K) of **1-Cl**. (**a**) ^{1}H NMR (500.13 MHz). (**b**) ^{13}C{^{1}H} NMR (125.76 MHz).

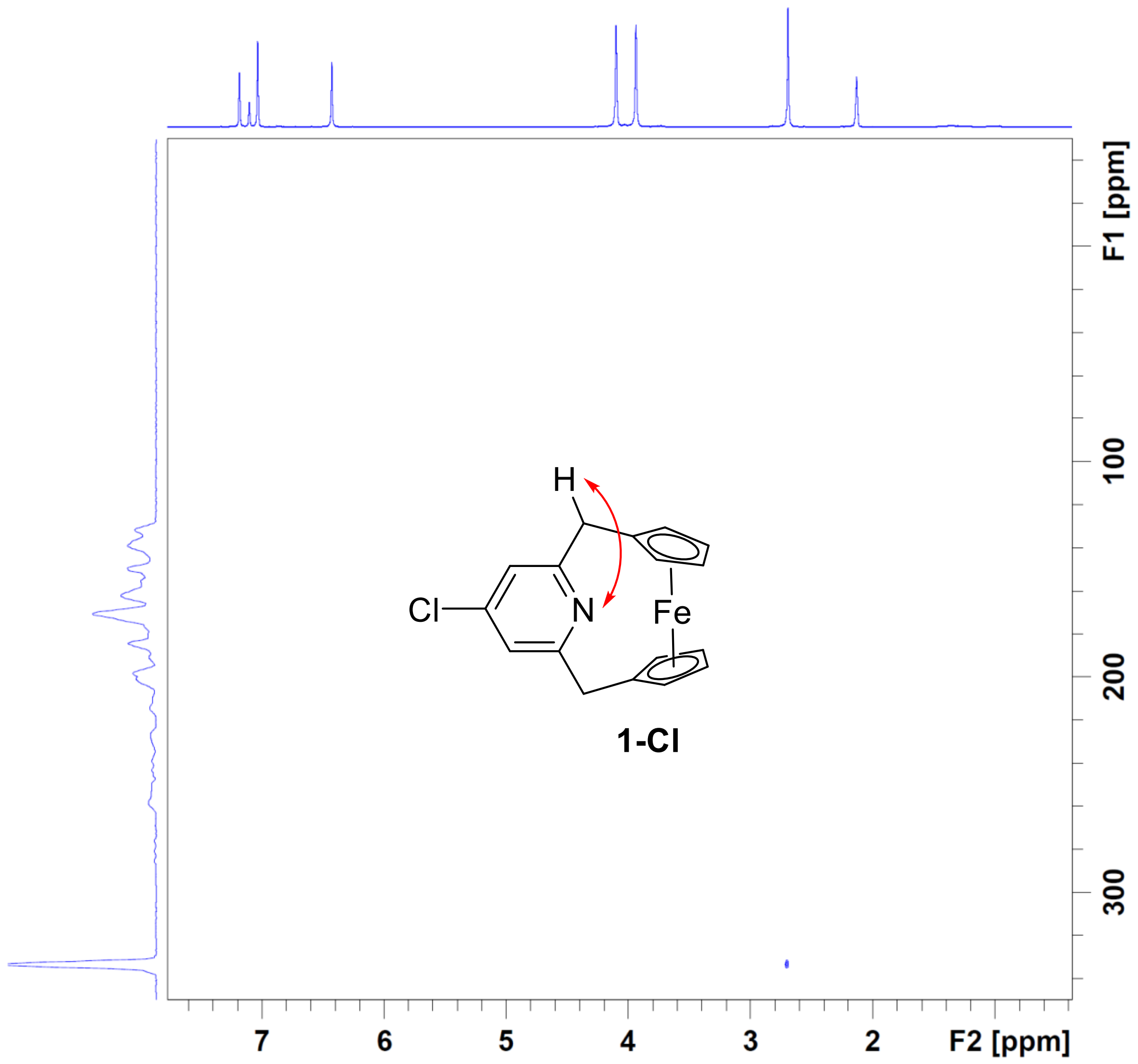

Supplementary Fig. 14.
^{1}H-^{15}N HMBC NMR spectrum (Toluene-d_8, 500.13 MHz, 233 K) of **1-Cl**.

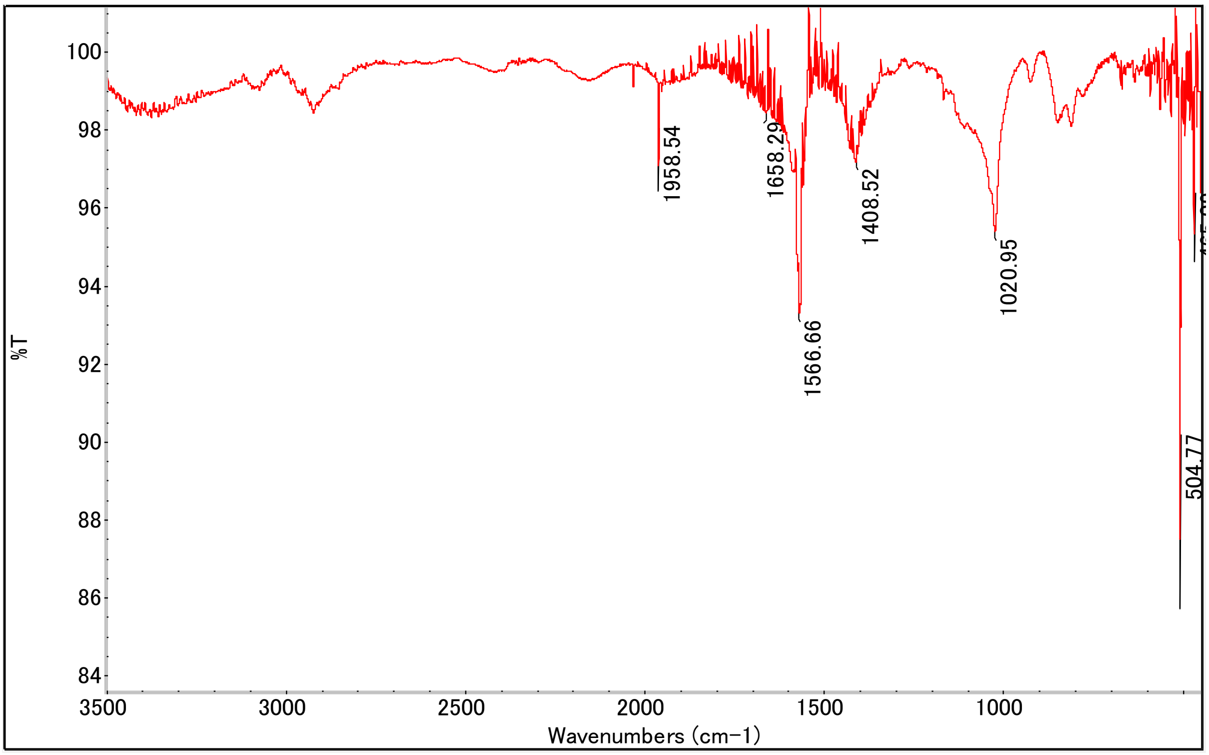

Supplementary Fig. 15.
FTIR spectrum (thin film) of **1-Cl**.

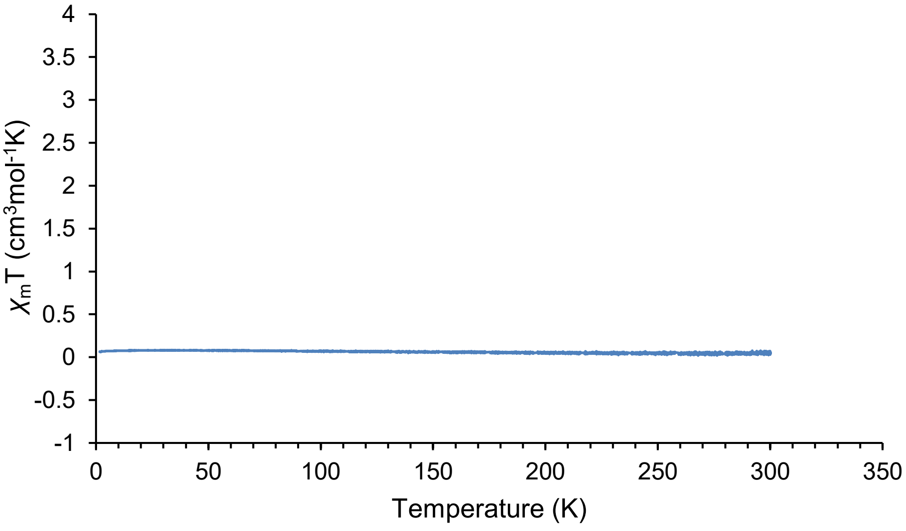

Supplementary Fig. 16.
χ_mT vs. T plot based on VSM measurement of a bulk solid of **1-Cl** at 2-300 K under 1000 Oe.

a

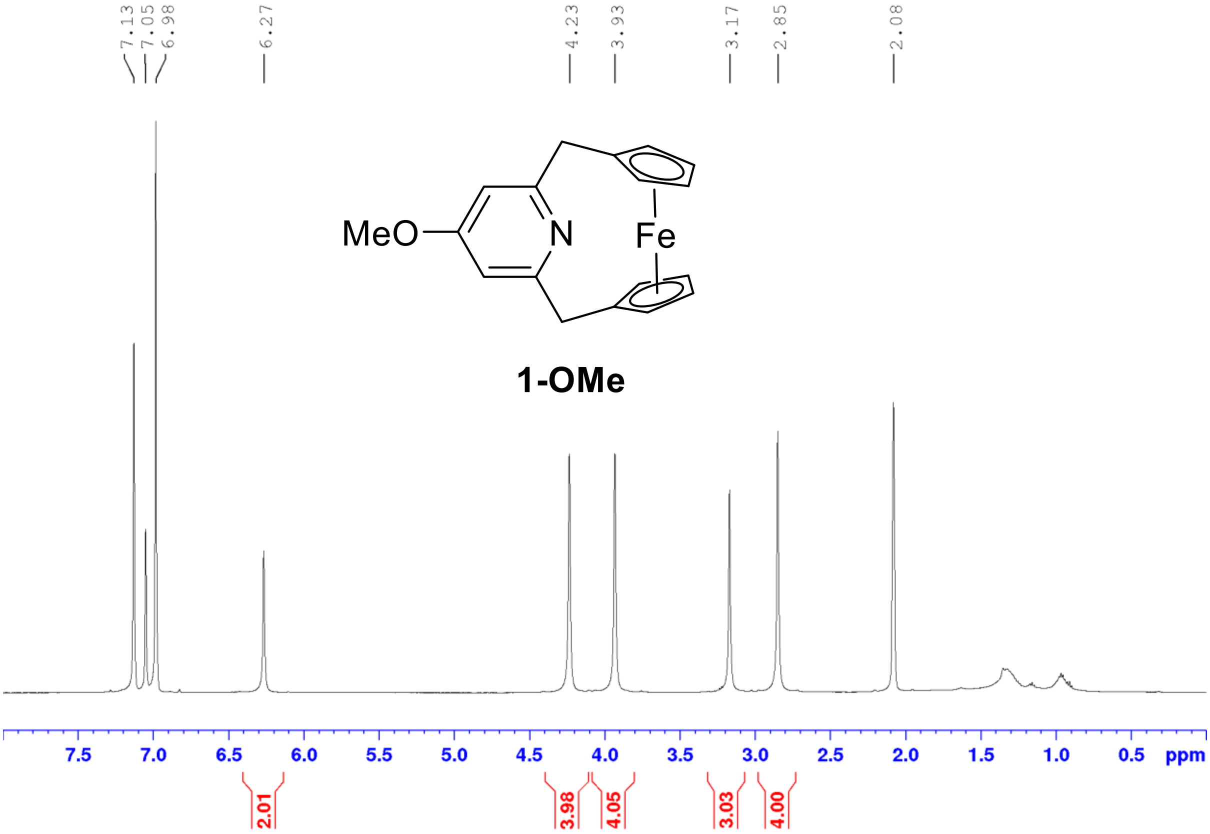

b

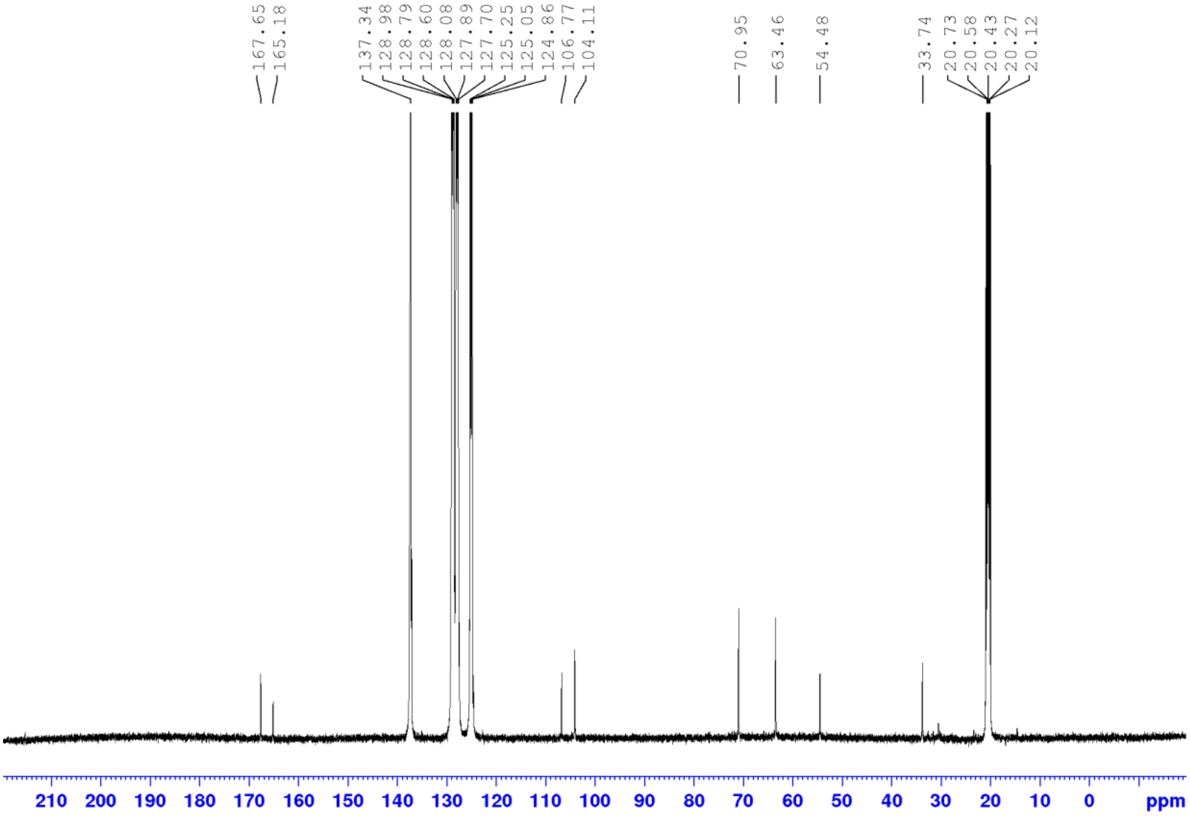

Supplementary Fig. 17.

NMR spectra (Toluene-d_8, 233 K) of **1-OMe**. (**a**) ^{1}H NMR (500.13 MHz). (**b**) $^{13}C\{^1H\}$ NMR(125.76 MHz).

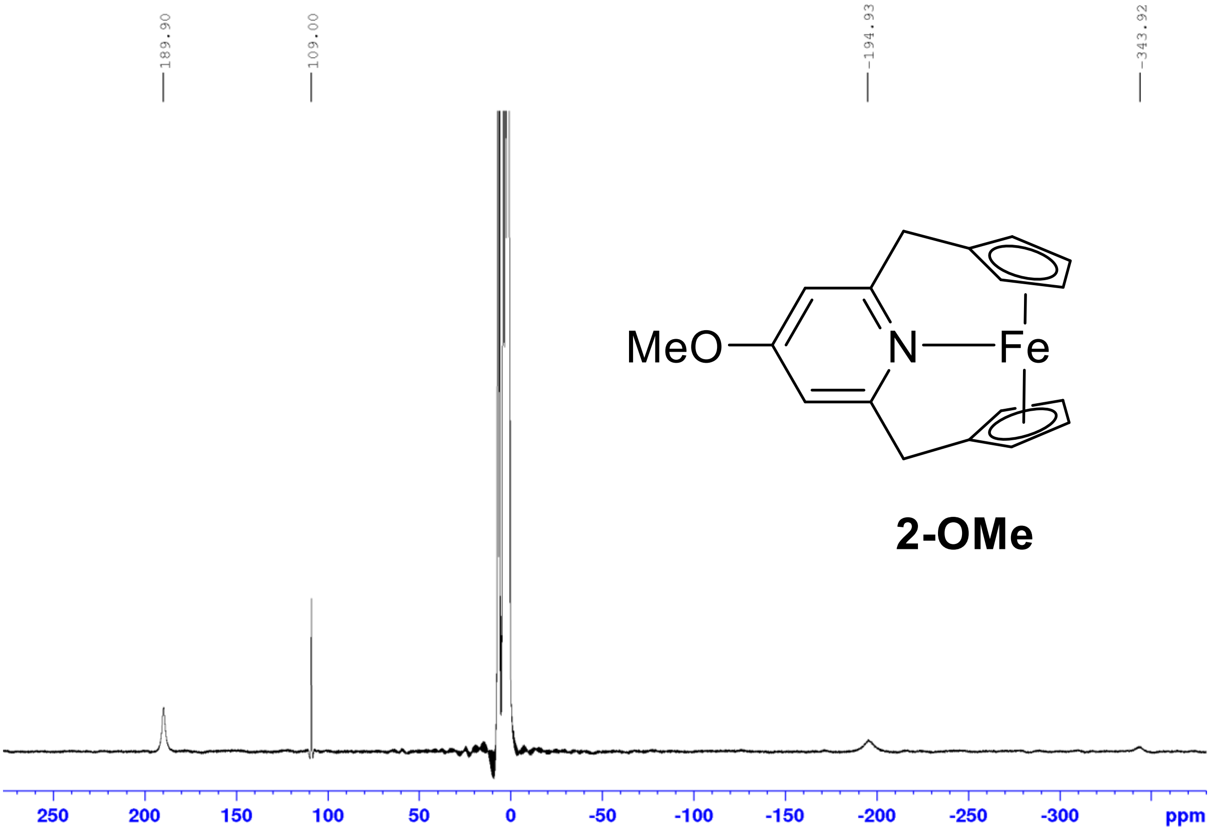

Supplementary Fig. 18.
^{1}H NMR spectrum (toluene-d_8, 500.13 MHz, 233 K) of **2-OMe**.

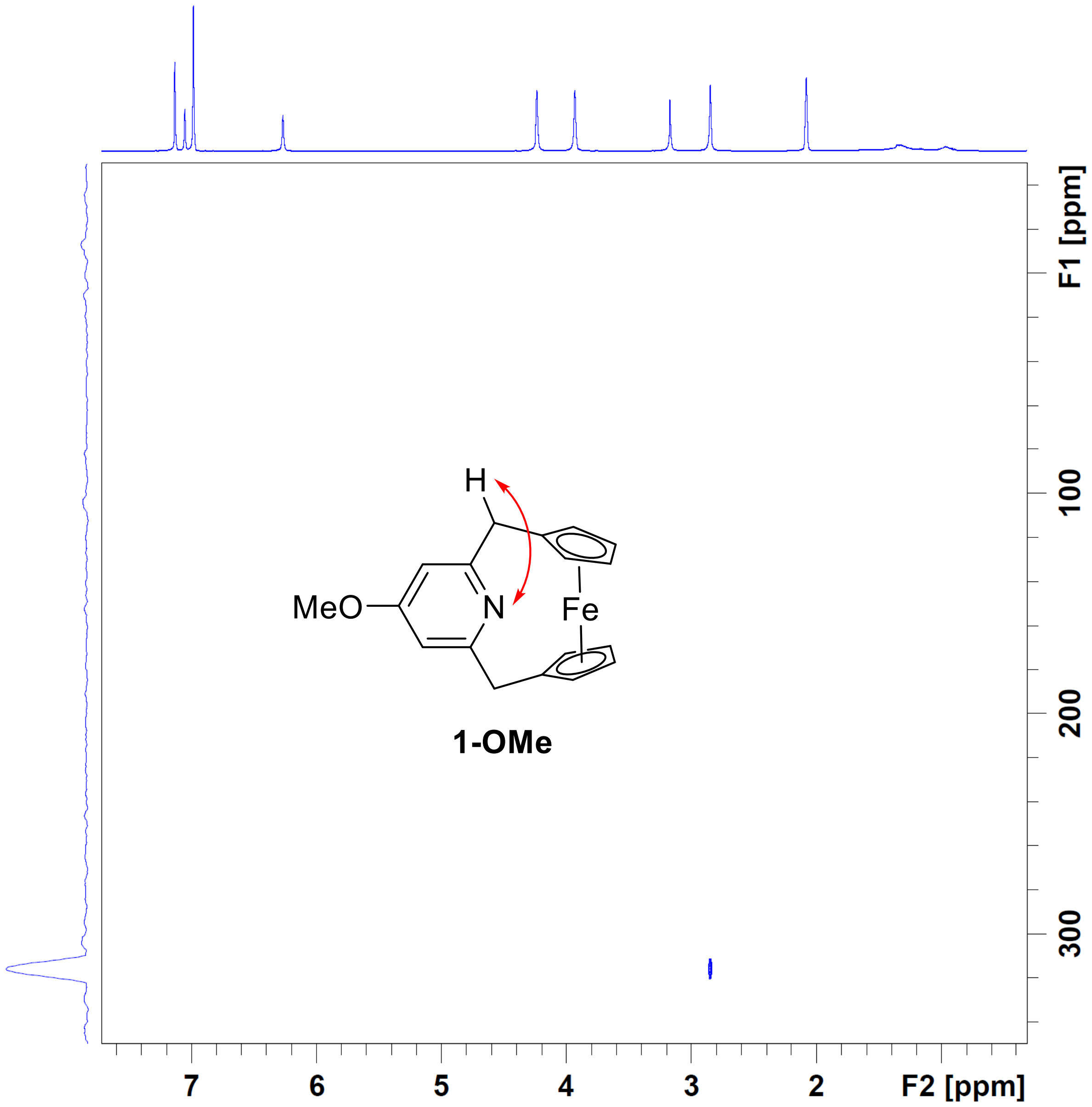

Supplementary Fig. 19.
^{1}H-^{15}N HMBC NMR spectrum (Toluene-d_8, 500.13 MHz, 233 K) of **1-OMe**.

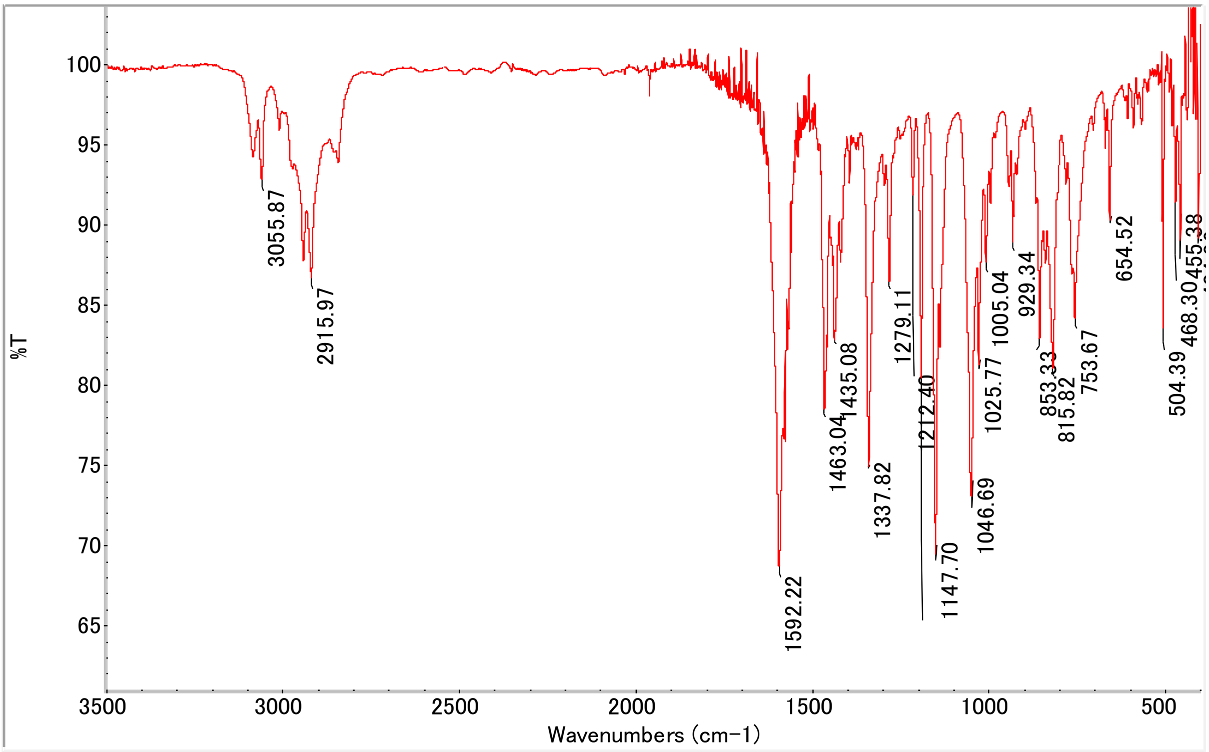

Supplementary Fig. 20.
FTIR spectrum (thin film) of **1-OMe**.

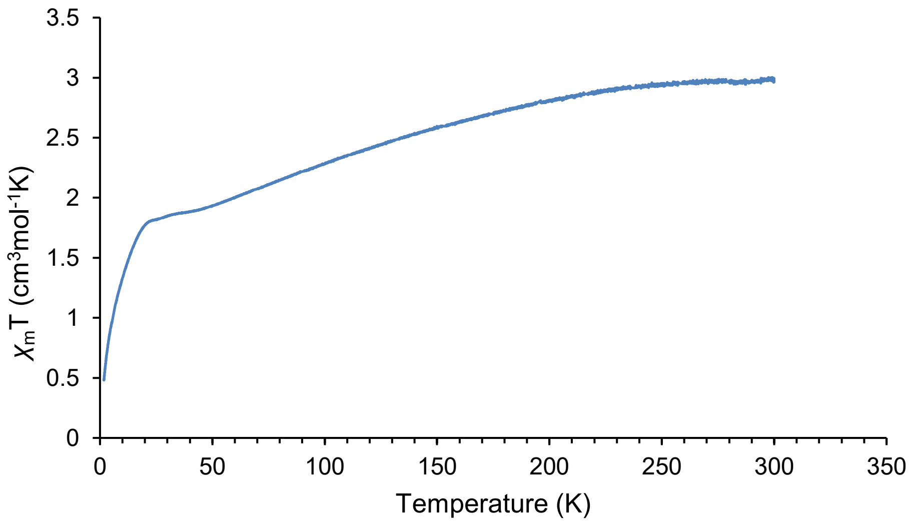

Supplementary Fig. 21.
χ_mT vs. T plot based on VSM measurement of a bulk solid of **1-OMe** at 2-300 K under 1000 Oe.

a

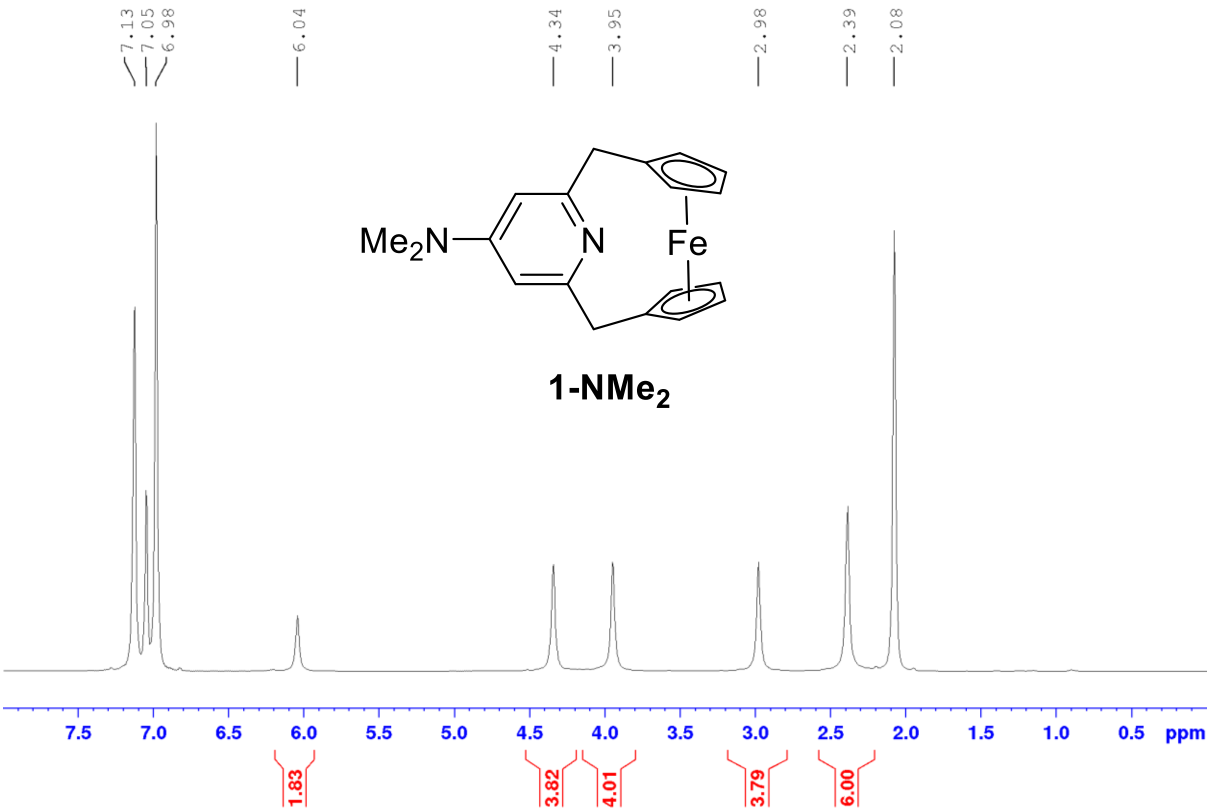

b

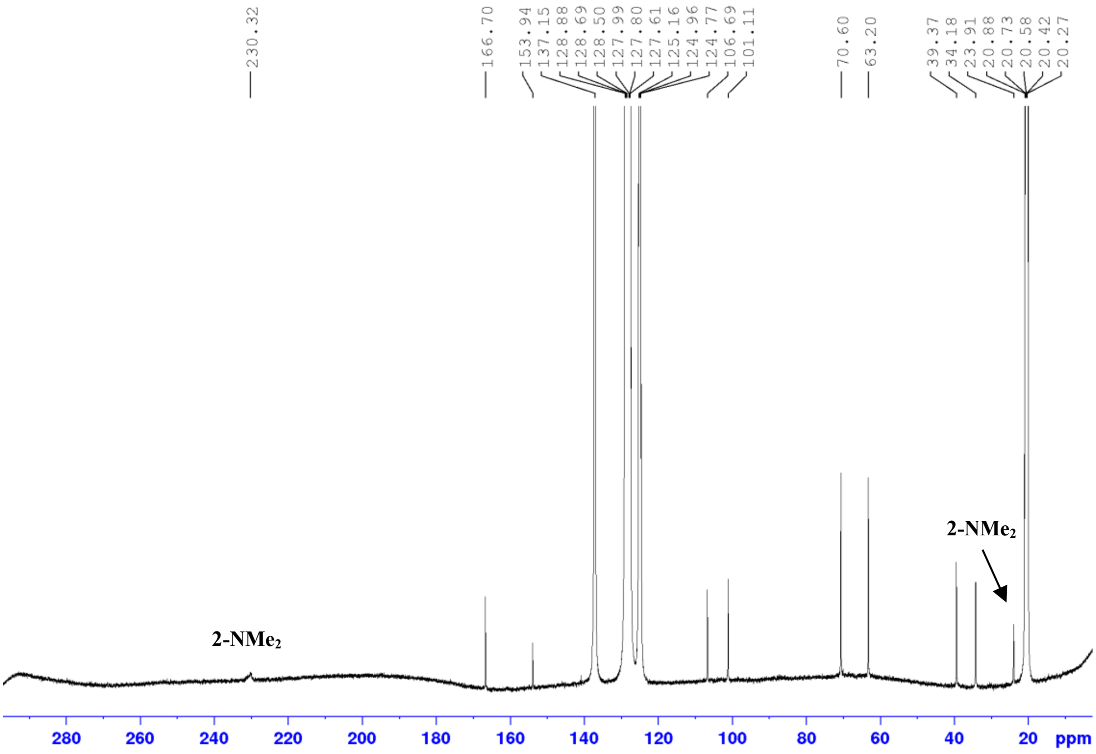

Supplementary Fig. 22.

NMR spectra (Toluene-d_8, 233 K) of **1-NMe₂**. (**a**) ^{1}H NMR (500.13 MHz). (**b**) ^{13}C{^{1}H} NMR (125.76 MHz).

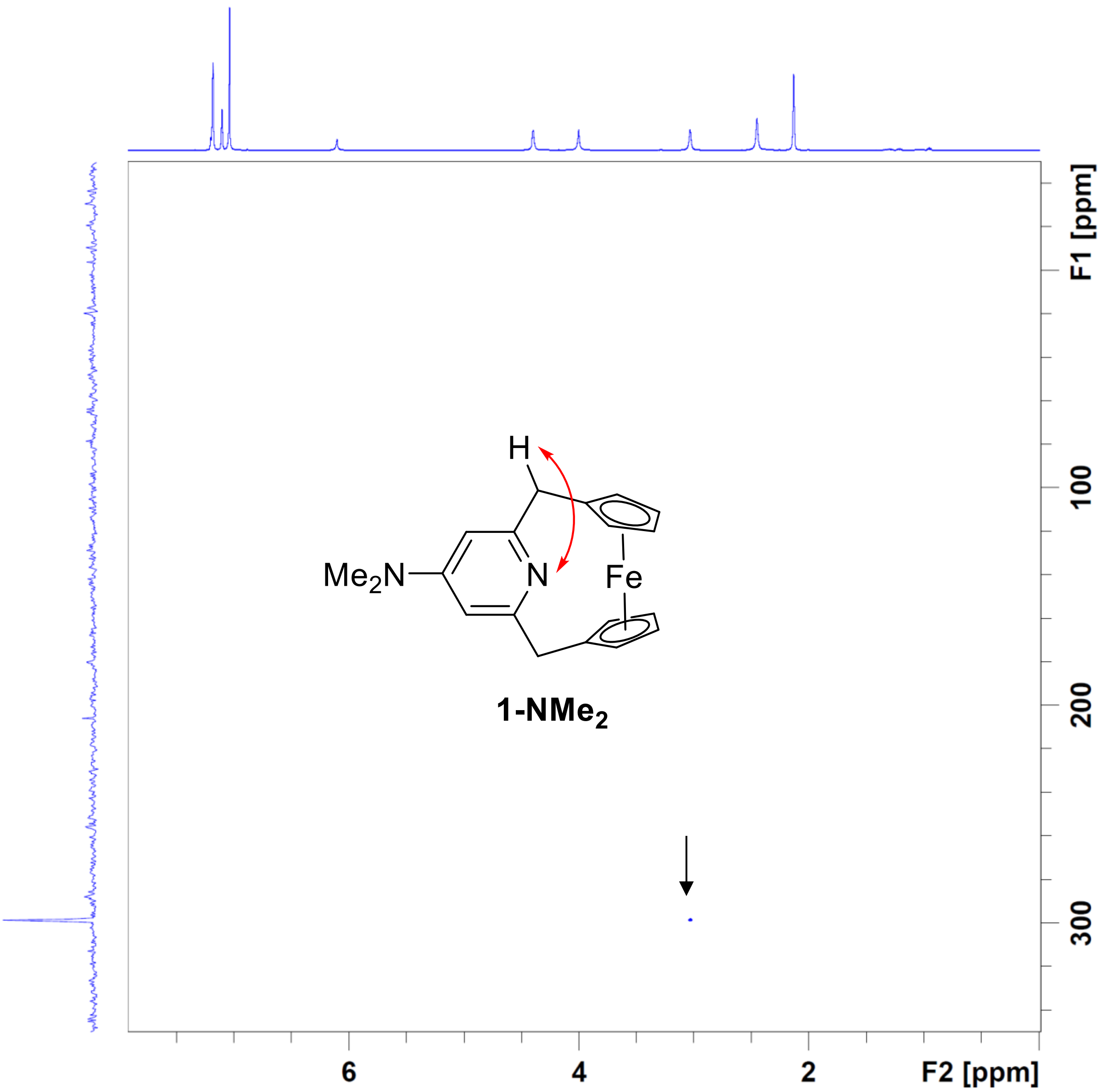

Supplementary Fig. 23.
^{1}H-^{15}N HMBC NMR spectrum (Toluene-d_8, 500.13 MHz, 233 K) of **1-NMe$_2$**.

a

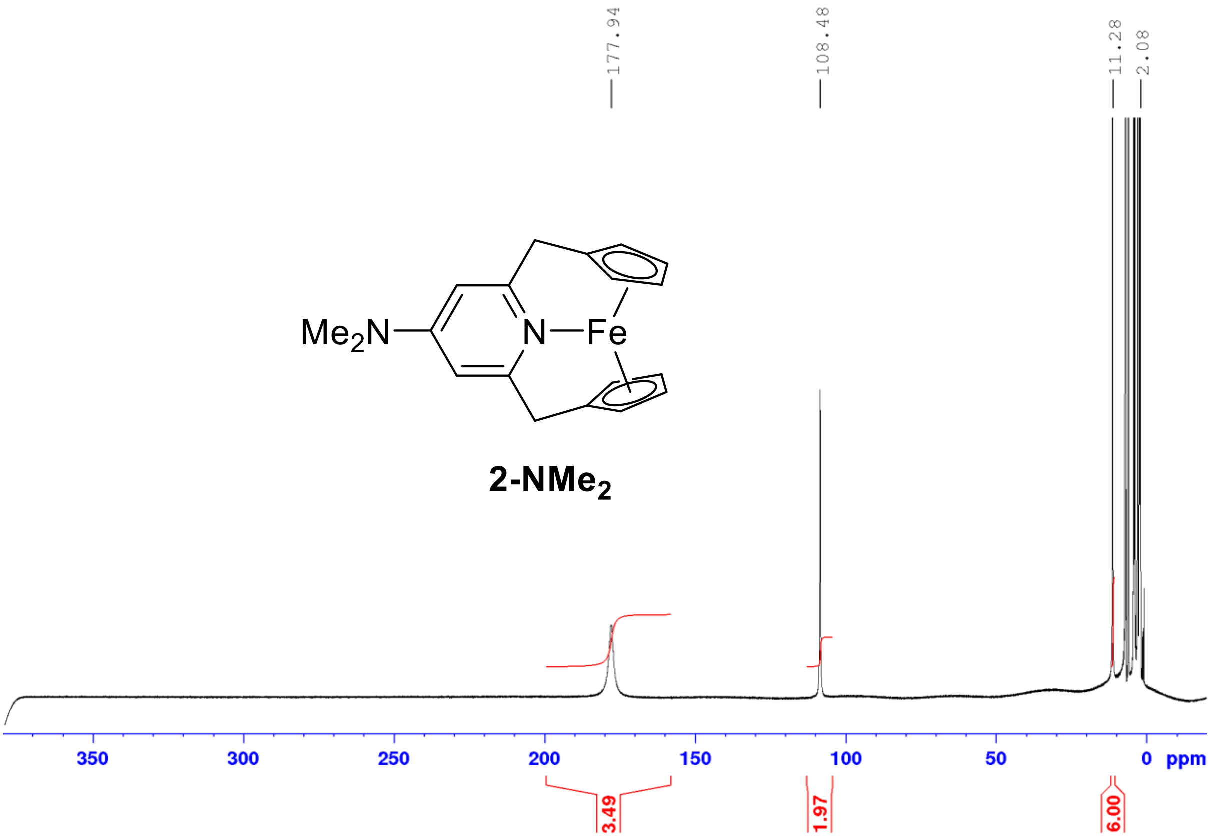

b

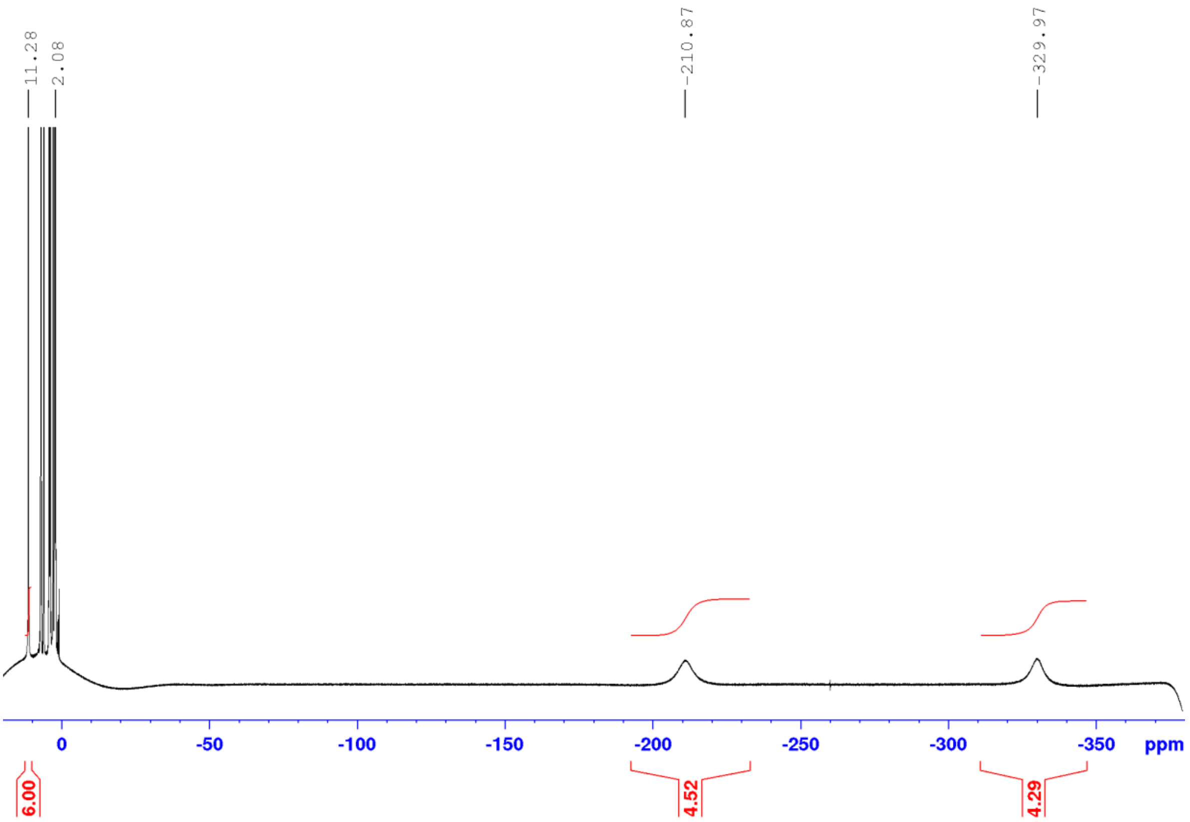

Supplementary Fig. 24.
^{1}H NMR spectra (Toluene-d_8, 500.13 MHz, 233 K) of **2-NMe₂**. Using the same sample, two separate spectra were recorded at different spectral ranges. NMe_2 signal at 11.28 ppm was used as an internal standard for the integration. (**a**) −20 to 380 ppm. (**b**) −380 to 20 ppm.

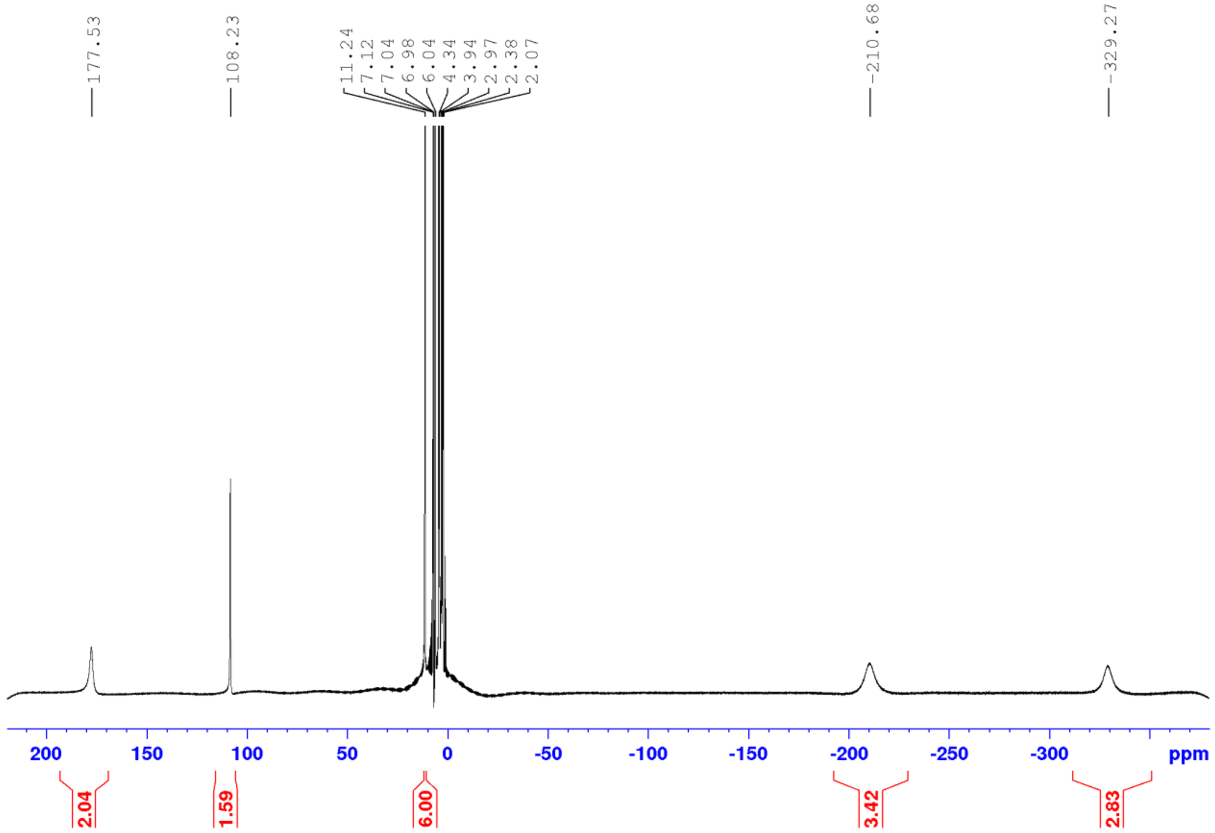

Supplementary Fig. 25.
^{1}H NMR spectrum (220 to −380 ppm, Toluene-d_8, 500.13 MHz, 233 K) of **2-NMe$_2$**.

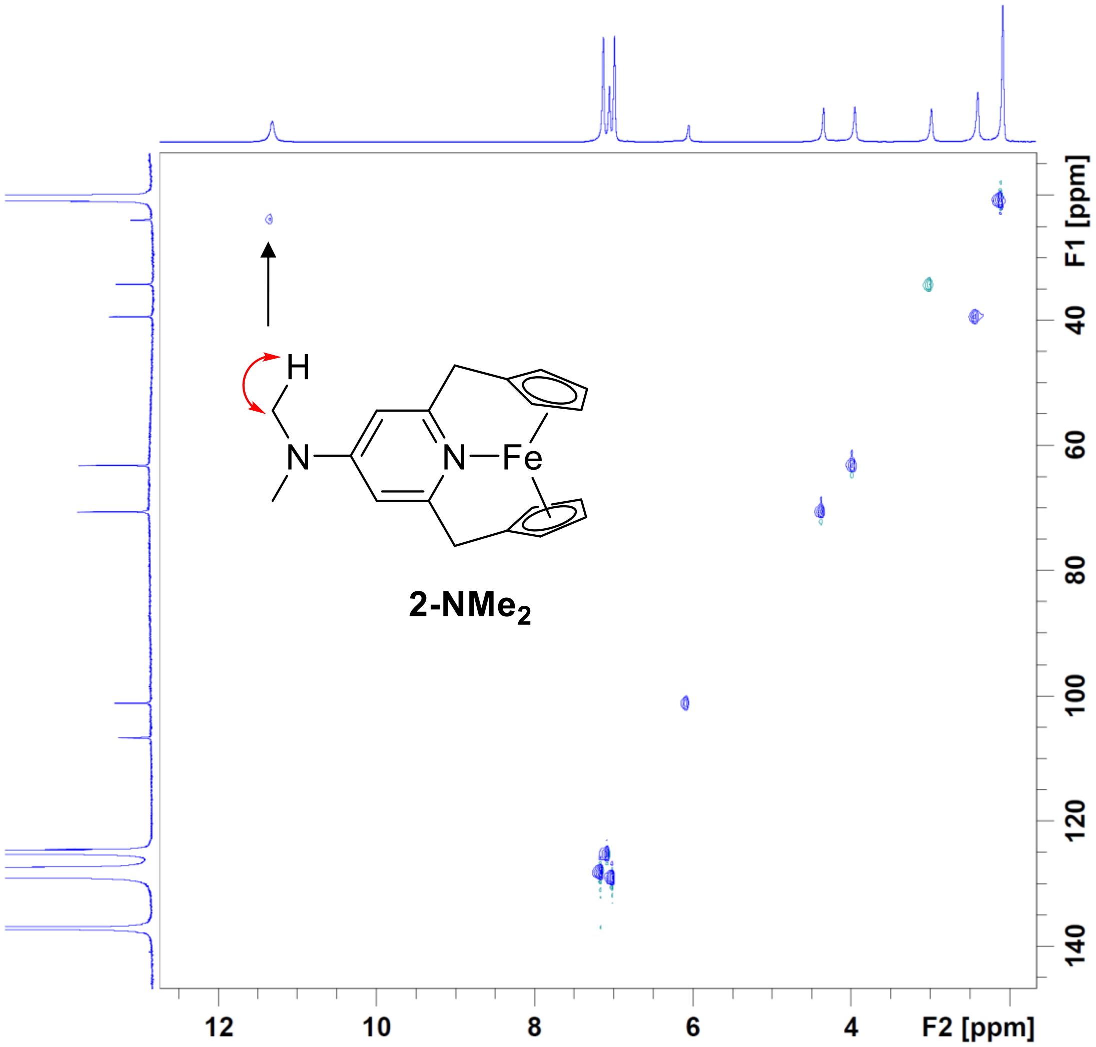

Supplementary Fig. 26.
^1H-^{13}C HSQC NMR spectrum (Toluene-d_8, 500.13 MHz, 233 K) of **2-NMe₂.**

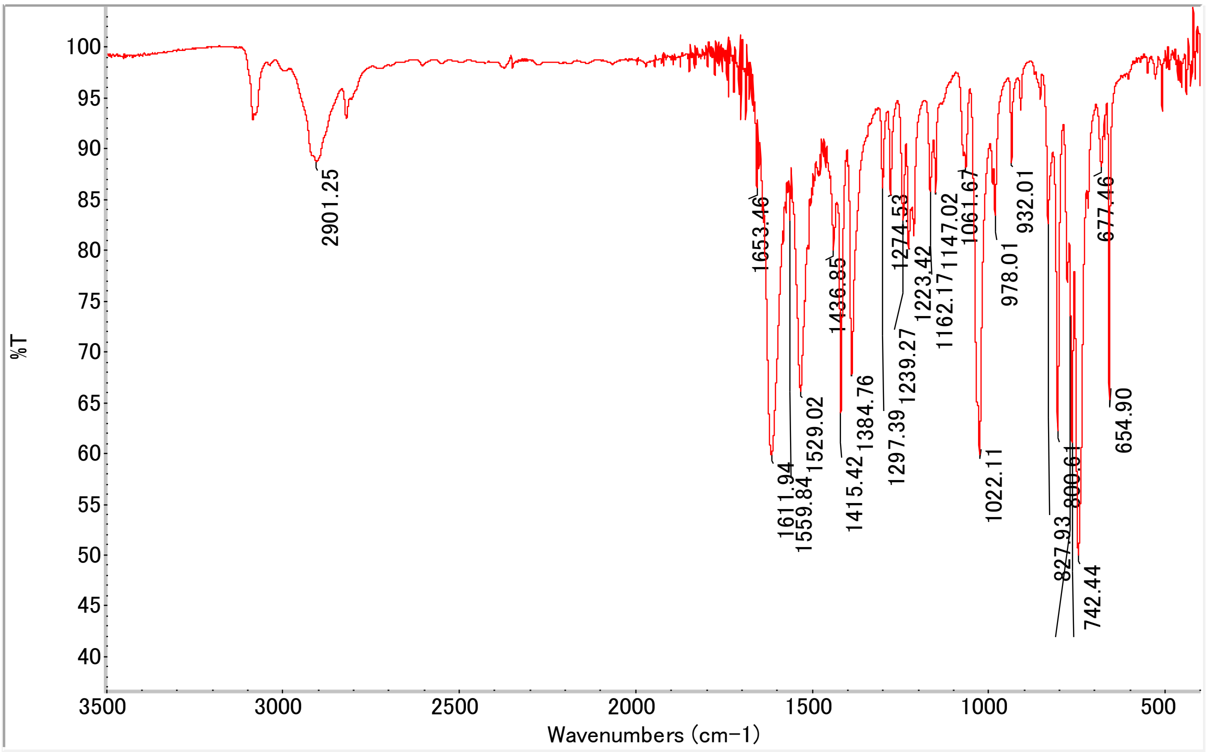

Supplementary Fig. 27

FTIR spectrum (KBr pellet) of **2-NMe₂**.

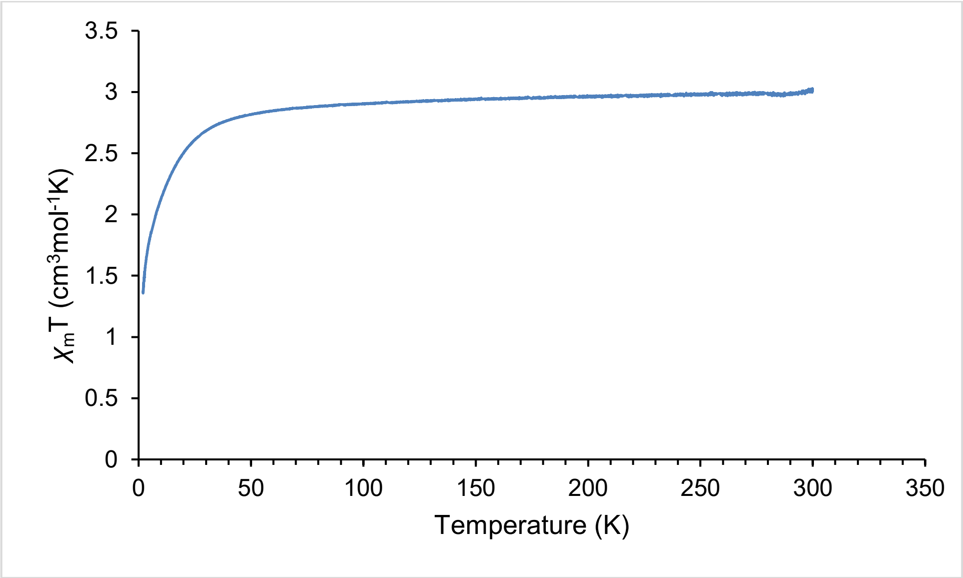

Supplementary Fig. 28.

χT vs. T plot based on VSM measurement of crystals of **2-NMe₂** at 2-300 K under 1000 Oe.

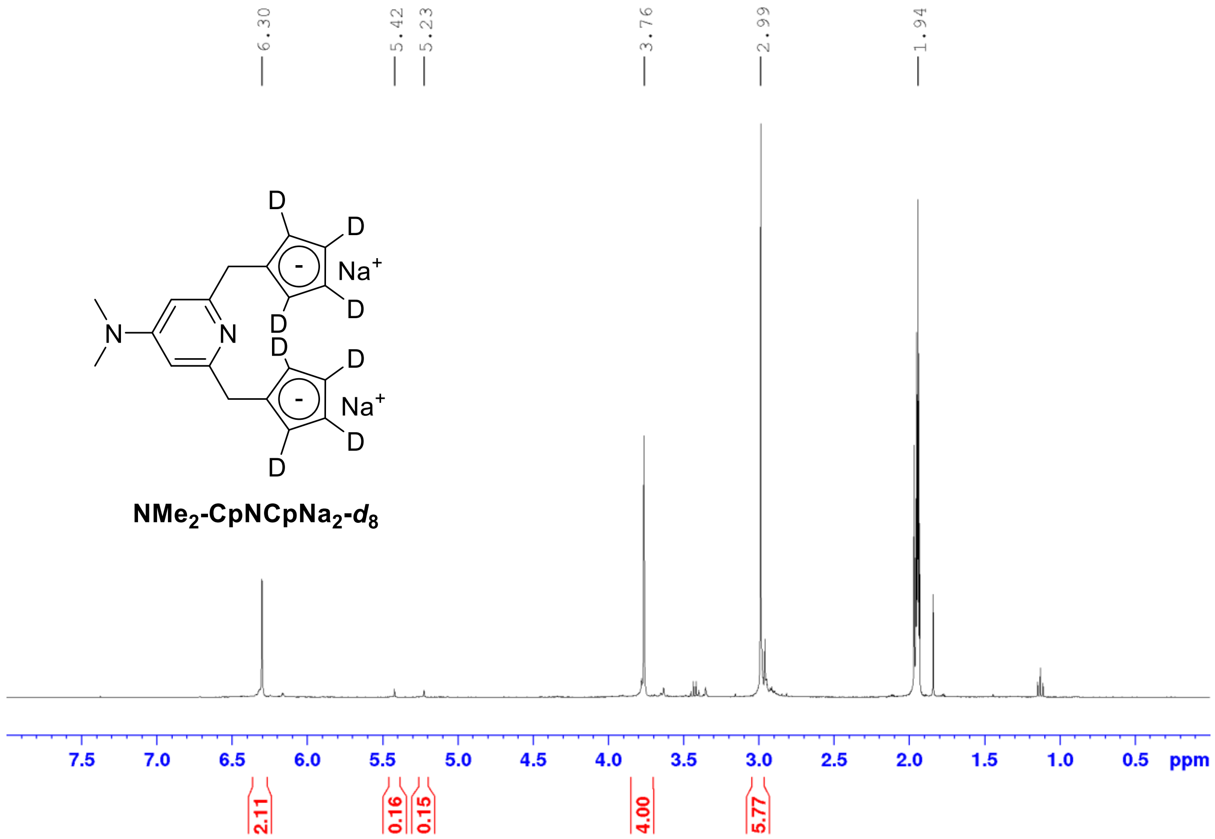

Supplementary Fig. 29.
^{1}H NMR spectrum (CD_3CN, 400.15 MHz, 298 K) of **NMe2-CpNCpNa2-*d*8**.

a

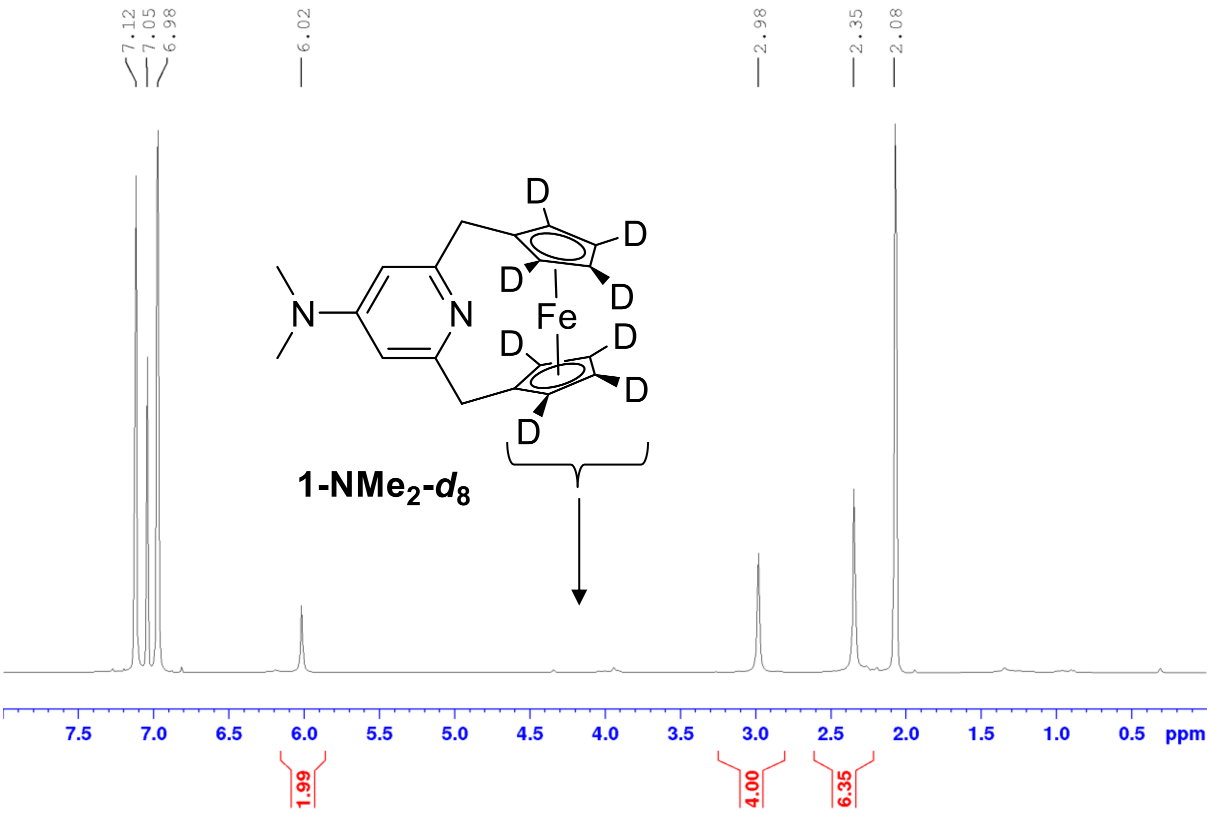

b

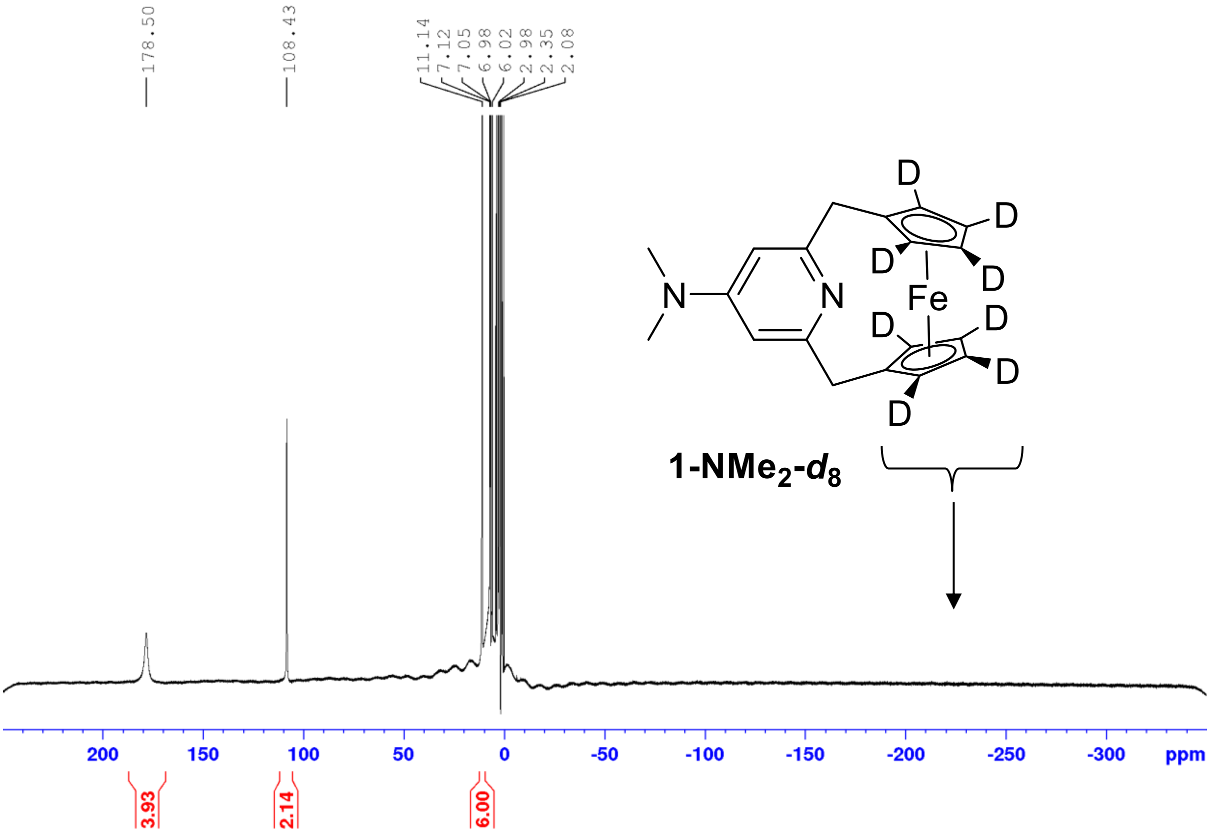

Supplementary Fig. 30.

^{1}H NMR spectra (Toluene-d_8, 500.13 MHz, 233 K) of (**a**) **1-NMe2-*d*8** and (**b**) **2-NMe2-*d*8**.

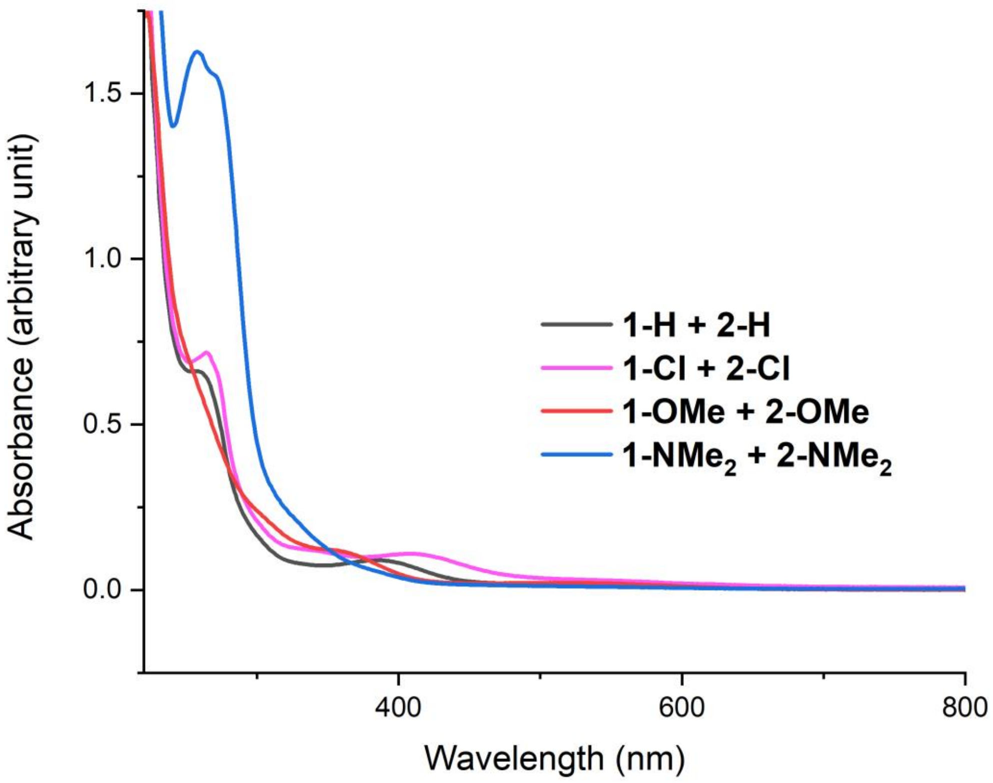

Supplementary Fig. 31.
UV-Vis spectra (0.1 mM in THF, 295 K) of an equilibrium mixture of **1-X** and **2-X**.

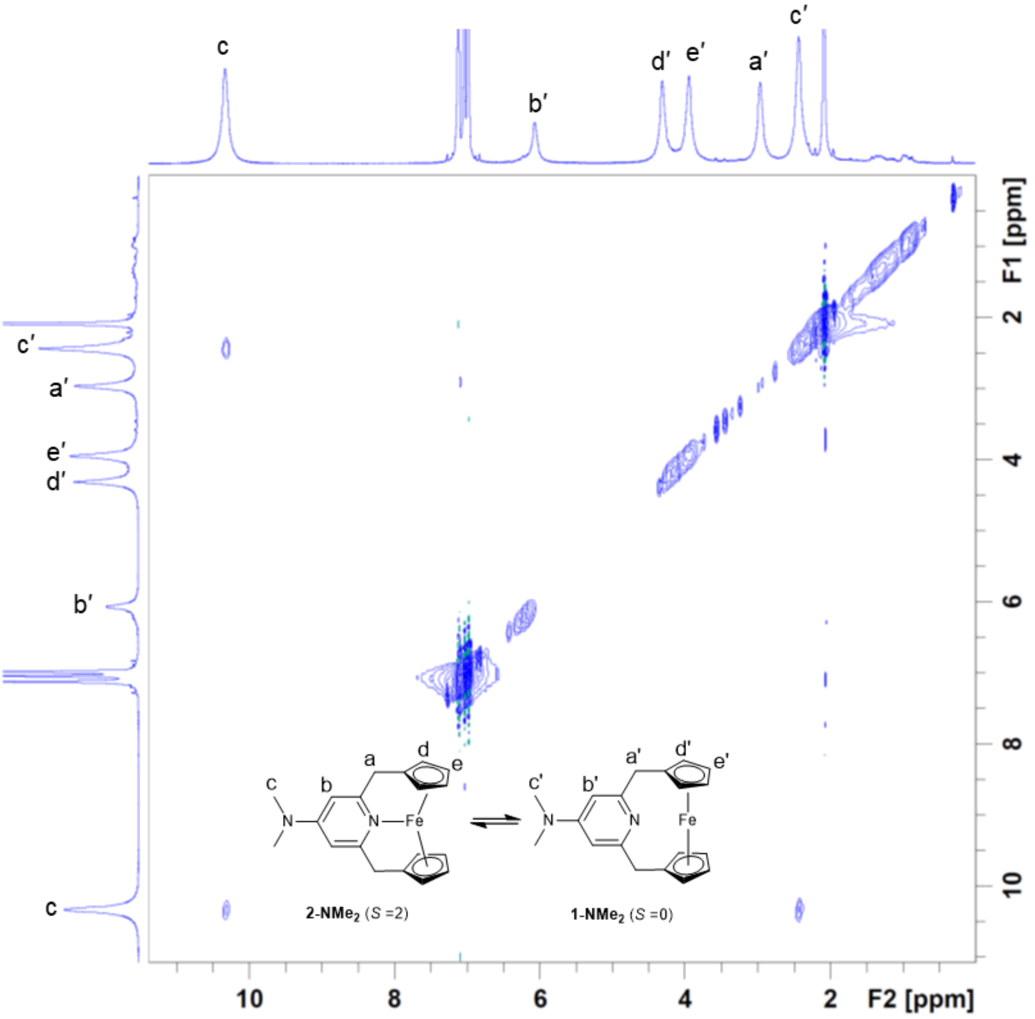

Supplementary Fig. 32.

^{1}H-^{1}H EXSY NMR spectrum (500.13 MHz) of **1-NMe2** and **2-NMe2**. The spectrum was recorded in toluene-d_8, at −20 °C, with mixing time of 0.3 s. Corresponding signal assignments (c and a′-e′) are shown in the figure.

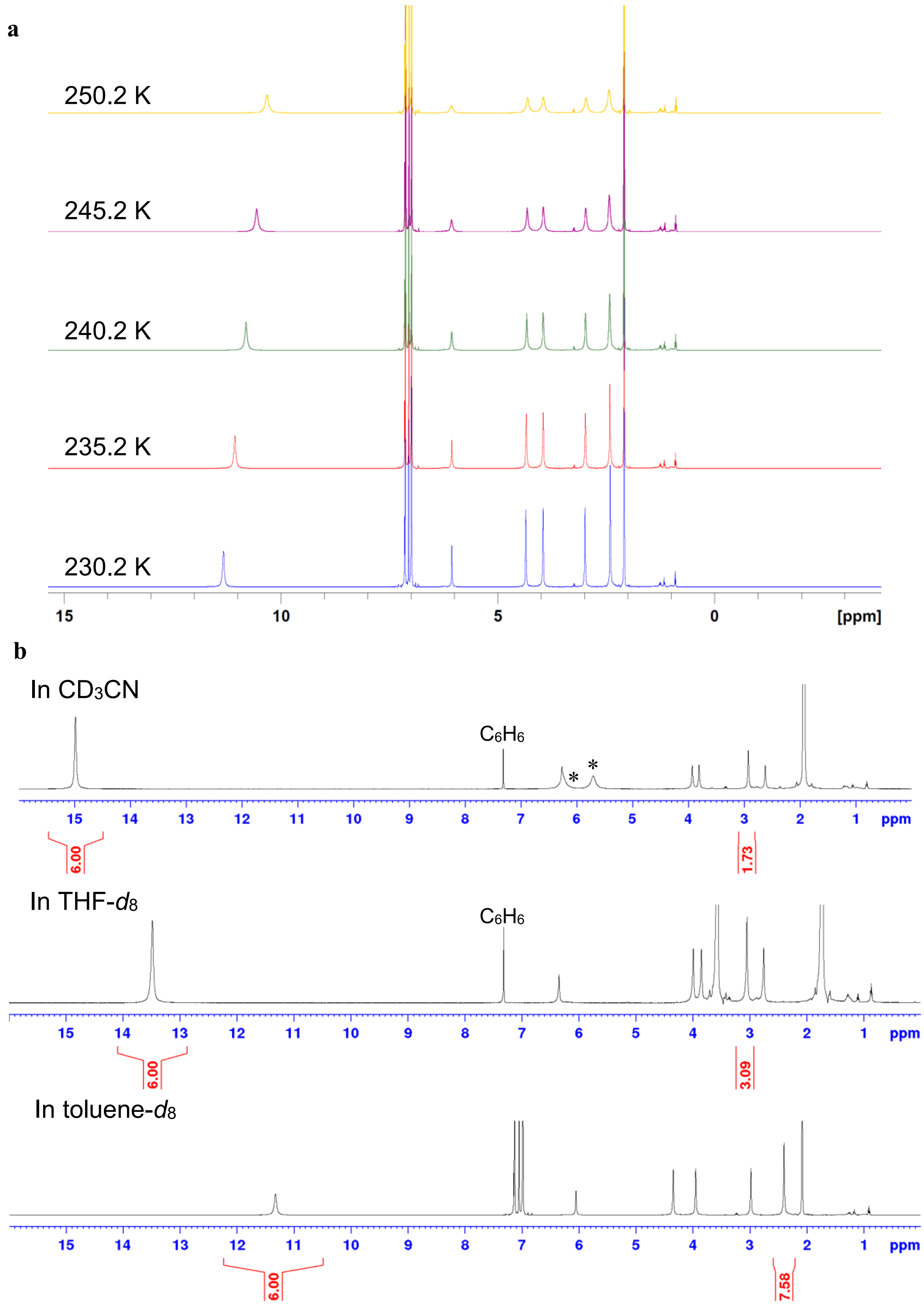

Supplementary Fig. 33.
(**a**) ^{1}H NMR spectra (Toluene-d_8, 500.13 MHz, 230.2-250.2 K) of **1-NMe₂** and **2-NMe₂**. (**b**) ^{1}H NMR spectra (230.2 K) of **1-NMe₂** and **2-NMe₂** in CD_3CN, THF-d_8, and toluene-d_8.*: Impurities from CD_3CN.

a

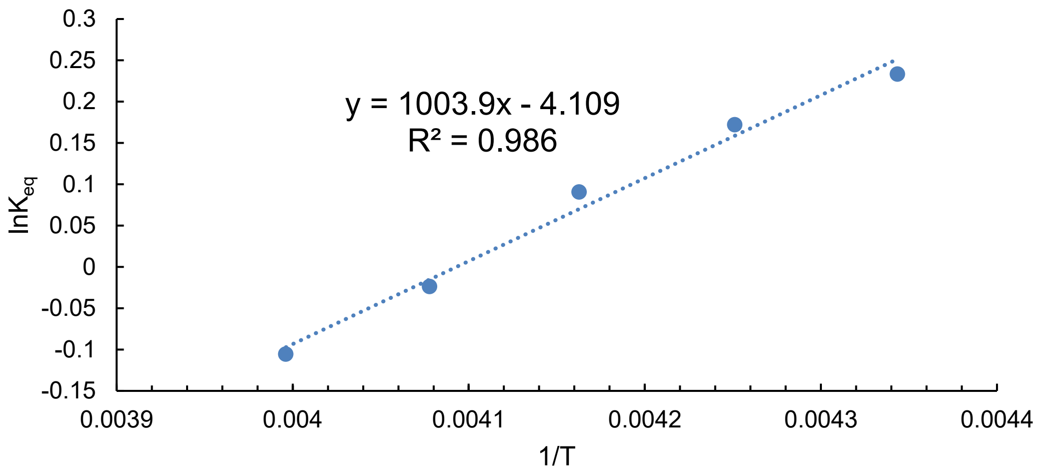

b

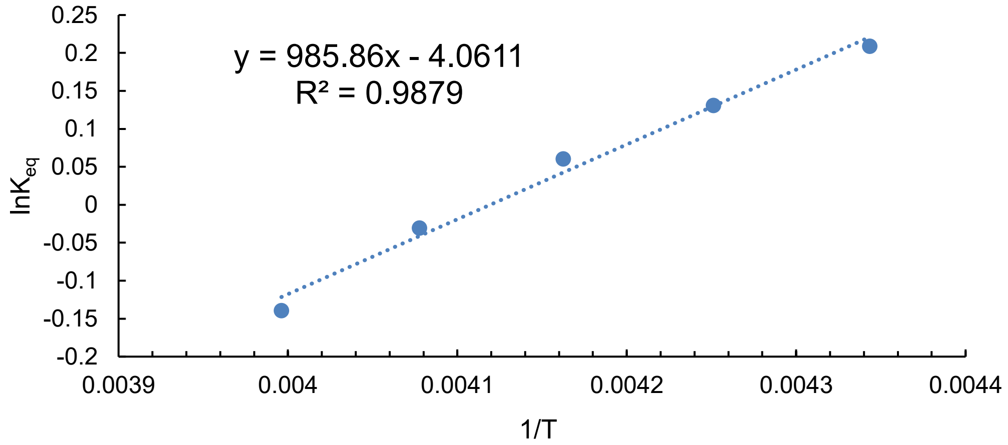

Supplementary Fig. 34.

Van't Hoff plots used to estimate the thermodynamic parameters for the equilibrium between **1-NMe2** and **2-NMe2**. (**a**) Using ^{1}H NMR integration values of NMe_2 signal of **2-NMe2** and CH_2 signal of **1-NMe2**. (**b**) Using ^{1}H NMR integration values of NMe_2 signals of **2-NMe2** and **1-NMe2**.

a

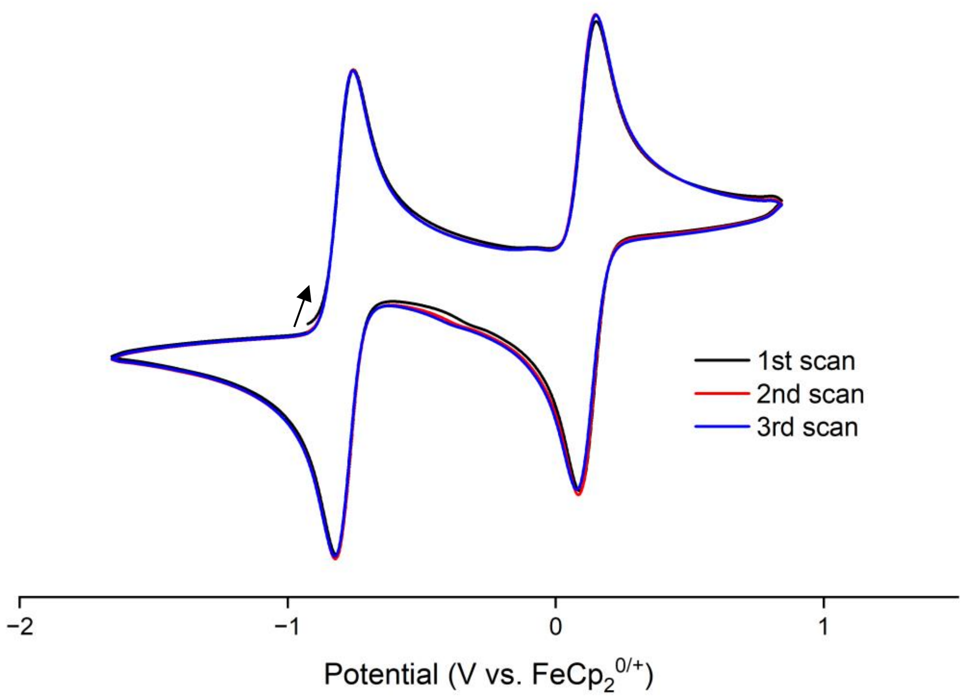

b

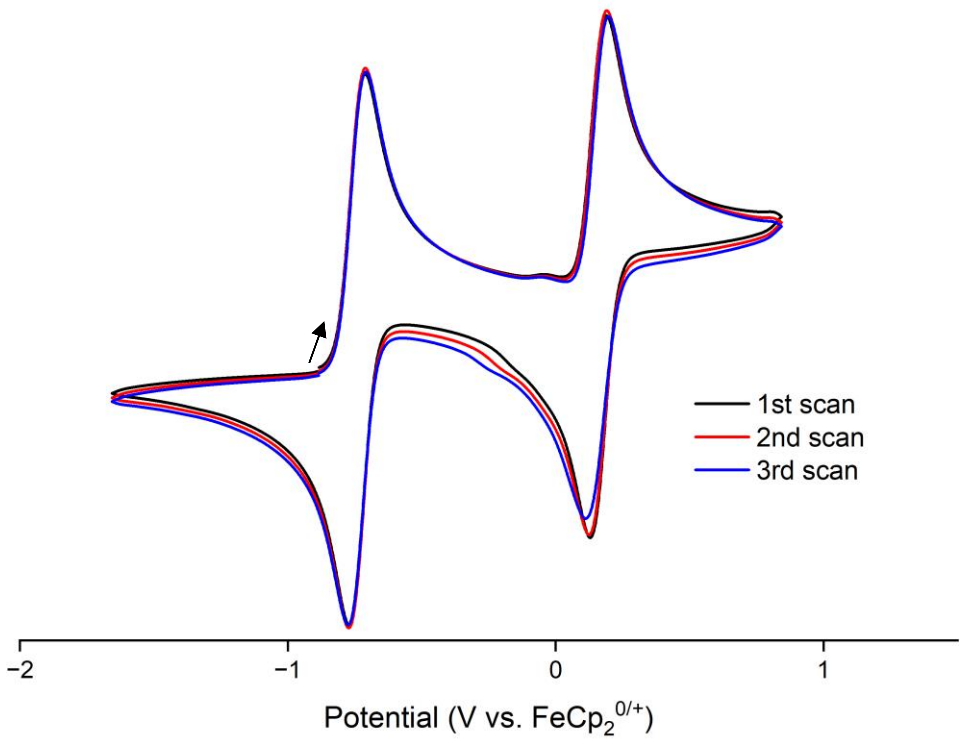

Supplementary Fig. 35.

Voltammograms of a 2.5 mM equilibrium mixture of (**a**) **1-H** and **2-H** and (**b**) **1-Cl** and **2-Cl**. In 0.2 M NBu_4PF_6 in THF at 23 °C, recorded at a scan rate of 0.1 V s^{-1}.

a

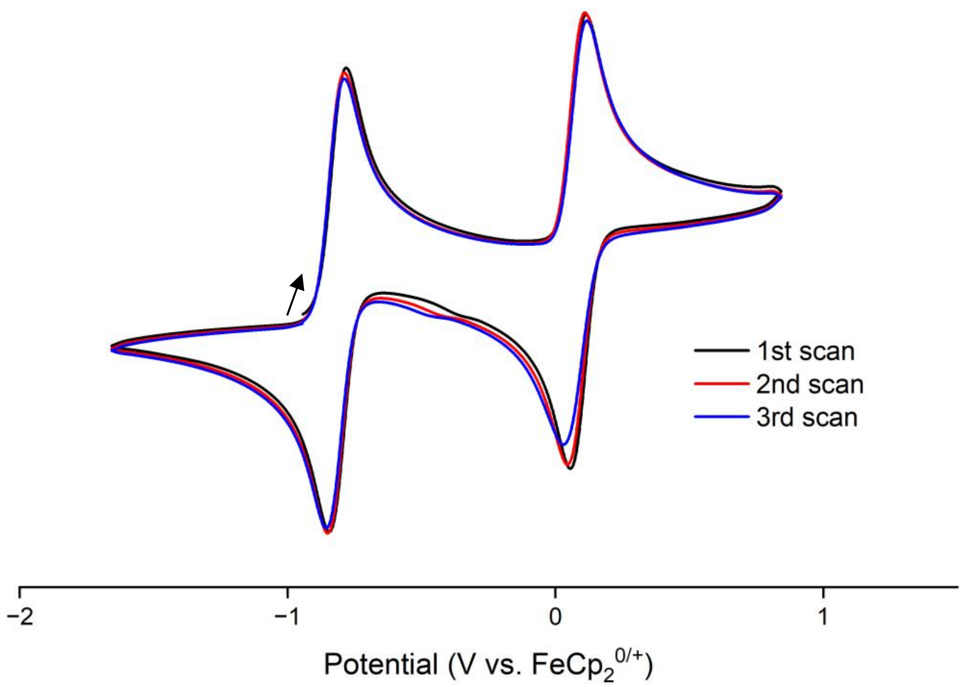

b

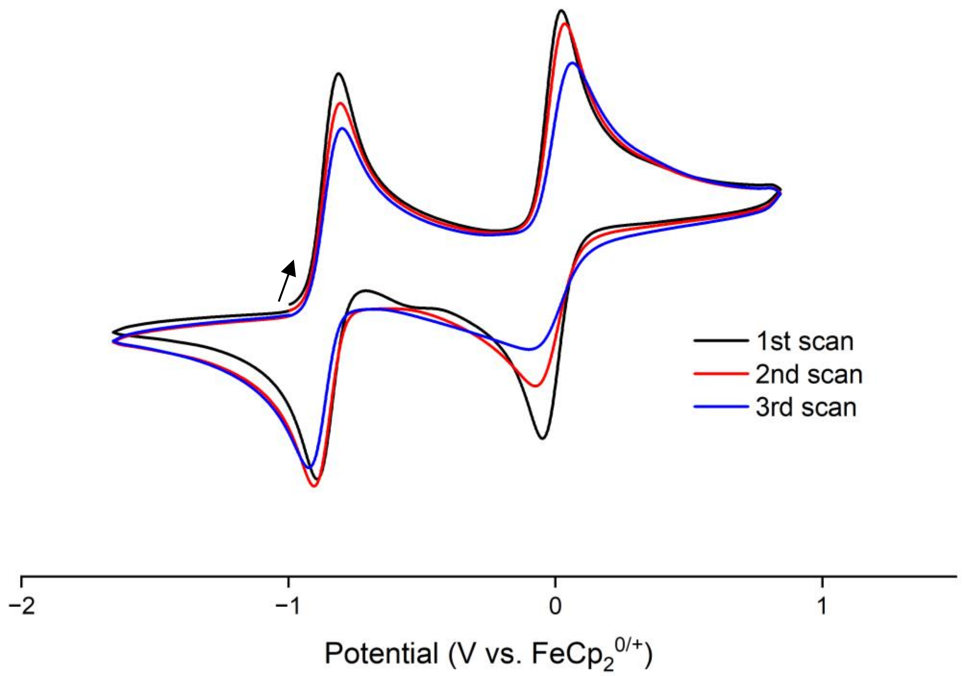

Supplementary Fig. 36.

Voltammograms of a 2.5 mM equilibrium mixture of (**a**) **1-OMe** and **2-OMe** and (**b**) **1-NMe$_2$** and **2-NMe$_2$**. In 0.2 M NBu_4PF_6 in THF at 23 °C, recorded at a scan rate of 0.1 V s^{-1}.

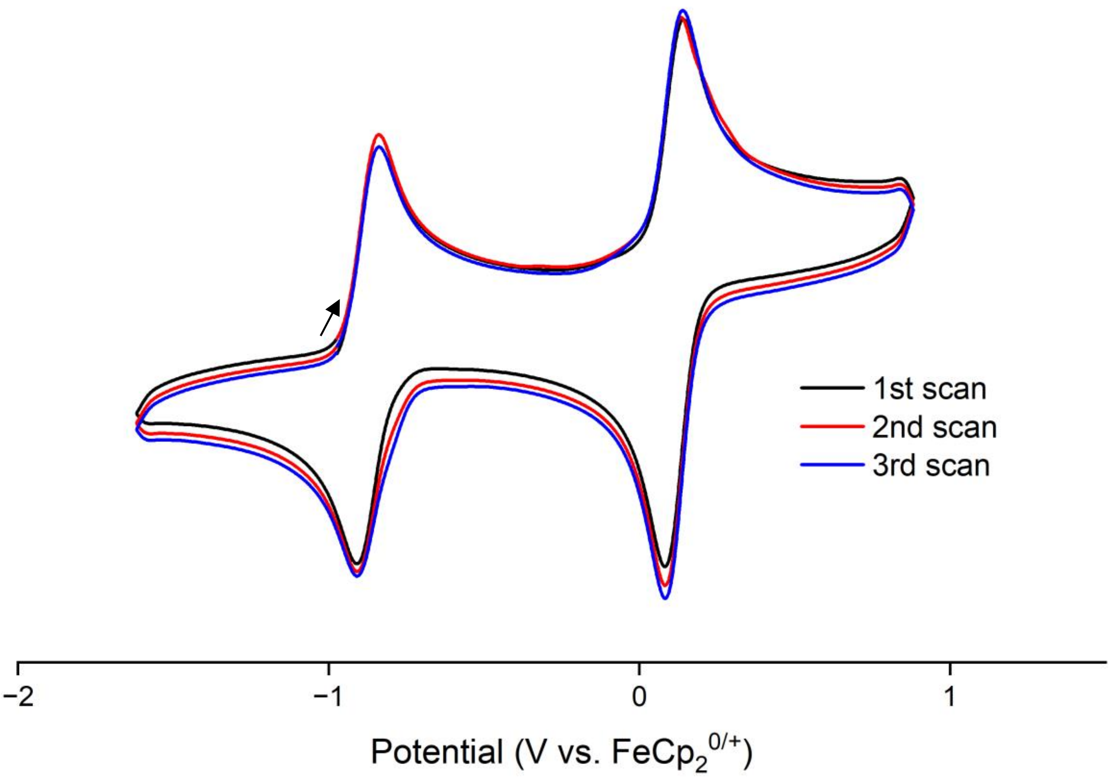

Supplementary Fig. 37.

Voltammograms of a 0.25 mM equilibrium mixture of **1-NMe$_2$** and **2-NMe$_2$**. In 0.1 M NBu_4PF_6 in CH_2Cl_2 at 23 °C, recorded at a scan rate of 0.1 V s^{-1}.

a

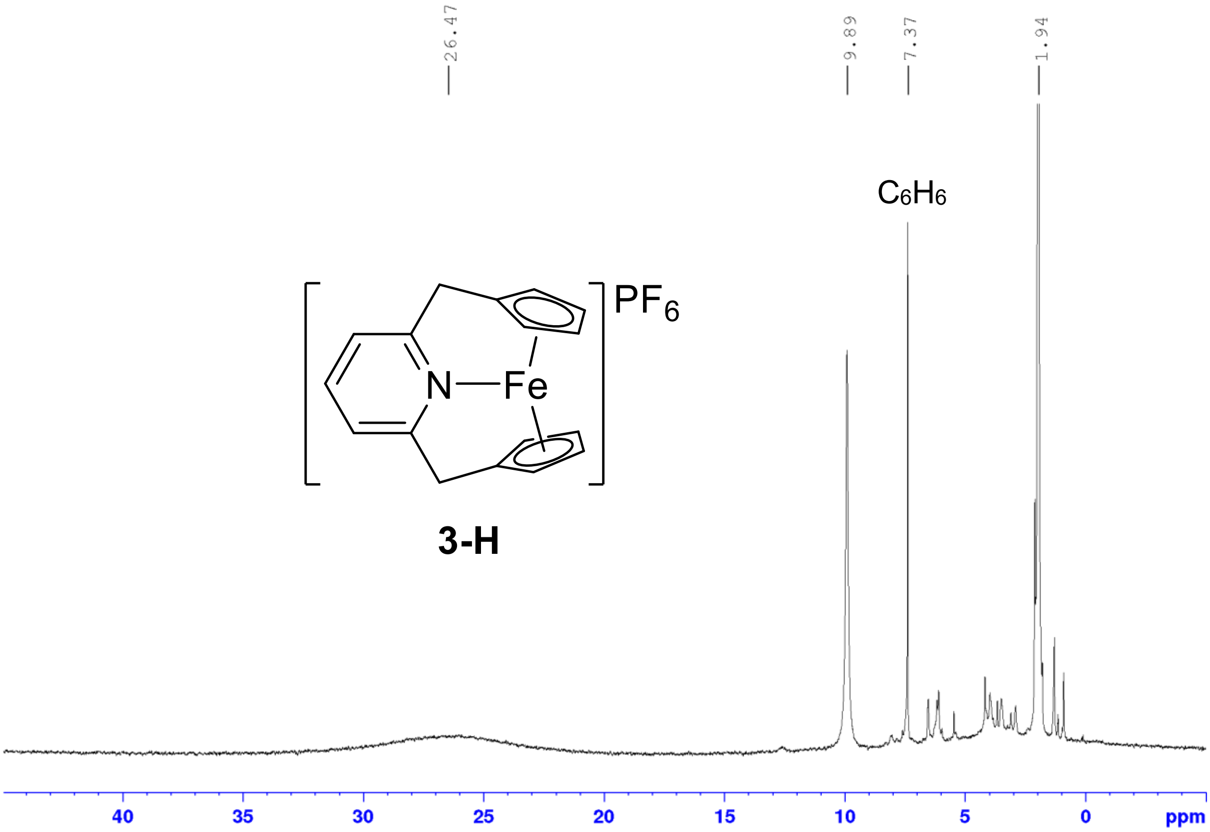

b

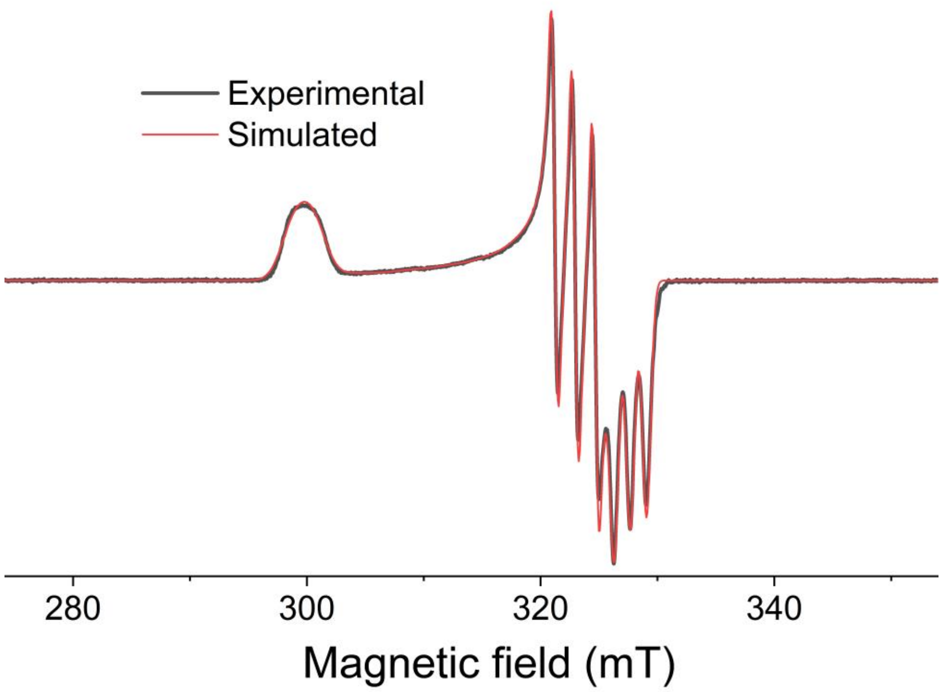

Supplementary Fig. 38.

(**a**) ^{1}H NMR (CD_3CN, 400.15 MHz, 298 K) and (**b**) X-band EPR (toluene/acetone glass, 77 K) spectra of **3-H**.

a

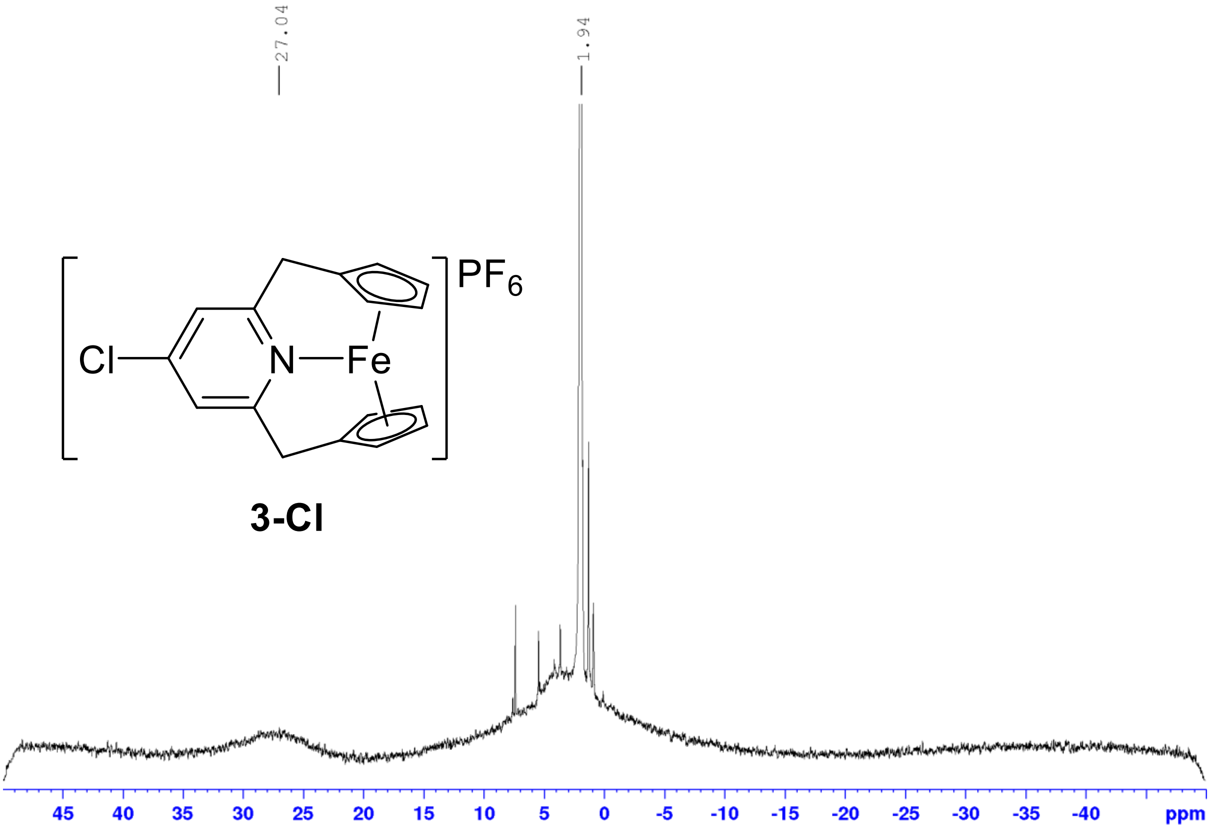

b

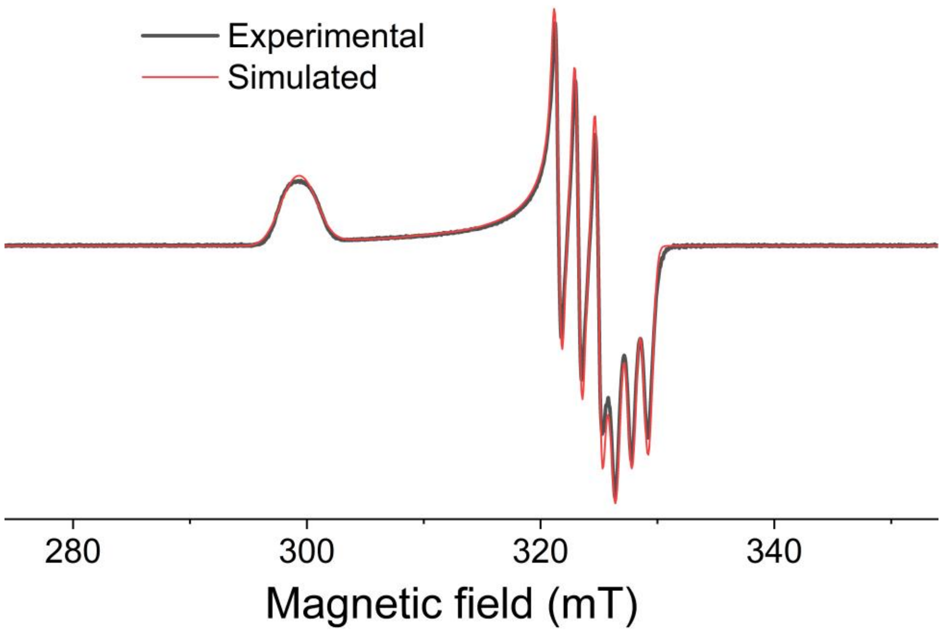

Supplementary Fig. 39.
(**a**) ^{1}H NMR (CD_3CN, 400.15 MHz, 298 K) and (**b**) X-band EPR (toluene/acetone glass, 77 K) spectra of **3-Cl**.

a

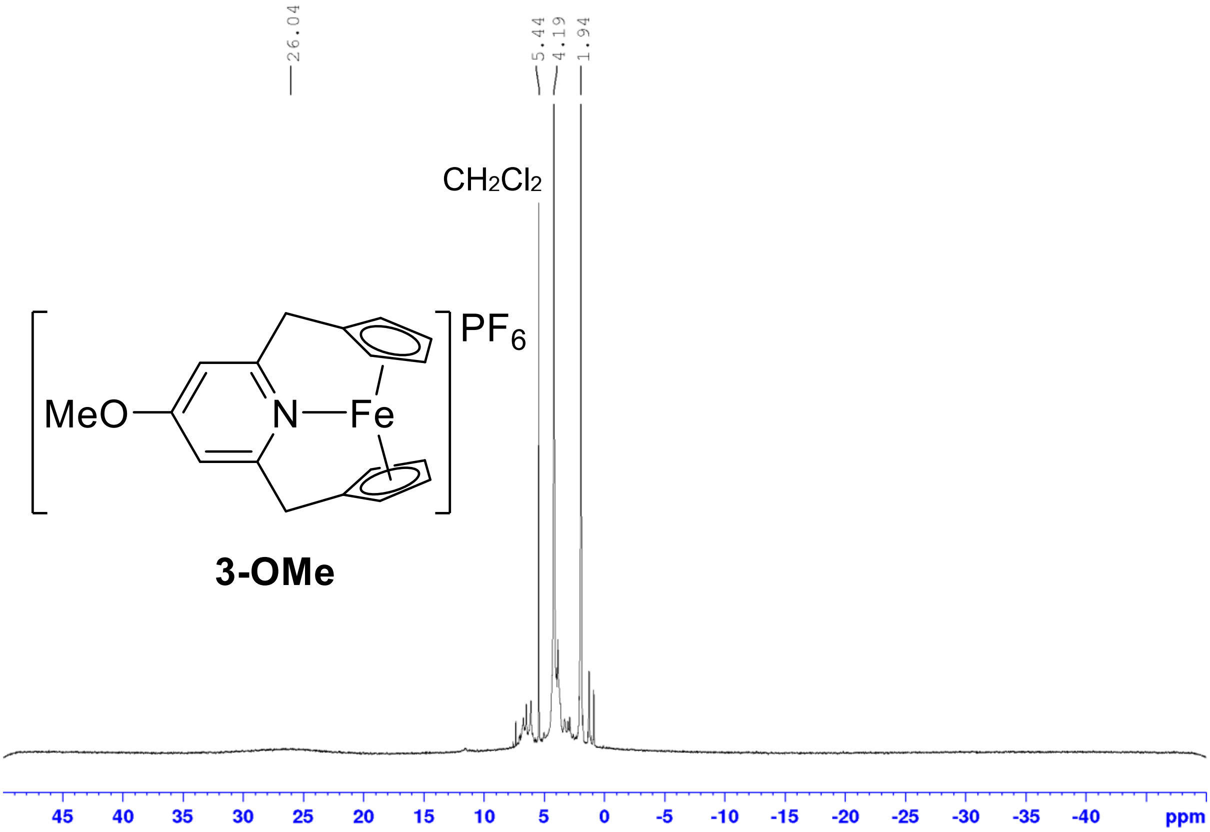

b

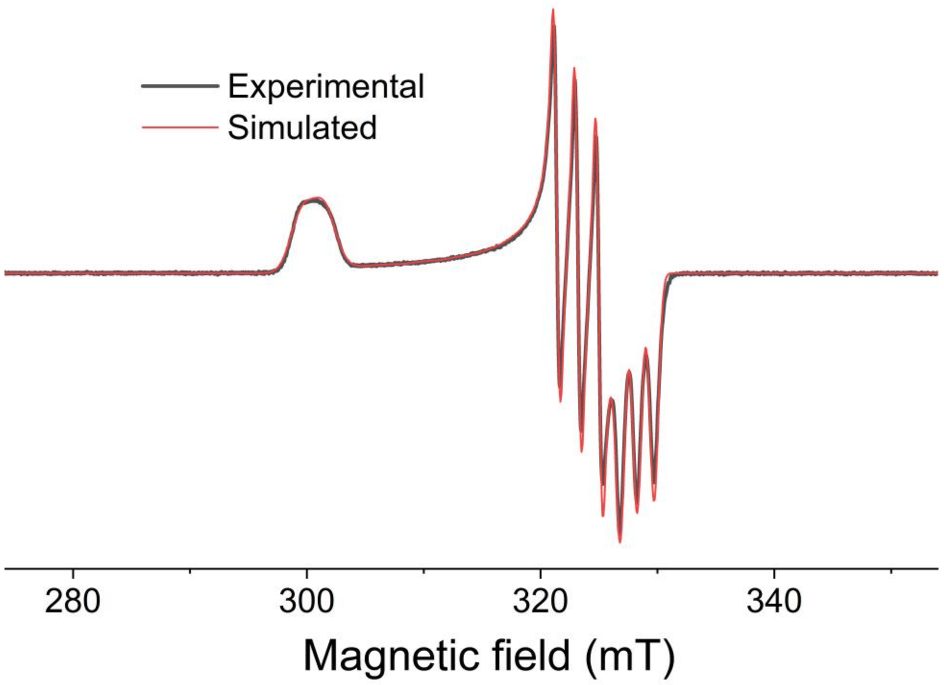

Supplementary Fig. 40.

(**a**) ^{1}H NMR (CD_3CN, 400.15 MHz, 298 K) and (**b**) X-band EPR (toluene/acetone glass, 77 K) spectra of **3-OMe**.

a

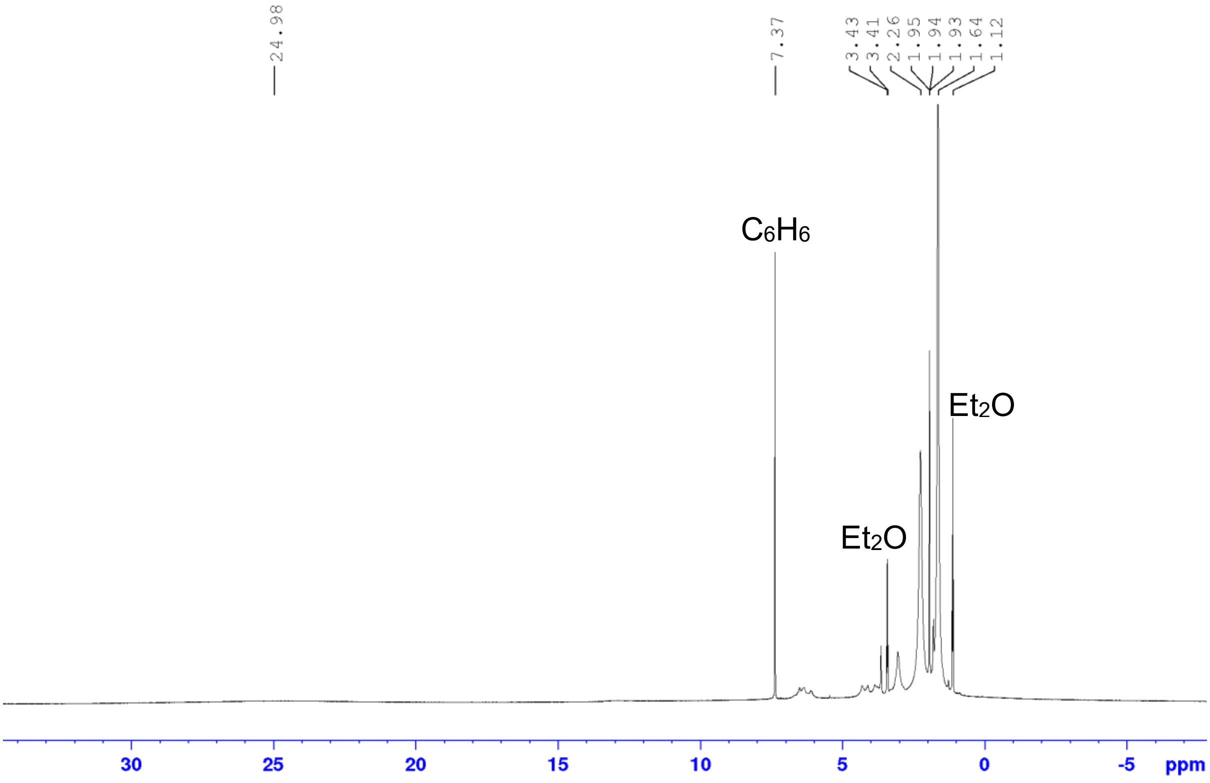

b

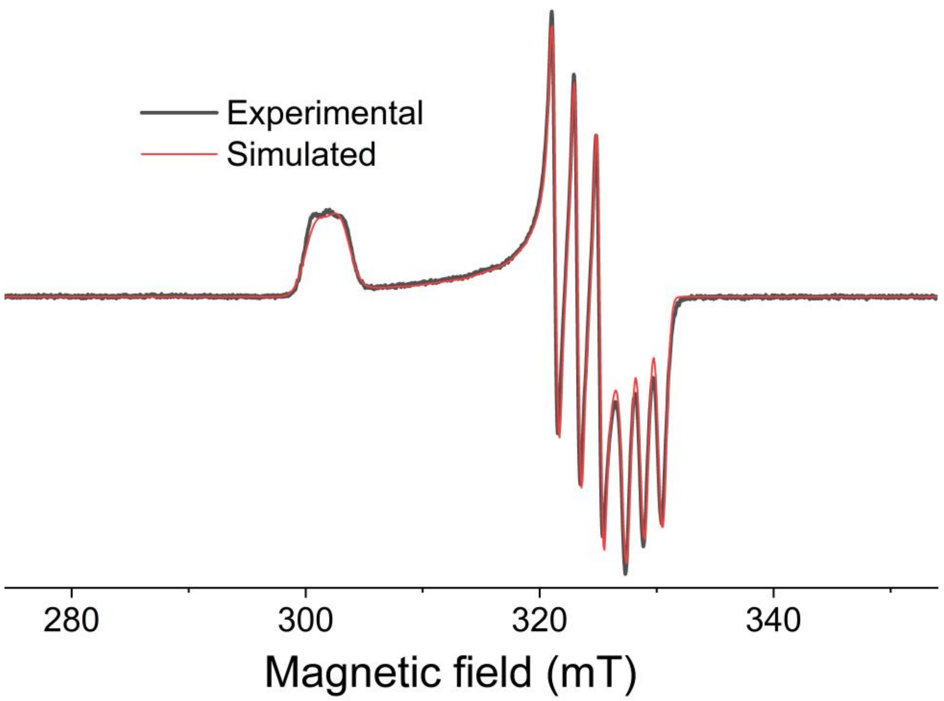

Supplementary Fig. 41.
(**a**) ^{1}H NMR (CD_3CN, 400.15 MHz, 298 K) and (**b**) X-band EPR (toluene/acetone glass, 77 K) spectra of **3-NMe$_2$**.

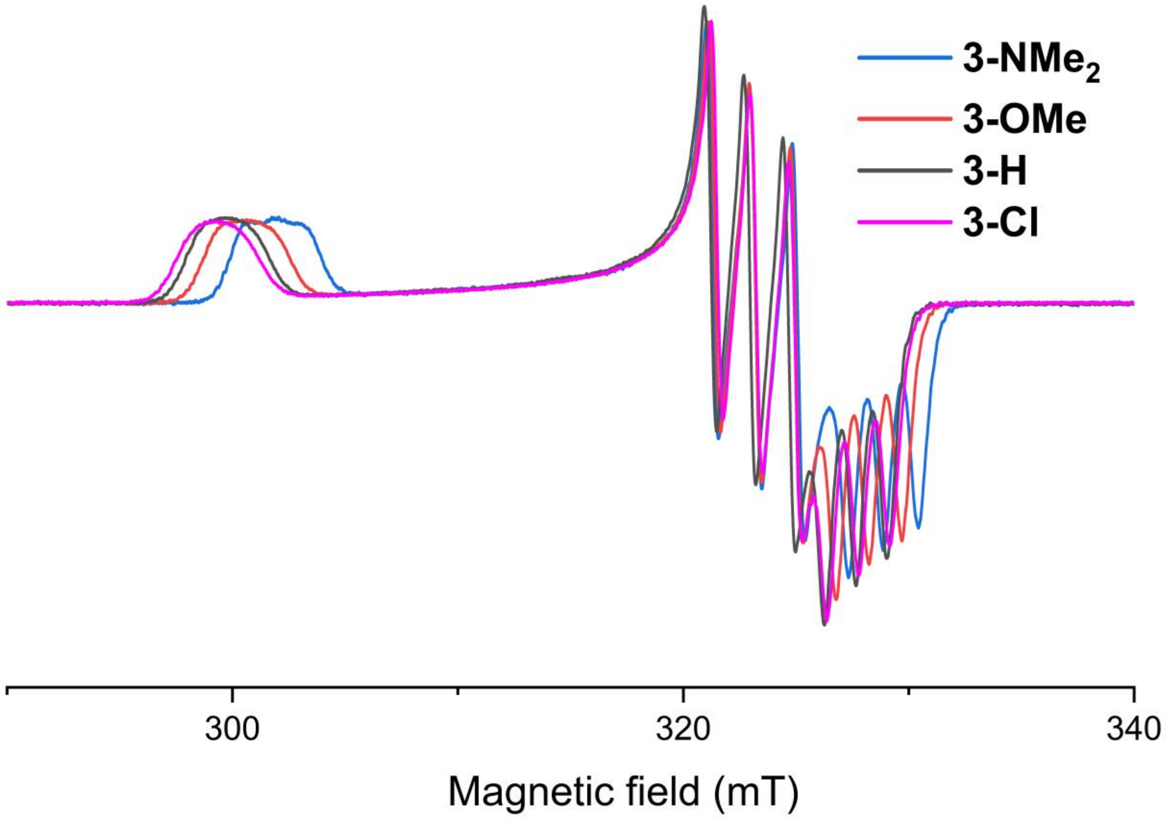

Supplementary Fig. 42.
Overlay of X-band EPR (toluene/acetone glass, 77 K) spectra of **3-X**.

a

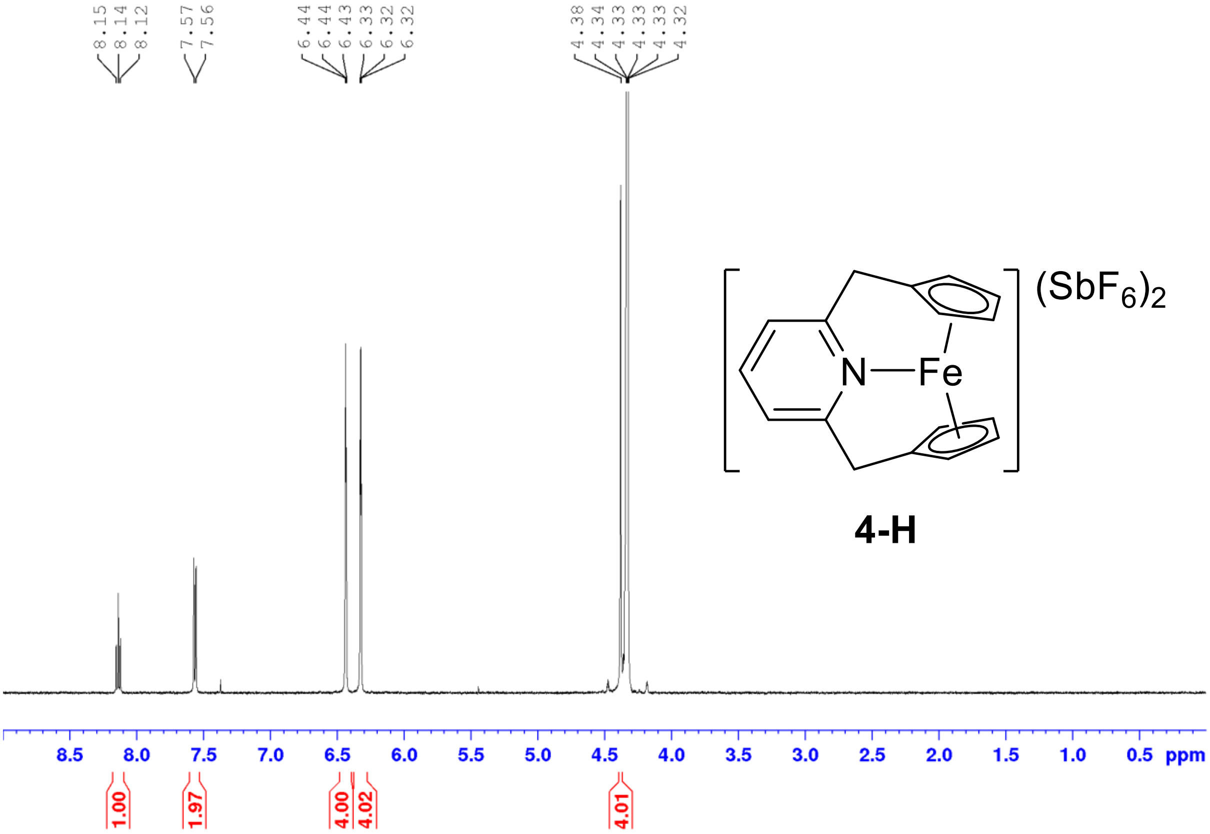

b

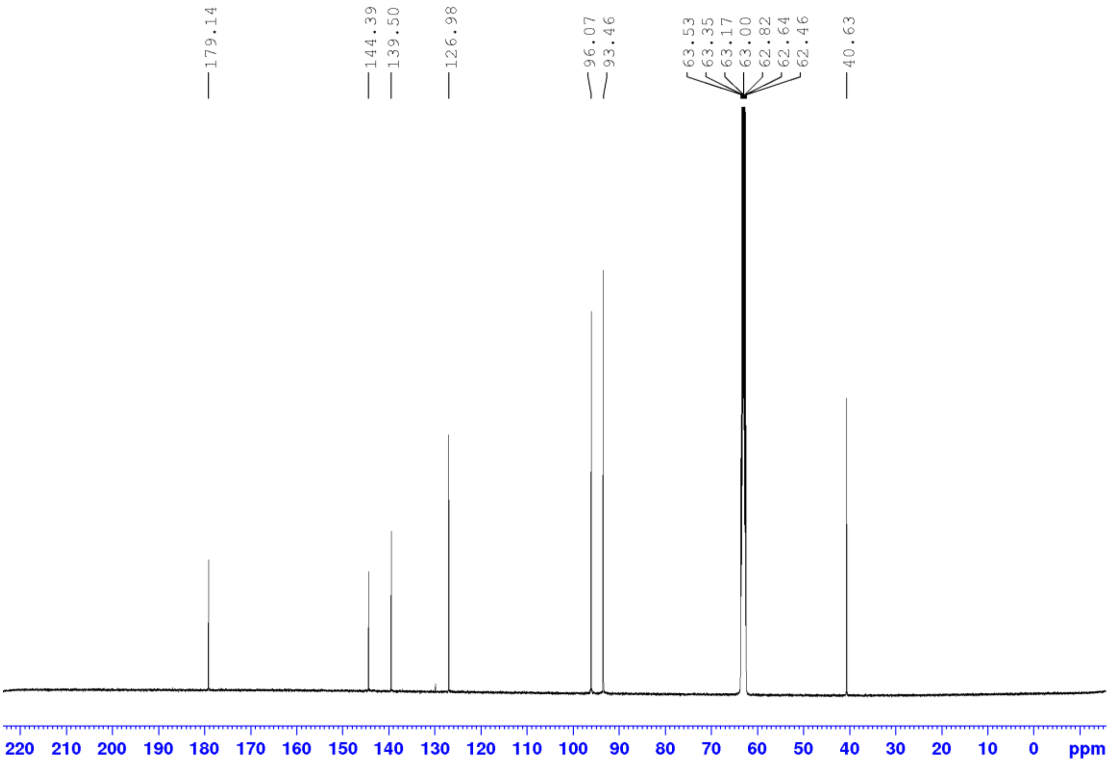

Supplementary Fig. 43.

NMR spectra (CD_3NO_2, 298 K) of **4-H**. (**a**) ^1H NMR (500.13 MHz). (**b**) $^{13}C\{^1H\}$ NMR (125.76 MHz).

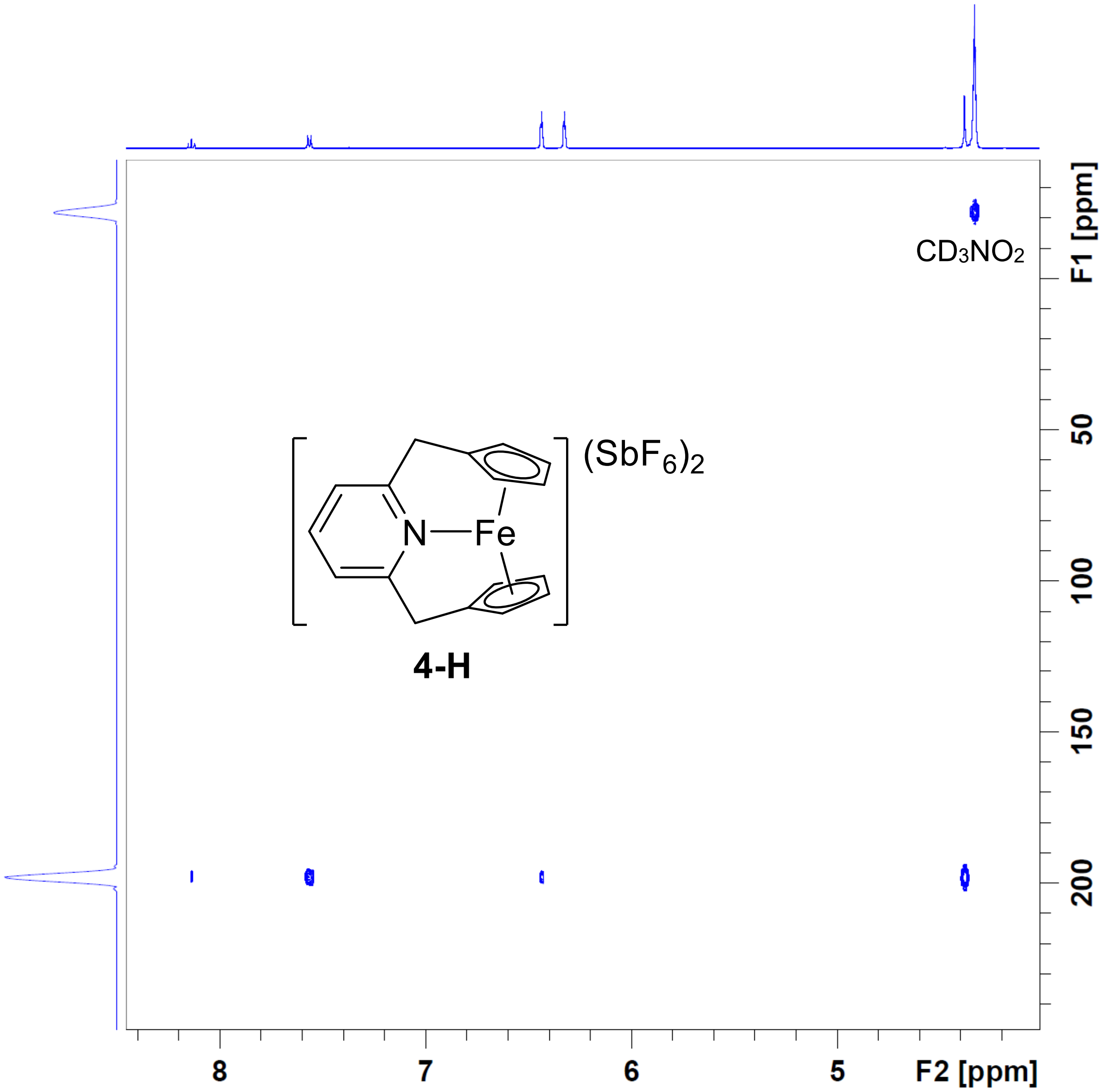

Supplementary Fig. 44.
^{1}H-^{15}N HMBC NMR spectrum (CD_3NO_2, 500.13 MHz, 298 K) of **4-H**.

a

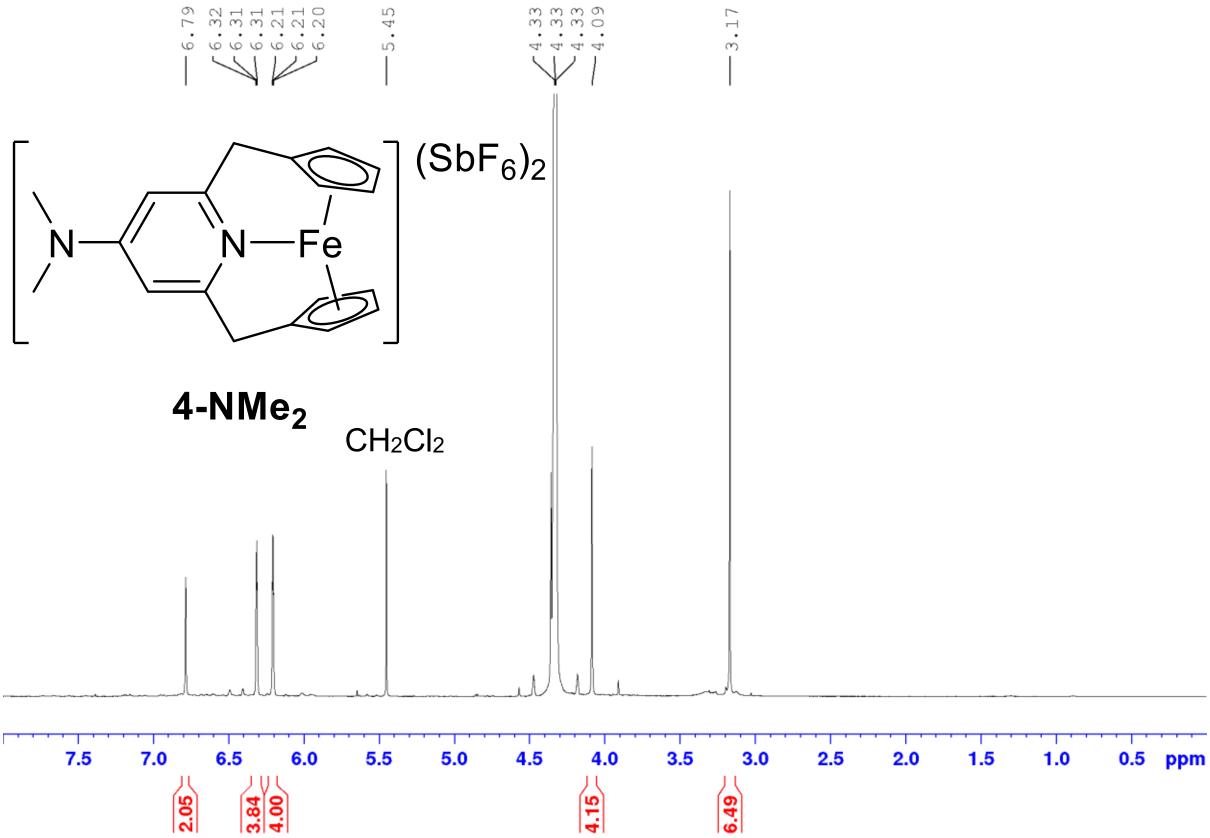

b

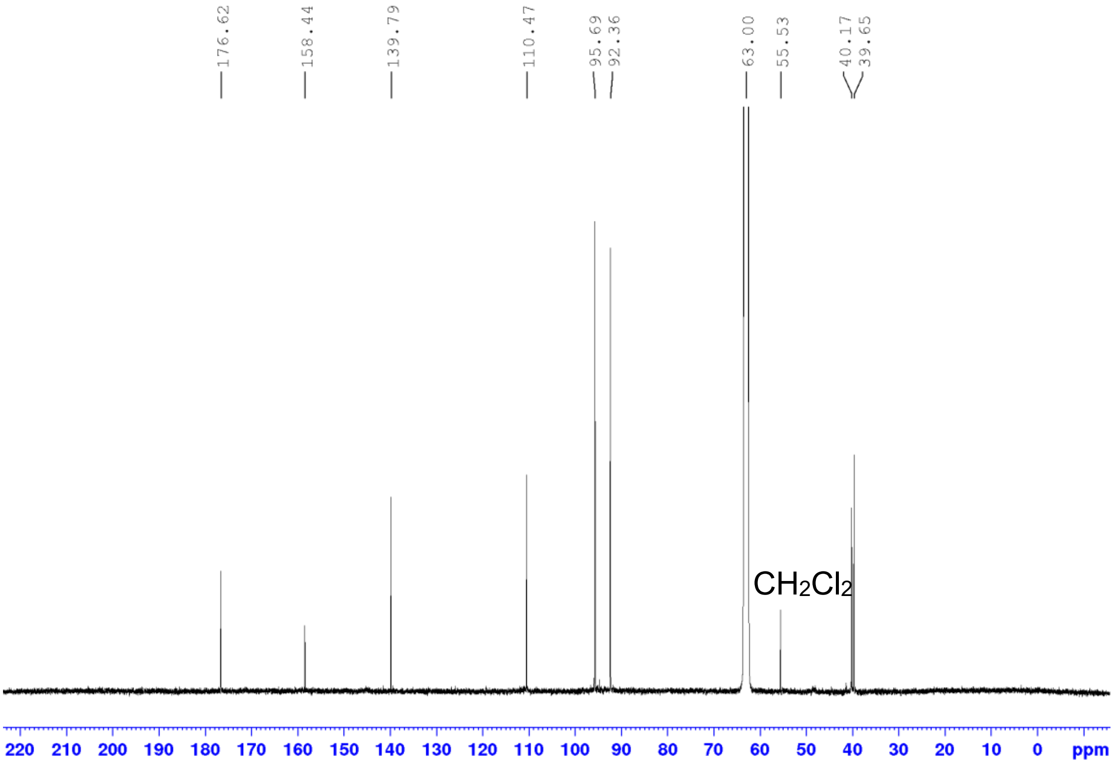

Supplementary Fig. 45.

NMR spectra (CD_3NO_2, 298 K) of **4-NMe2**. (**a**) ^{1}H NMR (500.13 MHz). (**b**) $^{13}C\{^1H\}$ NMR (125.76 MHz).

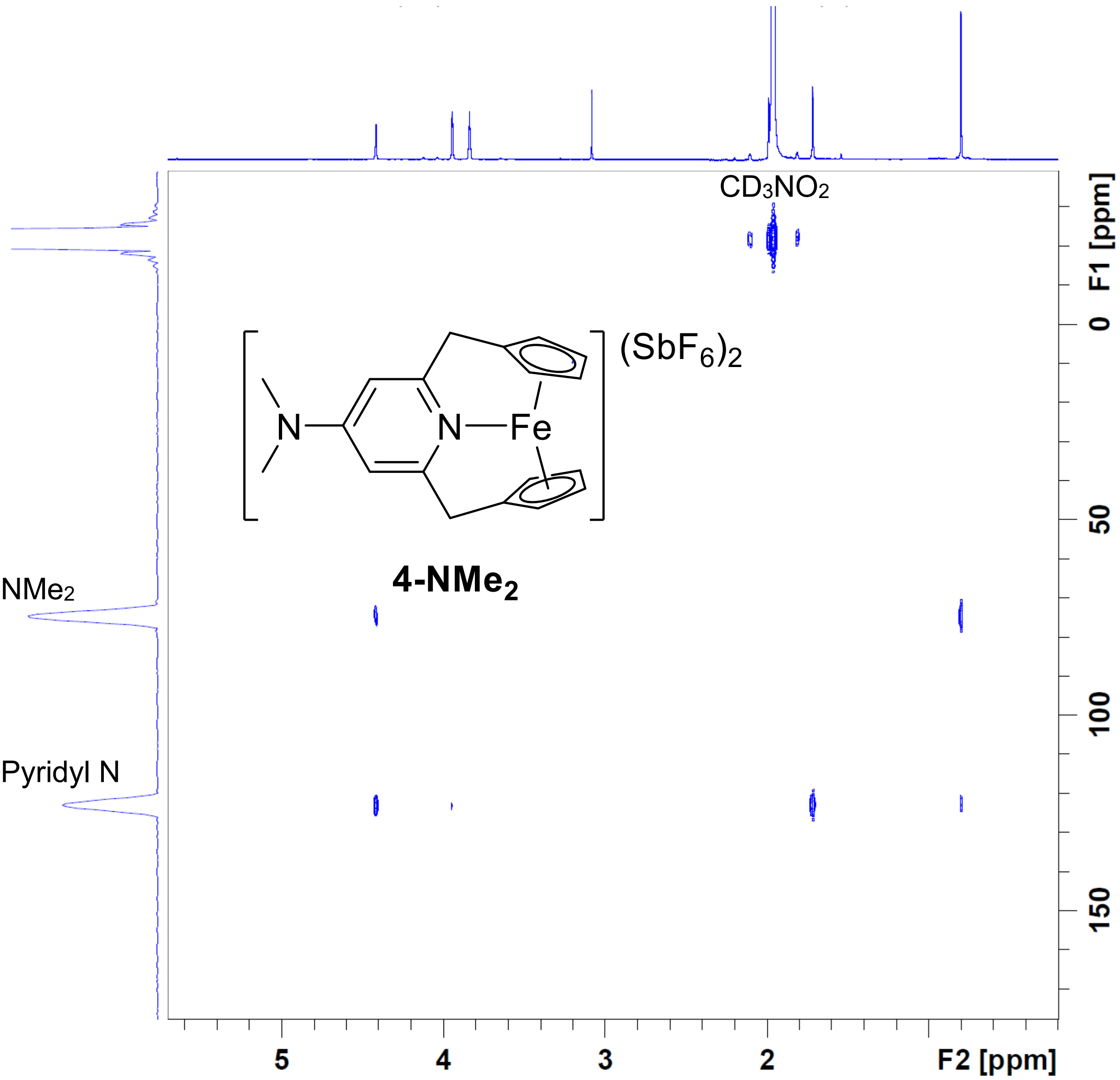

Supplementary Fig. 46.
^{1}H-^{15}N HMBC NMR spectrum (CD_3NO_2, 500.13 MHz, 298 K) of **4-NMe₂**.

a

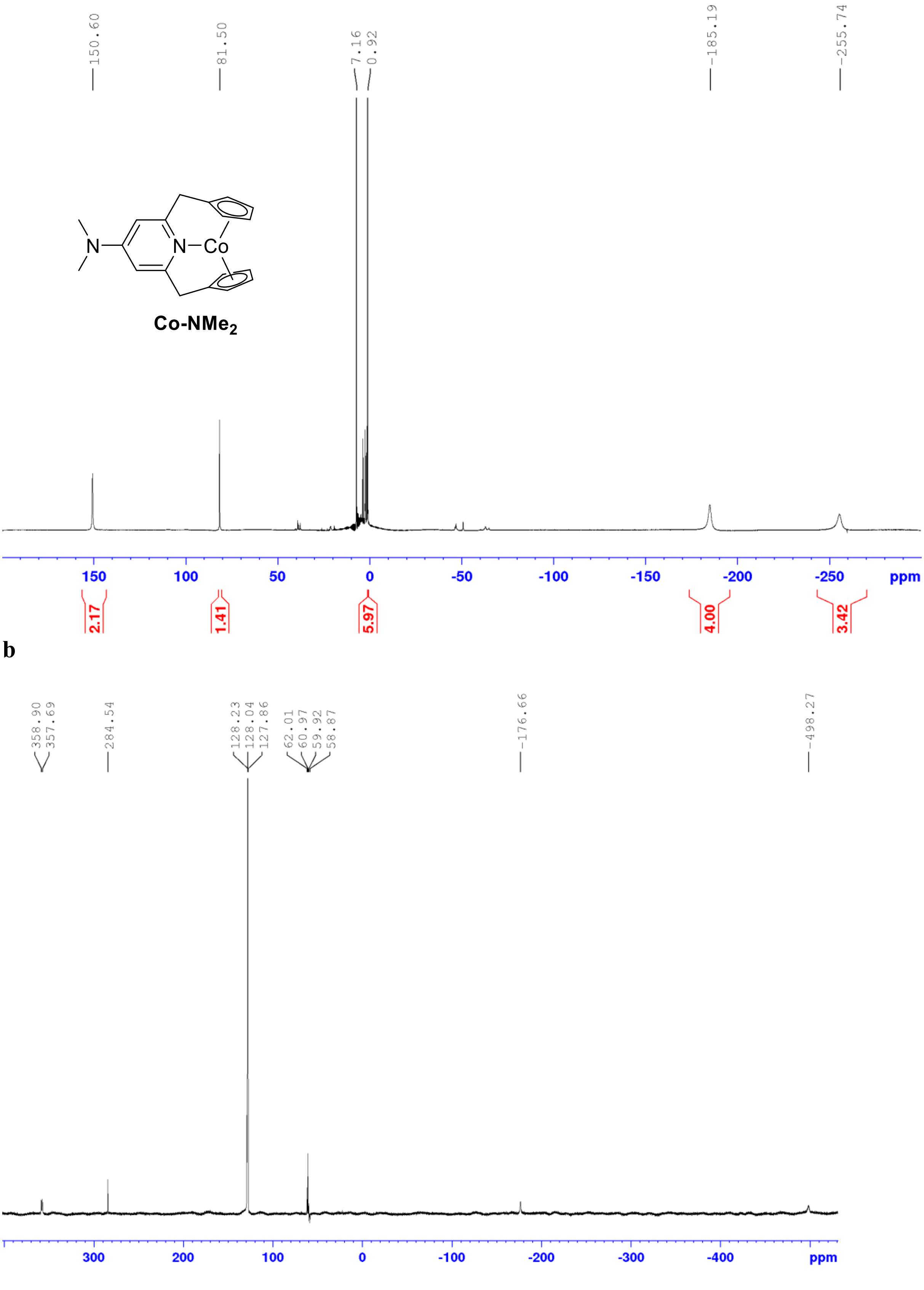

Supplementary Fig. 47.

NMR spectra (C_6D_6, 298 K) of **Co-NMe2**. (**a**) ^{1}H NMR (500.13 MHz). (**b**) ^{13}C NMR (125.76 MHz).

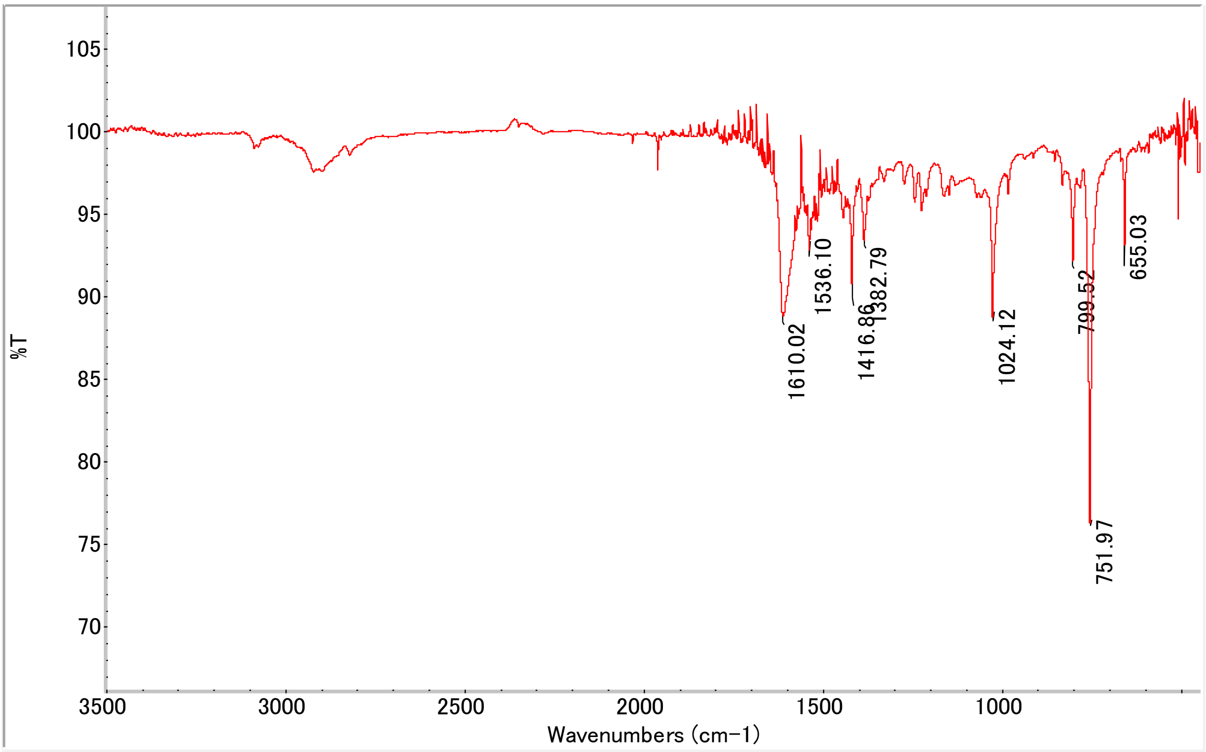

Supplementary Fig. 48.
FTIR spectrum (thin film) of **Co-NMe2**.

a

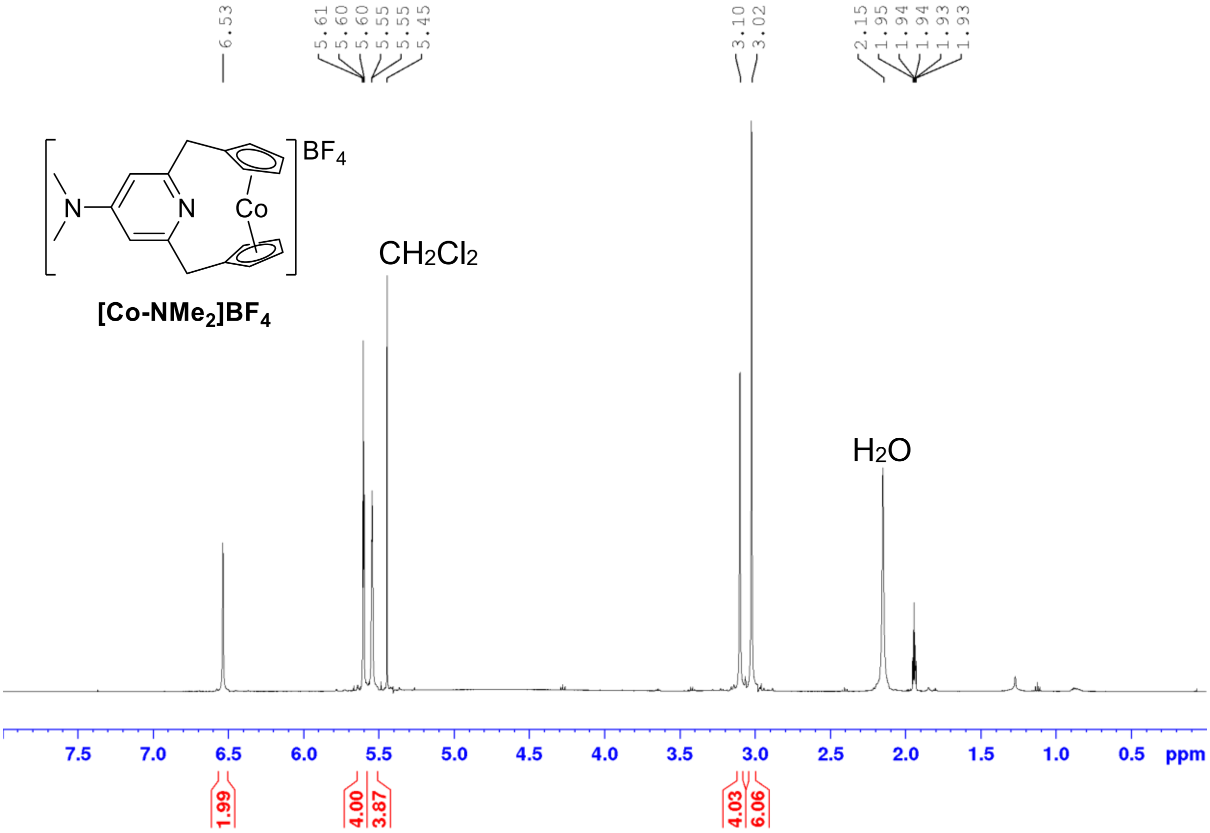

b

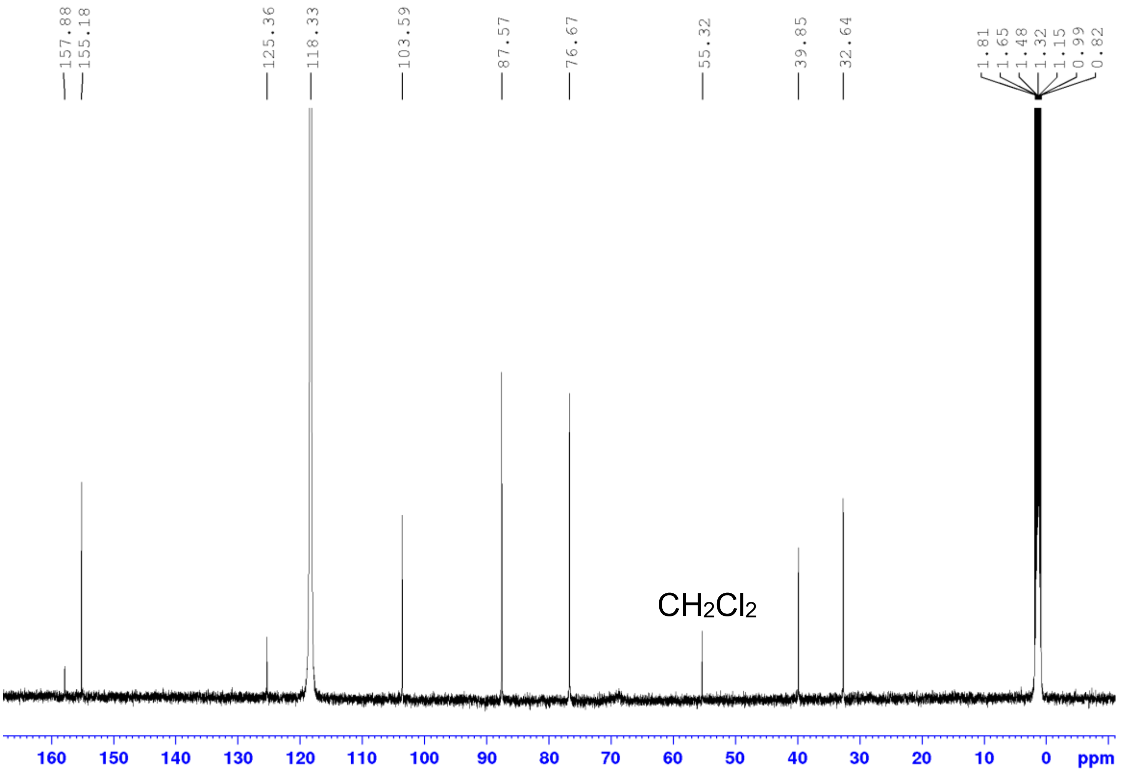

Supplementary Fig. 49.

NMR spectra (CD_3CN, 298 K) of **[Co-NMe₂]BF₄**. (**a**) ^{1}H NMR (500.13 MHz). (**b**) $^{13}C\{^{1}H\}$ NMR (125.76 MHz).

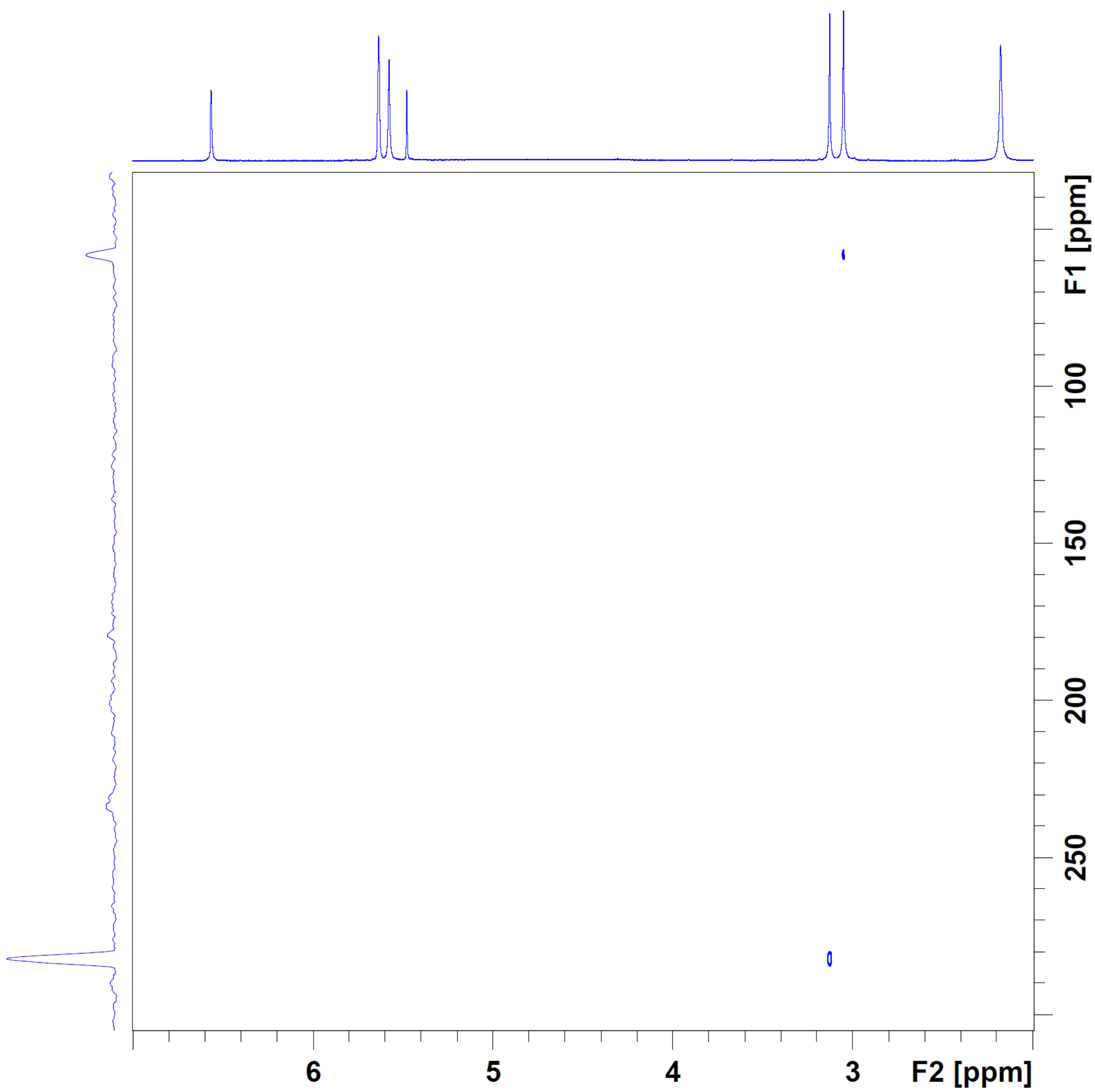

Supplementary Fig. 50.
^{1}H-^{15}N HMBC NMR spectrum (CD_3CN, 500.13 MHz, 298 K) of **[Co-NMe$_2$]BF$_4$**.

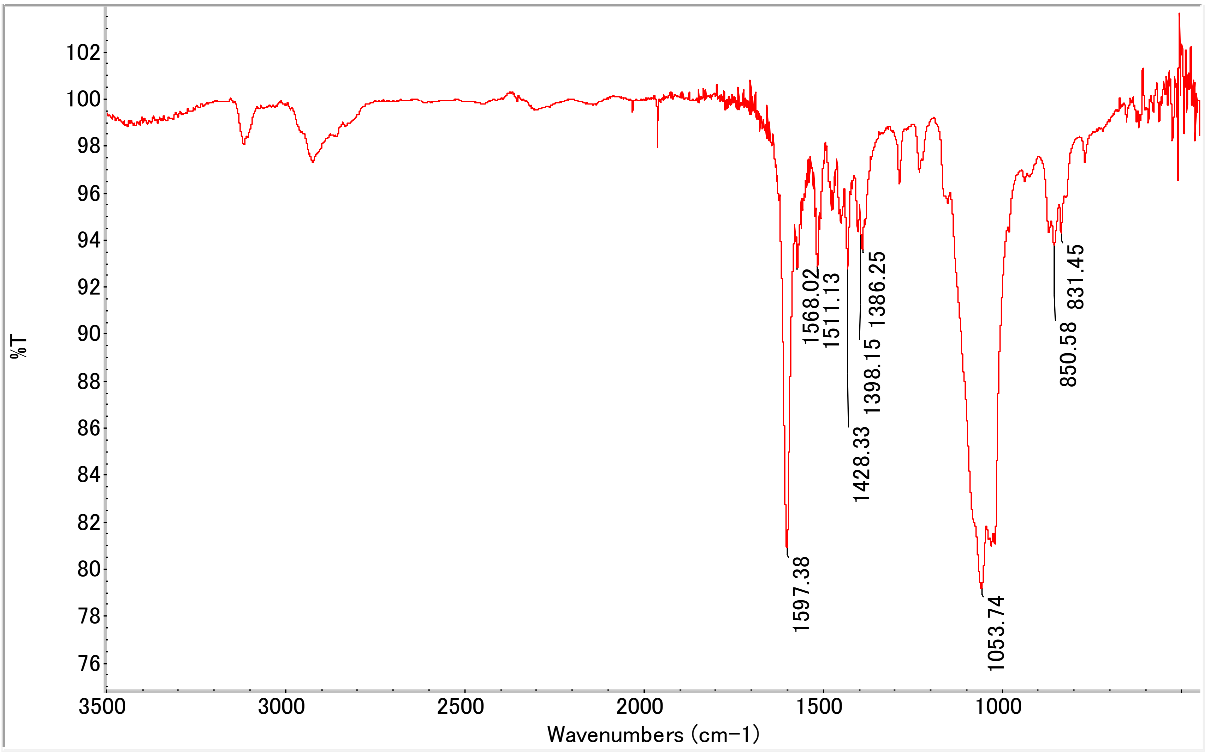

Supplementary Fig. 51.
FTIR spectrum (thin film) of **[Co-NMe$_2$]BF$_4$**.

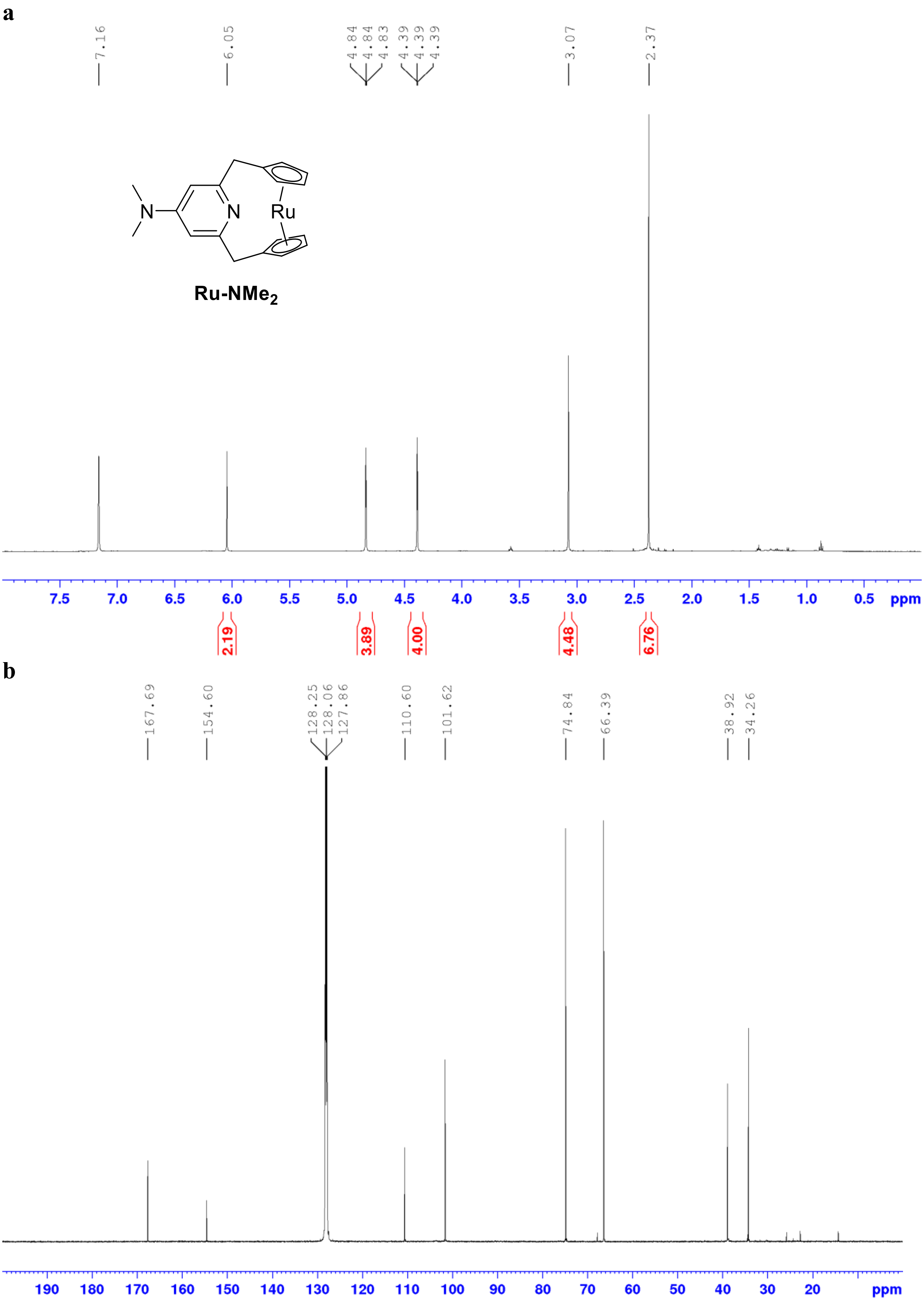

Supplementary Fig. 52.

NMR spectra (C_6D_6, 298 K) of **Ru-NMe₂**. (**a**) ^{1}H NMR (500.13 MHz). (**b**) $^{13}C\{^{1}H\}$ NMR (125.76 MHz).

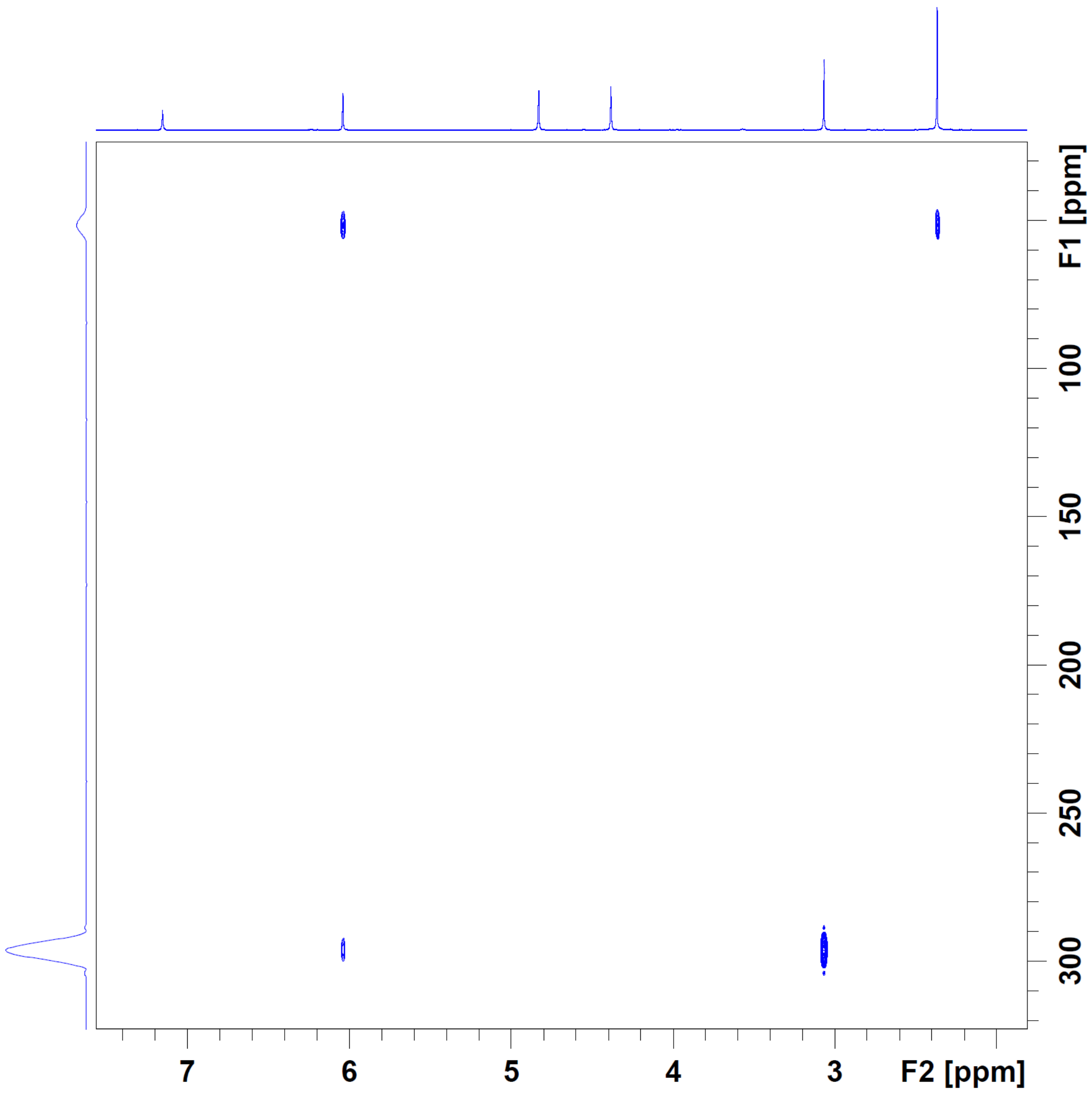

Supplementary Fig. 53.
^{1}H-^{15}N HMBC NMR spectrum (C_6D_6, 500.13 MHz, 298 K) of **Ru-NMe₂**.

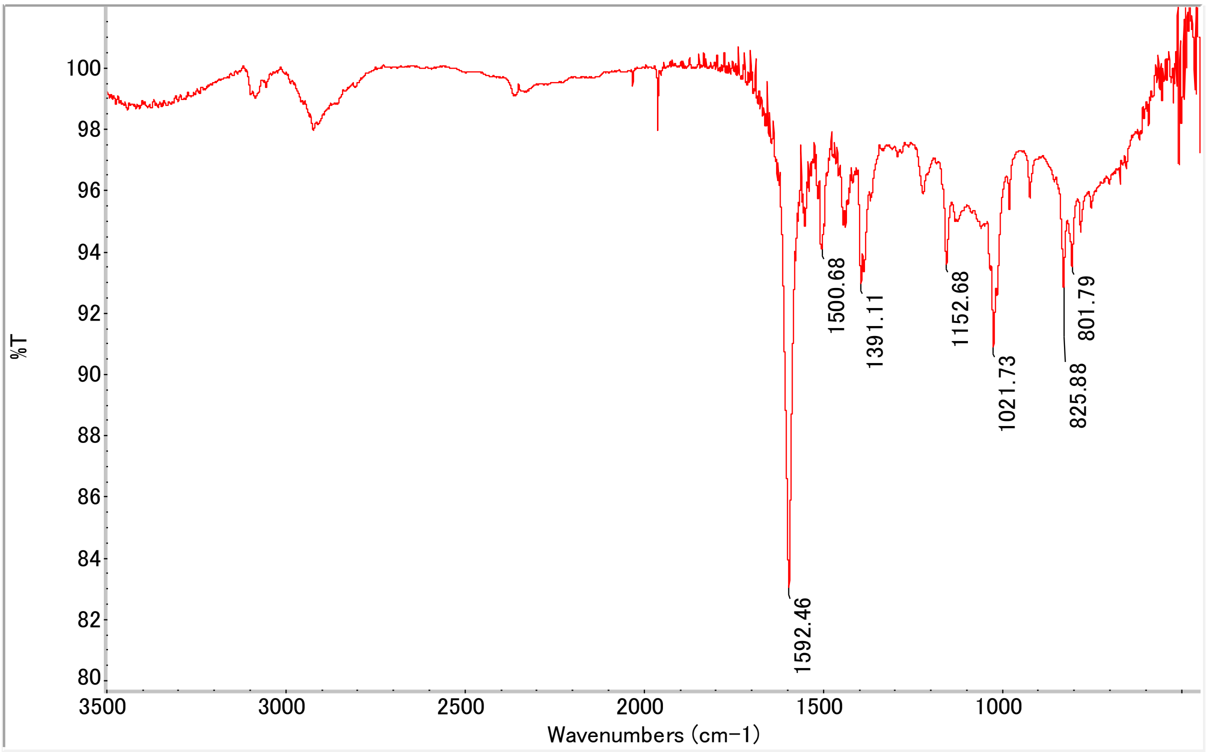

Supplementary Fig. 54.
FTIR spectrum (thin film) of **Ru-NMe$_2$**.

Supplementary Note 1. Preparation of ligands

Preparation of 2,6-bis(methylenecyclopentadienyl)pyridine disodium salt (**H-CpNCpNa2**)

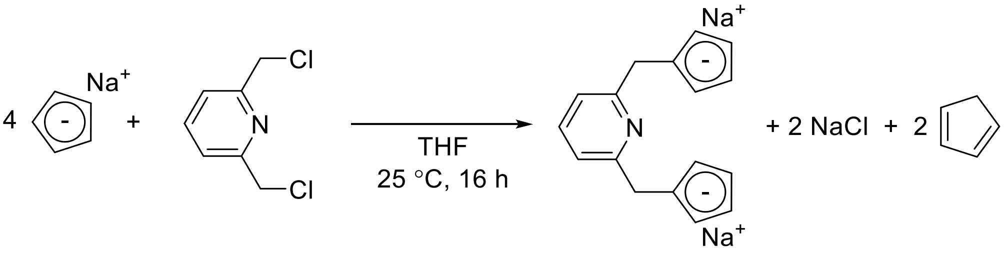

H-CpNCpNa2

A 20 mL vial equipped with a Teflon coated stirring bar was added NaCp (4.71 mL, 2.55 M THF solution from Aldrich, purple viscous solution, 12.0 mmol). 2,6-bis(chloromethyl)pyridine (528.1 mg, 3.00 mmol) in 6 mL THF was added to the vial dropwise using a pipet for about 5 min. The solution warmed up to about 50 °C because of the exothermic reaction and white precipitate of NaCl formed. The mixture was stirred for 16 h at 25 °C. The resulting pink solution and white precipitate was filtered using a plug of Celite prepared using a pipet. The Celite was washed three times with 1 mL each of THF, and the filtered pink solution was concentrated under vacuum. White crystalline solid of the product formed on the glass wall upon concentration to about 2 mL. More product precipitated from the concentrated solution upon storing the solution at -35 °C for overnight. The pink supernatant was decanted and the remaining solid was washed three times with cold diethyl ether to remove unreacted NaCp and pink colored material. The resulting white solid was dried overnight under high vacuum (< 0.2 mmHg) to remove coordinated THF molecules. Yield: 528.4 mg, 63%.
^{1}H NMR (500.13 MHz, THF-d_8, 298 K): δ 3.93 (4H, s, 2 CH_2), 5.34 (4H, pseudo t, 2,5-position of C_5H_4 groups), 5.52 (4H, pseudo t, 3,4-position of C_5H_4 groups), 6.89 (2H, d, 3 J_{HH}, = 7.5 Hz, 3,5-position of pyridine ring), 7.40 (1H, d, 3 J_{HH}, = 7.5 Hz, 4-position of pyridine ring).

Preparation of 4-chloro-2,6-bis(methylenecyclopentadienyl)pyridine disodium salt (**Cl-CpNCpNa2**)

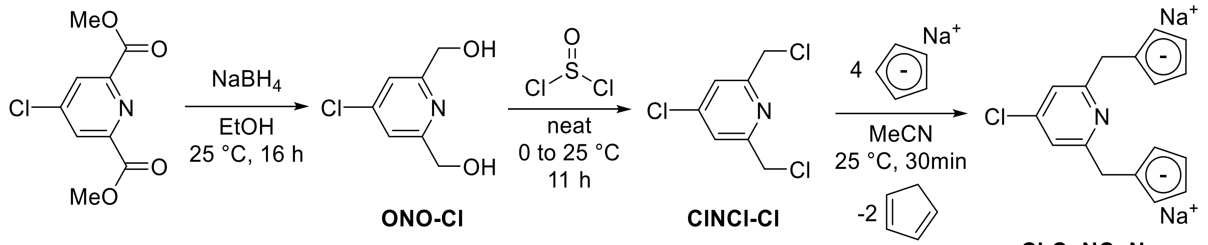

Preparation of ONO-Cl: ONO-Cl was prepared using a modified reported procedure[1]. A 50 mL round bottom flask equipped with a Teflon-coated magnetic stirring bar was charged with 4-chloro-2,6-pyridinedicarboxylic acid dimethyl ester (2.3026 g, 10.0 mmol, purchased from BLD Pharm) and 40 mL 99% ethanol. $NaBH_4$ (1.58 g, 41.8 mmol) was added to the flask at 25 °C under air. This exothermic reaction generates a large amount of H_2, so the addition of NaBH4 should be done portion-wise in a fume hood. The solution was stirred for 16 h at 25 °C. The initial red solution became a yellow solution after 16 h. The reaction was quenched by the addition of 5 mL water at 0 °C. The solution was concentrated to dryness and about 50 mL of sat. Na_2CO_3 was added to the flask. The flask was equipped with a condenser, and the

solution was heated at 100 °C for 1 h. The resulting biphasic mixture was extracted using EtOAc, and the EtOAc solution was dried by $MgSO_4$, and $MgSO_4$ was filtered out. The concentration of EtOAc solution gave a faint yellow solid of **ONO-Cl**. Yield: 1.5779 g, 91%. NMR spectra of **ONO-Cl** matched with the reported values[1].
^{1}H NMR (400.15 MHz, CD_3OD, 298 K): δ 4.66 (4H, s, 2 C*H_2*), 7.45 (2H, s, 3,5-position of pyridine ring).

Preparation of ClNCl-Cl: A 250 mL round bottom flask equipped with a Teflon-coated magnetic stirring bar was charged with **ONO-Cl** (1.576 g, 9.09 mmol). 12 mL thionyl chloride was added to the flask at 0 °C under air, and the solution was stirred for 11 h at 25 °C. The reaction was quenched by slow addition of 20 mL water (CAUTION: exothermic reaction with a formation of HCl gas) at 0 °C followed by slow addition of sat. Na_2CO_3 (CAUTION: exothermic reaction with a formation of CO_2 gas) until the evolution of CO_2 stopped and the solution became basic. The resulting floating white solid was filtered and washed three times with water and dried in a desiccator. Yield: 1.7644 g, 92%. NMR spectra of **ClNCl-Cl** matched with the reported values[1].
^{1}H NMR (400.15 MHz, $CDCl_3$, 298 K): δ 4.63 (4H, s, 2 C*H_2*), 7.47 (2H, s, 3,5-position of pyridine ring).

Preparation of Cl-CpNCpNa$_2$: In a nitrogen glovebox, a 20 mL vial equipped with a Teflon-coated stirring bar was charged with 1.5 M NaCp (1.4 mL in MeCN, 2.1 mmol) and 2 mL MeCN. **ClNCl-Cl** (105.5 mg, 0.501 mmol) in 2 mL MeCN was added to the solution dropwise for 5 min, the vial containing **ClNCl-Cl** was washed with 1 mL MeCN, and the MeCN solution was added to the reaction mixture. The solution was stirred at 25 °C for 30 min. The reaction mixture was passed through a plug of Celite using MeCN, and the resulting yellow solution was concentrated to produce a yellow oil. Addition of ether to the oil formed a small amount of white precipitate. The addition of *n*-pentane to the ether solution gave more off-white solid. The solid was decanted and washed three times with 1:1 *n*-pentane:ether mixture and dried. Yield: 111.9 mg, 58 % (contains a molecule of THF). Supplementary Fig. 1 shows ^{1}H and ^{13}C{^{1}H}NMR spectra of **Cl-CpNCpNa$_2$**.
^{1}H NMR (500.13 MHz, THF-*d_8*, 298 K): δ 3.91 (4H, s, 2 *CH_2*), 5.34 (4H, broad pseudo t, 2,5-position of *C_5H_4* groups), 5.52 (4H, broad m, 3,4-position of *C_5H_4* groups), 7.00 (2H, s, 3,5-position of pyridine ring).
^{13}C{^{1}H} NMR (125.76 MHz, THF-*d_8*, 298 K): δ 38.7 (s, *C*H_2), 102.4 (s, 3,4-position of *C_5H_4* groups), 103.4 (s, 2,5-position of *C_5H_4* groups), 113.8 (s, 1-position of *C_5H_4* groups), 118.8 (s, 3,5-position of pyridine ring), 143.2 (s, 4-position of pyridine ring), 167.2 (s, 2,6-position of pyridine ring).
^{15}N NMR (50.68 MHz, THF-*d_8*, 298 K, detected using ^{1}H-^{15}N HMBC): δ 294.3 (s).
HRMS (ESI/TOF, $[M + 3H]^+$): Calcd for $C_{17}H_{17}N_1Cl_1$: 270.1044. Found: 270.1037.

Preparation of 4-methoxy-2,6-bis(methylenecyclopentadienyl)pyridine disodium salt (**OMe-CpNCpNa2**)

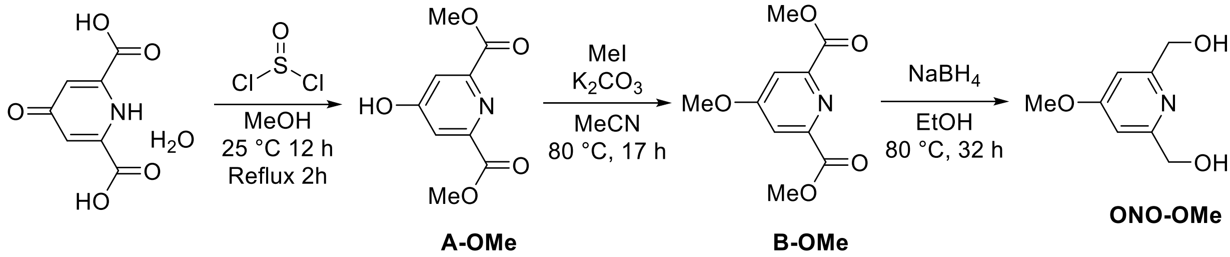

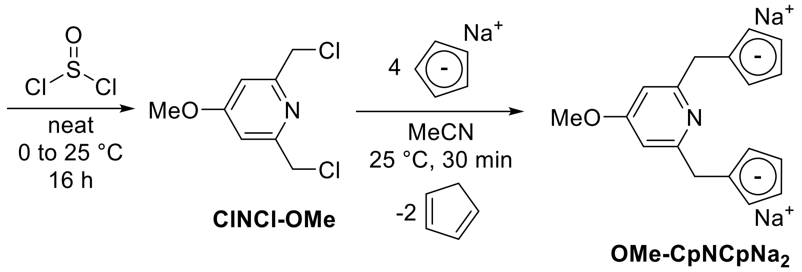

Preparation of B-OMe: B-OMe was prepared using a modified reported procedure[2]. A 100 mL round bottom flask equipped with a Teflon-coated magnetic stirring bar was charged with Chelidamic acid hydrate (4.0195 g, 19.9 mmol, purchased from BLD Pharm) and 40 mL methanol. 11.4 mL thionyl chloride was added dropwise to the flask at 0 °C under air, and the solution was stirred for 12 h at 25 °C. The solution was refluxed for 2 h under air, cooled down to r.t., and concentrated to dryness. A dark brown solid was obtained. ^{1}H NMR (in DMSO-d_6) spectrum of the solid showed clean formation of **A-OMe**.

^{1}H NMR (400.15 MHz, DMSO-d$_6$, 298 K): δ 3.87 (6H, s, 2 COOCH_3), 7.60 (2H, s, 3,5-position of pyridine ring), OH signal merged with a water peak.

K_2CO_3 (6.91g, 50.0 mmol), MeI (5 mL, 80 mmol), and 50 mL MeCN were then added to the flask. The solution was stirred for 17 h at 80 °C and cooled to r.t. Solid byproducts were removed by vacuum filtration. The brown solution was extracted using dichloromethane/water to remove KI, the dichloromethane layer was dried by $MgSO_4$, and $MgSO_4$ was removed by filtration. The concentration of the dichloromethane solution gave a brown solid of **B-OMe**. Yield: 4.3628 g, 97 % (two steps). ^{1}H NMR (in $CDCl_3$) spectrum showed clean formation of **B-OMe**[2].

^{1}H NMR (500.13 MHz, $CDCl_3$, 298 K): δ 3.98 (3H, s, OCH_3), 4.02 (6H, s, 2 COOCH_3), 7.60 (2H, s, 3,5-position of pyridine ring), OH signal merged with a water peak.

Preparation of ONO-OMe: ONO-OMe was prepared using a reported procedure[3].

A 50 mL round bottom flask equipped with a Teflon-coated magnetic stirring bar was charged with **B-OMe** (4.3633 g, 19.4 mmol), $NaBH_4$ (2.93 g, 77 mmol), and 50 mL 99% ethanol at 25 °C under air. The solution was stirred for 12 h at 80 °C. GC-MS analysis of the crude mixture showed presence of **ONO-OMe** and a partially hydrogenated product. $NaBH_4$ (2.99 g, 79 mmol) was added to the flask and the solution was stirred for 24 h at 80 °C. The reaction was quenched by the addition of about 10 mL sat. Na_2CO_3. The solution was concentrated to dryness and the solid was extracted using EtOAc, and the EtOAc solution was dried by $MgSO_4$, and $MgSO_4$ was filtered out. The concentration of EtOAc solution gave a faint yellow solid of **ONO-OMe**. Yield: 2.49 g, 76 %.

^{1}H NMR (400.15 MHz, CD_3OD, 298 K): δ 3.90 (3H, s, OCH_3), 4.62 (4H, s, 2 CH_2), 6.98 (2H, s, 3,5-position of pyridine ring).

Preparation of ClNCl-OMe: A 1 L round bottom flask equipped with a Teflon-coated magnetic stirring bar was charged with **ONO-OMe** (2.49 g, 14.7 mmol). 20 mL thionyl chloride was added to the flask at 0 °C under air, and the solution was stirred for 16 h at 25 °C. The reaction was quenched by slow addition of 20 mL water (CAUTION: exothermic reaction with a formation of HCl gas) at 0 °C followed by slow addition of sat. Na_2CO_3 (CAUTION: exothermic reaction with a formation of CO_2 gas) until the evolution of CO_2 stopped and the solution became basic. The resulting floating white solid was filtered and washed three times with water and dried in a desiccator. Yield: 2.2528 g, 74 %. NMR spectra of **ClNCl-OMe** matched with the reported values[4].
^{1}H NMR (500.13 MHz, $CDCl_3$, 298 K): δ 3.90 (3H, s, CH_3), 4.61 (4H, s, 2 CH_2), 7.00 (2H, s, 3,5-C*H* of the pyridine ring).
^{13}C{^{1}H} NMR (125.76 MHz, $CDCl_3$, 298 K): δ 46.6 (s, CH_2), 55.6 (s, CH_3), 108.3 (s, 3,5-*C*H of the pyridine ring), 158.1 (s, 2,6-*C* of the pyridine ring), 167.6 (s, 4-*C* of the pyridine ring).
HRMS (ESI/Orbitrap, $[M + H]^+$): Calcd for $C_8H_{10}NOCl_2$: 206.0134. Found: 206.0130.

Preparation of OMe-CpNCpNa$_2$: In a nitrogen glovebox, a 20 mL vial equipped with a Teflon-coated stirring bar was charged with 2.4 M NaCp (1.7 mL in THF, 4.08 mmol) and 4 mL MeCN. **ClNCl-OMe** (206.7 mg, 1.00 mmol) in 3 mL MeCN was added to the solution dropwise for 5 min, and the vial containing **ClNCl-OMe** was washed with 1 mL MeCN and the MeCN solution was added to the reaction mixture. The solution was stirred at 25 °C for 30 min. The reaction mixture was passed through a plug of Celite using MeCN, and the resulting yellow solution was concentrated to produce purple oil. The oil was washed with 4 mL ether three times and dried. Yield: 283.5 mg, 71%. Supplementary Fig. 2 shows ^{1}H, and ^{13}C{^{1}H} NMR spectra of **OMe-CpNCpNa$_2$**.
^{1}H NMR (500.13 MHz, THF-d_8, 298 K): δ 3.79 (3H, O*Me*), 3.86 (4H, s, 2 CH_2), 5.31 (4H, pseudo t, 2,5-position of C_5H_4 groups), 5.48 (4H, pseudo t, 3,4-position of C_5H_4 groups), 6.50 (2H, s, 3,5-position of pyridine ring).
^{13}C{^{1}H} NMR (125.76 MHz, THF-d_8, 298 K): δ 40.0 (s, CH_2), 55.1 (s, O*Me*), 103.1 (s, 3,4-position of C_5H_4 groups), 104.4 (s, 2,5-position of C_5H_4 groups), 105.7 (s, 3,5-position of pyridine ring),115.6 (s, 1-position of C_5H_4 groups), 167.0 (s, 4-position of pyridine ring), 167.6 (s, 2,6-position of pyridine ring).
^{15}N NMR (50.68 MHz, THF-d_8, 298 K, detected using ^{1}H-^{15}N HMBC): δ 277.8 (s).
HRMS (ESI/TOF, $[M + 3H]^+$): Calcd for $C_{18}H_{20}N_1O_1$: 266.1539. Found: 266.1535.

Preparation of 4-methoxy-2,6-bis(methylenecyclopentadienyl)pyridine disodium salt (**NMe2-CpNCpNa2**)

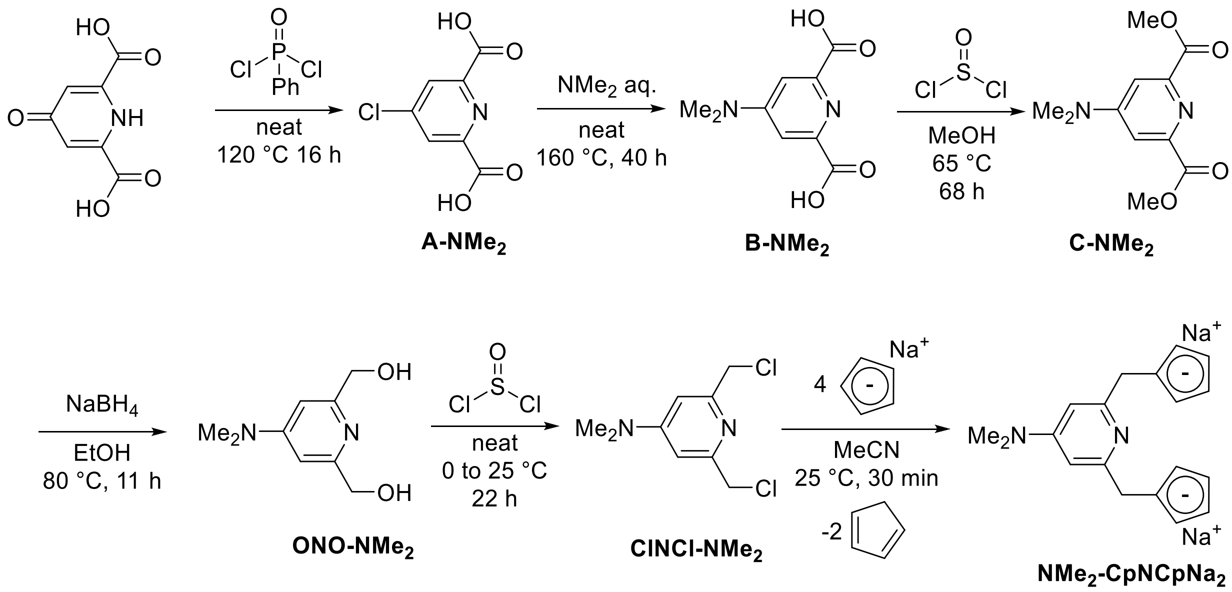

Preparation of A-NMe2 and B-NMe2: A-NMe2 and **B-NMe2** were prepared using a reported procedure[5] from chelidamic acid (BLD Pharm).

A 100mL round bottom flask was charged with chelidamic acid (5.01 g, 27.4 mmol, BLD Pharm) and $PPhOCl_2$ (11 mL, 78 mmol). The solution was heated at 120 °C for 16 h. The solution was cooled to 0 °C and water was added to form a chunk of solid. The solid was filtered and washed three times with water and then three times with dichloromethane. 1 H NMR analysis showed formation of **A-NMe2**.

^{1}H NMR of **A-NMe2** (400.15 MHz, DMSO-d$_6$, 298 K): δ 8.24 (2H, s, 3,5-C*H* of the pyridine ring), COOH signal merged with a water signal.

The solid was charged in a 90 mL Fisher-Porter tube and 16 mL 50% NMe_2H was added to the tube. The tube was sealed and the solution was heated at 160 °C for 40 h. The clear brown solution was transferred to a 200 mL Erlenmeyer flask and acidified by 2 mL H_2SO_4. The brown precipitate was filtered, washed with water and dichloromethane, and dried overnight. Two-step yield: 72%

^{1}H NMR of **B-NMe2** (400.15 MHz, DMSO-d$_6$, 298 K): δ 3.29 (6H, s, N*Me2*), 7.43 (2H, s, 3,5-C*H* of the pyridine ring), COOH signal merged with a water signal.

Preparation of C-NMe2: C-NMe2 was prepared using a modified reported procedure[6]. A 100 mL round bottom flask equipped with a Teflon-coated magnetic stirring bar was charged with **B-NMe2** (2.7025 g, 12.85 mmol) and 30 mL methanol. 7.5 mL thionyl chloride was added to the flask at 0 °C under air dropwise, and the solution was stirred for 68 h at 65 °C. The reaction was quenched by slow addition of 20 mL water (CAUTION: exothermic reaction with a formation of HCl gas) at 0 °C followed by slow addition of sat. Na_2CO_3 (CAUTION: exothermic reaction with a formation of CO_2 gas) until the evolution of CO_2 stopped and the solution became basic. The resulting white solid was filtered and washed three times with water and dried in a desiccator. Yield: 1.7297 g, 56%. NMR spectra of **C-NMe2** matched with the reported values[6].

^{1}H NMR (500.13 MHz, $CDCl_3$, 298 K): δ 3.13 (6H, s, N*Me2*), 3.99 (6H, s, 2 COOC*H3*), 7.51 (2H, s, 3,5-C*H* of the pyridine ring).

Preparation of ONO-NMe2: A 250 mL round bottom flask equipped with a Teflon-coated magnetic stirring bar was charged with **C-NMe2** (2.2794 g, 9.57 mmol) and 40 mL 99% ethanol. $NaBH_4$ (1.45 g, 38.3 mmol) was added to the flask at 25 °C under air. The solution was stirred for 11 h at 80 °C. The clear orange-red solution formed after 11 h. The reaction was quenched by the addition of 2 mL water at 0 °C. The solution was concentrated to dryness, and about 30 mL sat. Na_2CO_3 was added to the flask. The flask was equipped with a condenser, and the solution was heated at 100 °C for 2 h. The resulting biphasic mixture was extracted using EtOAc, and the EtOAc solution was dried by $MgSO_4$, and $MgSO_4$ was filtered out. The concentration of EtOAc solution gave a colorless oil of **ONO-NMe2**, which solidified upon keeping it in a -20 °C freezer. Yield: 1.4846 g, 85 %. Supplementary Fig. 3 shows ^{1}H and ^{13}C{^{1}H} NMR spectra of **ONO-NMe2**.
^{1}H NMR (500.13 MHz, $CDCl_3$, 298 K): δ 3.03 (6H, s, N*Me2*), 4.65 (4H, s, 2 *CH2*), 6.37 (2H, s, 3,5-C*H* of the pyridine ring).
^{13}C{^{1}H} NMR (125.76 MHz, $CDCl_3$, 298 K): δ 39.5 (s, N*Me2*), 64.6 (s, *C*H2), 102.0 (s, 3,5-*C*H of the pyridine ring), 158.4 (s, 2,6-*C* of the pyridine ring), 155.8 (s, 4-*C* of the pyridine ring).

Preparation of ClNCl-NMe2: A 250 mL round bottom flask equipped with a Teflon-coated magnetic stirring bar was charged with **ONO-NMe2** (1.4846 g, 8.15 mmol). 10 mL thionyl chloride was added to the flask at 0 °C under air, and the solution was stirred for 22 h at 25 °C. The reaction was quenched by slow addition of 10 mL water (CAUTION: exothermic reaction with a formation of HCl gas) at 0 °C followed by slow addition of sat. Na_2CO_3 (CAUTION: exothermic reaction with a formation of CO_2 gas) until the evolution of CO_2 stopped and the solution became basic. The resulting floating white solid was filtered and washed three times with water and dried in a desiccator. Yield: 1.6303 g, 91%. Supplementary Fig. 4 shows ^{1}H and ^{13}C{^{1}H} NMR spectra of **ClNCl-NMe2**.
^{1}H NMR (500.13 MHz, $CDCl_3$, 298 K): δ 3.05 (6H, s, N*Me2*), 4.56 (4H, s, 2 *CH2*), 6.61 (2H, s, 3,5-C*H* of the pyridine ring).
^{13}C{^{1}H} NMR (125.76 MHz, $CDCl_3$, 298 K): δ 39.5 (s, N*Me2*), 47.4 (br s, *C*H2), 104.8 (s, 3,5-*C*H of the pyridine ring), 156.1 (s, 4-*C* of the pyridine ring), 156.7 (s, 2,6-*C* of the pyridine ring).
HRMS (ESI/Orbitrap, $[M + H]^+$): Calcd for $C_9H_{13}N_2Cl_2$: 219.0450. Found: 219.0447.

Preparation of NMe2-CpNCpNa2: In a nitrogen glovebox, a 20 mL vial equipped with a Teflon-coated stirring bar was charged with 1.5M NaCp (1.4 mL in MeCN, 2.1 mmol) and 2 mL MeCN. **ClNCl-NMe2** (109.6 mg, 0.500 mmol) in 2 mL MeCN was added to the solution dropwise for 5 min, and the vial containing **ClNCl-NMe2** was washed by 1 mL MeCN and the MeCN solution was added to the reaction mixture. The solution was stirred at 25 °C for 30 min. The reaction mixture was passed through a plug of Celite using MeCN and the resulting colorless solution was concentrated to give a white solid and colorless oil. The addition of 6 mL ether to the oil formed a pink solid. The solid was decanted and washed once with 6 mL ether and dried. Yield: 149.8 mg, 85 % (contains 0.5 equiv. of ether). Supplementary Fig. 5 shows ^{1}H and ^{13}C{^{1}H} NMR spectra of **NMe2-CpNCpNa2**.
^{1}H NMR (500.13 MHz, THF-d_8, 298 K): δ 2.96 (6H, s, N*Me2*), 3.79 (4H, s, 2 *CH2*), 5.32 (4H, pseudo t, 2,5-position of C_5H_4 groups), 5.48 (4H, pseudo t, 3,4-position of C_5H_4 groups), 6.24 (2H, s, 3,5-C*H* of the pyridine ring).
^{13}C{^{1}H} NMR (125.76 MHz, THF-d_8, 298 K): δ 39.3 (s, N*Me2*), 40.3 (s, *C*H2), 102.98 (s, 3,4-position of C_5H_4 groups), 103.0 (s, 3,5-*C*H of the pyridine ring), 104.3 (s, 2,5-position of C_5H_4

groups), 116.3 (s, 1-position of C_5H_4 groups), 156.0 (s, 4-*C*H of the pyridine ring), 166.0 (s, 2,6-*C*H of the pyridine ring).
^{15}N NMR (50.68 MHz, 4:1 toluene-d_8:THF-d_8, 233 K, detected using ^{1}H-^{15}N HMBC): δ 51.7 (s, *N*Me$_2$), 261.5 (s, pyridyl *N*).
HRMS (ESI/Orbitrap, [M + 3H]$^+$): Calcd for $C_{19}H_{23}N_2$: 279.1856. Found: 279.1825.

Supplementary Note 2. Preparation and reactivity of complexes

Preparation of [Fe(H-CpNCp)] (**1-H**)

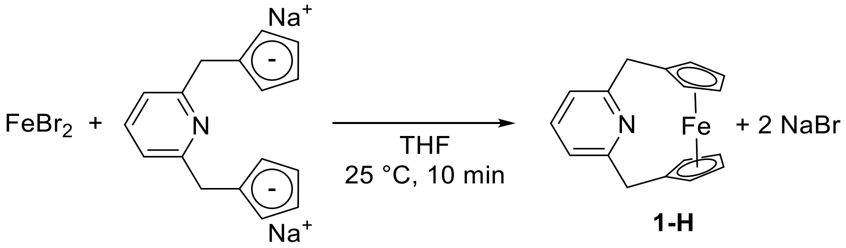

1-H

In a nitrogen glovebox, a 20 mL vial equipped with a Teflon-coated stirring bar was charged with $FeBr_2$ (220.6 mg, 1.05 mmol) and 10 mL THF. The solution was heated intermittently with a heat gun until a clear orange solution was obtained. A solid of H-CpNCpNa$_2$ (281.3 mg, 1.01 mmol) was added portion wise to the solution for about 10 min at 25 °C. We added **H-CpNCpNa$_2$** as a solid due to the low solubility of **H-CpNCpNa$_2$** in THF. As soon as the addition of **H-CpNCpNa$_2$** was completed, 4 mL *n*-pentane was added to the solution, and the resulting precipitate was removed by passing through a plug of Celite, and Celite was washed three times with ether, and the ether solution was combined to obtain dark red solution. The solution was concentrated to dryness, and the solid was extracted using ether and the ether solution was passed through a plug of Celite. The concentration of the ether solution gave dark red-brown crystals of **1-H**. Yield: 158.2 mg, 54%.

Supplementary Figs. 6-11 and 31 show ^{1}H, ^{13}C{^{1}H}, and ^{1}H-^{15}N HMBC NMR, FTIR, VSM, and UV-Vis data of **1-H**.

^{1}H NMR (500.13 MHz, toluene-d_8, 233 K): δ 2.88 (4H, s, 2 C*H$_2$*), 3.93 (4H, br s, 3,4-position of *C$_5$H$_4$* groups), 4.14 (4H, br s, 2,5-position of *C$_5$H$_4$* groups), 6.48 (2H, d, $^3J_{HH,}$ = 7.5 Hz, 3,5-position of pyridine ring), 6.86 (1H, d, $^3J_{HH,}$ = 7.5 Hz, 4-position of pyridine ring).

^{1}H NMR (500.13 MHz, toluene-d_8, 423 K under 5 bar N_2): δ -24.9 (4H, $\Delta\nu^{1/2}$ = 2871 Hz, a signal from *C$_5$H$_4$* groups), -10.1 (4H, $\Delta\nu^{1/2}$ = 828 Hz, a signal from *C$_5$H$_4$* groups), 3.00 (1H, $\Delta\nu^{1/2}$ = 72.5 Hz, a signal from 4-position of pyridine ring), 14.6 (2H, $\Delta\nu^{1/2}$ = 244 Hz, a signal from 3,5-position of pyridine ring), 18.7 (4H, $\Delta\nu^{1/2}$ = 887 Hz, 2 *CH$_2$*).

^{13}C{^{1}H} NMR (125.76 MHz, toluene-d_8, 233 K): δ 33.6 (s, 2 *C*H$_2$), 63.7 (s, 3,4-position of *C$_5$H$_4$* groups), 71.3 (s, 2,5-position of *C$_5$H$_4$* groups), 106.8 (s, 1-position of *C$_5$H$_4$* groups), 117.7 (s, 3,5-position of pyridine ring),133.8 (s, 4-position of pyridine ring), 165.8 (s, 2,6-position of pyridine ring).

^{15}N NMR (50.68 MHz, toluene-d_8, 233 K, detected using ^{1}H-^{15}N HMBC): δ 337.7 (s).

Effective magnetic moment: μ_{eff} (Evans' method, toluene-d_8, without considering thermal change of solvent density) = 0.69 (233 K), 0.86 (298 K), 1.8 (403 K) μ_B. μ_{eff} (VSM, bulk solid, 298.13 K) = 0 μ_B.

FTIR (Thin film, cm^{-1}): 1576 (w, C=C, and C=N stretch), 1453 (m, sp^3 C-H bending), 1032 (m, sp^2 C-H bending), 1015 (m, sp^2 C-H bending), 807 (s, sp^2 C-H bending), 758 (s, sp^2 C-H bending).

UV-Vis (THF, 25 °C): λ_{max} = 258 nm (ε = 6600 M^{-1} cm^{-1}), 383 nm (ε = 900 M^{-1} cm^{-1}).

EPR (9.079 GHz, 2.5 mM toluene glass, 4.2 K): No detectable signal from the sample.

XPS (298 K): 707.6 eV (Fe 2$p^{3/2}$), 720.4 eV (Fe 2$p^{1/2}$).

Elemental analysis: Calcd: C:70.61, H:5.23, N:4.84. Found: C:70.63, H:5.01, N:4.75.

HRMS (ESI/TOF, [M]$^+$): Calcd for $C_{17}H_{15}N_1Fe_1$: 289.0548. Found: 289.0542.

Preparation of [Fe(H-CpNCp-d_8)] (**1-H-*d*₈**)

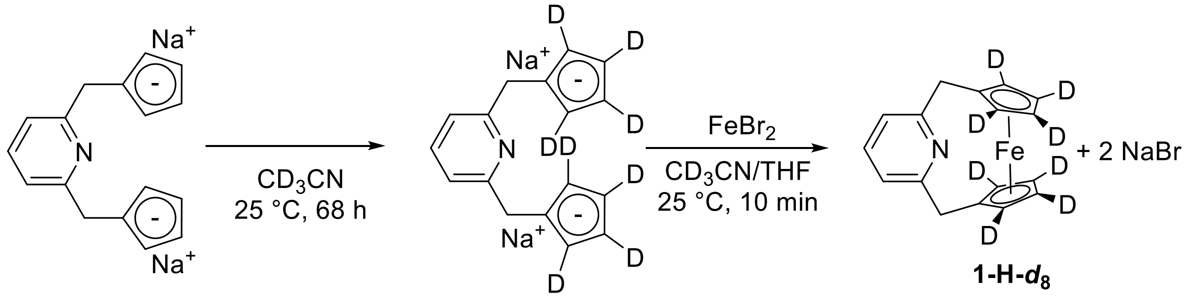

In a nitrogen glovebox, a J. Young NMR tube was charged with **H-CpNCpNa₂** (5.8 mg, 0.021 mmol) and 0.50 mL CD_3CN. The solution was left standing for 68 h to achieve >98% deuteration of the cyclopentadieneyl protons. The CD_3CN solution was then added quantitatively using 1 mL THF to a solution of $FeBr_2$ (7.5 mg, 0.035 mmol) in 2 mL THF. The solution was stirred for 10 min, and the solvent was removed under vacuum. The resulting brown solid was extracted using ether, and the ether solution was passed through a plug of Celite. The concentration of the ether solution gave dark brown crystals of **1-H-*d*₈**. Yield: 3.1 mg, 51%. Supplementary Fig. 12 shows ^{1}H NMR spectra of **1-H-*d*₈**.
^{1}H NMR (500.13 MHz, toluene-d_8, 233 K): δ 2.88 (4H, s, 2 CH_2), 3.92 (4H, s, 2% residual signal from 3,4-position of C_5H_4 groups), 4.13 (4H, s, 2% residual signal from 2,5-position of C_5H_4 groups), 6.48 (2H, d, $^3J_{HH,}$ = 7.5 Hz, 3,5-position of pyridine ring), 6.86 (1H, d, $^3J_{HH,}$ = 7.5 Hz, 4-position of pyridine ring).
^{1}H NMR (500.13 MHz, toluene-d_8, 423 K under 5 bar N_2): δ 3.33 (1H, $\Delta\nu^{1/2}$ not available due to overlapping with a signal from diethyl ether, a signal from 4-position of pyridine ring), 13.9 (2H, $\Delta\nu^{1/2}$ = 238 Hz, a signal from 3,5-position of pyridine ring), 17.5 (4H, $\Delta\nu^{1/2}$ = 755 Hz, 2 CH_2).
HRMS (ESI/TOF, $[M]^+$): Calcd for $C_{17}H_7D_8N_1Fe_1$: 297.1051. Found: 297.1062.

Preparation of [Fe(Cl-CpNCp)] (**1-Cl**)

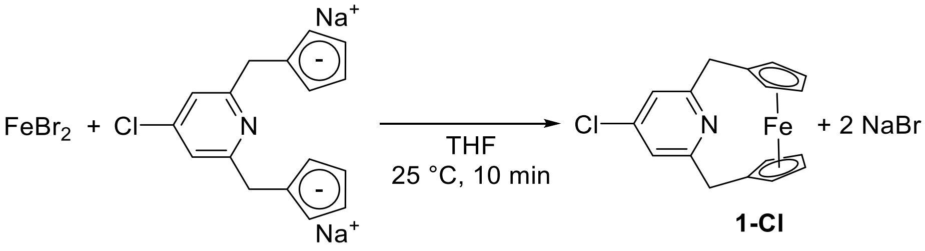

In a nitrogen glovebox, a 20 mL vial equipped with a Teflon-coated stirring bar was charged with $FeBr_2$ (75.2 mg, 0.349 mmol) and 8 mL THF. The solution was heated intermittently with a heat gun until a clear orange solution was obtained. To the solution at 25 °C was then added **Cl-CpNCpNa₂** (106.8 mg, 0.340 mmol) in 2 mL THF dropwise for about 5 min. The vial containing **Cl-CpNCpNa₂** was washed twice with 1 mL each of THF and the THF solution was added to the solution of $FeBr_2$. As soon as the addition of **Cl-CpNCpNa₂** was completed, the dark brown suspension was concentrated to dryness, and the resulting solid was extracted with benzene. The benzene extract was passed through a plug of Celite, and the Celite was washed three times with benzene. The concentration of combined benzene solution gave dark brown needles of **1-Cl**. The crystals were washed three times with *n*-pentane and dried. Yield: 59.7 mg, 54%.
Supplementary Figs. 13-16 and 31 show ^{1}H, ^{13}C{^{1}H}, and ^{1}H-^{15}N HMBC NMR, FTIR, VSM, and UV-Vis data of **1-Cl**.

^{1}H NMR (500.13 MHz, toluene-d_8, 233 K): δ 2.64 (4H, s, 2 C*H₂*), 3.89 (4H, br t, 3,4-position of C_5H_4 groups), 4.05 (4H, br t, 2,5-position of C_5H_4 groups), 6.38 (2H, s, 3,5-position of pyridine ring).
^{13}C{^{1}H} NMR (125.76 MHz, toluene-d_8, 233 K): δ 33.2 (s, 2 *C*H_2), 63.8 (s, 3,4-position of C_5H_4 groups), 71.2 (s, 2,5-position of C_5H_4 groups), 106.5 (s, 1-position of C_5H_4 groups), 118.1 (s, 3,5-position of pyridine ring),140.9 (s, 4-position of pyridine ring), 167.5 (s, 2,6-position of pyridine ring).
^{15}N NMR (50.68 MHz, toluene-d_8, 233 K, detected using ^{1}H-^{15}N HMBC): δ 333.6 (s).
Effective magnetic moment: μ_{eff} (VSM, bulk solid, 298.17 K) = 0 μ_B.
FTIR (Thin film, cm^{-1}): 1566 (m, C=C, and C=N stretch), 1408 (w, sp^3 C-H bending), 1021 (m, sp^2 C-H bending), 1015 (m, sp^2 C-H bending), 845 (s, sp^2 C-H bending), 808 (s, sp^2 C-H bending).
UV-Vis (THF, 25 °C): λ_{max} = 264 nm (ε = 7200 M^{-1} cm^{-1}), 407 nm (ε = 1100 M^{-1} cm^{-1}).
EPR (9.079 GHz, 2.5 mM toluene glass, 4.2 K): No detectable signal from the sample.
Elemental analysis: Calcd: C:63.10, H:4.36, N:4.33. Found: C:63.49, H:4.23, N:4.45.
HRMS (ESI/TOF, $[M]^+$): Calcd for $C_{17}H_{14}N_1Cl_1Fe_1$: 323.0159. Found: 323.0161.

Preparation of [Fe(CpNCp-OMe)] (**1-OMe** + **2-OMe**)

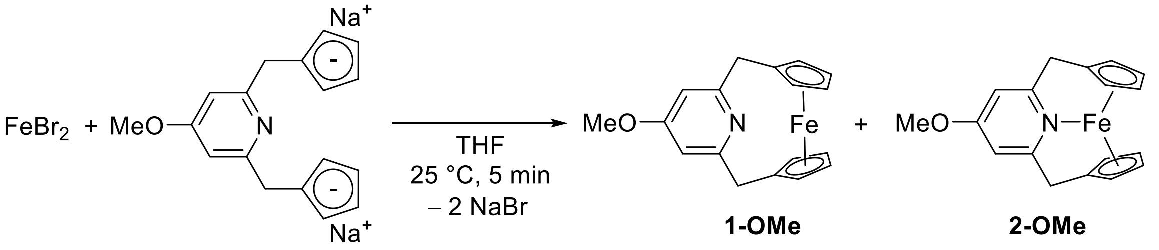

In a nitrogen glovebox, a 20 mL vial equipped with a Teflon-coated stirring bar was charged with $FeBr_2$ (215.1 mg, 0.997 mmol) and 12 mL THF. The solution was heated intermittently with a heat gun until a clear orange solution was obtained. To the solution at 25 °C was then added **MeO-CpNCpNa₂** (283.5 mg, 0.917 mmol) in 2 mL THF dropwise for about 5 min. The vial containing **MeO-CpNCpNa₂** was washed twice with 0.5 mL each of THF and the THF solution was added to the solution of $FeBr_2$. As soon as the addition of **MeO-CpNCpNa₂** was completed, the dark purple suspension was concentrated to dryness, and the resulting solid was extracted with benzene. The benzene extract was passed through a plug of Celite, and the Celite was washed three times with benzene. The concentration of the combined benzene solution gave a dark brown solid. The solid was extracted with 4:1 *n*-pentane: benzene solution, and the solution was passed through a plug of Celite, and the Celite wash washed with *n*-pentane. The concentration of the combined *n*-pentane-benzene solution gave a dark brown solid of a mixture of **1-OMe** and **2-OMe**. Yield: 87.0 mg, 30%.
Supplementary Figs. 17-21 and 31 show ^{1}H, ^{13}C{^{1}H}, and ^{1}H-^{15}N HMBC NMR, FTIR, VSM, and UV-Vis data of **1-OMe** and **2-OMe**.
^{1}H NMR (500.13 MHz, toluene-d_8, 233 K, signals from **1-OMe**): δ 2.85 (4H, s, 2 C*H₂*), 3.17 (3H, s, OC*H₃*), 3.93 (4H, br s, 3,4-position of C_5H_4 groups), 4.24 (4H, br s, 2,5-position of C_5H_4 groups), 6.27 (2H, s, 3,5-position of pyridine ring).
^{1}H NMR (500.13 MHz, toluene-d_8, 233 K, signals from **2-OMe**): δ -344.0 ($\Delta\nu^{1/2}$ = 2816 Hz), -195.0 ($\Delta\nu^{1/2}$ = 2971 Hz), 109.0 ($\Delta\nu^{1/2}$ = 157 Hz), 189.9 ($\Delta\nu^{1/2}$ = 101), one signal is missing likely due to overlapping with diamagnetic signals.
^{13}C{^{1}H} NMR (125.76 MHz, toluene-d_8, 233 K): δ 33.7 (s, 2 *C*H_2), 54.4 (s, O*C*H_3), 63.5 (s, 3,4-position of C_5H_4 groups), 71.0 (s, 2,5-position of C_5H_4 groups), 104.1 (*s*, 2 *C*H, 3,5-position

of pyridine ring), 106.8 (s, 1-position of *C_5H_4* groups), 165.2 (s, 4-position of pyridine ring), 167.7 (s, 2,6-position of pyridine ring). Detection of ^{13}C NMR signals from **2-OMe** was not attempted.
^{15}N NMR (50.68 MHz, toluene-*d_8*, 233 K, detected using ^{1}H-^{15}N HMBC, a signal from **1-OMe**): δ 316.0 (s). A signal form **2-OMe** was not detectable.
Effective magnetic moment: μ_{eff} (VSM, bulk solid, 298.15 K) = 4.88 μ_B.
FTIR (Thin film, cm^{-1}): 3056, 2916, 1566, 1408, 1337, 1188, 1148, 1047, 1015, 853, 815, 753.
UV-Vis (THF, 25 °C): λ_{max} = 365 nm (ε = 1100 M^{-1} cm^{-1}).
EPR (9.079 GHz, 2.5 mM toluene glass, 4.2 K): No detectable signal from the sample.
Elemental analysis: Calcd: C:67.73, H:5.37, N:4.39. Found: C:67.97, H:5.25, N:4.46.
HRMS (ESI/TOF, [M]$^+$): Calcd for $C_{18}H_{17}N_1O_1Fe_1$: 319.0654. Found: 319.0655.

Preparation of [Fe(CpNCp-NMe$_2$)] **(2-NMe$_2$)**

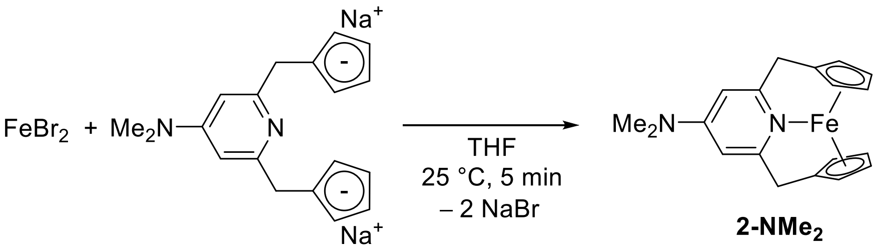

In a nitrogen glovebox, a 20 mL vial equipped with a Teflon-coated stirring bar was charged with $FeBr_2$ (108.3 mg, 0.50 mmol) and 10 mL THF. The solution was heated intermittently with a heat gun until a clear orange solution was obtained. To the solution at 25 °C was then added **NMe$_2$-CpNCpNa$_2$** (146.5 mg, 0.45 mmol) in 2 mL THF dropwise for about 5 min. The vial containing **NMe$_2$-CpNCpNa$_2$** was washed three times with 1 mL each of THF and the THF solution was added to the solution of $FeBr_2$. After the addition, the brown suspension was stirred for 5 min and concentrated to dryness. The resulting brown solid was extracted with benzene. The dark purple benzene extract was passed through a plug of Celite, and the Celite was washed three times with benzene. The concentration of the combined benzene solution gave an ivory/off-white solid of **2-NMe$_2$**. Yield: 59.4 mg, 39%. This solid can be further purified by extraction with pentane or by precipitation from a hot saturated benzene solution to obtain an off-white solid of **2-NMe$_2$**. This solid was used for Mössbauer spectroscopy and VSM measurements.
Supplementary Figs. 22-28 and 32 show ^{1}H, ^{13}C{^{1}H}, ^{1}H-^{1}H EXSY, and ^{1}H-^{13}C HMBC NMR, FTIR, VSM, and UV-Vis data of **1-NMe$_2$** and **2-NMe$_2$**.
^{1}H NMR (500.13 MHz, toluene-*d_8*, 233 K, signals from **1-NMe$_2$**): δ 2.39 (6H, s, N(C*H_3*)$_2$), 2.98 (4H, s, 2 C*H_2*), 3.95 (4H, br s, 3,4-position of *C_5H_4* groups), 4.34 (4H, br s, 2,5-position of *C_5H_4* groups), 6.05 (2H, s, 3,5-position of pyridine ring).
^{1}H NMR (500.13 MHz, toluene-*d_8*, 233 K, signals from **2-NMe$_2$**): δ -330.0 (4H, $\Delta\nu^{1/2}$ = 2560 Hz, 3,4-position of *C_5H_4* groups), -210.9 (4H, $\Delta\nu^{1/2}$ = 2762 Hz, 2,5-position of *C_5H_4* groups), 11.28 (6H, $\Delta\nu^{1/2}$ = 25.8 Hz, N(C*H_3*)$_2$), 108.5 (2H, $\Delta\nu^{1/2}$ = 117 Hz, 3,5-position of pyridine ring), 177.9 (4H, $\Delta\nu^{1/2}$ = 888, 2 C*H_2*). Assigned based on integration values and comparison to ^{1}H NMR spectra of previously reported *S* = 3/2, Co(CpNCp-H) complex.
^{13}C{^{1}H} NMR (125.76 MHz, toluene-*d_8*, 233 K, signals from **1-NMe$_2$**): δ 34.2 (s, 2 *C*H$_2$), 39.4 (s, N(*C*H$_3$)$_2$), 63.2 (s, 3,4-position of *C_5H_4* groups), 70.6 (s, 2,5-position of *C_5H_4* groups), 101.1 (*s*, 2 *C*H, 3,5-position of pyridine ring), 106.7 (s, 1-position of *C_5H_4* groups), 153.9 (s, 4-position of pyridine ring), 166.7 (s, 2,6-position of pyridine ring).

^{13}C{^{1}H} NMR (125.76 MHz, toluene-*d*$_8$, 233 K, signals from **2-NMe$_2$**): δ 23.9 (s, N(*C*H$_3$)$_2$, Confirmed by ^{1}H-^{13}C HSQC), 231.3 (*br*, most likely 3,5-position of pyridine ring. It appears as broad doublet in proton-coupled ^{13}C NMR). Other ^{13}C NMR signals were not detectable.
^{15}N NMR (50.68 MHz, toluene-*d*$_8$, 233 K, detected using ^{1}H-^{15}N HMBC, a signal from **1-NMe$_2$**): δ 298.6 (s). Signals form *N*Me$_2$ groups, and **2-NMe$_2$** were not detectable.
Effective magnetic moment: μ_{eff} (Evans' method, Toluene-*d*$_8$, 233 K) = 4.83 μ_B. μ_{eff} (VSM, 298.14 K) = 4.90 μ_B.
FTIR (KBr pellet, cm^{-1}): 3081, 2901, 1612 (s, C=C and C=N stretch), 1529, 1415, 1384, 1022, 800, 760, 742, 654.
UV-Vis (in THF, 25 °C): λ_{max} = 258 nm (ε = 16300 M^{-1} cm^{-1}), 267 nm (ε = 15600 M^{-1} cm^{-1}).
EPR (9.079 GHz, 2.5 mM toluene glass, 4.2 K): No detectable signal from the sample.
Elemental analysis: Calcd: C:68.69, H:6.07, N:8.43. Found: C:68.59, H:5.86, N:8.26.
HRMS (ESI/Orbitrap, [M]$^+$): Calcd for $C_{19}H_{20}N_2Fe_1$: 332.0970. Found: 332.0966.

Preparation of [Fe(NMe$_2$-CpNCp-*d*$_8$)] (**1-NMe$_2$-*d*$_8$**)

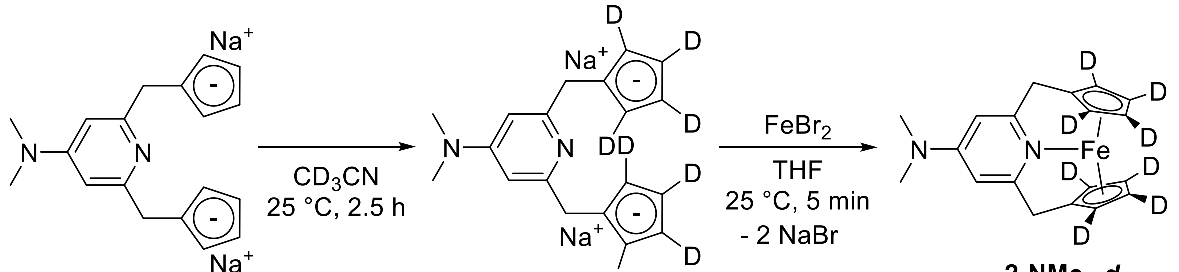

2-NMe$_2$-*d*$_8$

In a nitrogen glovebox, a 6 mL vial was charged with **NMe$_2$-CpNCpNa$_2$** (15.9 mg, 0.049 mmol) and 0.50 mL CD$_3$CN. The solution was left standing for 2.5 h to achieve >95% deuteration of the cyclopentadienyl protons. Fig. 29 shows ^{1}H NMR spectra of **NMe$_2$-CpNCpNa$_2$-*d*$_8$**. CD$_3$CN was removed under vacuum, and the resulting pink solid was dissolved in 2 mL THF. The solution was then added dropwise to a clear yellow solution of FeBr$_2$ (10.9 mg, 0.051 mmol) in 4 mL THF. The vial containing **NMe$_2$-CpNCpNa$_2$-*d*$_8$** was washed two times with 0.5 mL each of THF, and the THF solution was added to the solution of FeBr$_2$. The solution was stirred for 5 min, and the solvent was removed under vacuum. The resulting brown solid was extracted using benzene and the dark purple benzene solution was passed through a plug of Celite. The concentration of the benzene solution gave an off-white solid with yellow impurity. This solid was extracted using ~1:10 toluene/pentane mixture. The concentration of this solution gave an off-white solid of **2-NMe$_2$-*d*$_8$**. Yield: 7.0 mg, 42%.
Supplementary Fig. 30 shows ^{1}H NMR spectra of **1-NMe$_2$-*d*$_8$** and **2-NMe$_2$-*d*$_8$**.
^{1}H NMR (400.15 MHz, CD$_3$CN, 298 K, **NMe$_2$-CpNCpNa$_2$-*d*$_8$**): δ 2.99 (6H, s, N(C*H$_3$*)$_2$), 3.76 (4H, s, 2 C*H$_2$*), 5.23 (4H, br s, 4% residual signal from 3,4-position of *C$_5$H$_4$* groups), 5.42 (4H, br s, 4% residual signal from 2,5-position of *C$_5$H$_4$* groups), 6.30 (2H, s, 3,5-position of pyridine ring).
^{1}H NMR (500.13 MHz, toluene-*d*$_8$, 233 K, signals from **1-NMe$_2$-*d*$_8$**): δ 2.35 (6H, s, N(C*H$_3$*)$_2$), 2.98 (4H, s, 2 C*H$_2$*), 6.02 (2H, s, 3,5-position of pyridine ring).
^{1}H NMR (500.13 MHz, toluene-*d*$_8$, 233 K, signals from **2-NMe$_2$-*d*$_8$**): δ -329.4 (4H, $\Delta\nu^{1/2}$ = 2560 Hz, 3,4-position of *C$_5$H$_4$* groups), -210.8 (4H, $\Delta\nu^{1/2}$ = 2762 Hz, 2,5-position of *C$_5$H$_4$* groups), 11.32 (6H, $\Delta\nu^{1/2}$ = 25.8 Hz, N(C*H$_3$*)$_2$), 108.5 (2H, $\Delta\nu^{1/2}$ = 117 Hz, 3,5-position of pyridine ring), 177.8 (4H, $\Delta\nu^{1/2}$ = 888, 2 C*H$_2$*). Assigned based on integration values and comparison to 1H NMR spectra of previously reported *S* = 3/2, Co(CpNCp-H) complex.
HRMS (ESI/TOF, [M]$^+$): Calcd for $C_{17}H_7D_8N_1Fe_1$: 297.1051. Found: 297.1062.

Estimation of thermodynamic parameters for the equilibrium between **1-NMe2** and **2-NMe2**.

In a nitrogen glovebox, a J. Young NMR tube was charged with a solution of **1-NMe2** and **2-NMe2** prepared using 3.5 mg **2-NMe2** and 0.60 mL toluene-d_8. NMR spectra were recorded at 250.2, 245.2, 240.2, 235.2, and 230.2 ± 0.1 K (temperatures calibrated using CD_3OD[7]). Integral ratios between the NMe_2 group of **2-NMe2** and the NMe_2 group of **1-NM2** or the CH_2 group of **1-NM2** were used to calculate the equilibrium ratio of **2-NM2** and **1-NM2**. The ratios of **2-NM2**:**1-NM2** were 78:22 (in CD_3CN), 66:34 (in THF-d_8), and 44:56 (in toluene-d_8) at 230.2 K.

Supplementary Figs. 33-34 shows ^{1}H NMR spectra and van't Hoff plot used to estimate the thermodynamic parameters and ratios of **2-NM2** and **1-NM2**.

Reaction between ferrocene and 4-dimethylaminopyridine (DMAP).

In a nitrogen glovebox, a Wilmad medium wall quick pressure valve NMR tube (524-QPV-7) was charged with ferrocene (5.8 mg, 0.031 mmol), DMAP (3.8 mg, 0031 mmol) and 0.30 mL toluene-d_8. The tube was pressurized with 3 bar N_2, and NMR spectra were recorded at 298, 373, and 403 K (temperatures not calibrated). No significant sifting of NMR signals due to the formation of DMAP-coordinated species was observed.

Cyclic voltammetry (CV) of **1-X** and **2-X**

CV was measured inside a nitrogen glovebox using an ECstat-301WL potentiostat equipped with a glassy carbon working electrode (3.0 mm diameter), coiled platinum wire (0.5 mm diameter) counter electrode, and Ag/Ag^+ reference electrode (Ag wire in 0.01 M $AgNO_3$ in 0.1 M $[Bu_4N]PF_6$ MeCN solution). The sample solution was prepared by dissolving an appropriate sample in 0.2 M $[Bu_4]NPF_6$ THF or 0.1 M $[Bu_4]NPF_6$ CH_2Cl_2 solution. All potentials are reported using $FeCp_2^{0/+}$ couple (0 mV) as an external reference. The voltammograms were recorded starting from the open circuit potentials of each solution. Cyclic voltammograms of **1-X** and **2-X** in 0.2 M $[Bu_4]NPF_6$ THF are shown in Supplementary Figs. 35-36. Cyclic voltammogram of **1-NMe2** and **2- NMe2** in 0.1 M $[Bu_4]NPF_6$ CH_2Cl_2 is shown in Supplementary Fig. 37.

Preparation of $[FeCp_2]PF_6$

In a nitrogen glovebox, a 20 mL vial equipped with a Teflon-coated stirring bar was charged with $FeCp_2$ (189.8 mg, 1.02 mmol) and 2 mL dichloromethane. In a dark room, $AgPF_6$ (252.6 mg, 1.00 mmol) in dichloromethane 2 mL was added dropwise to the solution of $FeCp_2$ while stirring. The vial containing $AgPF_6$ was washed twice with 1 mL each of dichloromethane, and the wash was added to the $FeCp_2$ solution. The solution was stirred for 15 min, and was passed through a plug of Celite using MeCN. The resulting solution was concentrated to dryness. The solid was dissolved in about 2 mL MeCN, and 15 mL ether was layered on top of the MeCN solution. The slow diffusion of ether gave blue crystals of the product. The crystals were decanted, washed three times with ether, and dried to obtain dark blue crystals of the product. Yield: 279.1 mg, 84%.

Preparation of **3-H**

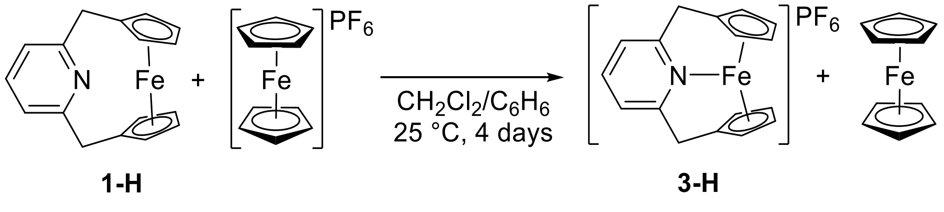

Method A: In a nitrogen glovebox, a 20 mL vial equipped with a Teflon-coated stirring bar was charged with $[FeCp_2]PF_6$ (34.0 mg, 0.103 mmol) and 10 mL dichloromethane. The suspension was stirred while heated intermittently by a heat gun until all $[FeCp_2]PF_6$ dissolved. The stirring bar was removed from the solution, and a solution of **1-H** (29.0 mg, 0.100 mmol) in 10 mL benzene was layered on top of the dichloromethane layer. The bilayer solution was left standing at 25 °C for four days, resulting in the formation of dark brown needles of product and yellow solution of ferrocene. The crystals were decanted, washed three times with benzene and dried to obtain dark green crystals of the product. Yield: 36.5 mg, 84%.

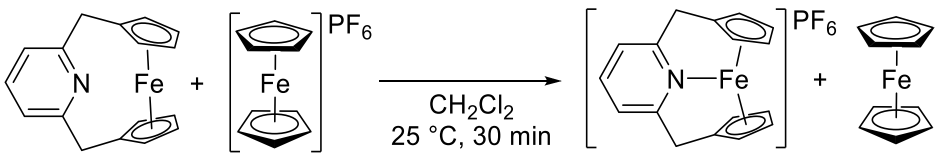

1-H **3-H**

Method B: In a nitrogen glovebox, a 20 mL vial equipped with a Teflon-coated stirring bar was charged with $[FeCp_2]PF_6$ (34.1 mg, 0.103 mmol) and 14 mL dichloromethane. The suspension was stirred while heated intermittently by a heat gun until all $[FeCp_2]PF_6$ dissolved. The stirring bar was removed from the solution, and a solid of **1-H** (29.0 mg, 0.100 mmol) was added to the vial. The solution was stirrer for 30 min at 25 °C. The resulting dark green microcrystals were decanted, washed three times with dichloromethane, and dried to obtain the dark green powder of the product. Yield: 35.4 mg, 81%.
Supplementary Fig. 38 shows ^{1}H NMR and EPR spectra of **3-H**.
^{1}H NMR (400.15 MHz, CD_3CN, 298 K): δ 9.89 (br, $\Delta v^{1/2}$ = 40.0 Hz), 26.5(br, $\Delta v^{1/2}$ = 2204 Hz).
^{31}P{^{1}H} NMR (202.45 MHz, CD_3CN, 298 K): δ -144.6 (septet, $^1J_{PF,}$ = 706 Hz,),
^{19}F NMR (470.54 MHz, CD_3CN, 298 K): δ 72.88 (d, $^1J_{FP,}$ = 706 Hz,),
Effective magnetic moment: μ_{eff} (Evans' method, CD_3CN, 298 K) = 2.0 μ_B.
EPR (9.071 GHz, 1.6 mM 1:1 toluene: acetone glass, 77 K): g1, g2, g3 = 2.163, 2.007, 1.978. A1, A2, A3 (^{14}N) = 10.8, 48.9, 38.3 MHz. A1, A2, A3 (^1H_a) = 51.2, 0, 0 MHz. A1, A2, A3, (^1H_b) = 19.3, 0, 0 MHz.
XPS (298 K): 708.4 eV (Fe $2p^{3/2}$), 721.2 eV (Fe $2p^{1/2}$).
Elemental analysis: Calcd: C:47.03, H:3.48, N:3.23. Found: C:46.74, H:3.33, N:3.23.
HRMS (ESI/TOF, $[M-PF_6]^+$): Calcd for $C_{17}H_{15}N_1Fe_1$: 289.0548. Found: 289.0548.

Preparation of **3-Cl**

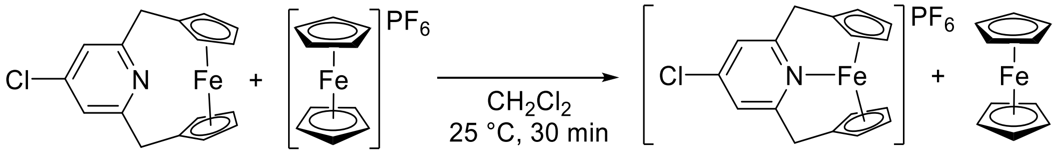

1-Cl **3-Cl**

In a nitrogen glovebox, a 20 mL vial equipped with a Teflon-coated stirring bar was charged with $[FeCp_2]PF_6$ (33.0 mg, 0.100 mmol) and **1-Cl** (33.2 mg, 0.103 mmol). To the vial was added 4 mL dichloromethane, and the solution was stirred for 30 min at 25 °C. 2 mL MeCN was added to the resulting dark green solution with insoluble black precipitate. The solution was passed through a plug of Celite using MeCN. The resulting solution was concentrated to about 0.5 mL. The resulting dark green solid was partially dissolved by the addition of 3 mL dichloromethane. 10 mL of benzene was layered on top of the dichloromethane layer. The

bilayer solution was left standing at 25 °C for one day, resulting in the formation of a green solid product and a yellow solution of ferrocene. The solid was decanted, washed three times with benzene, and dried to obtain the green solid of the product. Yield: 42.1 mg, 90%.
Supplementary Fig. 39 shows ^{1}H NMR and EPR spectra of **3-Cl**.
^{1}H NMR (400.15 MHz, CD_3CN, 298 K): δ 27.0 (br, $\Delta\nu^{1/2}$ = 2100 Hz).
^{31}P{^{1}H} NMR (202.45 MHz, CD_3CN, 298 K): δ -144.6 (septet, $^{1}J_{PF,}$ = 706 Hz,),
^{19}F NMR (470.54 MHz, CD_3CN, 298 K): δ 72.88 (d, $^{1}J_{FP,}$ = 706 Hz,),
Effective magnetic moment: μ_{eff} (Evans' method, CD_3CN, 298 K) = 2.2 μ_B.
EPR (9.079 GHz, 1.6 mM 1:1 toluene:acetone glass, 77 K): g1, g2, g3 = 2.168, 2.007, 1.979 MHz. A1, A2, A3 (^{14}N) = 0.9, 48.6, 38.4 MHz. A1, A2, A3 ($^{1}H_a$) = 48.8, 0, 0 MHz. A1, A2, A3, ($^{1}H_b$) = 12.7, 0, 0 MHz.
Elemental analysis: Calcd: C:43.58, H:3.01 N:2.99. Found: C:43.37, H:2.80, N:3.31.
HRMS (ESI/TOF, $[M{-}PF_6]^+$): Calcd for $C_{17}H_{14}N_1Cl_1Fe_1$: 323.0159. Found: 323.0150.

Preparation of **3-OMe**

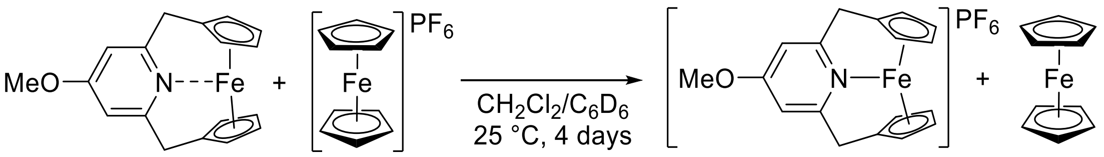

1-OMe + 2-OMe **3-OMe**

In a nitrogen glovebox, a 20 mL vial equipped with a Teflon-coated stirring bar was charged with $[FeCp_2]PF_6$ (22.1 mg, 0.067 mmol) and 10 mL dichloromethane. The suspension was stirred while heated intermittently by a heat gun until all $[FeCp_2]PF_6$ dissolved. The stirring bar was removed from the solution, and a solution of **1-OMe** + **2-OMe** (21.2 mg, 0.066 mmol) in 10 mL benzene was layered on top of the dichloromethane layer. The bilayer solution was left standing at 25 °C for four days, resulting in the formation of dark brown crystals of product and yellow solution of ferrocene. The crystals were decanted, washed three times with benzene, and dried to obtain brown-green crystals of the product. Yield: 29.1 mg, 94%.
Supplementary Fig. 40 shows ^{1}H NMR and EPR spectra of **3-OMe**.
^{1}H NMR (400.15 MHz, CD_3CN, 298 K): δ 4.19 (br, $\Delta\nu^{1/2}$ = 12.6 Hz), 26.0 (br, $\Delta\nu^{1/2}$ = 2591 Hz).
^{31}P{^{1}H} NMR (202.45 MHz, CD_3CN, 298 K): δ -144.6 (septet, $^{1}J_{PF,}$ = 706 Hz,),
^{19}F NMR (470.54 MHz, CD_3CN, 298 K): δ 72.88 (d, $^{1}J_{FP,}$ = 706 Hz,),
Effective magnetic moment: μ_{eff} (Evans' method, CD_3CN, 298 K) = 2.5 μ_B.
EPR (9.078 GHz, 1.6 mM 1:1 toluene:acetone glass, 77 K): g1, g2, g3 = 2.158, 2.007, 1.976 MHz. A1, A2, A3 (^{14}N) = 8.9, 50.9, 40.1 MHz. A1, A2, A3 ($^{1}H_a$) = 57.9, 0, 0 MHz. A1, A2, A3, ($^{1}H_b$) = 20.4, 0, 0 MHz.
Elemental analysis: Calcd for **3-OMe** + 0.2 CH_2Cl_2: C:45.43, H:3.65, N:2.91. Found: C:45.50, H:3.60, N:2.81.
HRMS (ESI/TOF, $[M{-}PF_6]^+$): Calcd for $C_{18}H_{17}N_1O_1Fe_1$: 319.0654. Found: 319.0654.

Preparation of **3-NMe$_2$**

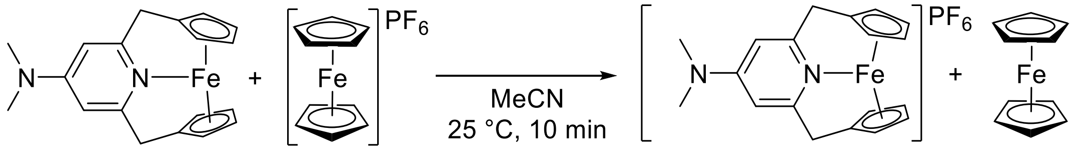

2-NMe$_2$ **3-NMe$_2$**

In a nitrogen glovebox, a 6 mL vial equipped was charged with $[FeCp_2]PF_6$ (6.8 mg, 0.021 mmol) and **2-NMe2** (6.6 mg, 0.020 mmol). To the vial, 2 mL MeCN was added, and all the solids were dissolved. The dark brown solution was left at 25 C for 10 min, layered with benzene, and left for 4 hours. The resulting precipitate was washed by benzene and dissolved in 1 mL MeCN. The brown MeCN solution was filtered through a plug of Celite using 1 mL MeCN. The combined MeCN solution was concentrated to dryness. The brown solid was washed three times with 2 mL each of pentane and dried. Yield: 9.3 mg, 98%. Single crystals suitable for the SC-XRD analysis were obtained upon the concentration of the MeCN solution to near dryness.

Supplementary Fig. 41 shows the ^{1}H NMR and EPR spectrum of **3-NMe2**.

^{1}H NMR (400.15 MHz, CD_3CN, 298 K): δ 1.64 (br, $\Delta\nu^{1/2}$ = 19.8 Hz), 2.26 (br, $\Delta\nu^{1/2}$ = 50.3 Hz), 25.0 (br, $\Delta\nu^{1/2}$ = 4056 Hz).

^{31}P{^{1}H} NMR (202.45 MHz, CD_3CN, 298 K): δ -144.6 (septet, $^1J_{PF,}$ = 706 Hz,),

^{19}F NMR (470.54 MHz, CD_3CN, 298 K): δ 72.88 (d, $^1J_{FP,}$ = 706 Hz,),

Effective magnetic moment: μ_{eff} (Evans' method, CD_3CN, 298 K) = 1.9 μ_B.

EPR (9.077 GHz, 1.6 mM 1:1 toluene:acetone glass, 77 K): g1, g2, g3 = 2.148, 2.006, 1.971 MHz. A1, A2, A3 (^{14}N) = 9.9, 53.6, 42.4 MHz. A1, A2, A3 (^1H_a) = 59.4, 0, 0 MHz. A1, A2, A3, (^1H_b) = 11.2, 0, 0 MHz.

Elemental analysis: Calcd for **3-NMe2**: C:47.82, H:4.22, N:5.87. Found: C:47.84, H:4.15, N:5.98.

HRMS (ESI/TOF, $[M-PF_6]^+$): Calcd for $C_{19}H_{20}N_2Fe_1$: 332.0971. Found: 332.0969.

<u>Oxidation of **3-NMe2** with 1 equiv. $[FeCp_2]PF_6$ in CH_2Cl_2/benzene</u>

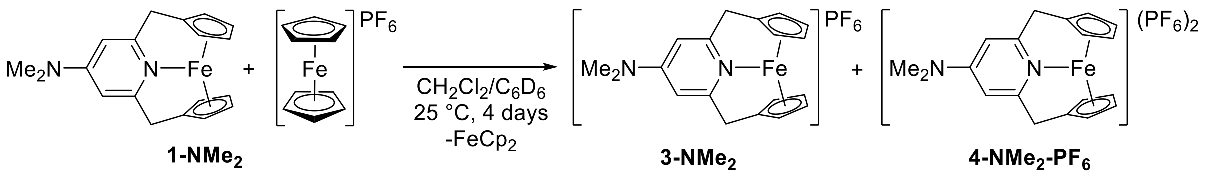

1-NMe2 **3-NMe2** **4-NMe2-PF6**

In a nitrogen glovebox, a 20 mL vial equipped with a Teflon-coated stirring bar was charged with $[FeCp_2]PF_6$ (33.3 mg, 0.101 mmol) and 10 mL dichloromethane. The suspension was stirred while heated intermittently by a heat gun until all $[FeCp_2]PF_6$ dissolved. The stirring bar was removed from the solution, and a solution of **2-NMe2** (33.3 mg, 0.100 mmol) in 10 mL benzene was layered on top of the dichloromethane layer. The bilayer solution was left standing at 25 °C for four days, resulting in the formation of dark brown crystals, a solid of product, and a yellow solution of ferrocene. The crystals and solid were washed three times with benzene and dried to obtain the product. Yield: 25.2 mg. EPR spectrum showed the formation of **3-NMe2**, while SC-XRD analysis of the dark brown crystal showed the formation of **4-NMe2-PF6**. No further purification and isolation of **4-NMe2-PF6** was attempted.

Supplementary Fig. 69 shows the SC-XRD structure of **4-NMe2-PF6**.

Elemental analysis: Calcd for 1/0.3 mixture of **3-NMe2/4-NMe2-PF6**: C:44.69, H:3.95, N:5.49. Found: C:45.01, H:3.90, N:5.09.

<u>Preparation of **4-H**</u>

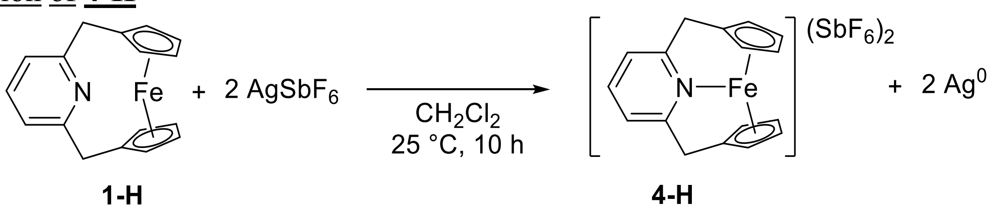

1-H **4-H**

In a nitrogen glovebox, a 20 mL vial equipped with a Teflon-coated stirring bar was charged with **1-H** (28.9 mg, 0.100 mmol) and 4 mL dichloromethane. To the stirring brown solution was added $AgSbF_6$ (84.6 mg, 0.246 mmol) using 2 mL dichloromethane at 25 °C. The solution was stirred for 10 h at 25 °C in a dark room, and a colorless solution with black precipitate was formed. To the vial was added 2 mL nitromethane to extract the insoluble product. The violet solution was passed through a plug of Celite to remove the black solid of Ag^0, and the Celite was washed three times with a 1:1 nitromethane: dichloromethane mixture. The dark violet solution was concentrated and formed a black solid of **4-H**. The solid was washed three times with dichloromethane to remove excess $AgSbF_6$ and dried. Yield: 75.9 mg, 99%.

Supplementary Figs. 43-44 show ^{1}H, ^{13}C{^{1}H}, and ^{1}H-^{15}N HMBC NMR spectra of **4-H**.

^{1}H NMR (500.13 MHz, CD_3NO_2, 298 K): δ 4.38 (4H, s, 2 C*H₂*), 6.32 (4H, br pseudo t, 3,4-position of *C₅H₄* groups), 6.44 (4H, br pseudo t, 2,5-position of *C₅H₄* groups), 7.56 (2H, d, $^3J_{HH}$, = 7.9 Hz, 3,5-position of pyridine ring), 8.14 (1H, d, $^3J_{HH}$, = 7.9 Hz, 4-position of pyridine ring).

^{13}C{^{1}H} NMR (125.76 MHz, CD_3NO_2, 298 K): δ 40.6 (s, 2 *C*H₂), 93.5 (s, 3,4-position of *C₅H₄* groups), 96.1 (s, 2,5-position of *C₅H₄* groups), 127.0 (s, 3,5-position of pyridine ring), 139.5 (s, 1-position of *C₅H₄* groups), 144.4 (s, 4-position of pyridine ring), 179.1 (s, 2,6-position of pyridine ring).

^{15}N NMR (50.68 MHz, CD_3NO_2, 298 K, detected using ^{1}H-^{15}N HMBC): δ 198.1 (s).

^{19}F NMR (470.54 MHz, CD_3NO_2, 298 K): δ 126.78 (overlapping br sextet ($^1J_{F\text{-}121Sb}$, = 1970 Hz), and br octet ($^1J_{F\text{-}123Sb}$, = 1050 Hz)).

XPS (298 K): 709.0 eV (Fe $2p^{3/2}$), 722.0 eV (Fe $2p^{1/2}$).

Elemental analysis: Calcd: C:26.84, H:1.99, N:1.84. Found: C:26.44, H:1.85, N:1.92.

HRMS (ESI/TOF, $[M-(SbF_6)_2]^+$): Calcd for $C_{17}H_{15}N_1Fe_1$: 289.0548. Found: 289.0557.

Preparation of **4-NMe₂**

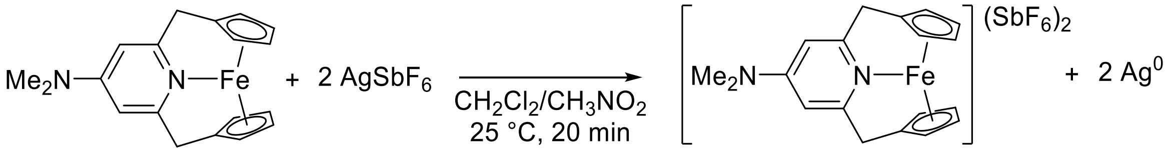

2-NMe₂ **4-NMe₂**

In a nitrogen glovebox, a 20 mL vial equipped with a Teflon-coated stirring bar was charged with **2-NMe₂** (17.0 mg, 0.051 mmol) and 2 mL dichloromethane. To the stirring purple solution was added $AgSbF_6$ (35.1 mg, 0.102 mmol) using 2 mL nitromethane at 25 °C. The dark brown solution was stirred for 20 min at 25 °C in a dark room. The dark brown solution was passed through a plug of Celite to remove the black solid of Ag^0, and the Celite was washed three times with a 1:1 nitromethane: dichloromethane mixture. The dark purple solution was concentrated to about 3 mL and layered with about 12 mL of benzene. Brown crystals of **4-NMe₂** suitable for SC-XRD analysis were obtained after 2 days. The crystals were washed three times with dichloromethane and dried to obtain 10.7 mg of **4-NMe₂**. Another crop of crystals (6.6 mg) was obtained upon standing of decanted mother liquor at 25 °C. Total yield: 17.3 mg, 42%.

Supplementary Figs. 45-46 show ^{1}H, ^{13}C, and ^{1}H-^{15}N HMBC NMR spectra of **4-NMe₂**.

^{1}H NMR (500.13 MHz, CD_3NO_2, 298 K): δ 3.17 (6H, s, N(C*H₃*)₂), 4.06 (4H, s, 2 C*H₂*), 6.21 (4H, br pseudo t, 3,4-position of *C₅H₄* groups), 6.31 (4H, br pseudo t, 2,5-position of *C₅H₄* groups), 6.79 (1H, s, 3,5-position of pyridine ring).

$^{13}C\{^1H\}$ NMR (125.76 MHz, CD_3NO_2, 298 K): δ 39.7 (s, 2 CH_2), 40.2 (s, $N(CH_3)_2$), 92.4 (s, 3,4-position of C_5H_4 groups), 95.7 (s, 2,5-position of C_5H_4 groups), 110.5 (s, 3,5-position of pyridine ring), 139.8 (s, 1-position of C_5H_4 groups), 158.4 (s, 4-position of pyridine ring), 176.6 (s, 2,6-position of pyridine ring).
^{15}N NMR (50.68 MHz, CD_3NO_2, 298 K, detected using ^1H-^{15}N HMBC): δ 74.7 (s, $N(CH_3)_2$), 123.0 (s, pyridyl *N*).
^{19}F NMR (470.54 MHz, CD_3NO_2, 298 K): δ 128.29 (br pseudo d).
Elemental analysis: Calcd: C:28.39, H:2.51, N:3.49. Found: C:28.10, H:2.27, N:3.30.
HRMS (ESI/TOF, $[M-(SbF_6)_2]^+$): Calcd for $C_{19}H_{20}N_2Fe_1$: 332.0971. Found: 332.0982.

Preparation of [Co(CpNCp-NMe$_2$)] **(Co-NMe$_2$)**

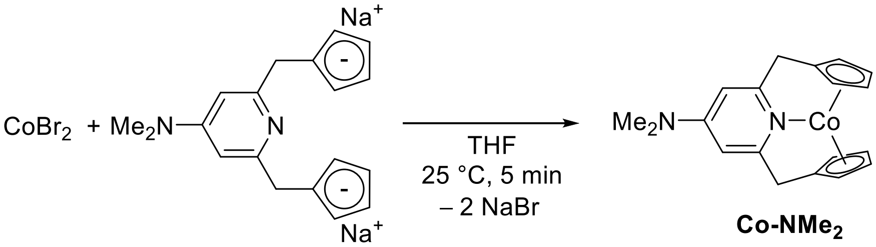

In a nitrogen glovebox, a 20 mL vial equipped with a Teflon-coated stirring bar was charged with $CoBr_2$ (22.8 mg, 0.10 mmol) and 4 mL THF. To the clear blue solution at 25 °C was then added **NMe$_2$-CpNCpNa$_2$** (32.5 mg, 0.10 mmol) in 1 mL THF. The vial containing **NMe$_2$-CpNCpNa$_2$** was washed two times with 0.5 mL each of THF and the THF solution was added to the solution of $FeBr_2$. After the addition, the brown suspension was stirred for 5 min and 6 mL n-pentane was added. The resulting red solution with grey precipitate was passed through a plug of Celite, and the Celite was washed three times with 1:1 THF:n-pentane mixture. The concentration of the combined THF/n-pentane solution gave a red-orange crystalline solid of **Co-NMe$_2$**. Yield: 19.2 mg, 57%. Consistent with the NMR analysis, SC-XRD measurement showed formation of the *N*-coordinated, formal 21-electron complex. Supplementary Figs. 47-48 show ^1H, and $^{13}C\{^1H\}$ NMR, and FTIR data of **Co-NMe$_2$**.
^1H NMR (500.13 MHz, C_6D_6, 298 K): δ -255.7 (4H, $\Delta\nu^{1/2}$ = 1267 Hz, C_5H_4 groups), -185.2 (4H, $\Delta\nu^{1/2}$ = 870 Hz, C_5H_4 groups), 0.92 (6H, $\Delta\nu^{1/2}$ = 3.8 Hz, $N(CH_3)_2$), 81.5 (2H, $\Delta\nu^{1/2}$ = 76 Hz, 3,5-position of pyridine ring), 150.6 (4H, $\Delta\nu^{1/2}$ = 211, 2 CH_2). Assigned based on integration values and comparison to ^1H NMR spectra of previously reported S = 3/2, Co(CpNCp-H) complex.
^{13}C NMR (125.76 MHz, C_6D_6, 298 K): δ -498.3 (br), -176.7 (br), 60.4 (distorted q, $^1J_{C\text{-}H}$ = 131.8 Hz, $N(CH_3)_2$), 284.5 (s), 358.3 (d, $^1J_{C\text{-}H}$ = 152.7 Hz, pyridyl 2 *C*H). Other ^{13}C NMR signals were not detectable.
FTIR (KBr pellet, cm^{-1}): 1610 (s, C=C and C=N stretch), 1536, 1417, 1383, 1024, 800, 752, 655.
HRMS (ESI/TOF, $[M]^+$): Calcd for $C_{19}H_{20}N_2Co_1$: 335.0953. Found: 335.0966.

Preparation of [Co(CpNCp-NMe$_2$)]BF$_4$ (**[Co-NMe$_2$]BF$_4$**)

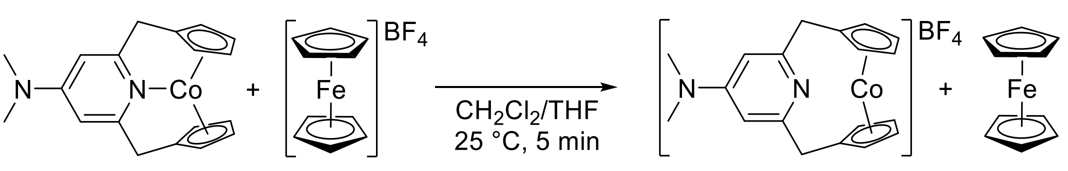

In a nitrogen glovebox, a 20 mL vial equipped with a Teflon coated stirring bar was charged with **Co-NMe2** (15.9 mg, 0.047 mmol), and 2 mL THF. To the clear red solution was then added ferrocenium tetrafluoroborate (13.1 mg, 0.048 mmol) using 4 mL CH_2Cl_2. The solution was stirred at 25 °C for 5 min, and dark purple solution was obtained. The solution was filtered using plug of Celite, and the Celite was washed by CH_2Cl_2. The combined solution was concentrated to dryness. The resulting solid was washed three times with n-pentane and dried under vacuum. Yield: 20.2 mg, 100%. Consistent with the NMR analysis, SC-XRD measurement showed formation of the *N*-noncoordinated, formal 18-electron complex.

Supplementary Figs. 49-51 show ^{1}H, ^{13}C{^{1}H}, and ^{1}H-^{15}N HMBC, NMR, and FTIR spectra of **3**.
^{1}H NMR (500.13 MHz, CD_3CN, 298 K): δ 3.02 (6H, s, 2 N*CH3*), 3.10 (4H, s, 2 *CH2*), 5.55 (4H, broad pseudo t, 3,4-position of *C5H4* groups), 5.60 (4H, pseudo t with roofing, $^3J_{CH}$, $^4J_{CH}$ = 2.0 Hz, 2,5-position of *C5H4* groups), 6.53 (2H, s, 3,5-position of pyridine ring).
^{13}C{^{1}H} NMR (125.76 MHz, CD_3CN, 298 K): δ 32.6 (s, 2 *C*H2), 39.9 (s, 2 N*C*H3), 76.7 (s, 3,4-position of *C5H4* groups), 87.6 (s, 2,5-position of *C5H4* groups), 103.6 (s, 3,5-position of pyridine ring), 125.4 (s, 1-position of *C5H4* groups), 155.2 (s, 2,6-position of pyridine ring), 157.9 (s, 4-position of pyridine ring).
^{15}N NMR (50.68 MHz, CD_3CN, 298 K, detected using ^{1}H-^{15}N HMBC): δ 58.3 (s, *N*$(CH_3)_2$), 282.3 (s, pyridine N).
FTIR (Thin film, cm^{-1}): 1597 (s, C=C and C=N stretch), 1053 (very strong, orvelapping sp^2 C-H bending and B-F stretch), 850 (m, sp^2 C-H bending).
HRMS (ESI/TOF, $[M]^+$): Calcd for $C_{19}H_{20}N_2Co_1$: 335.0953. Found: 335.0961.

<u>Preparation of [Ru(CpNCp-NMe2)] **(Ru-NMe2)**</u>

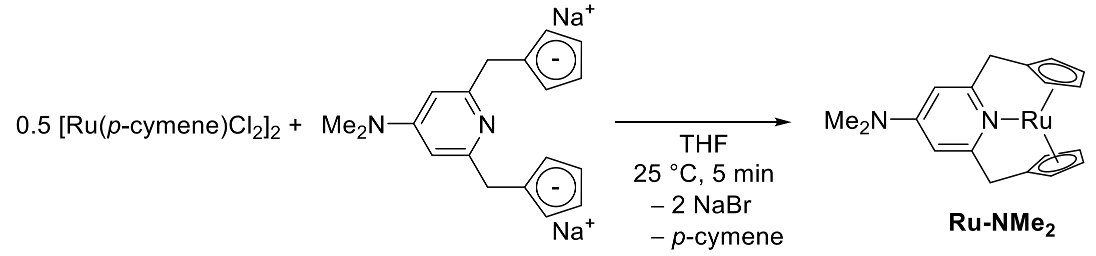

In a nitrogen glovebox, a 20 mL vial equipped with a Teflon-coated stirring bar was charged with $[Ru(p\text{-cymene})Cl_2]_2$ (31.4 mg, 0.051 mmol) and 4 mL CH_2Cl_2. To the clear orange solution at 25 °C was then added **NMe2-CpNCpNa2** (32.2 mg, 0.10 mmol) in 1 mL THF. The vial containing **NMe2-CpNCpNa2** was washed two times with 0.5 mL each of THF and the THF solution was added to the solution of $FeBr_2$. After the addition, orange cloudy solution was stirred for 5 min. The resulting orange solution with cloudy precipitate was passed through a plug of Celite, and the Celite was washed three times with 2:1 THF: CH_2Cl_2 mixture. The concentration of the combined solution gave an orange film. The product was extracted using benzene, and the benzene solution was concentrated to dryness. The crude product was dissolved in C_6D_6 and analyzed using NMR. NMR analysis showed the formation of **Ru-NMe2** and *p*-cymene coordinated complex. Heating this solution for 30 min at 80 °C resulted in clean formation of *p*-cymene and **Ru-NMe2**. The C_6D_6 solution was concentrated, and the resulting yellow solid was washed three times with pentane and dried. Yield: 5.4 mg, 14 %. Consistent with the NMR analysis, SC-XRD measurement showed formation of the *N*-noncoordinated, formal 18-electron complex.
Supplementary Figs. 52-54 show ^{1}H, and ^{13}C{^{1}H} NMR, and FTIR data of **Co-NMe2**.

^{1}H NMR (500.13 MHz, C_6D_6, 298 K): δ 2.37 (6H, s, 2 N*CH_3*), 3.07 (4H, s, 2 *CH_2*), 4.39 (4H, pseudo t, $^{3}J_{CH}$, $^{4}J_{CH}$ = 1.7 Hz 3,4-position of *C_5H_4* groups), 4.84 (4H, pseudo t with roofing, $^{3}J_{CH}$, $^{4}J_{CH}$ = 1.7 Hz, 2,5-position of *C_5H_4* groups), 6.05 (2H, s, 3,5-position of pyridine ring).
^{13}C{^{1}H} NMR (125.76 MHz, C_6D_6, 298 K): δ 34.3 (s, 2 *C*H_2), 38.9 (s, 2 N*C*H_3), 66.4 (s, 3,4-position of *C_5H_4* groups), 74.8 (s, 2,5-position of *C_5H_4* groups), 101.6 (s, 3,5-position of pyridine ring), 110.6 (s, 1-position of *C_5H_4* groups), 154.6 (s, 4-position of pyridine ring), 167.7 (s, 2,6-position of pyridine ring).
^{15}N NMR (50.68 MHz, C_6D_6, 298 K, detected using ^{1}H-^{15}N HMBC): δ 51.5 (s, *N*(CH_3)$_2$), 296.5 (s, pyridine N).
FTIR (Thin film, cm^{-1}): 1592 (s, C=C and C=N stretch), 1500, 1391, 1153, 1021, 850, 801.
HRMS (ESI/TOF, $[M + H]^{+}$): Calcd for $C_{19}H_{21}N_2Ru_1$: 379.0748. Found: 379.0755.

Supplementary Note 3. Mössbauer spectroscopy

Mössbauer spectra were recorded on a multichannel analyzer (Laboratory Equipment). The sample temperature was controlled using a liquid-nitrogen-flow-type cryostat (Oxford Instruments). Zero-field ^{57}Fe Mössbauer spectra were recorded using a ^{57}Co source with the doppler velocity range of ± 4 mm s^{-1}. The spectrometer (Topologic Systems) was calibrated with a spectrum of α-Fe foil at 300 K. In a nitrogen glovebox at OIST, samples were prepared by evenly spreading 35-100 mg of powdered solids to a 15 mm x 15 mm area at the center of 75 mm x 100 mm sheets of parafilm. The sheets were then folded multiple times to seal the solid in about 15 mm x 15 mm size. The sealed sample was placed in a heat-sealable nylon bag, and the bag was heat-sealed inside the glovebox. The resulting 20 mm x 20 mm sample was then sealed twice in larger sizes of heat-sealable nylon bags inside the glovebox. The sample was then shipped to the Mössbauer spectroscopy facility at Nagoya Institute of Technology. At the facility, the 20 mm x 20 mm samples were taken out from the nylon bags set to the spectrometer, and the spectra were recorded at 77 K using liquid nitrogen. The isomer shifts are reported relative to the center of the α-Fe signal at 300 K. The data was analyzed using NORMOS software, and quadrupole doublets were fitted to Voigt line shapes.
Table S1 shows best-fit parameters for Mössbauer spectra of **1-X**, **2-X**, **3-H**, and **4-H**. Figs. S55-57 shows Mössbauer spectra of **1-X**, **2-X**, **3-H**, and **4-H** recorded at 77 K.

Supplementary Table 1. Best fit parameters for Mössbauer spectra of **1-X**, **2-X**, **3-H**, and **4-H.**

Complex	Isomer shift δ (mm s^{-1})	Quadrupole splitting Δ (mm s^{-1})	Area (%)	Lorentzian width Γ (mm s^{-1})	Gaussian width (mm s^{-1})
**1-H**	0.63	2.65	100.0	0.28	0.09
**1-Cl**	0.60	2.59	100.0	0.28	0.13
**1-OMe**	0.63	2.64	58.4	0.28	0.12
**2-OMe**	1.18	1.90	41.6	0.28	0.11
**1-NMe$_2$**	0.56	2.52	5.6	0.28	0.11
**2-NMe$_2$**	1.15	1.78	94.4	0.28	0.07
**3-H**	0.68	2.87	100.0	0.28	0.15
**4-H**	0.48	3.76	100.0	0.28	0.09

a

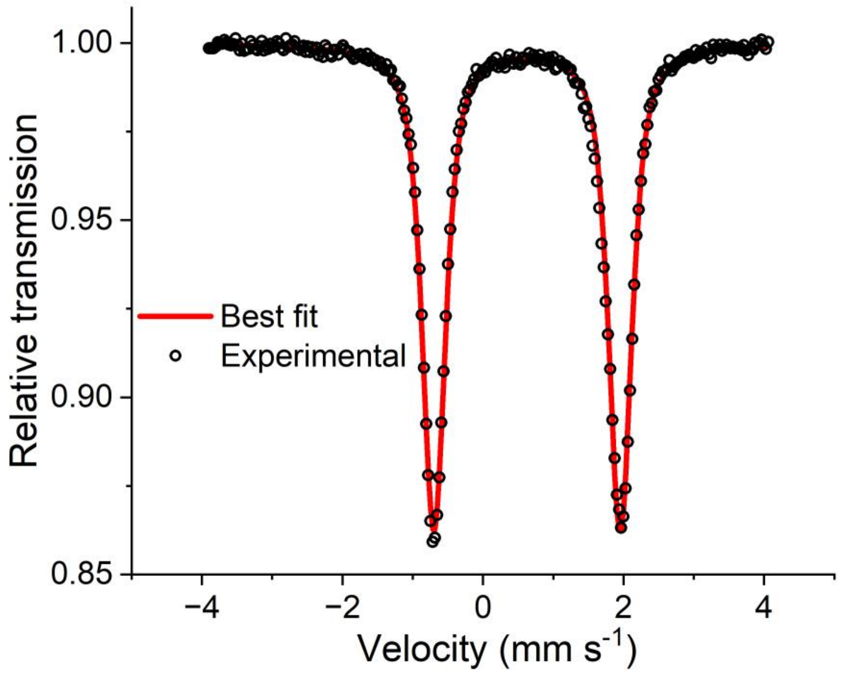

b

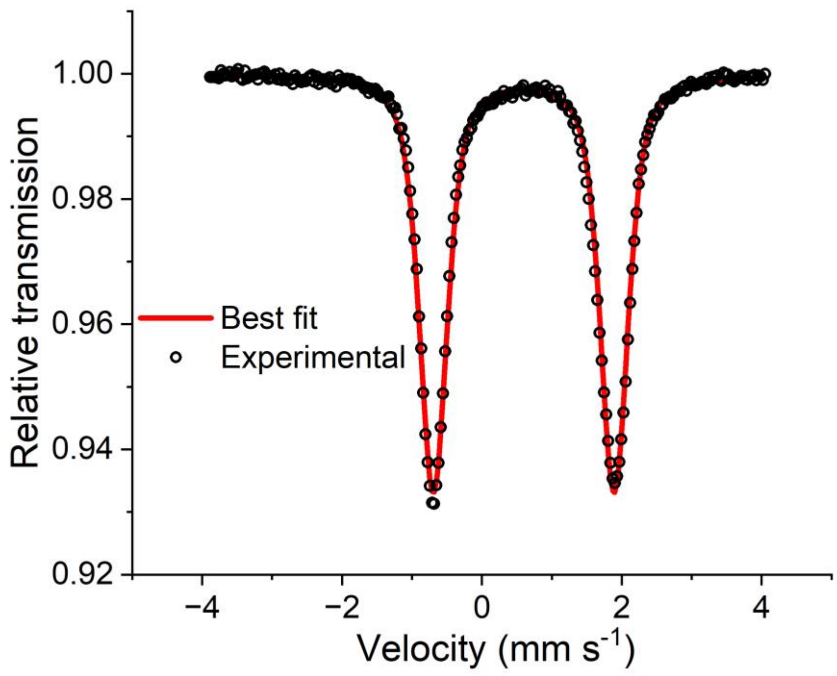

Supplementary Fig. 55.
Mössbauer spectra (77 K) of (**a**) **1-H** and (**b**) **1-Cl**

a

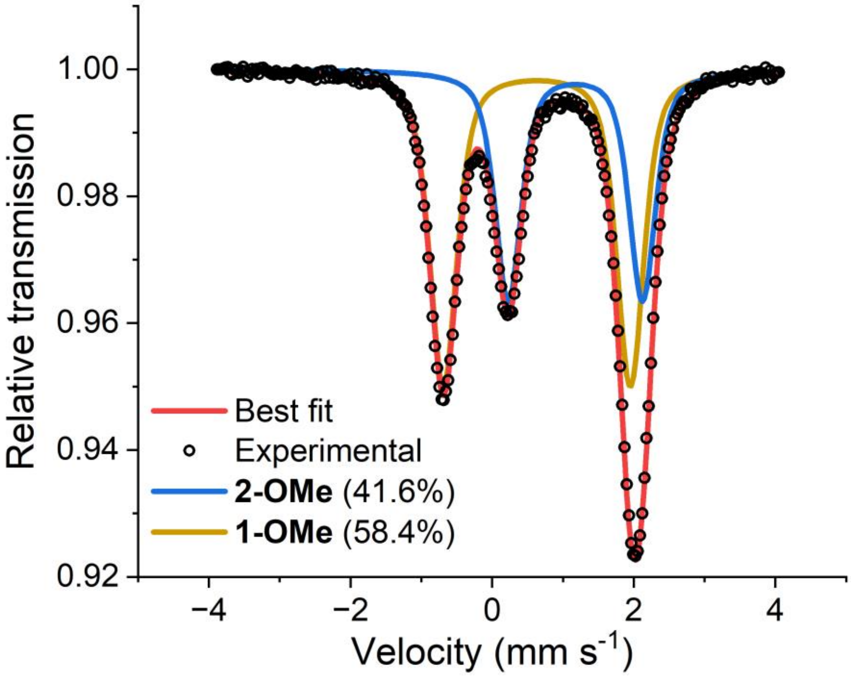

b

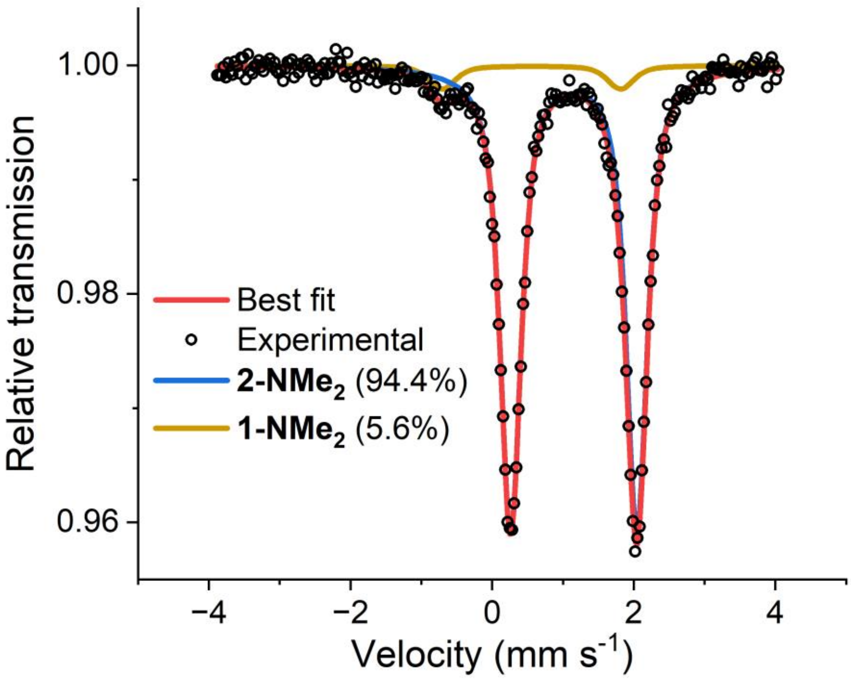

Supplementary Fig. 56.
Mössbauer spectra (77 K) of (**a**) **1-OMe** + **2-OMe** and (**b**) **1-NMe₂** + **2-NMe₂**.

a

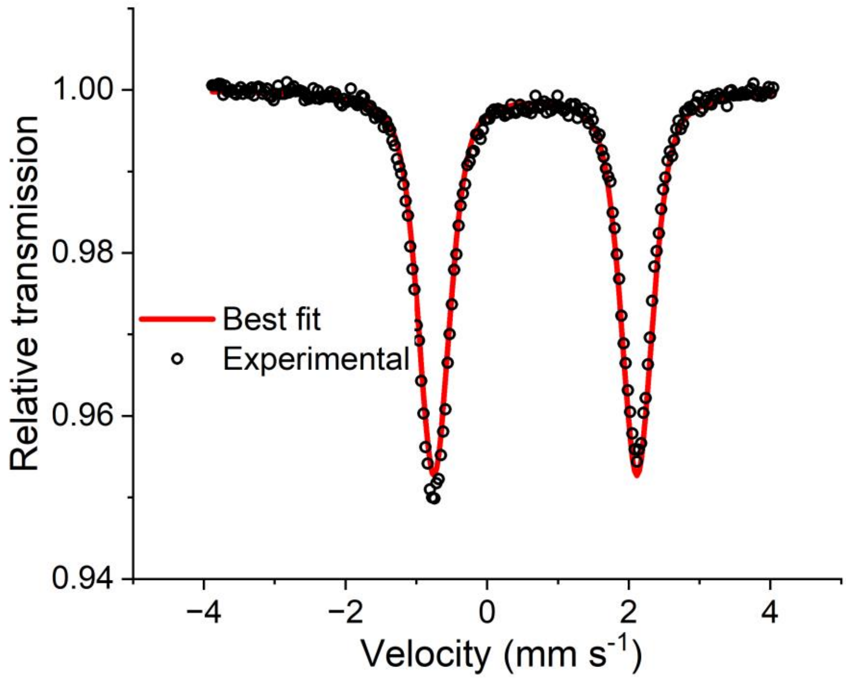

b

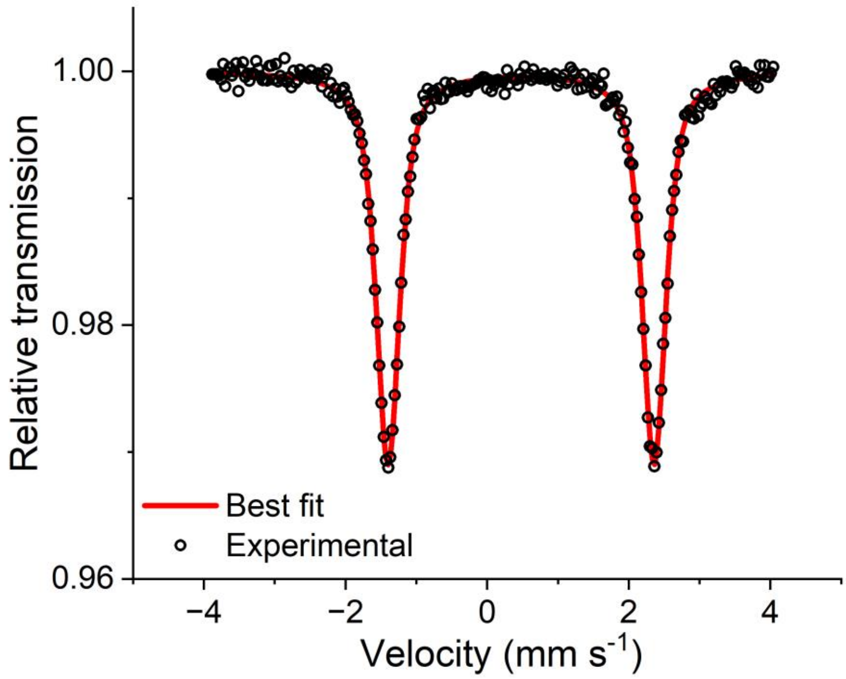

Supplementary Fig.57.
Mössbauer spectra (77 K) of (**a**) **3-H** and (**b**) **4-H**.

Supplementary Note 4. X-ray photoelectron spectroscopy (XPS)

XPS experiments were performed using a PHI Quantes instrument equipped with a monochromatic Al Kα source. A PHI Quantes special vessel was used for sample transfer from the nitrogen glovebox to the XPS chamber. The XPS samples were pressed on the top of the indium sheet attached to the double-sided carbon tape placed on the silicon substrate and placed on the instrument sample holder. The X-ray power of 50 W at 15 kV was used for a 200-micron beam size. The PHI double charge neutralization system was used on all samples. The ultimate Quantes instrumental resolution was better than 0.480 eV based on the Fermi edge of the valence band for sputtered metallic silver. XPS spectra were calibrated to the C1*s* peak of Highly Oriented Pyrolytic Graphite (HOPG, binding energy = 284.6 eV). XPS spectra with the energy step of 0.1 eV were recorded using software SmartSoft–VP v4.1.4.3 and processed using PHI MultiPack v9.9.1.1 at the pass energies of 55.0 for Fe 2*p*, N 1*s*, C 1*s*, O 1*s*, F 1*s*, Si 2*p*, P 2*p*, and Sb 3*d* regions, respectively.

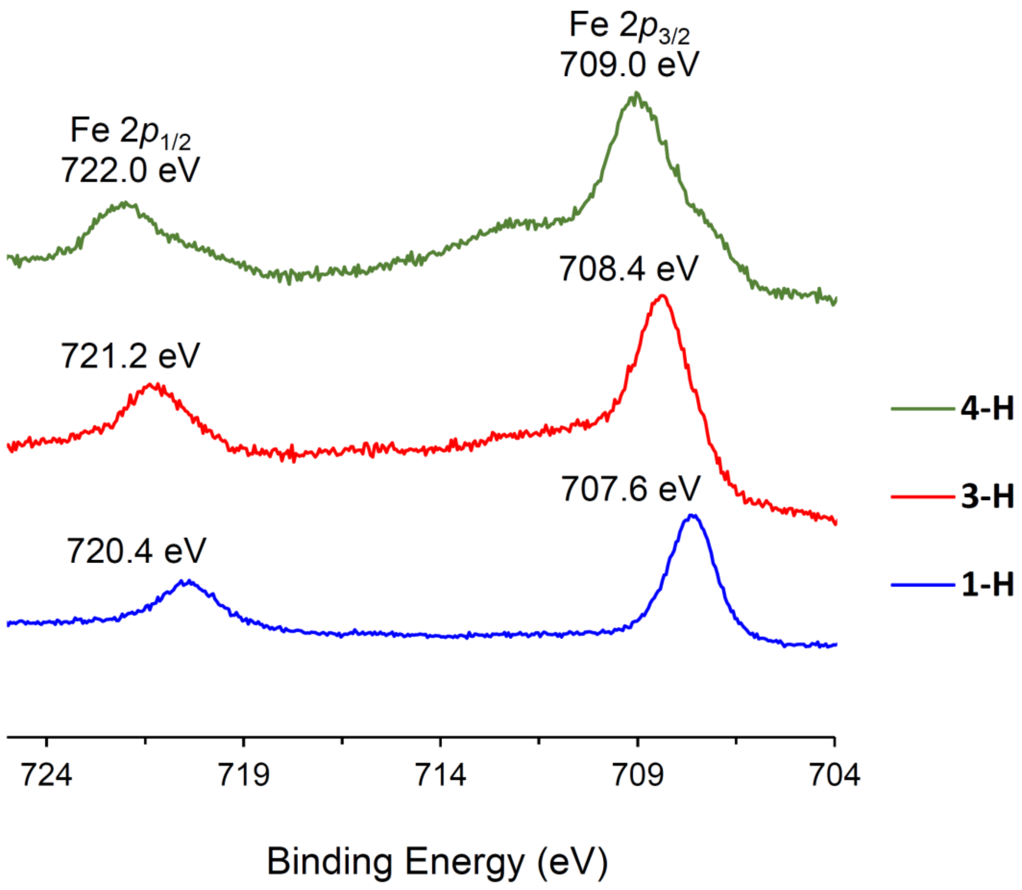

Supplementary Fig. 58.
Overlay of Fe 2*p* XPS spectra (298 K) of **1-H**, **3-H**, and **4-H**.

Supplementary Note 5. Computational study

Computational methods

DFT computations were performed with the ORCA software package (versions 5.0.2 and 5.0.4) unless otherwise noted[8-10]. For structure optimizations and harmonic vibrational frequencies, we employed the TPSS meta-GGA functional[11-13]. Additionally, we utilized Grimme's latest additive dispersion correction D4[14,15] and an Ahlrichs-type basis set with triple-zeta quality (def2-TZVPP)[16]. Minima on the potential energy surface were confirmed by the absence of imaginary frequencies. For accurate electronic energies and quasi-restricted orbitals (QROs)[17], we used the hybrid variant of this functional, i.e., TPSSh, together with the quadruple-zeta quality basis set (def2-QZVPP)[16]. Bulk solvation was considered implicitly using the SMD model for toluene, THF, and acetonitrile[18]. Unless noted otherwise, the resolution of identity (RI) and chain-of-spheres (RIJCOSX) approximations, as implemented in ORCA, were used for (meta)-GGA and hybrid functionals, respectively, together with the appropriate auxiliary basis set (def2/J)[19,20]. All computations performed with ORCA utilize tight SCF convergence criteria and the default integration grid (defgrid2). Analysis of the electron density was performed with Multiwfn 3.7[21].

Optimized structural parameters

In the neutral state, **1-X** and **2-X** are in equilibrium, with stronger donor ligands promoting the formation of the Fe−N bonded isomer **2-X**. In the oxidized state, only the Fe−N bonded derivatives **3-X** and **4-X** could be identified as minimum structures on the potential energy surface. Structure optimizations of singly or doubly oxidized **1-X** converged to **3-X** and **4-X**, respectively, exhibiting a Fe−N bond. Selected DFT-optimized structural parameters are given in Supplementary Table 2. The Fe−N bond length in **2-X** is 2.185 Å, which is accompanied by an increase in the Fe−Cp_c distance (2.01−2.04 Å) and a decrease in the Cp_c–Fe–Cp_c angle (146−150°) compared to **1-X**. Upon the first oxidation (**2-X** → **3-X**), the Fe−N bond is substantially elongated to 2.40−2.45 Å while the Fe−Cp_c distance is decreased to 1.73−1.74 Å. Removal of another electron (**3-X** → **4-X**) shortens the Fe−N bond significantly (2.049−2.057 Å), while the Fe−Cp coordination hardly changes structurally. The spin of the ground state of the individual compounds was verified by additional single-point energy computations (Supplementary Table 3).

Supplementary Table 2: Optimized bond lengths (in Å) and valence angles (in °) at TPSS-D4/def2-TZVPP level of theory. The spin of the ground state was verified by additional single-point energy computations (Supplementary Table 3)

	**1-X** (M_S = 1)			**2-X** (M_S = 5)		
X	Fe–N	Fe–Cp_c	Cp_c–Fe– Cp_c	Fe–N	Fe–Cp_c	Cp_c–Fe– Cp_c
Cl	3.012	1.657, 1.663	174.8	2.185	2.012, 2.031	147.0
H	3.006	1.657, 1.663	174.8	2.185	2.013, 2.032	146.8
OMe	3.026	1.657, 1.662	175.0	2.176	2.018, 2.032	146.1
NMe_2	3.054	1.656, 1.662	175.0	2.185	2.017, 2.039	150.0
	**3-X** (M_S = 2)			**4-X** (M_S = 1)		
X	Fe–N	Fe–Cp_c	Cp_c–Fe– Cp_c	Fe–N	Fe–Cp_c	Cp_c–Fe– Cp_c
Cl	2.449	1.735, 1.739	159.4	2.053	1.723, 1.724	147.0
H	2.453	1.732, 1.738	159.2	2.057	1.723, 1.724	146.9
OMe	2.423	1.732, 1.736	158.9	2.053	1.722, 1.723	147.4
NMe_2	2.401	1.733, 1.734	159.0	2.049	1.720, 1.721	147.9

Supplementary Table 3: Single-point energy computations at TPSSh-D4/def2-QZVPP//TPSS-D4/def2-TZVPP level considering different spin states. Electronic energies are given in kcal/mol, and the multiplicity of the ground state is highlighted in bold.

	**1-X**		**2-X**		
	$M_S = 3$	$\mathbf{M_S = 1}$	$\mathbf{M_S = 5}$	$M_S = 3$	$M_S = 1$
Cl	39.8	0.0	0.0	16.7	39.9
H	39.7	0.0	0.0	16.9	39.8
OMe	39.6	0.0	0.0	17.8	40.5
NMe_2	39.8	0.0	0.0	17.8	43.6
	**3-X**		**4-X**		
	$M_S = 4$	$\mathbf{M_S = 2}$	$M_S = 3$	$\mathbf{M_S = 1}$	
Cl	27.9	0.0	22.4	0.0	
H	28.2	0.0	23.1	0.0	
OMe	27.9	0.0	21.9	0.0	
NMe_2	27.6	0.0	18.9	0.0	

Calculated energies.

We calculated the relative energies of the **1-X**/**2-X** equilibrium. The results are summarized in Supplementary Table 4. The electronic energies in the gas phase at 0 K favor **1-X** and indicate the endothermic formation of the Fe−N bond ($\Delta E_{g,0} > 0$). Note that more electron-rich pyridine ligands (NMe_2 > OMe > H > Cl) increasingly favor the formation of **2-X**. Thermal, and entropic effects shift the equilibrium towards **2-X,** but the formation remains endergonic in the gas phase at 298 K ($\Delta G°_{g,298} > 0$).

Supplementary Table 4: Calculated zero-point corrected electronic energies at 0 K ($\Delta E_{g,0}$), enthalpies in the gas phase ($\Delta H°_{g,298}$), and Gibbs free energies at 298 K ($\Delta G°_{g,298}$) for Fe−N bond formation (**1-X** → **2-X**). Bulk solvation was considered implicitly with the SMD model for toluene, THF, and acetonitrile ($\Delta G_{s,298}$). * Values determined experimentally using ^{1}H NMR integration ratio of **1-NM2** and **2-NME2** at 230.2 K. All energies in kcal/mol.

					$\Delta G°_{s,298}$		
X	σ_{para}	$\Delta E_{g,0}$	$\Delta H°_{g,298}$	$\Delta G°_{g,298}$	toluene	THF	acetonitrile
Cl	0.23	6.3	7.6	3.4	1.8	1.3	1.0
H	0	5.8	7.1	3.0	1.3	0.7	0.4
OMe	−0.27	4.7	6.0	1.8	0.1	−0.5	−0.8
NMe_2	−0.83	4.5	5.8	1.7	−0.2 (1.4)*	−0.8 (−0.39)*	−1.1 (−0.74)*

We noticed that the formation of Fe-N bond is favoured with increasing donor strength of the substituents as observed experimentally. In fact, the reaction enthalpy correlates with the

Hammett para substituents constants (σ_{para})[22] (Supplementary Table 4, Supplementary Fig. 59). Moreover, the formation of Fe-N bond is favoured with increasing dipole moment of the solvent molecules (Supplementary Table 4, Supplementary Fig. 60) as observed experimentally (Supplementary Fig. 32).

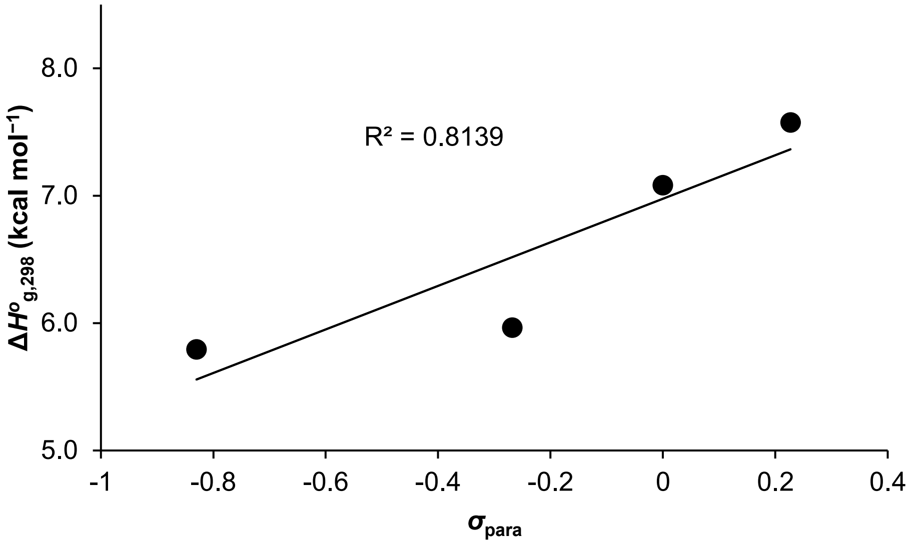

Supplementary Fig. 59.
Correlation of $\Delta H^{\circ}_{g,298}$ against the Hammett para substituents constants (σ_{para}).

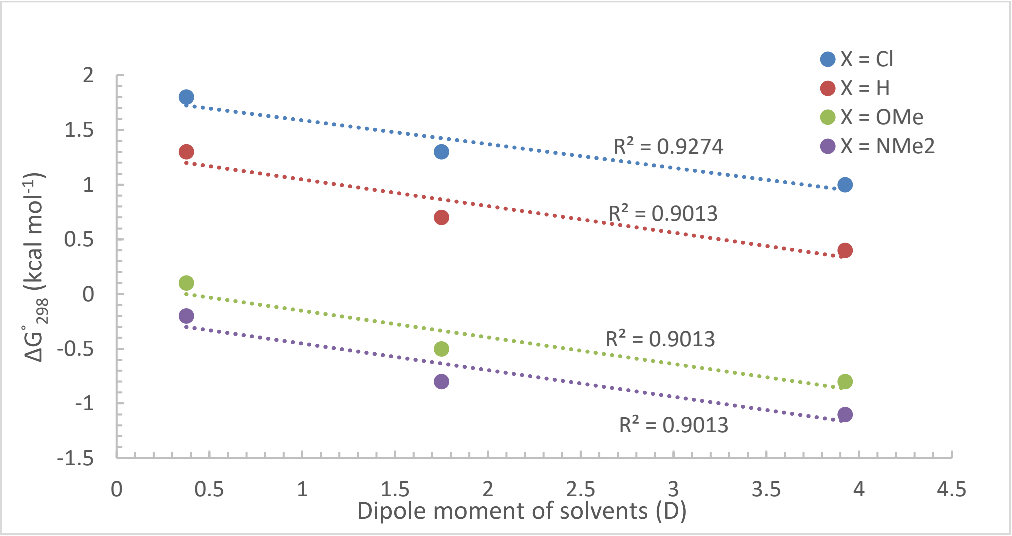

Supplementary Fig. 60.
Correlation of $\Delta G^{\circ}_{s,298}$ against dipole moment of solvents[23].

A closer inspection of the entropic contributions reveals that all components favor the formation of **2-X**, with the vibrational entropy having the greatest amount (Supplementary Table 5). In solution, however, **2-X** is stabilized with increasing polarity of the solvent, as evident from SMD computations to account for bulk solvation implicitly ($\Delta G^{\circ}_{s,298}$). Thus, the delicate interplay of the donor strength of the pyridine ligand, entropic effects caused by the **1-**

X /2-X transformation, and stabilizing solvent effects is decisive for the position of the equilibrium that favors **1-Cl** on one side and **2-NMe2** on the other.

Supplementary Table 5: Calculated entropic components for the Fe−N bond formation in the gas phase (**1-X** → **2-X**) at TPSS-D4/def2-TZVPP level. Translational (ΔS_{trans}), rotational (ΔS_{rot}), vibrational (ΔS_{vib}), and electronic (ΔS_{elec}) contributions and the sum ($\Delta_r S$) are given in cal/mol·K.

	ΔS_{trans}	ΔS_{rot}	ΔS_{vib}	ΔS_{elec}	sum ($\Delta_r S$)
Cl	0.0	0.3	10.5	3.2	14.0
H	0.0	0.3	10.2	3.2	13.7
OMe	0.0	0.2	10.5	3.2	13.9
NMe2	0.0	0.3	10.4	3.2	13.8

Electronic analysis of the Fe−N bond.

We performed a relaxed potential energy surface scan of the Fe−N bond interconnecting **1-NMe2** and **2-NMe2** (Supplementary Fig. 61). Thus, the intercept between the scans on the singlet and quintet potential energy surfaces reveals a minimum energy path with a low electronic barrier height of about 5 kcal/mol.

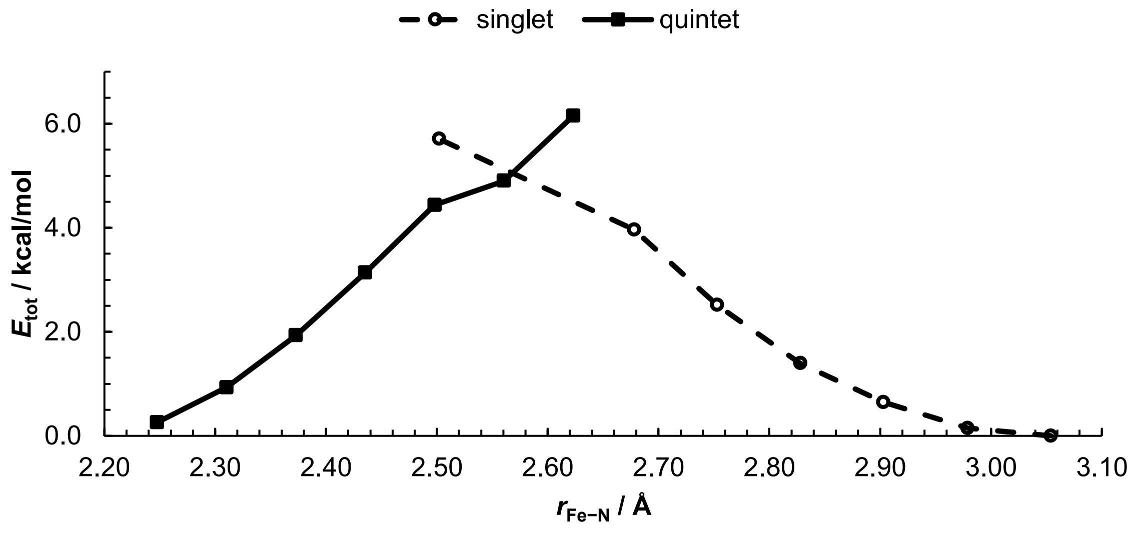

Supplementary Fig. 61.
Relaxed potential energy surface scan of the Fe−N bond of **1-NMe2**/**2-NMe2** in the gas phase at the TPSS-D4/de2-TZVPP level with subsequent single-point energies at the TPSSh-D4/def2-QZVPP level.

We analyzed the electron density of **1-X**, **2-X**, **3-X**, and **4-X** in terms of charge and spin population on Fe and the Laplacian of the electron density at the Fe−N bond critical point (Supplementary Table 6, Supplementary Fig. 62). The values hardly differ among the derivatives (R = Cl, H, OMe, NMe2) but differ notably for the coordination modes in **1-X**, **2-X**, **3-X**, and **4-X**. Strikingly, we located a bond critical point with a positive Laplacian ($\nabla^2\rho(r_{Fe-N}) > 0$) of the electron density between the Fe and N nuclei that is indicative of a coordinative Fe−N bond in **2-X**, **3-X**, and **4-X**. Concerning the open-shell complexes **2-X** and **3-X**, the residual spin density is predominantly located on Fe.

Supplementary Table 6: Calculated Hirshfeld charges at Fe, Laplacian at the bond critical point along the Fe–N axis, and spin population on Fe at TPSSh/def2-QZVPP//TPSS-D4/def2-TZVPP level.

	Fe charge (Hirshfeld)				$\nabla^2\rho(r_{\text{Fe–N}})$			Fe spin pop	
X	**1**	**2**	**3**	**4**	**2**	**3**	**4**	**2**	**3**
**Cl**	0.04	0.18	0.13	0.15	0.22	0.12	0.28	3.41	0.94
**H**	0.04	0.18	0.13	0.15	0.23	0.12	0.28	3.41	0.93
**OMe**	0.04	0.18	0.13	0.15	0.22	0.12	0.27	3.41	0.91
**NMe2**	0.04	0.18	0.13	0.15	0.22	0.13	0.27	3.41	0.89

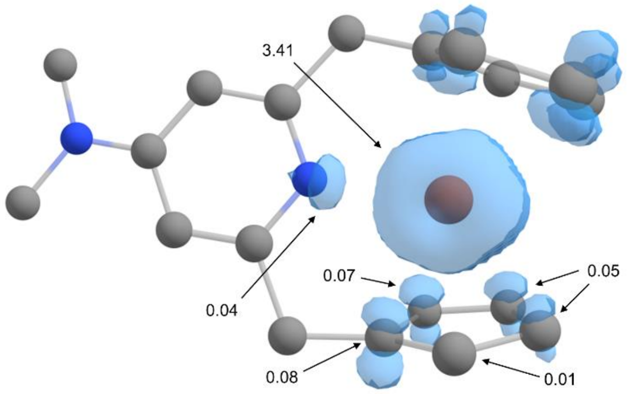

Supplementary Fig. 62.
Calculated spin population on **2-NMe2** at TPSSh/def2-QZVPP//TPSS-D4/def2-TZVPP level.

We also calculated the spin population for the Fe^{II}- and Fe^{III}-complexes (Supplementary Fig. 63). The highest spin density is located at the Fe-center and the substituents have an insignificant influence on the spin population. In contrast to the Fe^{III}-complexes, the Fe^{II}-complexes exhibit singly occupied antibonding Fe–Cp orbitals, and thus, there is residual spin density on the Cp ligands. In the Fe^{III} complexes, however, there is no spin density on the Cp ligands but on the pyridine ligand. Closer inspection reveals that the residual spin is mainly localized on the nitrogen (**3-H**: 0.08, **3-NMe2**: 0.09).

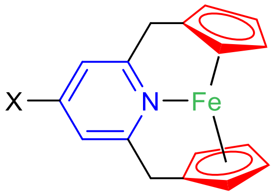

spin population

	**2-X** (Fe^{II})		**3-X** (Fe^{III})	
X =	H	NMe_2	H	NMe_2
Fe	3.41	3.41	0.93	0.89
2 Cp	0.47	0.50	-0.03	0
Py	0.06	0.06	0.09	0.11

Supplementary Fig. 63.
Calculated spin population of **2-H**, **2-NMe2**, **3-H**, and **3-NMe2** at TPSSh-D4/def2-QZVPP//TPSS-D4/def2-TZVPP level. Py: pyridine moiety.

Consistent with this finding is the antibonding Fe−N interaction of the singly occupied KS-MO (Supplementary Fig. 64). We investigated the influence of the oxidation state on the Fe−N bond order (Supplementary Table 7). Starting from Fe(II) in **2-H**, oxidation to Fe^{III} reduces the bond order from 0.57 to 0.42. Another oxidation increases the Fe−N bond order to 0.74.

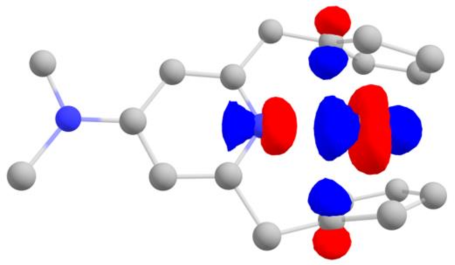

Supplementary Fig. 64.
Depiction of the singly occupied molecular orbital (SOMO) of **3-NMe2** computed as quasi-restricted canonical KS-MO (isovalue = 0.05).

Supplementary Table 7: Calculated Wiberg bond indices of the Fe−N bond in **2-X**, **3-X**, and **4-X** (X = H, NMe_2).

X	Fe^{II}	Fe^{III}	Fe^{IV}
H	0.57	0.42	0.74
NMe_2	0.57	0.46	0.76

Metal-N bond strength of the Mn^{II} or Co^{II} analogues of **2-X** (X = H or NMe_2) are examined based on DFT calculation. The calculated Wiberg bond indices of metal−N bonds are similar and virtually indistinguishable (Supplementary Table 8). Consistent with this observation, experimentally determined metal-N bond lengths of **2-NMe2** and **Co-NMe2**, 2.1476(10) and 2.1860(12) Å respectively, are similar.

Supplementary Table 8: Calculated Wiberg bond indices of the metal−N bond in **2-H** and **2-NMe2** and its Mn- and Co-analogue.

	Mn^{II*}	Fe^{II}	Co^{II*}
H	0.55	0.57	0.54
NMe_2	0.58	0.57	0.56

*Mn and Co-derivatives of **2-X** were computed in the sextet (M_S = 6) and quartet (M_S = 4) spin states, respectively.

Molecular Orbitals.

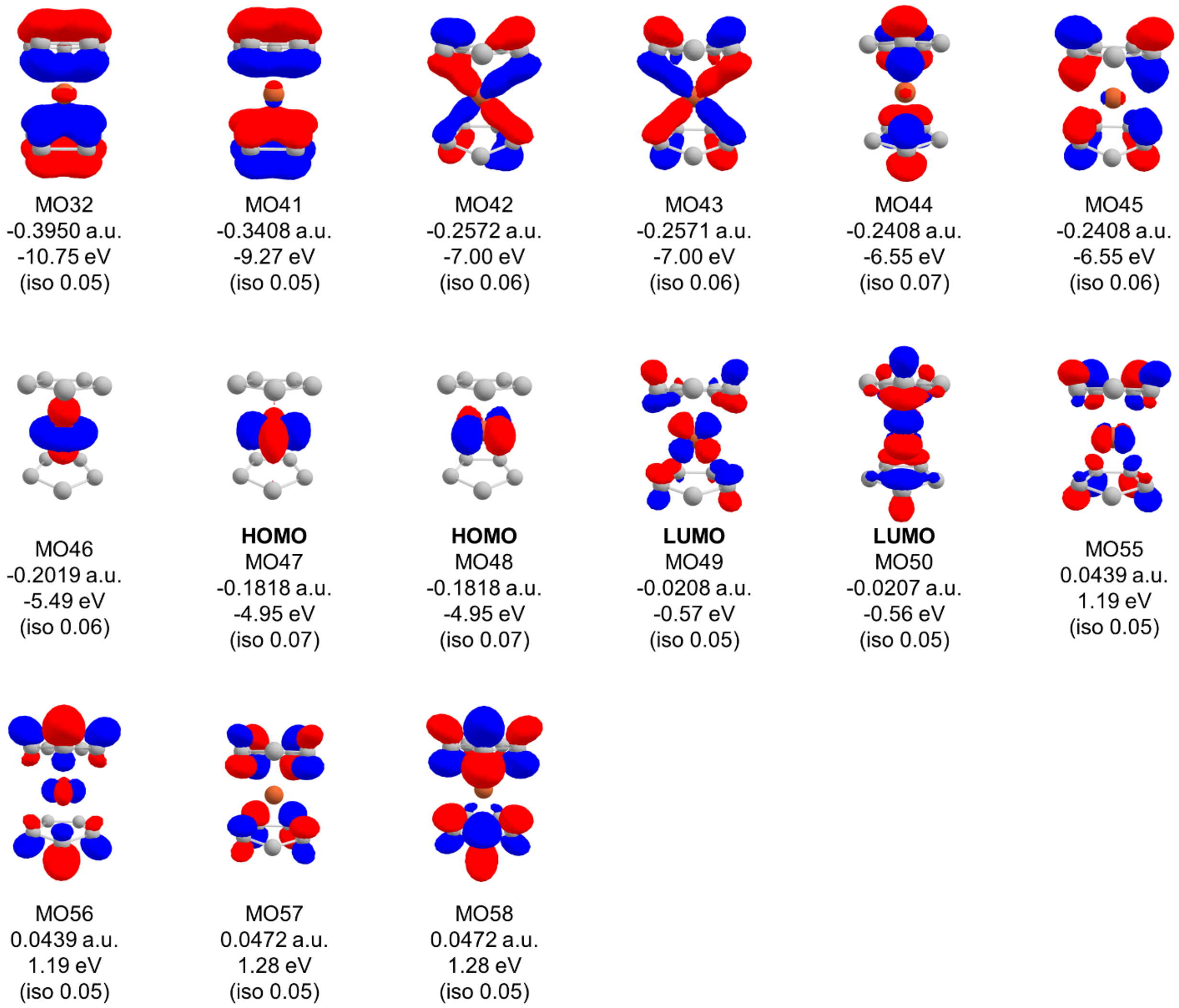

Supplementary Fig. 65: Canonical Kohn–Sham molecular orbitals of ferrocene computed at TPSSh/def2-QZVPP//TPSS-D4/def2-TZVPP. Energies are given in plain text and isovalues in parentheses.

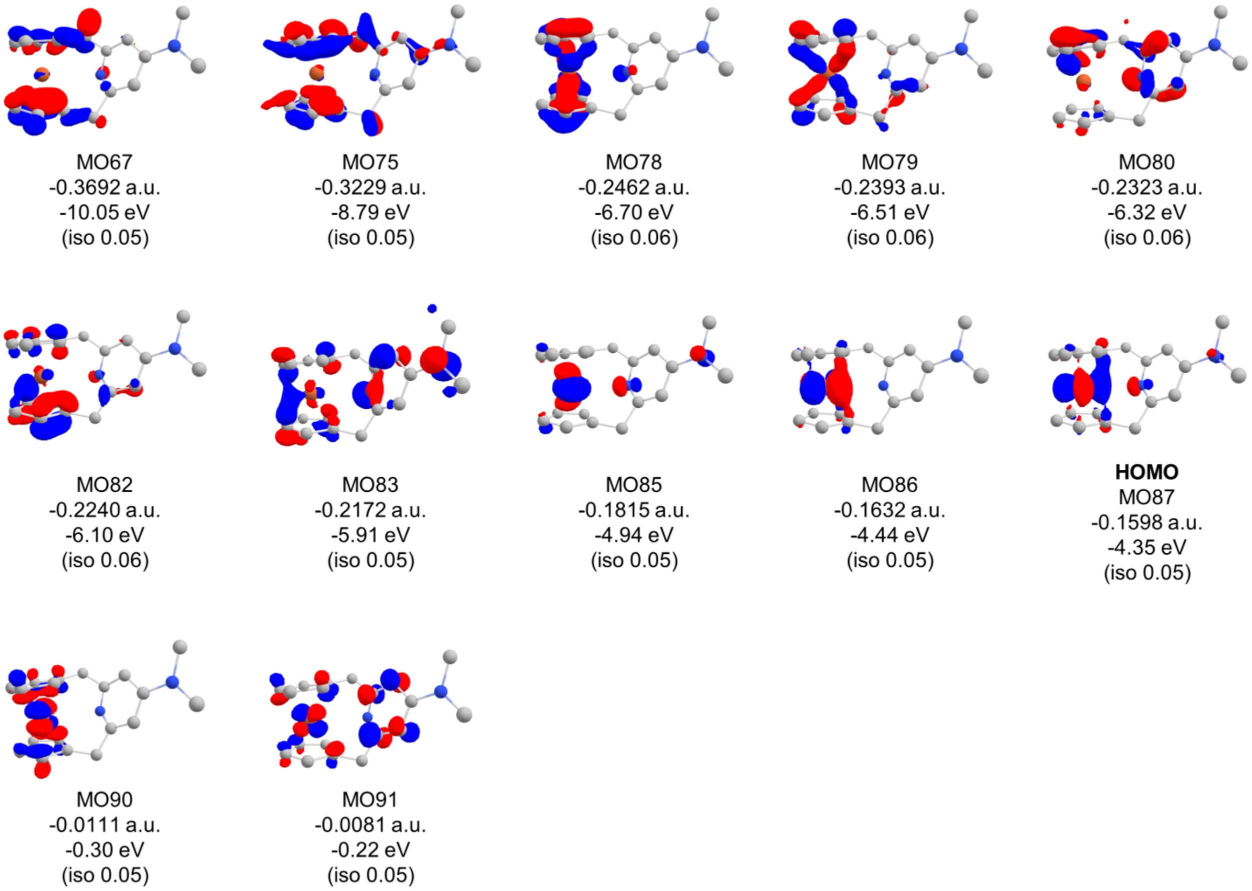

Supplementary Fig. 66: Canonical Kohn–Sham molecular orbitals of **1-NMe$_2$** computed at TPSSh/def2-QZVPP//TPSS-D4/def2-TZVPP. Energies are given in plain text and isovalues in parentheses.

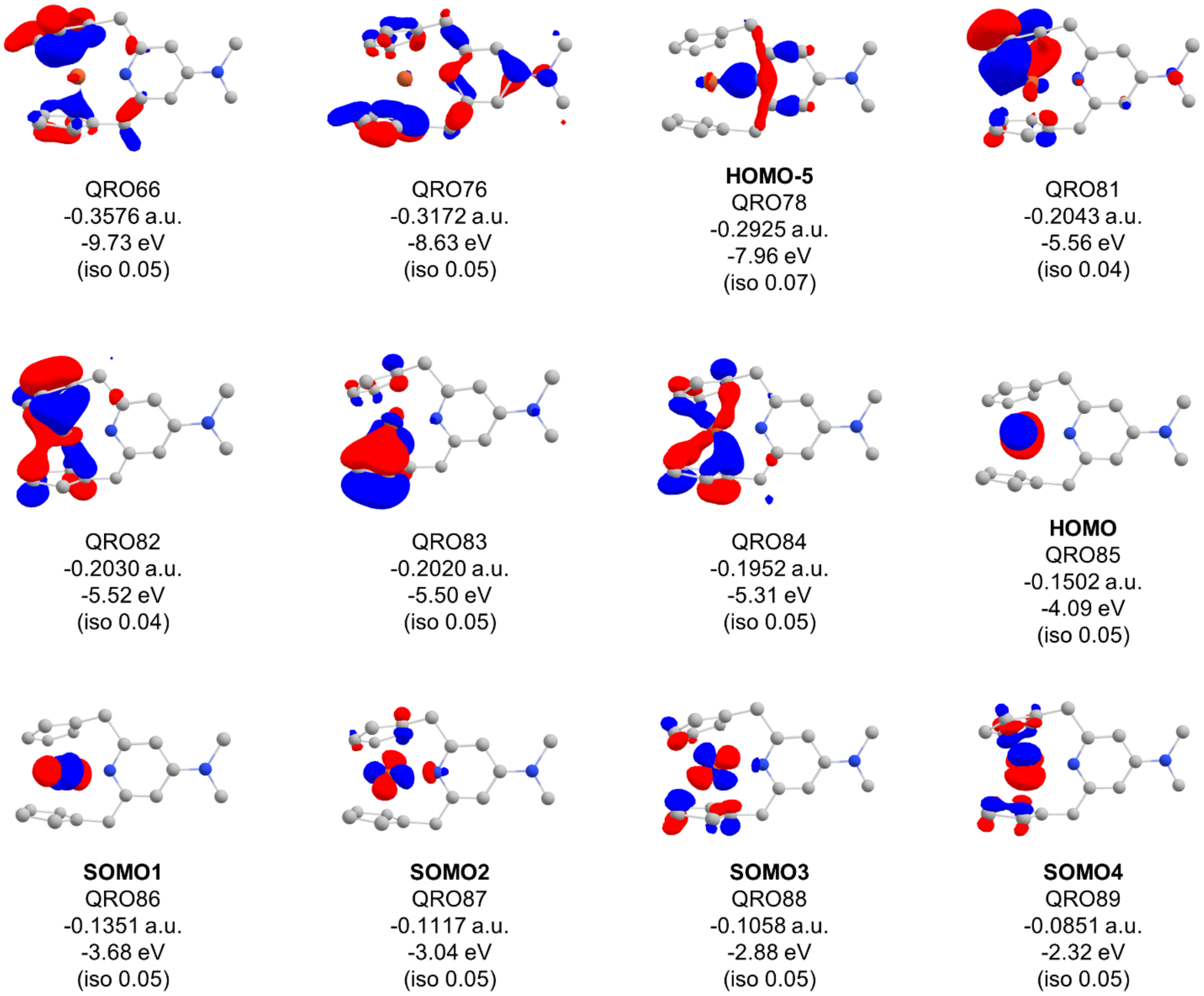

Supplementary Fig. 67: Canonical Kohn–Sham molecular orbitals of **2-NMe₂** computed at TPSSh/def2-QZVPP//TPSS-D4/def2-TZVPP. Energies are given in plain text and isovalues in parentheses.

Supplementary Note 6. Quantum topological study

A topological analysis of the theoretical electron density $\rho(\mathbf{r})$ and the per-particle potentials $\varphi_i(\mathbf{r})$ was performed using *Multiwfn* 3.8(dev)[24]. The electrostatic potential $\varphi_{\text{es}}(\mathbf{r})$ was computed using the built-in code[25]. The exchange-correlation potential $\varphi_x(\mathbf{r})$ and the total static potential $\varphi_{\text{em}}(\mathbf{r}) = -\varphi_{\text{es}}(\mathbf{r}) + \varphi_x(\mathbf{r})$ were approximated via the Müller relation[26]. The Coulomb correlation was not considered in this analysis. The kinetic potential $\varphi_k(\mathbf{r})$ can be represented as $\varphi_k(\mathbf{r}) = \mu - \varphi_{\text{em}}(\mathbf{r})$, where the electronic chemical potential μ is constant in our cases. The electrostatic and total static force densities, $\mathbf{F}_{\text{es}}(\mathbf{r})$ and $\mathcal{F}(\mathbf{r})$, were calculated as $\mathbf{F}_{\text{es}}(\mathbf{r}) = -\nabla[-\nabla\varphi_{\text{es}}(\mathbf{r})]$ and $\mathcal{F}(\mathbf{r}) = -\nabla\varphi_{\text{em}}(\mathbf{r})$. In equilibrium states, the equality $\mathbf{F}_k(\mathbf{r}) = -\mathcal{F}(\mathbf{r})$ holds, where $\mathbf{F}_k(\mathbf{r}) = -\nabla\varphi_k(\mathbf{r})$ represents the total kinetic force. The protocol for constructing superposition maps of the vector fields, along with the underlying principles of their interpretation, is detailed in our previous works [27,28]. The theoretical and practical aspects of the application of internuclear static potential barriers in chemical structure analysis is discussed in the referenced literature [29-31]. The electron delocalization indices δ(Fe, X)[32,33] were calculated using *AIMAll* (version 19.10.12, T. A. Keith, TK Gristmill Software, Overland Park KS, USA Version 19.10.12 (2019). https://aim.tkgristmill.com/).

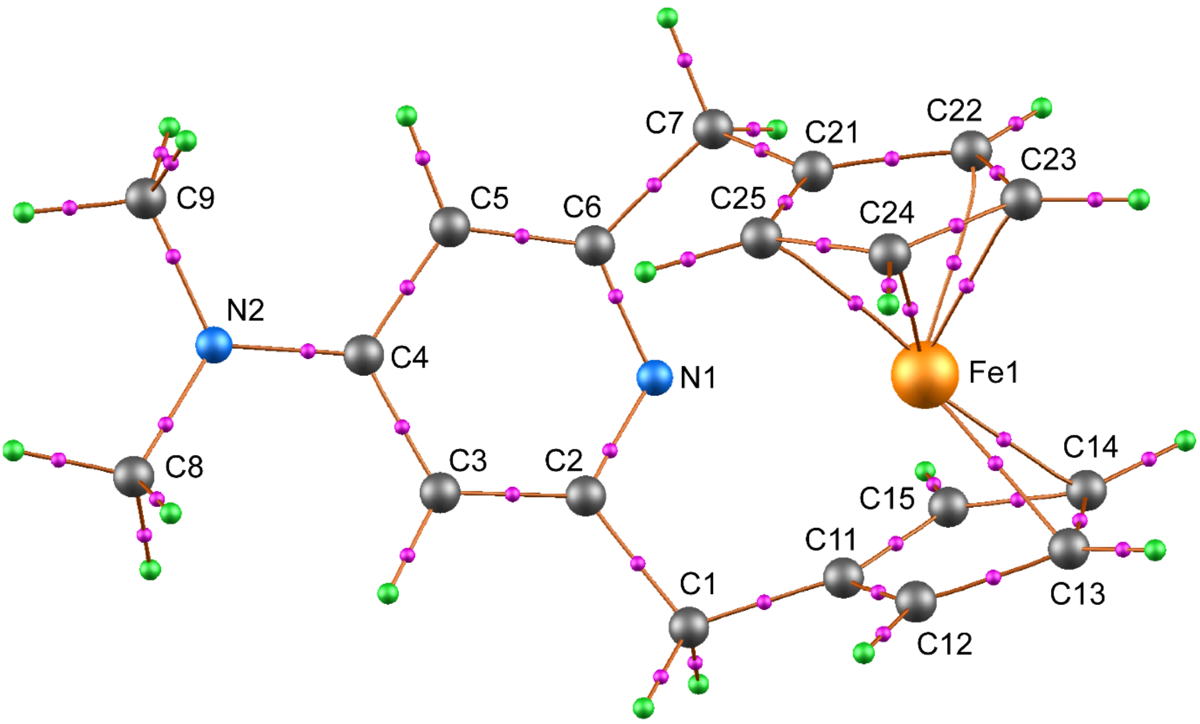

Supplementary Fig. 68. Molecular graph of the 18-electron ferrocene derivative **1-NMe₂**, based on theoretical data. The critical points (3, –1) in $\rho(\mathbf{r})$ are depicted as magenta spheres, while the corresponding internuclear bond paths are represented by brown lines.

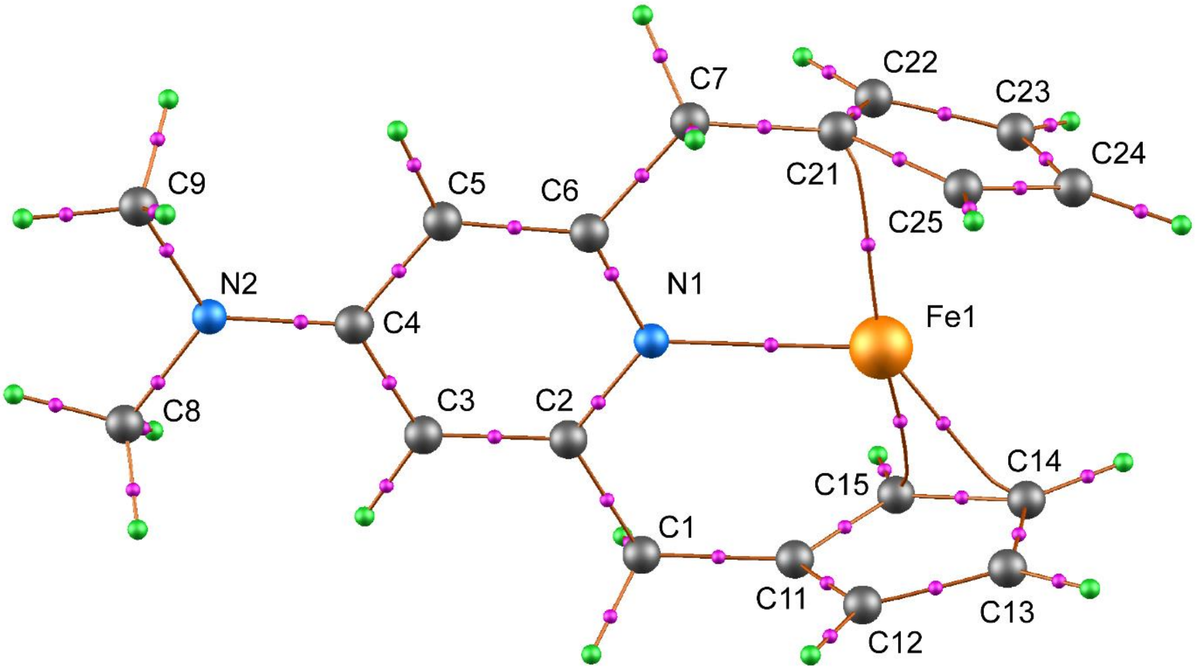

Supplementary Fig. 69. Molecular graph of the 20-electron ferrocene derivative **2-NMe₂**, based on theoretical data. The critical points (3, –1) in $\rho(\mathbf{r})$ are depicted as magenta spheres, while the corresponding internuclear bond paths are represented by brown lines.

Supplementary Table 9. Selected Properties of the Interatomic Contacts within the First Coordination Sphere in the 18-Electron Ferrocene Derivative **1-NMe2**, Based on Theoretical Data [a]

contact	ρ-path	φ_k-path	φ_{es}-path	$d(S–U)$, Å	$d(S–P)$, Å	barrier height	δ(Fe,X)
Fe1···N2	N	N	N	0.183	0.020	–0.407	0.053
Fe1···C11	N	Y	Y	0.106	0.074	–1.032	0.412
Fe1···C12	N	Y	Y	0.103	0.079	–1.082	0.476
Fe1–C13	Y	Y	Y	0.101	0.078	–1.116	0.498
Fe1–C14	Y	Y	Y	0.102	0.078	–1.110	0.493
Fe1···C15	N	Y	Y	0.103	0.079	–1.072	0.459
Fe1···C21	N	Y	Y	0.105	0.074	–1.042	0.418
Fe1–C22	Y	Y	Y	0.103	0.079	–1.088	0.479
Fe1–C23	Y	Y	Y	0.101	0.078	–1.118	0.498
Fe1–C24	Y	Y	Y	0.101	0.078	–1.113	0.497
Fe1–C25	Y	Y	Y	0.103	0.079	–1.081	0.471

[a] The letters "Y" and "N" indicate the presence and absence of an internuclear gradient path for a contact, respectively. The distance $d(S–U)$ represents the distance (in Å) between the minima in $\rho(\mathbf{r})$ and $\varphi_{es}(\mathbf{r})$, measured along a straight internuclear line for each contact. Similarly, $d(S-P)$ indicates the distance (in Å) between the minima in $\rho(\mathbf{r})$ and $\varphi_k(\mathbf{r})$, measured along the same line. The height of the total static potential barrier for an electron [in atomic units (a.u.)] was determined along a straight internuclear line for each contact. The delocalization index is denoted by δ(Fe, X) and given in atomic units (a.u.).

Supplementary Table 10. Selected Properties of the Interatomic Contacts within the First Coordination Sphere in the 20-Electron Ferrocene Derivative **2-NMe2**, Based on Theoretical Data [a]

contact	ρ-path	φ_k-path	φ_{es}-path	$d(S–U)$, Å	$d(S–P)$, Å	barrier height	δ(Fe,X)
Fe1–N2	Y	Y	Y	0.208	0.098	–0.917	0.364
Fe1···C11	N	N	Y	0.154	0.083	–0.827	0.212
Fe1···C12	N	N	N	0.197	0.123	–0.767	0.195
Fe1···C13	N	N	N	0.181	0.109	–0.792	0.215
Fe1–C14	Y	N	Y	0.137	0.080	–0.879	0.277
Fe1–C15	Y	Y	Y	0.146	0.090	–0.893	0.297
Fe1–C21	Y	Y	Y	0.135	0.079	–0.894	0.267
Fe1···C22	N	N	N	0.193	0.109	–0.789	0.215
Fe1···C23	N	N	N	0.201	0.126	–0.738	0.176
Fe1···C24	N	N	N	0.181	0.097	–0.794	0.215
Fe1···C25	N	Y	Y	0.146	0.109	–0.889	0.304

[a] The letters "Y" and "N" indicate the presence and absence of an internuclear gradient path for a contact, respectively. The distance $d(S–U)$ represents the distance (in Å) between the minima in $\rho(\mathbf{r})$ and $\varphi_{es}(\mathbf{r})$, measured along a straight internuclear line for each contact. Similarly, $d(S-P)$ indicates the distance (in Å) between the minima in $\rho(\mathbf{r})$ and $\varphi_k(\mathbf{r})$, measured along the same line. The height of the total static potential barrier for an electron [in atomic units (a.u.)] was determined along a straight internuclear line for each contact. The delocalization index is denoted by δ(Fe, X) and given in atomic units (a.u.).

Supplementary Table 11. Comparison of the Selected Properties of the Interatomic Contacts within the First Coordination Spheres in the Ferrocene Derivatives **1-NMe2** and **2-NMe2**, and Their Cobalt(II) Analogues Bearing the H-CpNCp Ligand [a]

property	**1-NMe2**	**2-NMe2**	**1-Co**	**2-Co**
barrier height (M,N)	–0.407	–0.917	–0.434	–0.876
barrier height (M,C)	–1.075(43)	–0.816(78)	–0.995(56)	–0.867(14)
δ(M,N)	0.053	0.364	0.073	0.363
δ(M,C)	0.455(43)	0.240(64)	0.366(100)	0.257(20)

[a] The height of the total static potential barrier for an electron (a.u.) was determined along a straight internuclear line for each contact. The delocalization index (a.u.) is denoted by δ.

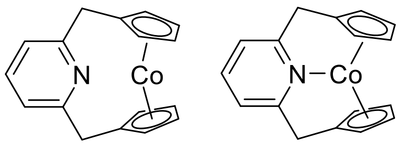

1-Co **2-Co**

Supplementary Note 7. SC-XRD study

Crystal structure determination

X-ray diffraction experiments for **1-H**, the cocrystal of **1-OMe** and **2-OMe**, **Co-NMe$_2$**, **[Co-NMe$_2$]BF$_4$**, and **Ru-NMe$_2$** were performed using a Bruker D8 Venture diffractometer equipped with a PHOTON II CPAD detector, an IμS 3.0 microfocus X-ray source (Mo *K*α radiation), and an Oxford Cryostream LT device. The data were collected at 100(2) K in φ/ω-scan mode in accordance with the recommended strategies. In each case, the final cell constants were determined through a global refinement of the reflections from the complete data set. The images were indexed and integrated using the *APEX*4 data reduction package. The data were corrected for systematic errors and absorption using a numerical absorption correction based on a multifaceted crystal model and/or an empirical absorption correction based on spherical harmonics in accordance with the point group symmetry, using equivalent reflections. *XPREP*-2014/2 was employed to analyze systematic absences and determine the space group.

X-ray diffraction data for the single crystals **1-Cl**, **2-MeO**, **2-NMe$_2$**, **3-H**, **3-Cl**, **3-OMe**, **3-NMe$_2$**, **4-H**, **4-NMe$_2$**, and **4-NMe$_2$-PF$_6$** were collected using a Rigaku XtaLab PRO instrument in ω-scan mode at 100(2) K. The diffractometer was equipped with a PILATUS3 R 200K hybrid pixel array detector and a MicroMax^{TM}-003 microfocus X-ray tube (Mo *K*α radiation). The images were indexed and integrated using the *CrysAlisPro* data reduction package. In each case, the final cell constants were determined by a global refinement of the reflections from the entire data set. The data were corrected for systematic errors and absorption using the *ABSPACK* module, which provides a numerical absorption correction based on Gaussian integration over a multifaceted crystal model and/or an empirical absorption correction based on spherical harmonics, according to the point group symmetry, using equivalent reflections. The *GRAL* module was utilized to analyze systematic absences and determine the subsequent space-group.

All structures were solved by the intrinsic phasing approach using *SHELXT*-2018/2[34] and subsequently refined by the full-matrix least-squares method on F^2 using *SHELXL*-2018/3[35]. The nonhydrogen atoms were refined anisotropically. The positions of the hydrogen atoms of methyl groups were determined through a rotating group refinement, taking into account the idealized tetrahedral angles. The remaining hydrogen atoms were introduced at the calculated positions and refined as riding atoms. The crystal structure of **1-Cl** was refined as a 2-component twin against a combined set of diffraction indices (HKLF 5). The unit cell of **4-NMe$_2$** contains highly disordered solvent molecules, which were treated as a diffuse contribution to the overall scattering without specific atom positions by PLATON/SQUEEZE[36]. Information regarding the “squeezed” solvent is not included in the formulae, and related items, such as molecular weights and calculated densities, are not included. Any disorder present was resolved using free variables and reasonable restraints on geometry and anisotropic displacement parameters. No anomalous bond lengths or angles were observed in the compounds under investigation.

Detailed crystallographic and structural parameters can be found in the provided CIF files. Deposition numbers CCDC 2380049 (**1-H**), 2380050 (**1-Cl**), 2380051 (cocrystal of **1-OMe** and **2-OMe**), 2380052 (**2-OMe**), 2380053 (**2-NMe$_2$**), 2380054 (**3-H**), 2380055 (**3-Cl**), 2380056 (**3-OMe**), 2380057 (**3-NMe$_2$**), 2380058 (**4-H**), 2380059 (**4-NMe$_2$**), 2406042 (**4-NMe$_2$-PF$_6$**), 2446847 (**Co-NMe$_2$**), 2446848 (**[Co-NMe$_2$]BF$_4$**), and 2446849 (**Ru-NMe$_2$**) contain supplementary crystallographic data for this paper. These data are provided free of charge by the joint Cambridge Crystallographic Data Centre and Fachinformationszentrum Karlsruhe Access Structures service (www.ccdc.cam.ac.uk/structures).

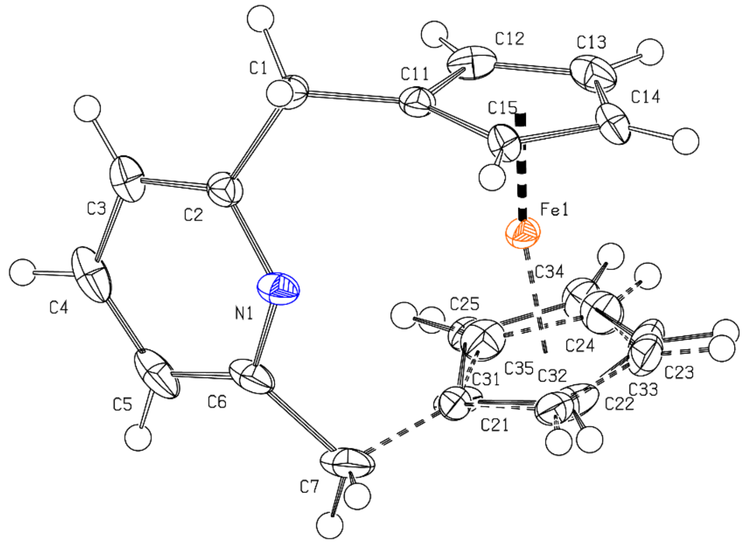

Supplementary Fig. 70. Oak Ridge Thermal-Ellipsoid Plot (ORTEP) showing the anisotropic displacement ellipsoids of non-hydrogen atoms for compound **1-H** at the 50 % probability level, as derived from the single-crystal X-ray diffraction (SC-XRD) data. The minor disorder component is shown by dashed, non-bold lines. Selected interatomic distances [Å]: Fe1–C11 2.1787(8), Fe1–C12 2.0885(9), Fe1–C13 2.0318(9), Fe1–C14 2.0433(8), Fe1–C15 2.1024(8), Fe1–C21 2.154(4), Fe1–C22 2.075(3), Fe1–C23 2.054(3), Fe1–C24 2.082(4), Fe1–C25 2.104(5), Fe1–C31 2.183(6), Fe1–C32 2.137(5), Fe1–C33 2.035(5), Fe1–C34 1.980(5), Fe1–C35 2.041(11).

Crystallographic data for **1-H**.
$C_{17}H_{15}FeN$, orange plate (0.289 × 0.241 × 0.038 mm^3), formula weight 289.15 g mol^{-1}; monoclinic, $P2_1/n$ (No. 14), a = 8.0729(2) Å, b = 5.8471(2) Å, c = 26.6860(8) Å, β = 91.3384(7)°, V = 1259.32(7) Å^3, Z = 4, Z' = 1, T = 100(2) K, d_{calc} = 1.525 g cm^{-3}, μ(Mo Kα) = 1.180 mm^{-1}, F(000) = 600; T_{max}/T_{min} = 0.7411/0.5567; 138858 reflections were collected (2.620° ≤ θ ≤ 47.397°, index ranges: –16 ≤ h ≤ 16, –11 ≤ k ≤ 12, and –54 ≤ l ≤ 54), 11608 of which were unique, R_{int} = 0.0613, $R_σ$ = 0.0317; completeness to θ of 47.397° 99.1 %. The refinement of 218 parameters with 335 restraints converged to $R1$ = 0.0481 and $wR2$ = 0.1238 for 10188 reflections with $I > 2σ(I)$ and $R1$ = 0.0542 and $wR2$ = 0.1285 for all data with goodness-of-fit S = 1.057 and residual electron density $ρ_{max}/ρ_{min}$ = 2.725/–1.406 e Å^{-3}, rms 0.102 e Å^{-3}; max shift/e.s.d. in the last cycle 0.002.

```
Alert level B
PLAT097_ALERT_2_B Large Reported Max.  (Positive) Residual Density       2.72 eA-3
```

Author Response: The Q-peak is close to the heavy atom Fe1.

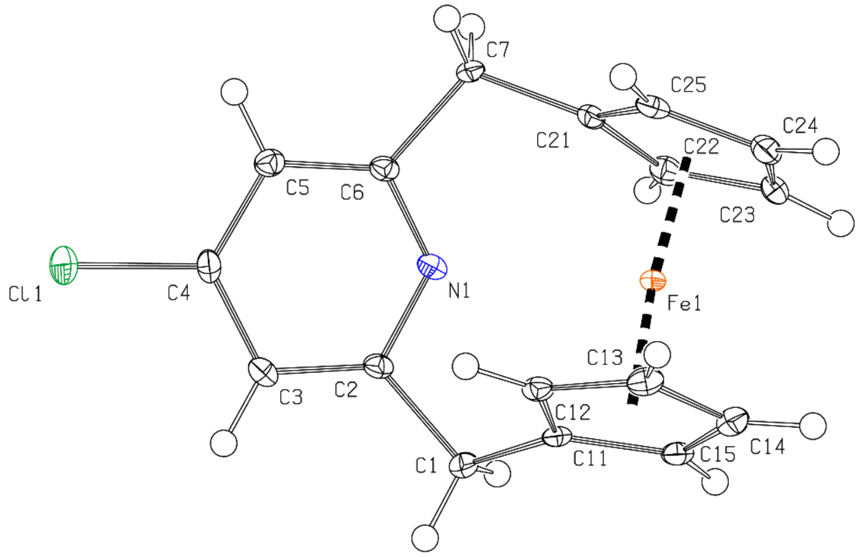

Supplementary Fig. 71. ORTEP showing the anisotropic displacement ellipsoids of non-hydrogen atoms for compound **1-Cl** at the 50 % probability level, as derived from the SC-XRD data. Selected interatomic distances [Å]: Fe1–C11 2.145(3), Fe1–C12 2.091(3), Fe1–C13 2.051(3), Fe1–C14 2.036(3), Fe1–C15 2.074(3), Fe1–C21 2.152(3), Fe1–C22 2.093(3), Fe1–C23 2.039(3), Fe1–C24 2.036(3), Fe1–C25 2.082(3).

Crystallographic data for **1-Cl**.
$C_{17}H_{14}ClFeN$, orange prism (0.194 × 0.092 × 0.069 mm^3), formula weight 323.59 g mol^{-1}; monoclinic, $P2_1/c$ (No. 14), a = 8.5448(2) Å, b = 10.1469(3) Å, c = 15.6951(4) Å, β = 97.395(3)°, V = 1349.50(6) $Å^3$, Z = 4, Z' = 1, T = 100(2) K, d_{calc} = 1.593 g cm^{-3}, μ(Mo $K\alpha$) = 1.302 mm^{-1}, $F(000)$ = 664; T_{max}/T_{min} = 1.00000/0.56934; 7250 reflections were collected (3.132° ≤ θ ≤ 32.104°, index ranges: –12 ≤ h ≤ 12, –14 ≤ k ≤ 14, and –18 ≤ l ≤ 23), 7250 of which were unique, R_σ = 0.0328; completeness to θ of 32.104° 92.6 %. The refinement of 182 parameters with no restraints converged to $R1$ = 0.0470 and $wR2$ = 0.1674 for 6493 reflections with $I > 2\sigma(I)$ and $R1$ = 0.0520 and $wR2$ = 0.1755 for all data with goodness-of-fit S = 1.060 and residual electron density ρ_{max}/ρ_{min} = 0.721/–0.565 e $Å^{-3}$, rms 0.161 e $Å^{-3}$; max shift/e.s.d. in the last cycle 0.001.

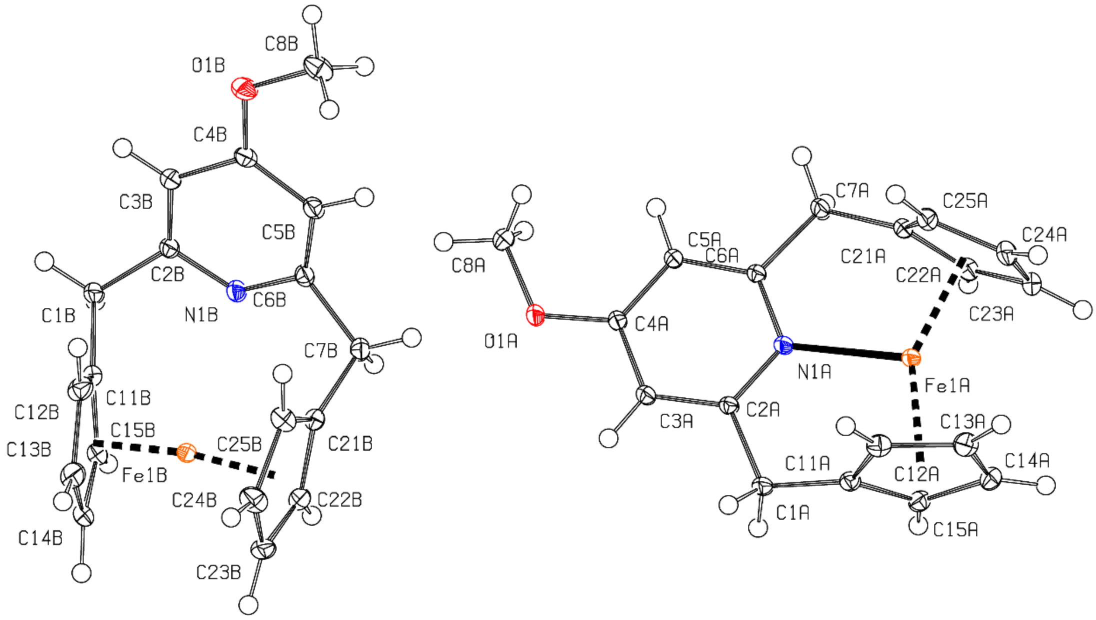

Supplementary Fig. 72. ORTEP showing the anisotropic displacement ellipsoids of non-hydrogen atoms for the cocrystal of **1-OMe** and **2-OMe** at the 50 % probability level, as derived from the SC-XRD data. Selected interatomic distances [Å]: Fe1A–N1A 2.1870(6), Fe1A–C11A 2.3114(7), Fe1A–C12A 2.3344(8), Fe1A–C13A 2.3979(8), Fe1A–C14A 2.4073(8), Fe1A–C15A 2.3528(7), Fe1A–C21A 2.3021(7), Fe1A–C22A 2.3367(8), Fe1A–C23A 2.3989(8), Fe1A–C24A 2.3938(8), Fe1A–C25A 2.3293(8), Fe1B–C11B 2.1740(7), Fe1B–C12B 2.0858(8), Fe1B–C13B 2.0273(8), Fe1B–C14B 2.0392(8), Fe1B–C15B 2.0992(8), Fe1B–C21B 2.1583(7), Fe1B–C22B 2.0910(8), Fe1B–C23B 2.0416(8), Fe1B–C24B 2.0366(8), Fe1B–C25B 2.0811(8).

Crystallographic data for the cocrystal of **1-OMe** and **2-OMe**.

$C_{18}H_{17}FeNO$, plate (0.305 × 0.261 × 0.044 mm^3), formula weight 319.17 g mol^{-1}; triclinic, $P\bar{1}$ (No. 2), a = 9.0350(3) Å, b = 10.5010(3) Å, c = 15.7739(5) Å, α = 73.1307(6)°, β = 80.0254(5)°, γ = 76.3633(5)°, V = 1382.94(7) Å^3, Z = 4, Z' = 1 + 1, T = 100(2) K, d_{calc} = 1.533 g cm^{-3}, μ(Mo Kα) = 1.087 mm^{-1}, F(000) = 664; T_{max}/T_{min} = 0.8486/0.6764; 290145 reflections were collected (2.334° ≤ θ ≤ 41.716°, index ranges: –16 ≤ h ≤ 16, –18 ≤ k ≤ 19, and –28 ≤ l ≤ 29), 18688 of which were unique, R_{int} = 0.0406, $R_σ$ = 0.0212; completeness to θ of 41.716° 98.2 %. The refinement of 381 parameters with no restraints converged to $R1$ = 0.0364 and $wR2$ = 0.0947 for 16463 reflections with $I > 2σ(I)$ and $R1$ = 0.0422 and $wR2$ = 0.0977 for all data with goodness-of-fit S = 1.080 and residual electron density $ρ_{max}/ρ_{min}$ = 1.408/–0.508 e Å^{-3}, rms 0.100 e Å^{-3}; max shift/e.s.d. in the last cycle 0.003.

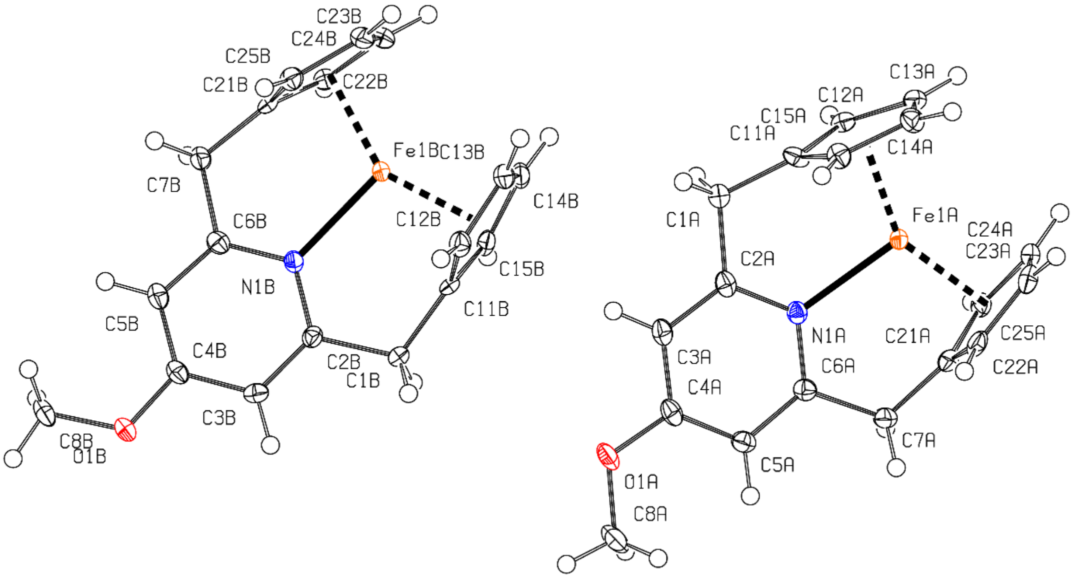

Supplementary Fig. 73. ORTEP showing the anisotropic displacement ellipsoids of non-hydrogen atoms for compound **2-OMe** at the 50 % probability level, as derived from the SC-XRD data. Selected interatomic distances [Å]: Fe1A–N1A 2.170(2), Fe1A–C11A 2.284(3), Fe1A–C12A 2.324(2), Fe1A–C13A 2.407(3), Fe1A–C14A 2.429(3), Fe1A–C15A 2.348(3), Fe1A–C21A 2.315(3), Fe1A–C22A 2.360(3), Fe1A–C23A 2.414(3), Fe1A–C24A 2.378(3), Fe1A–C25A 2.315(3), Fe1B–N1B 2.166(2), Fe1B–C11B 2.306(2), Fe1B–C12B 2.359(3), Fe1B–C13B 2.424(3), Fe1B–C14B 2.398(3), Fe1B–C15B 2.312(3), Fe1B–C21B 2.305(2), Fe1B–C22B 2.322(3), Fe1B–C23B 2.391(3), Fe1B–C24B 2.415(3), Fe1B–C25B 2.361(3).

Crystallographic data for **2-MeO**.

$C_{18}H_{17}FeNO$, colorless plate (0.075 × 0.054 × 0.020 mm^3), formula weight 319.17 g mol^{-1}; monoclinic, $P2_1/n$ (No. 14), a = 11.9433(8) Å, b = 13.3548(4) Å, c = 24.5444(17) Å, β = 133.700(12)°, V = 2830.3(5) Å^3, Z = 8, Z' = 2, T = 100(2) K, d_{calc} = 1.498 g cm^{-3}, μ(Mo Kα) = 1.063 mm^{-1}, F(000) = 1328; T_{max}/T_{min} = 1.000/0.877; 49881 reflections were collected (3.051° ≤ θ ≤ 27.913°, index ranges: $-15 \leq h \leq 15$, $-17 \leq k \leq 17$, and $-32 \leq l \leq 32$), 6707 of which were unique, R_{int} = 0.0497, R_σ = 0.0319; completeness to θ of 27.913° 98.9 %. The refinement of 375 parameters with no restraints converged to $R1$ = 0.0424 and $wR2$ = 0.0901 for 5178 reflections with $I > 2\sigma(I)$ and $R1$ = 0.0591 and $wR2$ = 0.0948 for all data with goodness-of-fit S = 1.146 and residual electron density ρ_{max}/ρ_{min} = 0.900/–0.500 e Å^{-3}, rms 0.079 e Å^{-3}; max shift/e.s.d. in the last cycle 0.001.

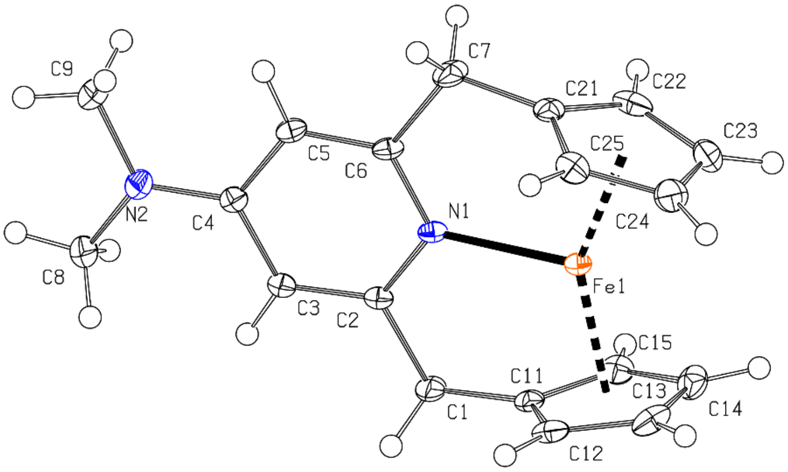

Supplementary Fig. 74. ORTEP showing the anisotropic displacement ellipsoids of non-hydrogen atoms for compound **2-NMe2** at the 50 % probability level, as derived from the SC-XRD data. Selected interatomic distances [Å]: Fe1–N1 2.1476(10), Fe1–C11 2.3170(11), Fe1–C12 2.3511(12), Fe1–C13 2.4157(12), Fe1–C14 2.4139(12), Fe1–C15 2.3467(11), Fe1–C21 2.3102(11), Fe1–C22 2.3414(12), Fe1–C23 2.4171(13), Fe1–C24 2.4197(12), Fe1–C25 2.3445(11).

Crystallographic data for **2-NMe2**.

$C_{19}H_{20}FeN_2$, colorless plate (0.092 × 0.067 × 0.017 mm^3), formula weight 332.22 g mol^{-1}; monoclinic, $P2_1/c$ (No. 14), a = 8.00094(16) Å, b = 8.25560(18) Å, c = 23.2469(5) Å, β = 92.6916(18)°, V = 1533.82(6) Å^3, Z = 4, Z' = 1, T = 100(2) K, d_{calc} = 1.439 g cm^{-3}, μ(Mo $K\alpha$) = 0.981 mm^{-1}, F(000) = 696; T_{max}/T_{min} = 1.000/0.806; 37135 reflections were collected (2.618° ≤ θ ≤ 32.645°, index ranges: –9 ≤ h ≤ 12, –12 ≤ k ≤ 12, and –34 ≤ l ≤ 32), 5308 of which were unique, R_{int} = 0.0306, R_σ = 0.0214; completeness to θ of 32.645° 94.3 %. The refinement of 201 parameters with no restraints converged to $R1$ = 0.0307 and $wR2$ = 0.0779 for 4614 reflections with $I > 2\sigma(I)$ and $R1$ = 0.0380 and $wR2$ = 0.0811 for all data with goodness-of-fit S = 1.035 and residual electron density ρ_{max}/ρ_{min} = 0.549/–0.256 e Å^{-3}, rms 0.066 e Å^{-3}; max shift/e.s.d. in the last cycle 0.003.

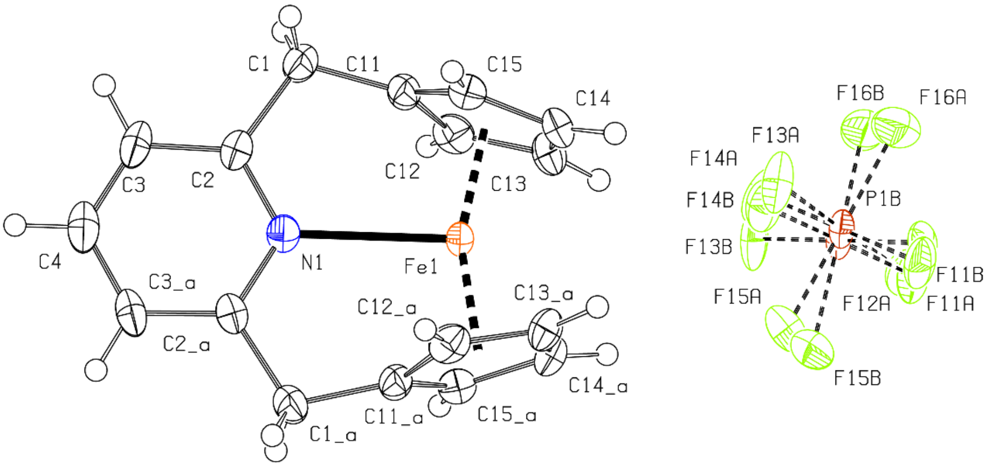

Supplementary Fig. 75. ORTEP showing the anisotropic displacement ellipsoids of non-hydrogen atoms for compound **3-H** at the 50 % probability level, as derived from the SC-XRD data. The minor disorder component is shown by dashed, non-bold lines. Selected interatomic distances [Å]: Fe1–N1 2.449(3), Fe1–C11 2.2239(18), Fe1–C12 2.1308(17), Fe1–C13 2.061(2), Fe1–C14 2.066(2), Fe1–C15 2.1448(18).

Crystallographic data for **3-H**.
$C_{17}H_{15}F_6FeNP$, brown plank (0.507 × 0.065 × 0.041 mm^3), formula weight 434.12 g mol^{-1}; orthorhombic, *Fdd*2 (No. 43), a = 11.7965(2) Å, b = 18.5303(4) Å, c = 14.7847(3) Å, V = 3231.83(11) Å^3, Z = 8, Z' = 0.5, T = 100(2) K, d_{calc} = 1.784 g cm^{-3}, μ(Mo *K*α) = 1.097 mm^{-1}, $F(000)$ = 1752; T_{max}/T_{min} = 1.000/0.499; 20685 reflections were collected (2.467° ≤ θ ≤ 30.009°, index ranges: $-16 \leq h \leq 15$, $-25 \leq k \leq 25$, and $-20 \leq l \leq 19$), 2166 of which were unique, R_{int} = 0.0296, R_{σ} = 0.0138; completeness to θ of 30.009° 94.7 %. The refinement of 209 parameters with 640 restraints converged to $R1$ = 0.0211 and $wR2$ = 0.0552 for 2125 reflections with $I > 2\sigma(I)$ and $R1$ = 0.0216 and $wR2$ = 0.0554 for all data with goodness-of-fit S = 1.035 and residual electron density ρ_{max}/ρ_{min} = 0.341/–0.197 e Å^{-3}, rms 0.042 e Å^{-3}; max shift/e.s.d. in the last cycle 0.000.

Alert level B

```
PLAT090_ALERT_3_B Poor Data / Parameter Ratio (Zmax > 18) ........       5.56 Note
```

Author Response: The resolution, redundancy, completeness, and quality of the data appear satisfactory for structure determination. Reasonable restraints were used to refine the disorder. Hydrogen atoms were refined in the riding model.

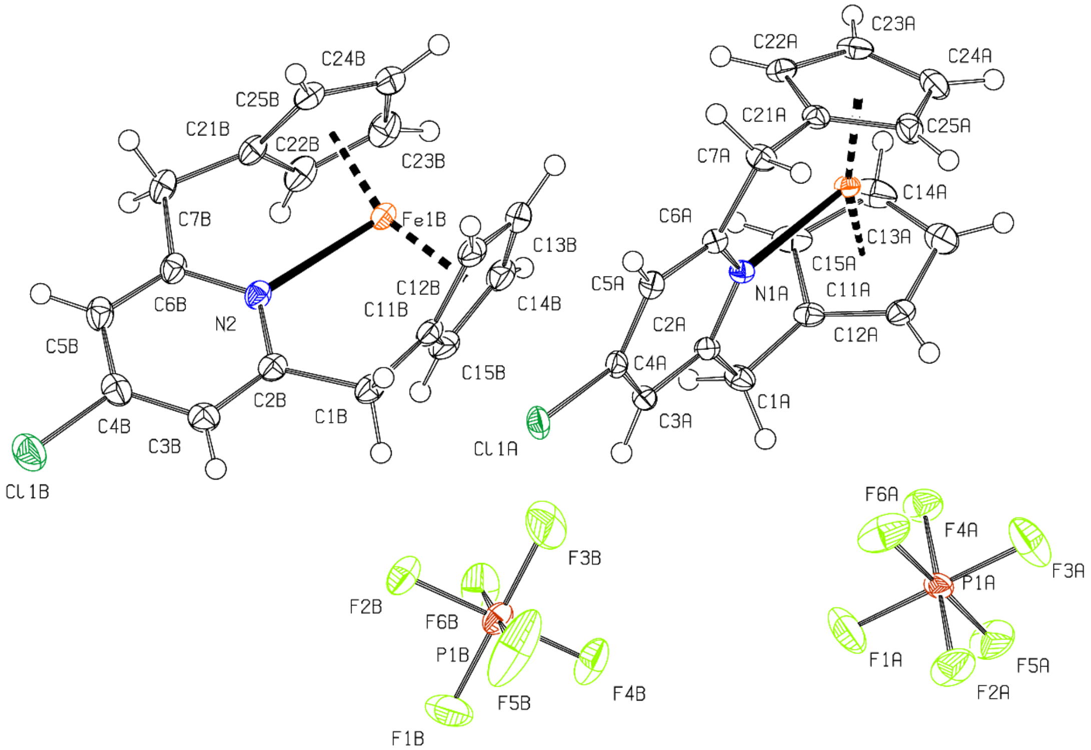

Supplementary Fig.76. ORTEP showing the anisotropic displacement ellipsoids of non-hydrogen atoms for compound **3-Cl** at the 50 % probability level, as derived from the SC-XRD data. Selected interatomic distances [Å]: Fe1A–N1A 2.4160(10), Fe1A–C11A 2.2421(11), Fe1A–C12A 2.1375(12), Fe1A–C13A 2.0569(13), Fe1A–C14A 2.0655(13), Fe1A–C15A 2.1552(13), Fe1A–C21A 2.2206(11), Fe1A–C22A 2.1384(12), Fe1A–C23A 2.0680(12), Fe1A–C24A 2.0735(13), Fe1A–C25A 2.1460(13), Fe1B–N2 2.4335(11), Fe1B–C11B 2.2194(12), Fe1B–C12B 2.1397(12), Fe1B–C13B 2.0724(13), Fe1B–C14B 2.0707(13), Fe1B–C15B 2.1381(12), Fe1B–C21B 2.2338(12), Fe1B–C22B 2.1427(13), Fe1B–C23B 2.0637(14), Fe1B–C24B 2.0628(13), Fe1B–C25B 2.1420(12).

Crystallographic data for **3-Cl**.

$C_{17}H_{14}ClF_6FeNP$, dark brown plank (0.278 × 0.099 × 0.068 mm^3), formula weight 468.56 g mol^{-1}; monoclinic, $P2_1/c$ (No. 14), a = 10.22686(11) Å, b = 37.8697(4) Å, c = 9.36176(11) Å, β = 110.3810(12)°, V = 3398.72(7) Å^3, Z = 8, Z' = 2, T = 100(2) K, d_{calc} = 1.831 g cm^{-3}, μ(Mo $K\alpha$) = 1.203 mm^{-1}, F(000) = 1880; T_{max}/T_{min} = 1.000/0.509; 244310 reflections were collected (2.558° ≤ θ ≤ 34.476°, index ranges: –16 ≤ h ≤ 16, –58 ≤ k ≤ 60, and –14 ≤ l ≤ 14), 13995 of which were unique, R_{int} = 0.0397, R_σ = 0.0155; completeness to θ of 34.476° 97.3 %. The refinement of 487 parameters with no restraints converged to $R1$ = 0.0340 and $wR2$ = 0.0808 for 12768 reflections with $I > 2\sigma(I)$ and $R1$ = 0.0381 and $wR2$ = 0.0824 for all data with goodness-of-fit S = 1.070 and residual electron density ρ_{max}/ρ_{min} = 0.816/–0.626 e Å^{-3}, rms 0.072 e Å^{-3}; max shift/e.s.d. in the last cycle 0.003.

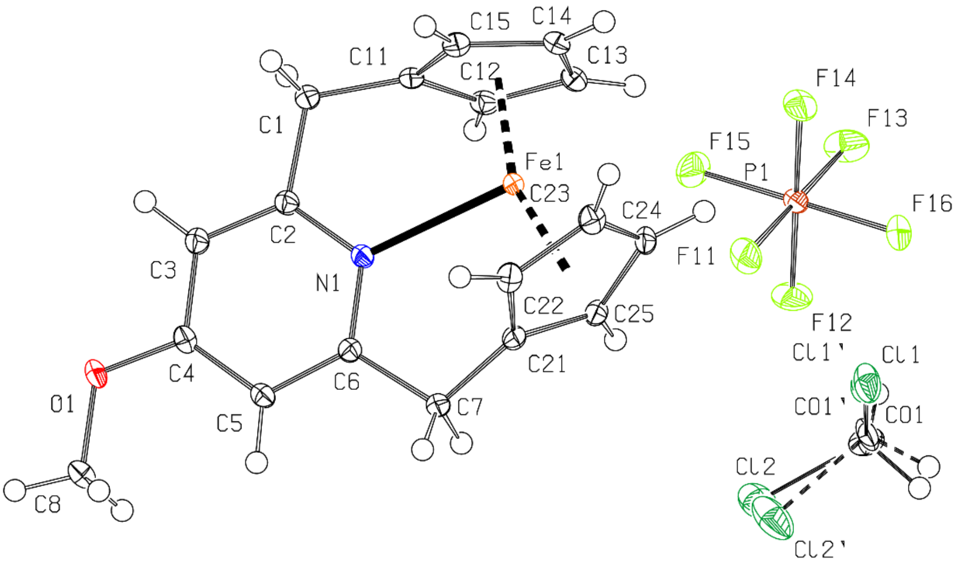

Supplementary Fig. 77. ORTEP showing the anisotropic displacement ellipsoids of non-hydrogen atoms for compound **3-MeO** at the 50 % probability level, as derived from the SC-XRD data. The minor disorder component is shown by dashed, non-bold lines. Selected interatomic distances [Å]: Fe1–N1 2.3972(10), Fe1–C11 2.2219(12), Fe1–C12 2.1392(12), Fe1–C13 2.0754(12), Fe1–C14 2.0736(12), Fe1–C15 2.1445(13), Fe1–C21 2.2172(12), Fe1–C22 2.1257(12), Fe1–C23 2.0679(12), Fe1–C24 2.0770(12), Fe1–C25 2.1590(12).

Crystallographic data for **3-OMe**.

$C_{19}H_{19}Cl_2F_6FeNOP$, yellow plank (0.152 × 0.118 × 0.055 mm^3), formula weight 549.07 g mol^{-1}; triclinic, $P\overline{1}$ (No. 2), a = 9.37232(19) Å, b = 10.5196(2) Å, c = 11.2599(2) Å, α = 100.7472(19)°, β = 105.5663(18)°, γ = 94.7978(18)°, V = 1040.14(4) Å^3, Z = 2, Z' = 1, T = 100(2) K, d_{calc} = 1.753 g cm^{-3}, μ(Mo $K\alpha$) = 1.124 mm^{-1}, F(000) = 554; T_{max}/T_{min} = 1.000/0.670; 29879 reflections were collected (2.520° ≤ θ ≤ 34.224°, index ranges: –14 ≤ h ≤ 14, –15 ≤ k ≤ 16, and –17 ≤ l ≤ 16), 7828 of which were unique, R_{int} = 0.0310, R_σ = 0.0344; completeness to θ of 34.224° 90.7 %. The refinement of 309 parameters with 87 restraints converged to $R1$ = 0.0311 and $wR2$ = 0.0697 for 6513 reflections with $I > 2\sigma(I)$ and $R1$ = 0.0422 and $wR2$ = 0.0731 for all data with goodness-of-fit S = 1.037 and residual electron density ρ_{max}/ρ_{min} = 0.559/–0.322 e Å^{-3}, rms 0.073 e Å^{-3}; max shift/e.s.d. in the last cycle 0.001.

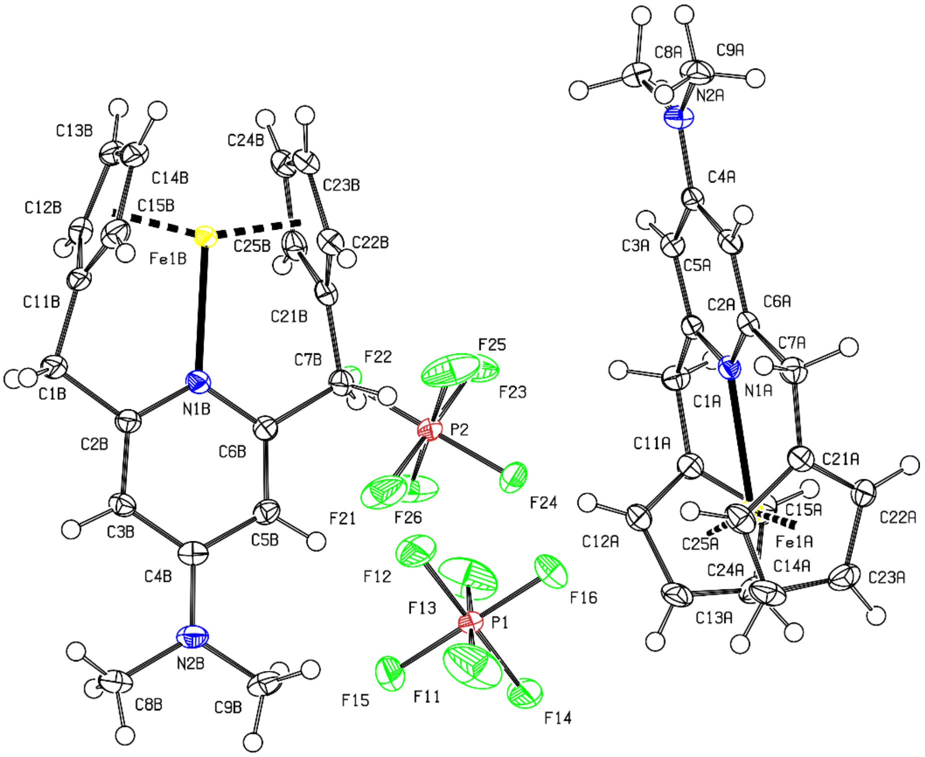

Supplementary Fig. 78. ORTEP showing the anisotropic displacement ellipsoids of non-hydrogen atoms for compound **3-NMe₂** at the 50 % probability level, as derived from the SC-XRD data. Selected interatomic distances [Å]: Fe1A–N1A 2.3785(16), Fe1A–C11A 2.227(2), Fe1A–C12A 2.1347(19), Fe1A–C13A 2.0656(19), Fe1A–C14A 2.061(2), Fe1A–C15A 2.150(2), Fe1A–C21A 2.2413(19), Fe1A–C22A 2.1519(19), Fe1A–C23A 2.063(2), Fe1A–C24A 2.063(2), Fe1A–C25A 2.149(2), Fe1B–N1B 2.3853(16), Fe1B–C11B 2.2314(19), Fe1B–C12B 2.150(2), Fe1B–C13B 2.0668(19), Fe1B–C14B 2.059(2), Fe1B–C15B 2.132(2), Fe1B–C21B 2.2300(19), Fe1B–C22B 2.148(2), Fe1B–C23B 2.068(2), Fe1B–C24B 2.0672(19), Fe1B–C25B 2.136(2).

Crystallographic data for **3-NMe₂**.

$C_{19}H_{20}F_6FeN_2P$, brown plank (0.327 × 0.074 × 0.057 mm^3), formula weight 477.19 g mol^{-1}; monoclinic, $P2_1/c$ (No. 14), a = 11.2509(3) Å, b = 16.8066(3) Å, c = 19.4121(4) Å, β = 97.1306(18)°, V = 3642.23(12) Å^3, Z = 8, Z' = 2, T = 100(2) K, d_{calc} = 1.740 g cm^{-3}, μ(Mo $K\alpha$) = 0.984 mm^{-1}, $F(000)$ = 1944; T_{max}/T_{min} = 1.000/0.580; 83326 reflections were collected (2.883° ≤ θ ≤ 30.062°, index ranges: –15 ≤ h ≤ 15, –23 ≤ k ≤ 23, and –27 ≤ l ≤ 26), 9815 of which were unique, R_{int} = 0.0442, R_σ = 0.0271; completeness to θ of 30.062° 91.9 %. The refinement of 527 parameters with no restraints converged to $R1$ = 0.0409 and $wR2$ = 0.1016 for 8403 reflections with $I > 2\sigma(I)$ and $R1$ = 0.0498 and $wR2$ = 0.1059 for all data with goodness-of-fit S = 1.036 and residual electron density ρ_{max}/ρ_{min} = 1.081/–0.769 e Å^{-3}, rms 0.090 e Å^{-3}; max shift/e.s.d. in the last cycle 0.001.

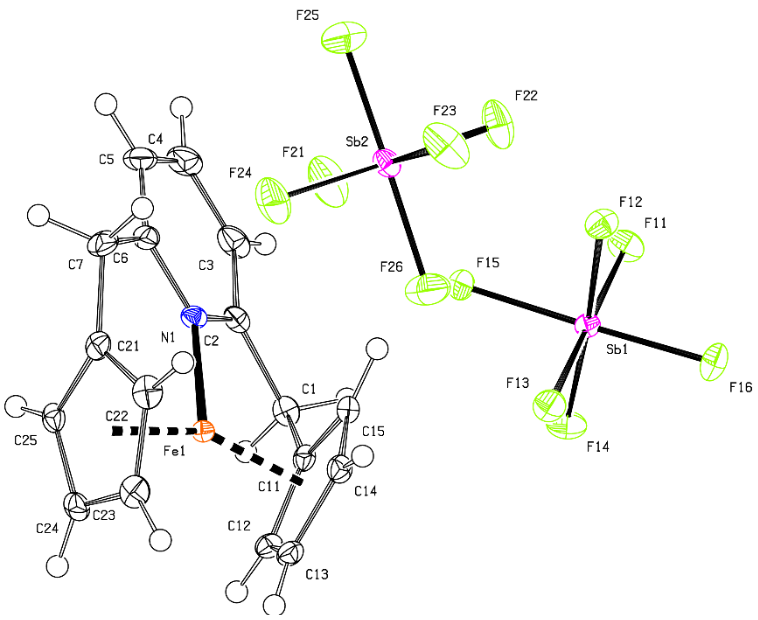

Supplementary Fig. 79. ORTEP showing the anisotropic displacement ellipsoids of non-hydrogen atoms for compound **4-H** at the 50 % probability level, as derived from the SC-XRD data. Selected interatomic distances [Å]: Fe1–N1 2.0437(14), Fe1–C11 2.0891(16), Fe1–C12 2.1285(16), Fe1–C13 2.1423(17), Fe1–C14 2.1375(17), Fe1–C15 2.0899(17), Fe1–C21 2.0864(17), Fe1–C22 2.1159(17), Fe1–C23 2.1321(18), Fe1–C24 2.1390(17), Fe1–C25 2.1112(17).

Crystallographic data for **4-H**.

$C_{17}H_{15}F_{12}FeNSb_2$, dark purple plate (0.303 × 0.075 × 0.036 mm^3), formula weight 760.65 g mol^{-1}; monoclinic, $P2_1/c$ (No. 14), a = 10.02230(18) Å, b = 15.7169(3) Å, c = 13.9886(3) Å, β = 103.3146(18)°, V = 2144.25(7) Å^3, Z = 4, Z' = 1, T = 100(2) K, d_{calc} = 2.356 g cm^{-3}, μ(Mo Kα) = 3.278 mm^{-1}, F(000) = 1440; T_{max}/T_{min} = 1.000/0.275; 48430 reflections were collected (2.993° ≤ θ ≤ 32.164°, index ranges: –14 ≤ h ≤ 14, –22 ≤ k ≤ 22, and –19 ≤ l ≤ 20), 6851 of which were unique, R_{int} = 0.0415, $R_σ$ = 0.0262; completeness to θ of 32.164° 90.9 %. The refinement of 299 parameters with no restraints converged to $R1$ = 0.0213 and $wR2$ = 0.0486 for 6266 reflections with $I > 2σ(I)$ and $R1$ = 0.0243 and $wR2$ = 0.0496 for all data with goodness-of-fit S = 1.020 and residual electron density $ρ_{max}/ρ_{min}$ = 0.751/–0.712 e Å^{-3}, rms 0.106 e Å^{-3}; max shift/e.s.d. in the last cycle 0.002.

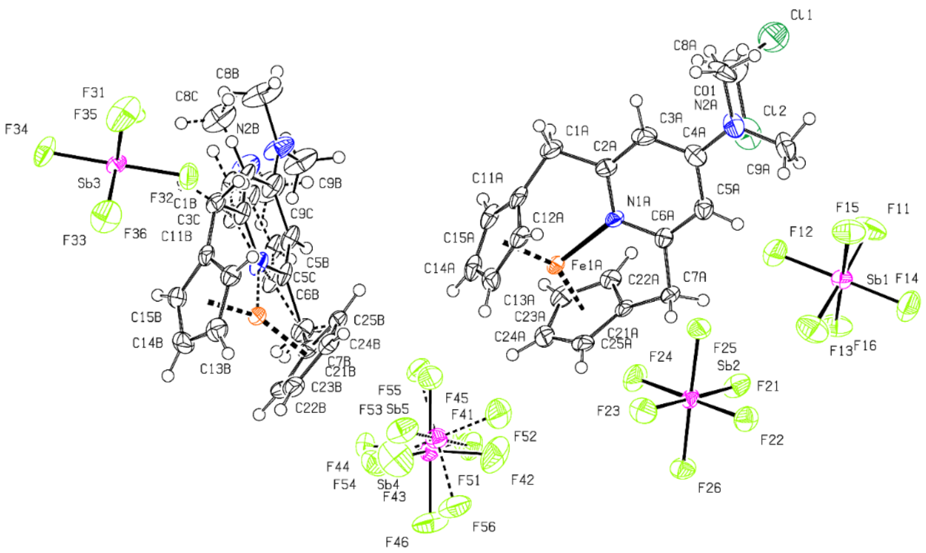

Supplementary Fig. 80. ORTEP showing the anisotropic displacement ellipsoids of non-hydrogen atoms for compound **4-NMe2** at the 50 % probability level, as derived from the SC-XRD data. The minor disorder components are shown by dashed, non-bold lines. Selected interatomic distances [Å]: Fe1A–N1A 2.029(5), Fe1A–C11A 2.086(6), Fe1A–C12A 2.095(6), Fe1A–C13A 2.127(6), Fe1A–C14A 2.116(6), Fe1A–C15A 2.123(6), Fe1A–C21A 2.095(5), Fe1A–C22A 2.101(6), Fe1A–C23A 2.138(6), Fe1A–C24A 2.130(6), Fe1A–C25A 2.121(6), Fe1B–N1B 2.033(6), Fe1B–N1C 2.024(10), Fe1B–C11B 2.101(6), Fe1B–C12B 2.122(6), Fe1B–C13B 2.127(7), Fe1B–C14B 2.136(7), Fe1B–C15B 2.097(6), Fe1B–C21B 2.089(6), Fe1B–C22B 2.124(6), Fe1B–C23B 2.130(7), Fe1B–C24B 2.142(7), Fe1B–C25B 2.104(6).

Crystallographic data for **4-NMe2**.

The unit cell contains highly disordered solvent molecules, which were treated as a diffuse contribution to the overall scattering without specific atom positions by PLATON/SQUEEZE. SQUEEZED solvent info is not included in the formula, and related items, such as molecular weight and calculated density, are not included. $C_{19.5}H_{21}ClF_{12}FeN_2Sb_2$, dark brown plank ($0.189 \times 0.137 \times 0.109$ mm^3), formula weight 846.18 g mol^{-1}; monoclinic, $P2_1/c$ (No. 14), a = 14.8950(3) Å, b = 16.0780(3) Å, c = 23.0988(4) Å, β = 93.3992(16)°, V = 5522.01(17) Å^3, Z = 8, Z' = 2, T = 100(2) K, d_{calc} = 2.036 g cm^{-3}, μ(Mo $K\alpha$) = 2.652 mm^{-1}, $F(000)$ = 3240; T_{max}/T_{min} = 1.000/0.572; 98384 reflections were collected (2.989° ≤ θ ≤ 27.950°, index ranges: $-19 \leq h \leq 19$, $-19 \leq k \leq 21$, and $-29 \leq l \leq 30$), 13155 of which were unique, R_{int} = 0.0352, R_σ = 0.0213; completeness to θ of 27.950° 99.1 %. The refinement of 828 parameters with 1264 restraints converged to $R1$ = 0.0565 and $wR2$ = 0.1313 for 12073 reflections with $I > 2\sigma(I)$ and $R1$ = 0.0618 and $wR2$ = 0.1335 for all data with goodness-of-fit S = 1.265 and residual electron density ρ_{max}/ρ_{min} = 3.598/–1.349 e Å^{-3}, rms 0.176 e Å^{-3}; max shift/e.s.d. in the last cycle 0.003.

Alert level B

```
PLAT910_ALERT_3_B Missing # of FCF Reflection(s) Below Theta(Min).        16 Note
                 1  0  0,    2  0  0,    1  1  0,    0  2  0,    1  2  0,   -1  1  1,
                 0  1  1,    1  1  1,    0  2  1,   -1  0  2,    0  0  2,    1  0  2,
                -1  1  2,    0  1  2,    1  1  2,    0  1  3,
PLAT971_ALERT_2_B Check Calcd Resid. Dens.   0.86Ang From Sb2           3.45 eA-3
```

Author Response: The Q-peaks are close to the heavy Sb atoms.

```
PLAT971_ALERT_2_B Check Calcd Resid. Dens.   0.81Ang From Sb3           2.64 eA-3
```

Author Response: The Q-peaks are close to the heavy Sb atoms.

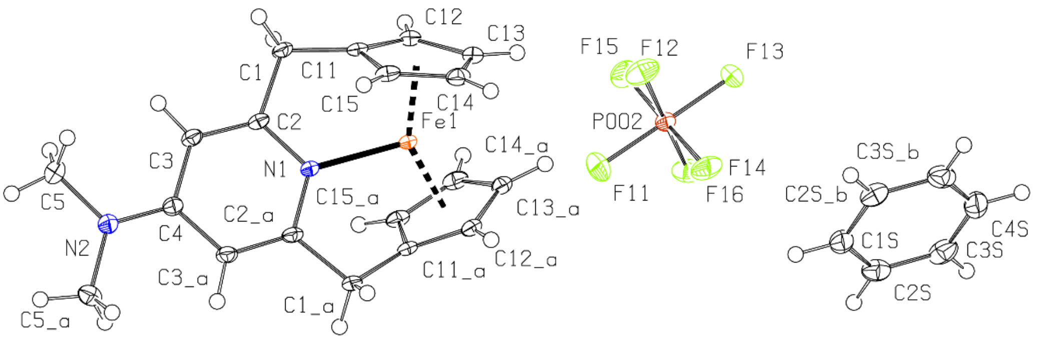

Supplementary Fig. 81. ORTEP showing the anisotropic displacement ellipsoids of non-hydrogen atoms for compound **4-NMe$_2$-PF$_6$** at the 50 % probability level, as derived from the SC-XRD data. Selected interatomic distances [Å]: Fe1–N1 2.030(2), Fe1–C11 2.0936(15), Fe1–C12 2.1259(15), Fe1–C13 2.1276(17), Fe1–C14 2.1283(17), Fe1–C15 2.1041(16). Symmetry-related atoms are indicated by _a and _b.

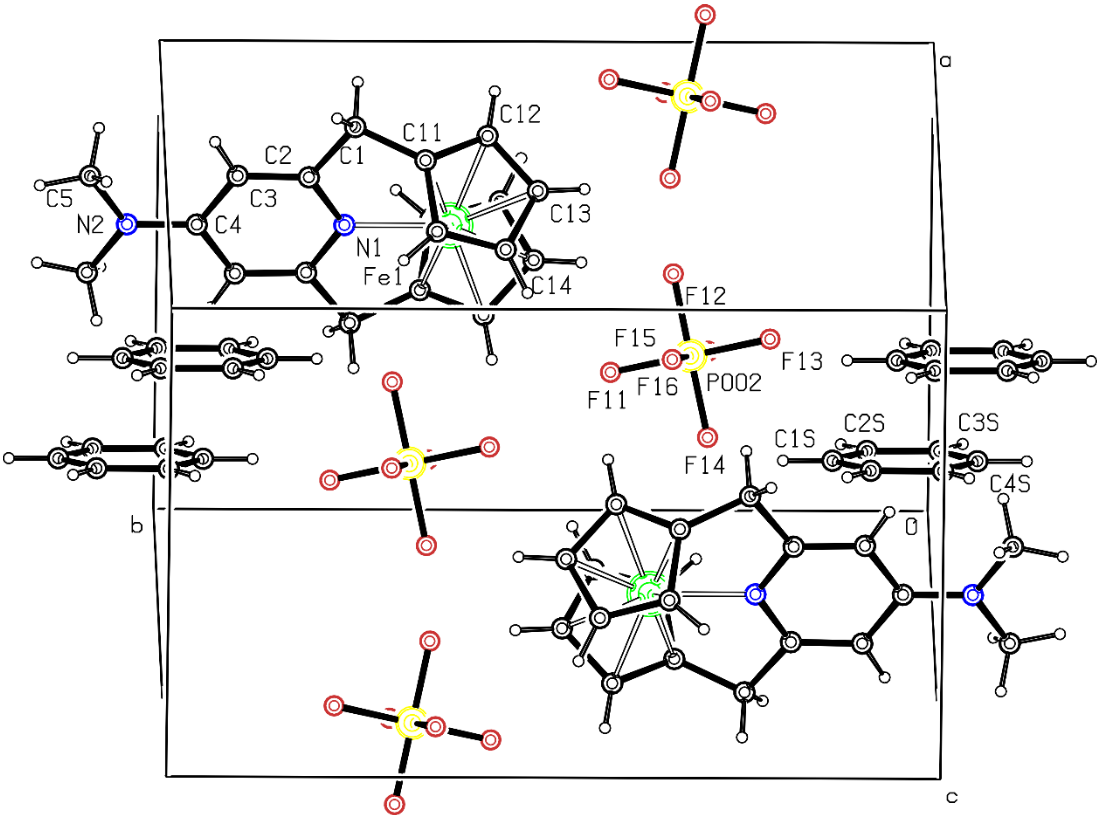

Supplementary Fig. 82. Crystal packing of **4-NMe$_2$-PF$_6$**.

Crystallographic data for **4-NMe$_2$-PF$_6$**.
$C_{25}H_{26}F_{12}FeN_2P_2$, black plank (0.141 × 0.107 × 0.056 mm^3), formula weight 700.27 g mol^{-1}; monoclinic, $P2/n$ (No. 13), a = 9.4079(3) Å, b = 14.8665(5) Å, c = 9.7030(3) Å, V = 1311.56(7) Å^3, Z = 2, Z' = 0.5, T = 100(2) K, d_{calc} = 1.773 g cm^{-3}, μ(Mo Kα) = 0.804 mm^{-1}, F(000) = 708; T_{max}/T_{min} = 1.000/0.623; 20360 reflections were collected (2.626° ≤ θ ≤ 31.984°, index ranges: $-13 \leq h \leq 13$, $-21 \leq k \leq 16$, and $-14 \leq l \leq 14$), 4020 of which were unique, R_{int} = 0.0501, R_σ = 0.0406; completeness to θ of 31.984° 88.2 %. The refinement of 194 parameters with no restraints converged to $R1$ = 0.0389 and $wR2$ = 0.1052 for 3512 reflections with $I > 2\sigma(I)$ and $R1$ = 0.0447 and $wR2$ = 0.1075 for all data with goodness-of-fit S = 1.075 and residual electron density ρ_{max}/ρ_{min} = 0.670/–0.415 e Å^{-3}, rms 0.095 e Å^{-3}; max shift/e.s.d. in the last cycle 0.000.

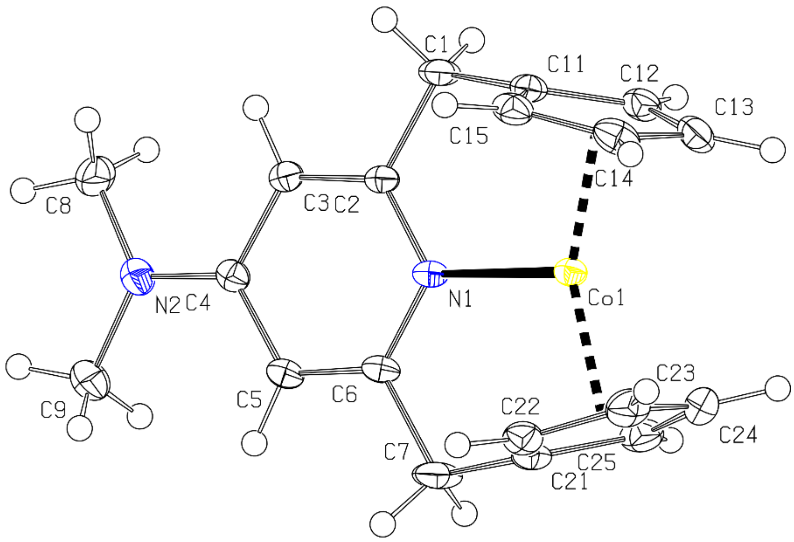

Supplementary Fig. 83. ORTEP showing the anisotropic displacement ellipsoids of non-hydrogen atoms for compound **Co-NMe$_2$** at the 50 % probability level, as derived from the SC-XRD data. Selected interatomic distances [Å]: Co1–N1 2.1860(12), Co1–C11 2.3265(13), Co1–C12 2.3201(13), Co1–C13 2.3058(14), Co1–C14 2.2955(14), Co1–C15 2.3030(13), Co1–C21 2.3231(13), Co1–C22 2.3094(13), Co1–C23 2.3025(15), Co1–C24 2.2974(15), Co1–C25 2.3048(14).

Crystallographic data for **Co-NMe$_2$**.

$C_{19}H_{20}CoN_2$, plate (0.153 × 0.126 × 0.018 mm^3), formula weight 335.30 g mol^{-1}; monoclinic, $P2_1/c$ (No. 14), a = 7.9776(4) Å, b = 8.2217(4) Å, c = 23.0717(11) Å, β = 92.5974(13)°, V = 1511.71(13) Å^3, Z = 4, Z' = 1, T = 100(2) K, d_{calc} = 1.473 g cm^{-3}, μ(Mo Kα) = 1.133 mm^{-1}, F(000) = 700; T_{max}/T_{min} = 0.9705/0.8498; 97746 reflections were collected (2.556° ≤ θ ≤ 34.334°, index ranges: –12 ≤ h ≤ 12, –12 ≤ k ≤ 13, and –36 ≤ l ≤ 36), 6284 of which were unique, R_{int} = 0.0429, $R_σ$ = 0.0215; completeness to θ of 34.334° 99.4 %. The refinement of 201 parameters with no restraints converged to $R1$ = 0.0419 and $wR2$ = 0.1025 for 5540 reflections with $I > 2σ(I)$ and $R1$ = 0.0482 and $wR2$ = 0.1055 for all data with goodness-of-fit S = 1.137 and residual electron density $ρ_{max}/ρ_{min}$ = 0.765/–0.610 e Å^{-3}, rms 0.088 e Å^{-3}; max shift/e.s.d. in the last cycle 0.002.

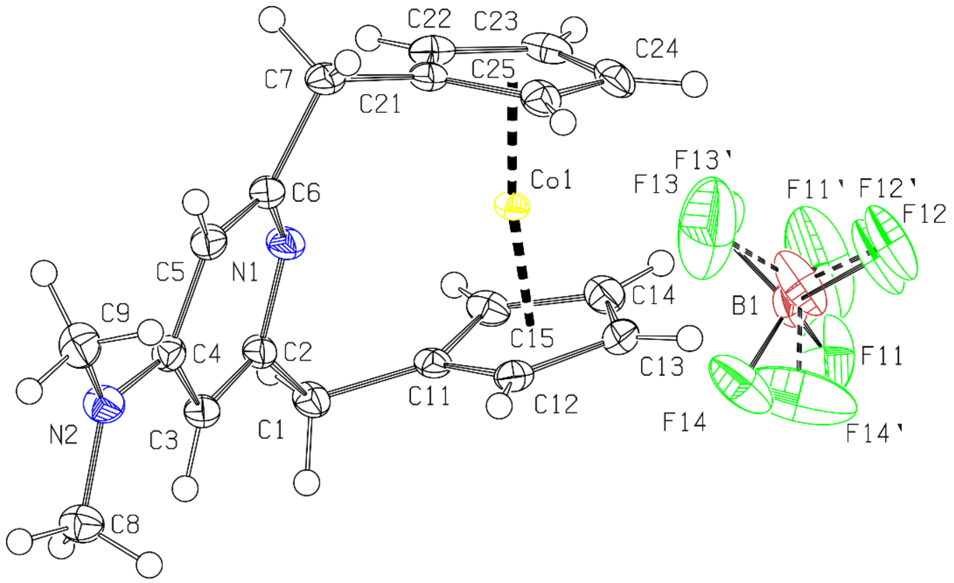

Supplementary Fig. 84. ORTEP showing the anisotropic displacement ellipsoids of non-hydrogen atoms for compound **[Co-NMe$_2$]BF$_4$** at the 50 % probability level, as derived from the SC-XRD data. Selected interatomic distances [Å]: Co1–C11 2.1702(14), Co1–C12 2.0806(14), Co1–C13 2.0248(15), Co1–C14 2.0172(15), Co1–C15 2.0770(14), Co1–C21 2.1891(14), Co1–C22 2.0807(15), Co1–C23 2.0167(15), Co1–C24 2.0120(15), Co1–C25 2.0864(14).

Crystallographic data for **[Co-NMe$_2$]BF$_4$**.
$C_{19}H_{20}BCoF_4N_2$, plate (0.240 × 0.170 × 0.018 mm^3), formula weight 422.11 g mol^{-1}; monoclinic, $P2_1/c$ (No. 14), a = 10.8499(4) Å, b = 9.2315(3) Å, c = 17.5780(6) Å, β = 102.8754(9)°, V = 1716.36(10) Å^3, Z = 4, Z' = 1, T = 100(2) K, d_{calc} = 1.634 g cm^{-3}, μ(Mo Kα) = 1.047 mm^{-1}, F(000) = 864; T_{max}/T_{min} = 0.9705/0.7785; 107253 reflections were collected (2.506° ≤ θ ≤ 33.727°, index ranges: –16 ≤ h ≤ 16, –14 ≤ k ≤ 14, and –26 ≤ l ≤ 27), 6822 of which were unique, R_{int} = 0.0388, $R_σ$ = 0.0191; completeness to θ of 33.727° 99.6 %. The refinement of 286 parameters with 304 restraints converged to $R1$ = 0.0451 and $wR2$ = 0.1190 for 5786 reflections with $I > 2σ(I)$ and $R1$ = 0.0533 and $wR2$ = 0.1247 for all data with goodness-of-fit S = 1.068 and residual electron density $ρ_{max}/ρ_{min}$ = 1.106/–0.514 e Å^{-3}, rms 0.110 e Å^{-3}; max shift/e.s.d. in the last cycle 0.002.

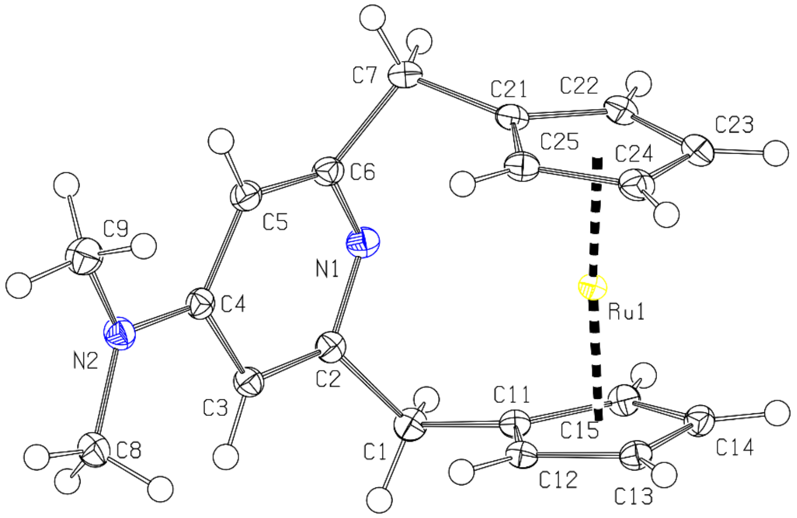

Supplementary Fig. 85. ORTEP showing the anisotropic displacement ellipsoids of non-hydrogen atoms for compound **Ru-NMe2** at the 50 % probability level, as derived from the SC-XRD data. Selected interatomic distances [Å]: Ru1–C11 2.2773(9), Ru1–C12 2.2092(9), Ru1–C13 2.1676(9), Ru1–C14 2.1650(10), Ru1–C15 2.2180(10), Ru1–C21 2.2768(10), Ru1–C22 2.2060(10), Ru1–C23 2.1598(11), Ru1–C24 2.1658(10), Ru1–C25 2.2112(10).

Crystallographic data for **Ru-NMe2**.

$C_{19}H_{20}N2Ru$, prism (0.179 × 0.121 × 0.041 mm^3), formula weight 377.44 g mol^{-1}; monoclinic, $P2_1/n$ (No. 14), a = 9.2034(3) Å, b = 10.7736(3) Å, c = 15.5177(4) Å, β = 100.6261(6)°, V = 1512.25(8) Å^3, Z = 4, Z' = 1, T = 100(2) K, d_{calc} = 1.658 g cm^{-3}, μ(Mo $K\alpha$) = 1.034 mm^{-1}, F(000) = 768; T_{max}/T_{min} = 0.9148/0.7875; 108675 reflections were collected (2.397° ≤ θ ≤ 45.292°, index ranges: –17 ≤ h ≤ 17, –20 ≤ k ≤ 21, and –26 ≤ l ≤ 30), 12193 of which were unique, R_{int} = 0.0437, R_σ = 0.0341; completeness to θ of 45.292° 96.2 %. The refinement of 201 parameters with no restraints converged to $R1$ = 0.0324 and $wR2$ = 0.0707 for 9944 reflections with $I > 2\sigma(I)$ and $R1$ = 0.0429 and $wR2$ = 0.0758 for all data with goodness-of-fit S = 1.055 and residual electron density ρ_{max}/ρ_{min} = 1.668/–0.662 e Å^{-3}, rms 0.123 e Å^{-3}; max shift/e.s.d. in the last cycle 0.004.

Alert level B

```
PLAT973_ALERT_2_B Check Calcd Positive Resid. Density on       Ru1       1.60 eA-3
```

Author Response: Specifically arranged residual electron density peaks and holes are observed, most likely arising from the use of the spherical atomic model for structural refinement, which inherently does not account for electron density deformation.

Supplementary Table 12. List of crystallographically determined Fe–N bond distances of pyridine-based neutral $S = 2$, Fe^{II}PNP pincer complexes.

CCDC number	Fe–N bond distance (Å)	DOI
1937834	2.2334(17)	10.1246/cl.190115
1937835	2.1998(14)	10.1246/cl.190115
715363	2.3286(13)	10.1021/om800425p
2081555	2.201(2)	10.1021/acs.organomet.1c00296
2081556	2.239(3)	10.1021/acs.organomet.1c00296
2081557	2.304(2)	10.1021/acs.organomet.1c00296
285954	2.303(2)	10.1016/j.ica.2005.11.039
2109421	2.3268(11)	10.1021/acs.inorgchem.1c02909
814330	2.1915(16)	10.1021/om1001638
641037	2.2504(18)	10.1021/om060802o
1050950	2.289(3)	10.1002/ejic.201500646
1050951	2.2996(8)	10.1002/ejic.201500646
1445976	2.141(2)	10.1007/s00706-016-1706-x
683350	2.2309(11)	10.1002/anie.200803665
1016572	2.2544(4)	10.1016/j.jorganchem.2014.08.029
1566438	2.304(2)	10.5517/ccdc.csd.cc1pl08k
839105	2.3118(11)	10.1016/j.jorganchem.2014.08.029
839105	2.2714(8)	10.1016/j.jorganchem.2014.08.029
1050104	2.2912(17)	10.1016/j.crci.2015.07.004
1050104	2.2630(14)	10.1016/j.crci.2015.07.004
1050105	2.247(2)	10.1016/j.crci.2015.07.004
1431434	2.281(4)	10.1016/j.poly.2015.12.037
Average	2.26(5)	

Supplementary Table 13. Selected Internuclear Distances (in Å) for **1-H**, **3-H**, and **4-H** in the crystals according to SC-XRD data

Bond	**1-H**	**3-H**	**4-H**
N1–C2	1.3297(11)	1.342(2)	1.358(2)
N1–C6	1.3330(11)	n/a	1.360(2)
C2–C3	1.3958(12)	1.397(3)	1.386(2)
C3–C4	1.3911(15)	1.380(3)	1.385(3)
C4–C5	1.389(2)	n/a	1.384(3)
C5–C6	1.3987(17)	n/a	1.383(3)

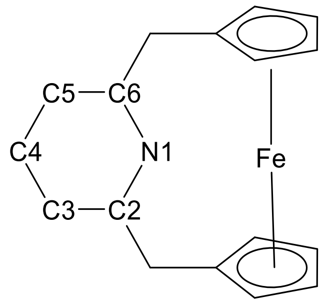

Supplementary Table 13. Methods used to prepare crystals for the SC-XRD study

Complex	Method
**1-H**	Cooling of a concentrated diethyl ether solution to –35 °C
**1-Cl**	Evaporation of a C_6H_6 solution
**1-OMe/2-OMe**	Faster evaporation of a THF solution
**2-OMe**	Slow evaporation of a THF solution
**2-NMe$_2$**	Evaporation of a CH_2Cl_2 solution
**3-H**	Diffusion of a C_6H_6 solution of **1-H** to a CH_2Cl_2 solution of $[FeCp_2]PF_6$
**3-Cl**	Diffusion of a C_6H_6 solution of **1-Cl** to a CH_2Cl_2 solution of $[FeCp_2]PF_6$
**3-OMe**	Cooling of a concentrated CH_2Cl_2 solution to –35 °C
**3-NMe$_2$**	Evaporation of an MeCN solution
**4-H**	Diffusion of C_6H_6 to a $CH_2Cl_2/MeNO_2$ solution
**4-NMe$_2$**	Diffusion of C_6H_6 to a $CH_2Cl_2/MeNO_2$ solution
**4-NMe$_2$-PF$_6$**	Diffusion of a C_6H_6 solution of **2-NMe$_2$** to a CH_2Cl_2 solution of $[FeCp_2]PF_6$
**Co-NMe$_2$**	Concentration of THF solution
**[Co-NMe$_2$]BF$_4$**	Concentration of CH_2Cl_2 solution
**Ru-NMe$_2$**	Concentration of THF solution

9. References

1 Dantignana, V. *et al.* Characterization of a Ferryl Flip in Electronically Tuned Nonheme Complexes. Consequences in Hydrogen Atom Transfer Reactivity. *Angew. Chem. Int. Ed.* **62**, e202211361 (2023).

2 Ramogida, C. F. *et al.* Synthesis and characterization of lipophilic cationic Ga(iii) complexes based on the H_2*CHX*dedpa and H_2dedpa ligands and their $^{67/68}$Ga radiolabeling studies. *RSC Adv.* **6**, 103763-103773 (2016).

3 Fan, R. *et al.* Spectroscopic and DFT Characterization of a Highly Reactive Nonheme FeV–Oxo Intermediate. *J. Am. Chem. Soc.* **140**, 3916-3928 (2018).

4 Bridger, G. J. *et al.* Synthesis and Structure−Activity Relationships of Phenylenebis(methylene)- Linked Bis-azamacrocycles That Inhibit HIV-1 and HIV-2 Replication by Antagonism of the Chemokine Receptor CXCR4. *J. Med. Chem.* **42**, 3971-3981 (1999).

5 Hommes, P., Fischer, C., Lindner, C., Zipse, H. & Reissig, H.-U. Unprecedented Strong Lewis Bases—Synthesis and Methyl Cation Affinities of Dimethylamino-Substituted Terpyridines. *Angew. Chem. Int. Ed.* **53**, 7647-7651 (2014).

6 Bhattacharya, S., Snehalatha, K. & George, S. K. Synthesis of Some Copper(II)-Chelating (Dialkylamino)pyridine Amphiphiles and Evaluation of Their Esterolytic Capacities in Cationic Micellar Media. *J. Org. Chem.* **63**, 27-35 (1998).

7 Karschin, N., Krenek, S., Heyer, D. & Griesinger, C. Extension and improvement of the methanol-d_4 NMR thermometer calibration. *Magn. Reson. Chem.* **60**, 203-209 (2022).

8 Neese, F. The ORCA program system. *WIREs Comput. Mol. Sci.* **2**, 73-78 (2012).

9 Neese, F. Software update: the ORCA program system, version 4.0. *WIREs Comput. Mol. Sci.* **8**, e1327 (2018).

10 Neese, F. Software update: The ORCA program system—Version 5.0. *WIREs Comput. Mol. Sci.* **12**, e1606 (2022).

11 Tao, J., Perdew, J. P., Staroverov, V. N. & Scuseria, G. E. Climbing the Density Functional Ladder: Nonempirical Meta--Generalized Gradient Approximation Designed for Molecules and Solids. *Phys. Rev. Lett.* **91**, 146401 (2003).

12 Staroverov, V. N., Scuseria, G. E., Tao, J. & Perdew, J. P. Comparative assessment of a new nonempirical density functional: Molecules and hydrogen-bonded complexes. *J. Chem. Phys.* **119**, 12129-12137 (2003).

13 Staroverov, V. N., Scuseria, G. E., Tao, J. & Perdew, J. P. Erratum: "Comparative assessment of a new nonempirical density functional: Molecules and hydrogen-bonded complexes" [J. Chem. Phys. 119, 12129 (2003)]. *J. Chem. Phys.* **121**, 11507-11507 (2004).

14 Caldeweyher, E., Bannwarth, C. & Grimme, S. Extension of the D3 dispersion coefficient model. *J. Chem. Phys.* **147**, 034112 (2017).

15 Caldeweyher, E. *et al.* A generally applicable atomic-charge dependent London dispersion correction. *J. Chem. Phys.* **150**, 154122 (2019).

16 Weigend, F. & Ahlrichs, R. Balanced basis sets of split valence, triple zeta valence and quadruple zeta valence quality for H to Rn: Design and assessment of accuracy. *Phys. Chem. Chem. Phys.* **7**, 3297-3305 (2005).

17 Neese, F. Importance of Direct Spin−Spin Coupling and Spin-Flip Excitations for the Zero-Field Splittings of Transition Metal Complexes: A Case Study. *J. Am. Chem. Soc.* **128**, 10213-10222 (2006).

18 Marenich, A. V., Cramer, C. J. & Truhlar, D. G. Universal Solvation Model Based on Solute Electron Density and on a Continuum Model of the Solvent Defined by the

Bulk Dielectric Constant and Atomic Surface Tensions. *J. Phys. Chem. B* **113**, 6378-6396 (2009).
19 Helmich-Paris, B., de Souza, B., Neese, F. & Izsák, R. An improved chain of spheres for exchange algorithm. *J. Chem. Phys.* **155**, 104109 (2021).
20 Lu, T. & Chen, F. Multiwfn: A multifunctional wavefunction analyzer. *J. Comput. Chem.* **33**, 580-592 (2012).
21 Kesharwani, M. K., Brauer, B. & Martin, J. M. L. Frequency and Zero-Point Vibrational Energy Scale Factors for Double-Hybrid Density Functionals (and Other Selected Methods): Can Anharmonic Force Fields Be Avoided? *J. Phys. Chem. A* **119**, 1701-1714 (2015).
22 Hansch, C., Leo, A. & Taft, R. W. A survey of Hammett substituent constants and resonance and field parameters. *Chem. Rev.* **91**, 165-195 (1991).
23 Rumble, J. R., Bruno, T. J. & Doa, M. J. *CRC handbook of chemistry and physics : a ready-reference book of chemical and physical data*. 101st edition edn, CRC Press/Taylor & Francis Group, (2020).
24 Lu, T. A comprehensive electron wavefunction analysis toolbox for chemists, Multiwfn. *J. Chem. Phys.* **161**, 082503 (2024).
25 Zhang, J. & Lu, T. Efficient evaluation of electrostatic potential with computerized optimized code. *Phys. Chem. Chem. Phys.* **23**, 20323-20328 (2021).
26 Müller, A. M. K. Explicit approximate relation between reduced two- and one-particle density matrices. *Phys. Lett. A* **105**, 446-452 (1984).
27 Kartashov, S. V., Shteingolts, S. A., Stash, A. I., Tsirelson, V. G. & Fayzullin, R. R. Electronic and Crystal Packing Effects in Terms of Static and Kinetic Force Field Features: Picolinic Acid N-Oxide and Methimazole. *Cryst. Growth Des.* **23**, 1726-1742 (2023).
28 Kartashov, S. V., Fedonin, A. P. & Fayzullin, R. R. Electronic Force Density Fields: Insights into Partial Bonds, Transition States, and Chemical Structure Evolution. *J. Phys. Chem. A* **128**, 7471-7488 (2024).
29 Saifina, A. F., Kartashov, S. V., Stash, A. I., Tsirelson, V. G. & Fayzullin, R. R. Unified Picture of Interatomic Interactions, Structures, and Chemical Reactions by Means of Electrostatic and Kinetic Force Density Fields: Appel's Salt and Its Ion Pairs. *Cryst. Growth Des.* **23**, 3002-3018 (2023).
30 Kartashov, S. V., Saifina, A. F. & Fayzullin, R. R. Toward the Chemical Structure of Diborane: Electronic Force Density Fields, Effective Electronegativity, and Internuclear Turning Surface Properties. *J. Phys. Chem. A* **128**, 7284-7297 (2024).
31 Zhao, D.-X. & Yang, Z.-Z. Investigation of the distinction between van der Waals interaction and chemical bonding based on the PAEM-MO diagram. *J. Comput. Chem.* **35**, 965-977 (2014).
32 Bader, R. F. W. & Stephens, M. E. Spatial localization of the electronic pair and number distributions in molecules. *J. Am. Chem. Soc.* **97**, 7391-7399 (1975).
33 Fradera, X., Austen, M. A. & Bader, R. F. W. The Lewis Model and Beyond. *J. Phys. Chem. A* **103**, 304-314 (1999).
34 Sheldrick, G. SHELXT - Integrated space-group and crystal-structure determination. *Acta Crystallogr. A* **71**, 3-8 (2015).
35 Sheldrick, G. Crystal structure refinement with SHELXL. *Acta Crystallogr. C* **71**, 3-8 (2015).
36 Spek, A. PLATON SQUEEZE: a tool for the calculation of the disordered solvent contribution to the calculated structure factors. *Acta Crystallogr. C* **71**, 9-18 (2015).
